# iDNA-Prot: Identification of DNA Binding Proteins Using Random Forest with Grey Model

**DOI:** 10.1371/journal.pone.0024756

**Published:** 2011-09-15

**Authors:** Wei-Zhong Lin, Jian-An Fang, Xuan Xiao, Kuo-Chen Chou

**Affiliations:** 1 Information Science and Technology School, Donghua University, Shanghai, China; 2 Computer Department, Jing-De-Zhen Ceramic Institute, Jing-De-Zhen, China; 3 Gordon Life Science Institute, San Diego, California, United States of America; University of South Florida College of Medicine, United States of America

## Abstract

DNA-binding proteins play crucial roles in various cellular processes. Developing high throughput tools for rapidly and effectively identifying DNA-binding proteins is one of the major challenges in the field of genome annotation. Although many efforts have been made in this regard, further effort is needed to enhance the prediction power.

By incorporating the features into the general form of pseudo amino acid composition that were extracted from protein sequences via the “grey model” and by adopting the random forest operation engine, we proposed a new predictor, called **iDNA-Prot**, for identifying uncharacterized proteins as DNA-binding proteins or non-DNA binding proteins based on their amino acid sequences information alone. The overall success rate by **iDNA-Prot** was 83.96% that was obtained via jackknife tests on a newly constructed stringent benchmark dataset in which none of the proteins included has 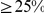 pairwise sequence identity to any other in a same subset. In addition to achieving high success rate, the computational time for **iDNA-Prot** is remarkably shorter in comparison with the relevant existing predictors. Hence it is anticipated that **iDNA-Prot** may become a useful high throughput tool for large-scale analysis of DNA-binding proteins.

As a user-friendly web-server, **iDNA-Prot** is freely accessible to the public at the web-site on http://icpr.jci.edu.cn/bioinfo/iDNA-Prot or http://www.jci-bioinfo.cn/iDNA-Prot. Moreover, for the convenience of the vast majority of experimental scientists, a step-by-step guide is provided on how to use the web-server to get the desired results.

## Introduction

DNA-binding proteins play a vitally important role in many biological processes, such as recognition of specific nucleotide sequences, regulation of transcription, and regulation of gene expression. At present, several experimental techniques (such as filter binding assays, genetic analysis, chromatin immunoprecipitation on microarrays, and X-ray crystallography) have been used for identifying DNA-binding proteins. Although these techniques can provide a detailed picture about the binding, they are both time-consuming and expensive [1]. Particularly, the number of newly discovered protein sequences has been increasing extremely fast. For example, in 1986 the Swiss-Prot [2] database contained only 3,939 protein sequence entries, but now the number has jumped to 530,264 according to the release 2011_07 on 28-Jun-2011 by the UniProtKB/Swiss-Prot at http://web.expasy.org/docs/relnotes/relstat.html, meaning that the number of protein sequence entries now is more than 134 times the number from about 25 years ago. Facing the avalanche of new protein sequences generated in the postgenomic age, it is highly desired to develop automated methods for rapidly and effectively identifying and characterizing DNA-binding proteins based on the protein sequence information alone.

Actually, numerous predictors were developed in this regard. For instance: Shanahan et al. [3] demonstrated how structural features were employed to determine whether a protein of known structure and unknown function was a DNA-binding proteins or not; Ahmad et al. [4] depicted how to distinguish DNA-binding and non DNA-binding proteins with net charge, net dipole moment and quadrupole moment, respectively; Nordhoff et al. [5] introduced identification and characterization of DNA-binding proteins by mass spectrometry, which was regarded as the most sensitive and specific analytical technique available for protein identification [6]. All the aforementioned methods were considerably relied on the results from biochemical experiments. Among the existing methods, those which are purely based on theoretical approaches are of using various classifying engines, such as support vector machine (SVM) [6], [7], [8], [9], [10], [11], [12], [13], [14], [15], artificial neural network (ANN) [16], [17], [18], [19], [20], [21], random forest [22], [23], [24], nearest neighbor [25], and boosted decision trees [26].

In addition to using different prediction engines, a remarkable difference among the existing methods is in that different features were extracted to represent protein samples. For instance, Bhardwaj [13] used a 40-D (dimensional) feature vector to formulate a protein sample that contains positive potential surface patches, overall charge of the protein, and overall surface composition. Yu et al. [10] constructed a 132-D feature vector to represent a protein sequence by using the information of hydrophobicity, predicted secondary structure, solvent accessibility, normalized van der Waals volume, polarity, and polarizability. Bhardwaj and Lu [9] represented the sample of a protein by harnessing the 70 features of the DNA-binding residues, including the residue's identity, charge, solvent accessibility, average potential, secondary structure, neighboring residues, and location in a cationic patch. Kumar et al. [14] extracted the features from the PSSM (Position-Specific Scoring Matrix) profile obtained from PSI-BLAST [27] to represent the protein sample. Subsequently, a different approach was proposed [22] to encode each protein sequence with 116 features by incorporating various physic-chemical properties of amino acids. Meanwhile, Nanni and Lumini [15] proposed a method to represent a protein sample by combing ontologies and dipeptide composition. Later, the same authors [6] introduced the grouped weight to represent protein samples for predicting DNA-binding proteins. Langlois and Lu [1] represented a protein sample with 472 features, of which 240 were secondary structure features, 231 dipeptide composition features, and one for the total charge over its amino acid sequence.

However, the existing predictors have the following shortcomings. (**1**) The extracting features are very complicated and their dimensions are too large. Particularly, during the prediction process, some of the existing predictors even need the informations of query proteins that were obtained from other experiments, such as their three-dimensional (3D) structures, functions, and the other relevant knowledge. (**2**) The computational time needed by these predictors is usually very long; for instance, the predictor **iDBPs** [23] would usually take about 30 minutes for predicting one query protein. (**3**) Most predictors did not provide a web-server for the public usage, while the others claimed they did but are currently not in working condition and hence their practical application value is quite limited.

In view of this, the present study was initiated in an attempt to develop a new and more powerful predictor by addressing the aforementioned three problems.

According to a recent comprehensive review [28], to establish a really useful statistical predictor for a protein system, we need to consider the following procedures: (i) construct or select a valid benchmark dataset to train and test the predictor; (ii) formulate the protein samples with an effective mathematical expression that can truly reflect their intrinsic correlation with the attribute to be predicted; (iii) introduce or develop a powerful algorithm (or engine) to operate the prediction; (iv) properly perform cross-validation tests to objectively evaluate the anticipated accuracy of the predictor; (v) establish a user-friendly web-server for the predictor that is accessible to the public. Below, let us describe how to deal with these steps.

## Materials and Methods

### 1. Benchmark datasets

DNA-binding protein sequences were collected from the Protein Data Bank (PDB) release 03-May-2011 at http://www.pdb.org/, in which there are 72,838 structures. By searching the keywords of “Protein-DNA complex” and “DNA binding” through the “Advanced Search Interface”, we extracted 3,689 structures from (PDB).

To construct a high quality benchmark dataset with a wider coverage scope and lower homology bias, the data obtained above were screened strictly according to the following criteria. (**1**) Sequences with less than 50 amino acid (AA) residues were removed because they might just belong to fragments [29]. (**2**) Sequences with more than 10 consecutive character of “X” were taken away because they contained too many unknown amino acids. (**3**) To reduce redundancy and homology bias, the PISCES [30], [31] was utilized to cutoff those sequences that have 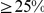 pairwise sequence identity to any other in the dataset. Finally, we obtained 212 DNA-binding proteins. Similarly, 212 non DNA-binding protein domains were randomly picked from the data bank. Accordingly, the benchmark dataset 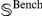 thus obtained consists of 424 protein sequences of which half are DNA-binding protein sequences and the other half non-binding protein sequences. Their accession codes and sequences are given in Information S1.

### 2. A novel pseudo amino acid composition of grey model

To develop a powerful predictor for a protein system, one of the keys is to formulate the protein samples with an effective mathematical expression that can truly reflect their intrinsic correlation with the attribute to be predicted [28]. To realize this, the concept of pseudo amino acid composition (PseAAC) was proposed [32] to replace the simple amino acid composition (AAC) for representing the sample of a protein. According to Eq. 6 of [28], the general form of PseAAC for a protein 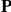 can be formulated as
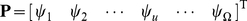
(1)where 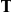 is a transpose operator, while the subscript 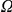 is an integer and its value as well as the components 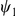, 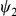, … will depend on how to extract the desired information from the amino acid sequence of 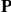.

In this study, we are to use the grey model parameters to define the 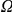 elements in Eq. 1. In 1989, Deng [33] proposed a grey system theory to investigate the uncertainty of a system. According to this theory, if the information of a system investigated is fully known, it is called a “white system”; if completely unknown, a “black system”; if partially known, a “grey system”. The model developed on the basis of such a theory is called “grey model”, which is a kind of nonlinear and dynamic model formulated by a differential equation. The grey model is particularly useful for solving complicated problems that are lack of sufficient information, or need to process uncertain information and reduce random effects of acquired data.

In the grey system theory, an important and generally used model is called GM(1,1). It is quite effective for monotonic series, with good simulating effect and small error, as reflected by the fact that using the GM(1,1) model has remarkably improved the success rates in predicting protein structural classes [34]. However, if the series concerned are not monotonic, the simulating effect of GM(1,1) would not be good and its error might be quite large.

To overcome the above problem, the grey system theory used in the current study is a special grey dynamic model called GM(2,1), which can be used to handle the oscillation series. In GM(2,1) the strategy of minimum squares will be adopted to determine the uncertain parameters, as can be briefly described below.

One of the most commonly used approaches in the grey system theory is the accumulative generation operation (AGO), which can convert a series without any obvious regularity into a strict monotonic increasing series so as to reduce the randomness and enhance the smoothness of the series, and minimize interference from the random information. Let us assume that 
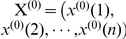
 is the original series of real numbers with an irregular distribution, and it is a non-negative original data sequence. Then, 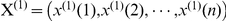 is viewed as the first-order accumulative generation operation (1-AGO) series for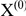; i.e., the components in 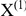 are given by
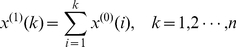
(2)The GM(2,1) model can be expressed by the following second-order grey differential equation with one variable:
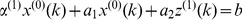
(3)where:
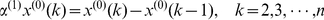
(4)

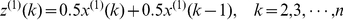
(5)In Eq. 3, the coefficients 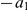
and 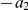 are the developing coefficients, and 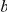 the influence coefficient. Then we have
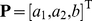
(6)Thus, it follows by the least-squares method that
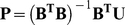
(7)where
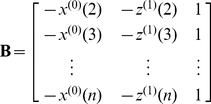
(8)

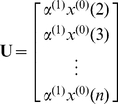
(9)The least-square estimator for the coefficients 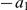, 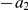 and 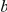 should carry some intrinsic information contained in the discrete data sequence 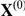 sampled from the system investigated. In view of this, the incorporation of these coefficients into the general form of PseAAC (Eq. 1) will make the formulation of a protein sample better to reflect its intrinsic correlation with the attribute to be predicted. This is the key of the novel approach. The concrete procedures are as follows.

A protein sequence is composed of 20 different types of native amino acids denoted by A, C, D, E, F, G, H, I, K, L, M, N, P, Q, R, S, T, V, W and Y. Before using the grey dynamic model GM(2,1), we need to represent the protein sequence by a series of real numbers. Listed in **Table 1** is the numerical codes used in this study to represent the 20 amino acids.

**Table 1:** The numerical codes of 20 native amino acids.

Amino acid	Factor score [35]	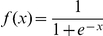
A	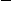 0.733	0.325
C	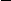 0.862	0.297
D	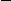 3.656	0.025
E	1.477	0.814
F	1.891	0.869
G	1.330	0.791
H	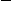 1.673	0.158
I	2.131	0.849
K	0.533	0.630
L	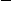 1.505	0.182
M	2.219	0.902
N	1.299	0.720
P	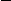 1.628	0.164
Q	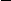 3.005	0.047
R	1.502	0.818
S	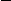 4.760	0.008
T	2.213	0.901
V	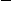 0.544	0.367
W	0.672	0.662
Y	3.097	0.957

The factor score for molecular volume are adopted [35] that is related to the molecular size or volume as well as side chain weight [35]. Because in the current study, only the non-negative sequences will be considered, we can adopt the following function for modeling
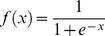
(10)Through the above function, each of the 20 amino acids can be translated into numerical variable within the region of (0, 1) (**Table 1**). With the numerical codes thus obtained, we can convert a protein sequence to a series of real numbers. Thus, the three coefficients 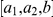 for any protein sequence can be derived with the grey model GM(2,1) by following Eqs.2–9.

### 3. Predicting algorithm

The three coefficients obtained in the above section, in addition to the 20 components in the classical amino acid composition [36], can be used to form a new mode of PseAAC, with 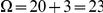 components. Thus, according Eq. 1, the protein 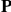 can be formulated with a new mode of PseAAC as given by
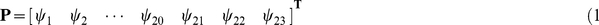
(11)where 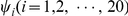 are the occurrence frequencies of the 20 different types of amino acids in the protein concerned, while 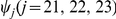 represent the absolute value of coefficients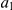,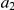 and 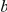, respectively.

Now the Random Forest (RF) algorithm was adopted to perform the prediction. RF is a popular machine learning algorithm and recently it has been successfully employed in dealing with various biological prediction problems [37], [38], [39], [40]. RF is a combination of tree predictors such that each tree depends on the values of a random vector sampled independently and with the same distribution for all trees in the forest. It has been shown that combining multiple trees produced in randomly selected subspaces can significantly improve the prediction accuracy. RF performs a type of cross-validation by using out-of-bag samples. During the training process, each tree is constructed using a different bootstrap sample from the original data. For the detailed description about of the RF algorithm, refer to the papers [41], [42], [43].

The RF algorithm is available via the link at http://www.stat.berkeley.edu/~breiman/RandomForests/cc_home.htm. Recently, the RF tool for the MATLAB windows is also available at http://code.google.com/p/randomforest-matlab/that has two important functions: one is “classRF_train” for training given data and returning the prediction model, and the other is “classRF_predict” for predicting query input with the prediction model. The classifier in this paper was developed based on the RF tool for the MATLAB windows.

The classifier thus established is called **iDNA-Prot**, which can be used to predict whether a protein can bind with DNA according to its sequence information alone.

For practical applications, a web-server of **iDNA-Prot** was established at the web-site http://icpr.jci.edu.cn/bioinfo/iDNA-Prot. Moreover, for the convenience of the vast majority of experimental scientists, a step-by-step guide on how to use the web-server is given in Information S3, by which users can easily get their desired results without the need to follow the complicated mathematic equations involved in developing the **iDNA-Prot** predictor.

## Results and Discussion

In statistical prediction, the following three cross-validation methods are often used to examine a predictor for its effectiveness in practical application: independent dataset test, subsampling test, and jackknife test [44]. However, of the three test methods, the jackknife test is deemed the most objective [45]. The reasons are as follows. (1) For the independent dataset test, although all the proteins used to test the predictor are outside the training dataset used to train it so as to exclude the “memory” effect or bias, the way of how to select the independent proteins to test the predictor could be quite arbitrary unless the number of independent proteins is sufficiently large. This kind of arbitrariness might result in completely different conclusions. For instance, a predictor achieving a higher success rate than the other predictor for a given independent testing dataset might fail to keep so when tested by another independent testing dataset [44]. (2) For the subsampling test, the concrete procedure usually used in literatures is the 5-fold, 7-fold or 10-fold cross-validation. The problem with this kind of subsampling test is that the number of possible selections in dividing a benchmark dataset is an astronomical figure even for a very simple dataset, as demonstrated by Eqs.28–30 in [28]. Therefore, in any actual subsampling cross-validation tests, only an extremely small fraction of the possible selections are taken into account. Since different selections will always lead to different results even for a same benchmark dataset and a same predictor, the subsampling test cannot avoid the arbitrariness either. A test method unable to yield a unique outcome cannot be deemed as a good one. (3) In the jackknife test, all the proteins in the benchmark dataset will be singled out one-by-one and tested by the predictor trained by the remaining protein samples. During the process of jackknifing, both the training dataset and testing dataset are actually open, and each protein sample will be in turn moved between the two. The jackknife test can exclude the “memory” effect. Also, the arbitrariness problem as mentioned above for the independent dataset test and subsampling test can be avoided because the outcome obtained by the jackknife cross-validation is always unique for a given benchmark dataset. Accordingly, the jackknife test has been increasingly and widely used by those investigators with strong math background to examine the quality of various predictors (see, e.g.,[46], [47], [48], [49], [50], [51], [52], [53], [54], [55], [56], [57]). In view of this, here the jackknife cross-validation was also used to examine the prediction quality of the current predictor.

The results thus obtained with **iDNA-Prot** on the benchmark dataset 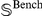 of Information S1 is given in **Table 2**, from which we can see that the overall success rate was 83.96% in identifying proteins as DNA-binding proteins and non-DNA-binding proteins.

**Table 2:** Results obtained by **iDNA-Prot** on the benchmark dataset of Information S1 through the jackknife test^a^.

Protein type	Number of proteins	Number of correct prediction	Success rate
DNA-binding protein	212	179	84.43%
Non DNA-binding protein	212	177	83.49%
Overall	424	356	83.96%

a The following parameters were used for Random Forest algorithm: the number of tree grown was 560 and the number of predictors sampled for splitting at each node was 5.

Furthermore, as a demonstration to show that the current predictor **iDNA-Prot** is superior to the existing ones, let us compare **iDNA-Prot** with **DNA-Prot** [22]. The reason we chose **DNA-Prot** for comparison is because among the existing methods for predicting DNA-binding proteins, the reported success rate achieved by **DNA-Prot** [22] is the highest. The datasets used to train and test **DNA-Prot** [22] as well as its standalone version can be obtained from http://www3.ntu.edu.sg/home/EPNSugan/index_files/dnaprot.htm.

The training dataset 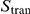 used for **DNA-Prot** [22] contains 146 DNA-binding proteins and 250 non-DNA-binding proteins.

The data used to test **DNA-Prot** [22] contain the following three sets: (i) testing dataset-1, 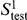, consisting of 92 DNA-binding proteins and 100 non DNA-binding proteins; (ii) testing dataset-2, 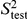, consisting of 823 DNA-binding proteins and 823 non DNA-binding proteins; and (iii) testing dataset-3, 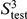, consisting of 88 DNA-binding proteins and 233 non DNA-binding proteins. All these datasets were elaborated in [22].

However, it was found (see Information S2) that there are 10 identical protein sequences between the 146 DNA-binding proteins in the training dataset 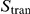 and the 92 DNA-binding proteins in the 1^st^ testing dataset 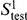, that 19 identical protein sequences between the 146 DNA-binding proteins in the training dataset 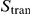 and the 88 DNA-binding proteins in the 3rd testing dataset 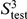, and that 94 identical protein sequences between the 250 non-DNA-binding proteins in the training dataset 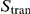 and the 233 non-DNA-binding proteins in the 3rd testing dataset 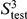. In other words, the so-called independent datasets used by **DNA-Prot** [22] were actually not independent and hence would lead to over-estimated success rates.

Accordingly, to perform an objective and fair comparison of **iDNA-Prot** with **DNA-Prot** [22], let us construct a real independent dataset by randomly picking some DNA-binding proteins and non DNA-binding proteins from PDB (Protein Data Bank) according to the following criteria. These proteins must not occur in the training dataset of **iDNA-Prot** nor in the training dataset for **DNA-Prot** [22], and that none of the proteins included has more than 40% sequence identity to any other in a same subset. By doing so, we obtained a real independent dataset 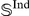, in which 122 proteins are DNA-binding proteins and 122 non DNA-binding proteins. The sequences and accession numbers for such 244 independent proteins are given in the Information S4.

Listed in **Table 3** are the tested results obtained respectively by **DNA-Prot** [22] and **iDNA-Prot** on the 244 independent proteins in 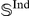
(Information S4). From the table we can see that the success rates by **iDNA-Prot** in identifying both DNA-binding and non-DNA-binding proteins are remarkably higher than those by **DNA-Prot** [22], and that the overall success rate achieved by **iDNA-Prot** is about 13% higher than that by **DNA-Prot** [22].

**Table 3:** A comparison of the predicted results by **DNA-Prot** [22] and **iDNA-Prot** on the independent dataset in the Information S3.

Protein type	DNA-Prot [22]	iDNA-Prot
DNA-binding protein	87/122 = 71.31%	109/122 = 89.34%
Non DNA-binding protein	101/122 = 82.79%	111/122 = 90.98%
Overall	188/244 = 77.05%	220/244 = 90.16%

In addition to yielding higher success rates than those by the relevant existing predictors, the computational time needed by **iDNA-Prot** to complete a prediction is also significantly shorter than any of its counterparts, and hence **iDNA-Prot** may become a useful high throughput tool for large-scale investigation of DNA-binding proteins.

Moreover, as a demonstration to show the efficiency of the current method, the hypothetical proteins that are annotated as DNA-binding proteins were used to test **iDNA-Prot**. The information about this kind of hypothetical proteins can be obtained at http://www.ncbi.nlm.nih.gov/protein/?term=DNAbindinghypothetical, from which we randomly picked 100 DNA-binding hypothetical proteins for test. The results predicted by **iDNA-Prot** on these proteins are given in **Table 4**, from which we can see the overall success rate is 90%.

**Table 4:** The predicted results by **iDNA-Prot** on the 100 DNA-binding hypothetical proteins from http://www.ncbi.nlm.nih.gov/protein/?term=DNAbindinghypothetical.

GI code	Predicted result	GI code	Predicted result	GI code	Predicted result
21960164	DBP	29122980	non DBP	26832636	DBP
21957418	DBP	21671920	DBP	26832400	DBP
21961058	DBP	32880245	DBP	21835917	DBP
21960858	DBP	90578605	DBP	21835539	DBP
21960204	DBP	90410315	DBP	21843120	DBP
21958545	DBP	89076244	non DBP	21836833	DBP
21958841	non DBP	23326729	non DBP	14627522	DBP
14828174	DBP	90439438	DBP	78363301	DBP
52629876	DBP	90328556	DBP	30116886	DBP
21958534	DBP	89048073	non DBP	20673954	DBP
21958313	DBP	30724697	DBP	88595361	DBP
21957779	non DBP	30726408	DBP	15769834	DBP
21958822	DBP	30726252	DBP	68057023	DBP
21957238	DBP	30725976	DBP	59480370	non DBP
1552778	DBP	30725598	DBP	52004347	DBP
21960397	DBP	30725306	DBP	11114707	DBP
21960196	DBP	30725067	DBP	22984739	DBP
21960777	DBP	30724845	DBP	22984549	DBP
21960121	DBP	16882676	DBP	14564202	DBP
21960008	DBP	30687056	DBP	14563738	non DBP
21959358	non DBP	30686776	DBP	14563550	DBP
21959322	DBP	30686615	DBP	90406920	DBP
21959035	DBP	30686107	DBP	14563368	DBP
21958991	DBP	30685947	DBP	29434364	DBP
21957969	DBP	30685727	DBP	23327083	non DBP
21957386	DBP	30685502	DBP	93211002	DBP
21956952	DBP	30685211	DBP	14498544	DBP
21877200	DBP	30977777	DBP	14526726	DBP
26832542	DBP	18859138	DBP	14527329	DBP
26832403	DBP	52002457	DBP	22981160	DBP
33989345	DBP	17093877	DBP	46913396	DBP
33989345	DBP	30891446	DBP	71382240	DBP
33875610	DBP	26832638	DBP		
52004491	DBP	26832637	DBP		

## Supporting Information

Information S1
**The benchmark dataset** 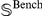 **includes 424 proteins, classified into 212 DNA-binding proteins and 212 non DNA-binding proteins.** Both the accession identifier of PDB (Protein Data Bank) and sequences are given. None of the proteins has more than 25% sequence identity to any other in a same subset. See the text of the paper for further explanation.(PDF)

Information S2 **List of protein codes that occur in both the training and testing datasets for DNA-Prot (Kumar et al., 2009).** See the main paper for further explanation.
(PDF)

Information S3 **A step-by-step guide on how to use the web-server of iDNA-Prot to get the desired results.**
(PDF)

Information S4
**The independent dataset** 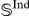 **includes 244 proteins, classified into 122 DNA-binding proteins and 122 non DNA-binding proteins.** Both the accession identifier of PDB (Protein Data Bank) and sequences are given. None of the proteins has more than 40% sequence identity to any other in a same subset. See the text of the paper for further explanation.(PDF)
