# Supplementary material for: iDNA-Prot: Identification of DNA Binding Proteins Using Random Forest with Grey Model
Source: PLoS One. 2011 Sep 15;6(9):e24756. doi: 10.1371/journal.pone.0024756 (PMC3174210; doi:10.1371/journal.pone.0024756)
Supplement: Information S1 — The benchmark dataset includes 424 proteins, classified into 212 DNA-binding proteins and 212 non DNA-binding proteins. Both the accession identifier of PDB (Protein Data Bank) and sequences are given. None of the proteins has more than 25% sequence identity to any other in a same subset. See the text of the paper for further explanation. (PDF) [file pone.0024756.s001.pdf]

Online Supporting Information S1. The benchmark dataset $\mathbb{S}^{\text{Bench}}$ includes 424 proteins, classified into 212 DNA-binding proteins and 212 non DNA-binding proteins. Both the accession identifier of PDB (Protein Data Bank) and sequences are given. None of the proteins has more than 25% sequence identity to any other in a same subset. See the text of the paper for further explanation.

---

(1) 212 DNA-binding proteins

>2WQIA
GSDEDTYYLQVRGRENFEILMKLKESLELMELVPQPLVDSYRQQQQLLQRP
>3F8MA
GSGAVTAGAAPRILKHQVVRAELDRMLDGMRIGDPFPAEREIAEQFEVARETVRQALRELLIDGRV
ERRGRTTVVARPKIRQPLGMGSYTEAAKAQGLSAGRILVAWSDLTADEVLAGVLGVDVGAPVLQLE
RVLTTDGVRVGLETTKLPAQRYPGLRETFDHEASLYAEIRSRGIAFTRTVDTIDTALPDAREAALL
GADARTPMFLLNRVSYDQDDVAIEQRRSLYRGDRMTFTAVMHAKNSAIVS
>3F8TA
MFRLGTTPRATTYDPDARIGEVASRFGLPTRVLIEIVRTESFQRSLARVTSGKPVVLDLRELDSDL
ASWIATHARLVEPALRELVRTVAPDVEPRVRFRGLPHRFRRVERIRPMDGALISIEGVVREVRGAE
RLEHAIVDTGSELVAVRLHGHRLGPGLRVEILGIVRSATLDALEVHKKDPIPEVHPDPAELEEFRE
LADKDPLTTFARAIAPLPGAEEVGKMLALQLFSCVGKNSERLHVLLAGYPVVCSEILHHVLDHLAP
RGVYVDLRRTELTDLTAVLKEDRGWALRAGAAVLADGGILAVDHLEGAPEPHRWALMEAMDKGTVT
VDGIALNARCAVLAAINPGEQWPSDPPIARIDLDQDFLSHFDLIAFLGVDPRPGEPEEQDTEVPSY
TLLRRYLLYAIREHPAPELTEEARKRLEHWYETRREEVEERLGMGLPTLPVTRRQLESVERLAKAH
ARMRLSDDVEPEDVDIAAELVDWYLETAMQIPGGDEIRISSLKP
>2FQZA
MQRLSPGEFKTLISKERKSHFITPFALVYKTFCDLGYDQKNSDYFLNNPSEYIIAMRKNCWKEFEP
FEKEFTTRMLSYLIDEERIKDMSPYDAIRDFTMEYPTHIYDLALSNTQSRRSRAGKEFESILELLM
MGAGIPVDVQGAIGKSFFQKNQIGKLVDLVMPGVVQYTSNKRNTMLISAKTTLRERWQEVPEEVNR
TGIREMYLATLDDSFSEETINILYEANVVVVTTVENKNFKYKNNNRVLTFEDMLQSAMELSRKWNN
VSYTDSEKEEIQQSILKQIEKYSDFPYVVNYYRNRLSALFD
>2ZQEA
MREVKEVDLRGLTVAEALLEVDQALEEARALGLSTLRLLHGKGTGALRQAIREALRRDKRVESFAD
APPGEGGHGVTVVALRP
>1R7JA
AKKKSKLEIIQAILEACKSGSPKTRIMYGANLSYALTGRYIKMLMDLEIIRQEGKQYMLTKKGEEL
LEDIRKFNEMRKNMDQLKEKINSVLSIRQ
>3M4AA
MPSRTELLAEEYLRREGHDPQKFRYVHPDGTPYTDADGLARIARLAVPPAYQDVYVSPDAENELQA
FGRDAAGRLQYRYHPDFVQAGALKKWQRLTRFAGALPTLKVATTADLRASGLPPRKVMALMTRLLH
VARFRVGSDIYARQHKTYGLSTLRQRHVVVDGNTVTFRFKGKHGVSQHKATSDRTLAANMQKLLDL
PGPWLFQTVDAGGGERRRIHSTELNAYLREVIGPFTAKDFRTWGGTLLAAEYLAQQGTESSERQAK
KVLVDCVKFVADDLGNTPAVTRGSYICPVIFDRYLDGKVLDDYEPRTERQEAELEGLTRSEGALKR
MLESERTLRQRGRALK
>2FE3A

MAAHELKEALETLKETGVRITPQRHAILEYLVNSMAHPTADDIYKALEGKFPNMSVATVYNNLRVF
RESGLVKELTYGDASSRFDFVTSDHYHAICENCGKIVDFHYPGLDEVEQLAAHVTGFKVSHHRLEI
YGVCQECSKKENH
>2I13A
ISEFGSSSSVAQAALEPGEKPYACPECGKSFSRSDHLAEHQRTHTGEKPYKCPECGKSFSDKKDLT
RHQRTHTGEKPYKCPECGKSFSQRANLRAHQRTHTGEKPYACPECGKSFSQLAHLRAHQRTHTGEK
PYKCPECGKSFSREDNLHTHQRTHTGEKPYKCPECGKSFSRRDALNVHQRTHTGKKTS
>1R9WA
KQGAMLAVFKDTYGLSFTDLVRNFKSDKTTCTDWVTAIFGVNPTIAEGFKTLIQPFILYAHIQCLD
CKWGVLILALLRYKCGKSRLTVAKGLSTLLHVPETCMLIQPPKLRSSVAALYWYRTGISNISEVMG
DTPEWIQRLTIIQ
>3FBLA
YHKLRLAIKEICKTDGIPNIKWGMYIAFGEKLLKSYLKMKAGSASSDMIAEYINNAISAFSSRTGI
SQETAQKIADFITSNY
>2XSEA
TNLMVSTAVEKKKYLDSEFLLHCISAQLLDMWKQARARWLELVGKEWAHMLALNPERKDFLWKNQS
EMNSAFFDLCEVGKQVMLGLLGKEVALPKEEQAFWIMYAVHLSAACAEELHMPEVAMSLRKLNVKL
KDFNFGGTRYFKDMPPEEKKRRMERKQRIEEARRHGMP
>2HANB
GSAPRVQEELCLVCGDRASGYHYNALTCEGCKGFFRRSVTKSAVYCCKFGRACEMDMYMRRKCQEC
RLKKCLAVGMRPECVVPENQCAMKRREKKAQKEKDKMTTSPSSQHGSPGIHRD
>2HAXA
MQRGKVKWFNNEKGYGFIEVEGGSDVFVHFTAIQGEGFKTLEEGQEVSFEIVQGNRGPQAANVVKL
>2YVAA
MQERIKACFTESIQTQIAAAEALPDAISRAAMTLVQSLLNGNKILCCGNGTSAANAQHFAASMINR
FETERPSLPAIALNTDNVVLTAIANDRLHDEVYAKQVRALGHAGDVLLAISTRGNSRDIVKAVEAA
VTRDMTIVALTGYDGGELAGLLGPQDVEIRIPSHRSARIQEMHMLTVNCLCDLIDNTLFPHQDD
>1AE2A
MIKVEIKPSQAQFTTRSGVSRQGKPYSLNEQRCYVDLGNEYPVLVKITLDEGQPAYAPGLYTVHLS
SFKVGQFGSLMIDRLRLVPAK
>2A1IA
MGSSHHHHHHSQDPAKSNSIIVSPRQRGNPVLKFVRNVPWEFGDVIPDYVLGQSTCALFLSLRYHN
LHPDYIHGRLQSLGKNFALRVLLVQVDVKDPQQALKELAKMCILADCTLILAWSPEEAGRYLETYK
AYEQKPADLLMEKL
>2RIQA
MVDEVAKKKSKKEKDKDSKLEKALKAQNDLIWNIKDELKKVCSTNDLKELLIFNKQQVPSGESAIL
DRVADGMVFGALLPCEECSGQLVFKSDAYYCTGDVTAWTKCMVKTQTPNRKEWVTPKEFREISYLK
KLKVKKQDRIFPPETSASVALEHHHHHH
>2C2FA
MTKKSTKSEAASKTKKSGVPETGAQGVRAGGADHADAAHLGTVNNALVNHHYLEEKEFQTVAETLQ
RNLATTISLYLKFKKYHWDIRGRFFRDLHLAYDEFIAEIFPSIDEQAERLVALGGSPLAAPADLAR
YSTVQVPQETVRDARTQVADLVQDLSRVGKGYRDDSQACDEANDPVTADMYNGYAATIDKIRWMLQ
AIMDDERLD
>1IN4A

MSEFLTPERTVYDSGVQFLRPKSLDEFIGQENVKKKLSLALEAAKMRGEVLDHVLLAGPPGLGKTT
LAHIIASELQTNIHVTSGPVLVKQGDMAAILTSLERGDVLFIDEIHRLNKAVEELLYSAIEDFQID
IMIGKGPSAKSIRIDIQPFTLVGATTRSGLLSSPLRSRFGIILELDFYTVKELKEIIKRAASLMDV
EIEDAAAEMIAKRSRGTPRIAIRLTKRVRDMLTVVKADRINTDIVLKTMEVLNIDDEGLDEFDRKI
LKTIIEIYRGGPVGLNALAASLGVEADTLSEVYEPYLLQAGFLARTPRGRIVTEKAYKHLKYEVPE
NRLF
>2OBPA
GMSDPGNEQNGDGIDPAIVEVLLVLREAGIENGATPWSLPKIAKRAQLPMSVLRRVLTQLQAAGLA
DVSVEADGRGHASLTQEGAALAAQLFPDPF
>2JGNA
MHHHHHHENLYFQGSTSENITQKVVWVEESDKRSFLLDLLNATGKDSLTLVFVETKKGADSLEDFL
YHEGYACTSIHGDRSQRDREEALHQFRSGKSPILVATAVAARGLDISNVKHVINFDLPSDIEEYVH
RIGRTGRVGNLGLATSFFNERNINITKDLLDLLVEAKQEVPSWLENMAYEHHY
>3HJHA
MPEQYRYTLPVKAGEQRLLGELTGAACATLVAEIAERHAGPVVLIAPDMQNALRLHDEISQFTDQM
VMNLADWETLPYDSFSPHQDIISSRLSTLYQLPTMQRGVLIVPVNTLMQRVCPHSFLHGHALVMKK
GQRLSRDALRTQLDSAGYRHVDQVMEHGEYATRGALLDLFPMGSELPYRLDFFDDEIDSLRVFDVD
SQRTLEEVEAINLLPAHEFPTDKAAIELFRSQWRDTFEVKRDPEHIYQQVSKGTLPAGIEYWQPLF
FSEPLPPLFSYFPANTLLVNTGDLETSAERFQADTLARFENRGVDPMRPLLPPQSLWLRVDELFSE
LKNWPRVQLKTEHLPTKAANANLGFQKLPDLAVQAQQKAPLDALRKFLETFDGPVVFSVESEGRRE
ALGELLARIKIAPQRIMRLDEASDRGRYLMIGAAEHGFVDTVRNLALICESDLLGERVARRRQDSR
RTINPDTLKLAAALEHHHHHH
>2E1NA
GPLGSSRVSVLSMDFEDIYRFFQDPPPHYLSKELAVCYVLAVLRHEDSYGTELIQHLETHWPNYRL
SDTVLYTALKFLEDEQIISGYWKKVEGRGRPRRMYQLAQANDDRSRDLAQLWERYLSSSAATDRQL
IPVEAR
>2OKFA
GMSARDVFHEVVKTALKKDGWQITDDPLTISVGGVNLSIDLAAQKLIAAERQGQKIAVEVKSFLKQ
SSAISEFHTALGQFINYRGALRKVEPDRVLYLAVPLTTYKTFFQLDFPKEIIIENQVKMLVYDVEQ
EVIFQWIN
>2WCWA
GTMGKSKGTRFERDLLVELWKAGFAAIRVAGAGVSPFPCPDIVAGNGRTYLAIEVKMRKELPLYLS
ADEVEQLVTFARGFGAEAYVALKLPRAAWRFFPVQMLERTEKNFKIDESVYPLGLEIAEVAGKFFQ
ERFGEKV
>1WCV1
MLRAKVRRIALANQKGGVGKTTTAINLAAYLARLGKRVLLVDLDPQGNATSGLGVRAERGVYHLLQ
GEPLEGLVHPVDGFHLLPATPDLVGATVELAGAPTALREALRDEGYDLVLLDAPPSLSPLTLNALA
AAEGVVVPVQAEYYALEGVAGLLATLEEVRAGLNPRLRLLGILVTMYDGRTLLAQQVEAQLRAHFG
EKVFWTVIPRNVRLAEAPSFGKTIAQHAPTSPGAHAYRRLAEEVMARVQEAGSHHHHHH
>3HO7A
IIEEEQQSLTGRLNIAVLPTIAPYLLPRVFPIWKKELAGLEIHVSEMQTSRCLASLLSGEIDMAII
ASKAETEGLEDDLLYYEEFLGYVSRCEPLFEQDVIRTTEVNPHRLWLLDEGHCFRDQLVRFCQMKG
LHERQTAYSGGSMEAFMRLVESGQGITFIPQLTVEQLSPSQKELVRPFGMPRPVREVRLAVRQDYS
RRKLREQLIGLLRSAVPSDMHKLQTGQHLAHHHH

>3H1DA
MGSSHHHHHHSSGLEVLFQGPHMDFDVKRKYFRQELERLDEGLRKEDMAVHVRRDHVFEDSYRELH
RKSPEEMKNRLYIVFEGEEGQDAGGLLREWYMIISREMFNPMYALFRTSPGDRVTYTINPSSHANP
NHLSYFKFVGRIVAKAVYDNRLLECYFTRSFYKHILGKSVRYTDMESEDYHFYQGLVYLLENDVST
LGYDLTFSTEVQEFGVAEVRDLKPNGANILVTEENKKEYVHLVCQMRMTGAIRKQLAAFLEGFYEI
IPKRLISIFTEQELELLISGLPTIDIDDLKSNTEYHKYQSNSIQIQWFWRALRSFDQADRAKFLQF
VTGTSKVPLQGFAALEGMNGIQKFQIHRDDRSTDRLPSAHTCFNQLDLPAYESFEKLRHMLLLAIQ
EASEGFGLA
>3II2A
GSEMSVVEYEVVSKNLTSKMSHELLFSVKKRWFVKPFRHDRQLGKLHYKLLPGNYIKFGLYVLKNQ
DYARFEIAWVHVDKDGKIEERTVYSIETYWHIFIDIENDLNCPYVLAKFIEMRPEFHKTAWVEESN
YSIAEDDIQMVESIKRYLERKIASD
>3KORA
MGSSHHHHHHSSGLVPRGSMQIEKLRGAALDELFDAILTLENREECYQFFDDLCTVNEIQSLSQRL
QVAKMIKQGYTYATIEQESGASTATISRVKRSLQWGNDAYTMILDRMNIETNE
>3KOSA
MLDRFATKQTQEKLKIGVVGTFAIGCLFPLLSDFKRSYPHIDLHISTHNNRVDPAAEGLDYTIRYG
GGAWHDTDAQYLCSALMSPLCSPTLASQIQTPADILKFPLLRSYRRDEWALWMQTVGEAPPSPTHN
VMVFDSSVTMLEAAQAGMGVAIAPVRMFTHLLSSERIVQPFLTQIDLGSYWITRLQSRPETPAMRE
FSRWLTGVLHKTSGSHHHHHH
>3KOJA
MGHHHHHHSHMNSCILQATVVEAPQLRYAQDNQTPVAEMVVQFPGLSSKDAPARLKVVGWGAVAQE
LQDRCRLNDEVVLEGRLRINSLLKPDGNREKQTELTVTRVHH
>2PKHA
SNAARGHRHTCKVMVLKEEAAGSERALALDMREGQRVFHSLIVHFENDIPVQIEDRFVNAQVAPDY
LKQDFTLQTPYAYLSQVAPLTEGEHVVEAILAEADECKLLQIDAGEPCLLIRRRTWSGRQPVTAAR
LIHPGSRHRLEGRFTK
>2RH3A
IQVFLSARPPAPEVSKIYDNLILQYSPSKSLQMILRRALGDFENMLADGSFRAAPKSYPIPHTAFE
KSIIVQTSRMFPVSLIEAARNHFDPLGLETARAFGHKLATAALACFFAREKATNS
>3F6VA
MGSSHHHHHHSSGRENLYFQGHMNSPTSRPLRRDPLHNALVTTNVLVVLDQLEVAAEPTRRRLVQL
LTSGEQTVNNLAAHFPASRSAISQHLRVLTEAGLVTPRKDGRFRYYRLDPQGLAQLRALFDSFWID
ELDRLVADATEEAASKGDS
>1ZELA
GAMVVSPAGADRRIPTWASRVVSGLARDRPVVVTKEDLTQRLTEAGCGRDPDSAIRELRRIGWLVQ
LPVKGTWAFIPPGEAAISDPYLPLRSWLARDQNAGFMLAGASAAWHLGYLDRQPDGRIPIWLPPAK
RLPDGLASYVSVVRIPWNAADTALLAPRPALLVRRRLDLVAWATGLPALGPEALLVQIATRPASFG
PWADLVPHLDDLVADCSDERLERLLSGRPTSAWQRASYLLDSGGEPARGQALLAKRHTEVMPVTRF
TTAHSRDRGESVWAPEYQLVDELVVPLLRVIGKA
>1DC1A
MNTQKPFENHLKSVDDLKTTYEEYRAGFIAFALEKNKRSTPYIERARALKVAASVAKTPKDLLYLE
DIQDALLYASGISDKAKKFLTEDDKKESINNLIENFLEPAGEEFIDELIFRYLLFQGDSLGGTMRN
IAGALAQQKLTRAIISALDIANIPYKWLDSRDKKYTNWMDKPEDDYELETFAKGISWTINGKHRTL

MYNITVSLVKKNVDICLFNCEPEIYTPQKVHQQPEKYLLLGELKGGIDPAGADEHWKTANTALTRI
RNKFSEKGLSPKTIFIGAAIEHSMAEEIWDQLQSGSLTNSANLTKTEQVGSLCRWIINI
>3CJDA
GMAGKVEARKAALREKLIDLAEAQIEAEGLASLRARELARQADCAVGAIYTHFQDLNALTLEVNGR
TFARLGAAVGAVVADGQDDHPNERLIAMSHAYLAFAREHPKLWRALFDVEMRSDGPVPQWYGHAMA
QLFSYITTPLAKIFPESDDAELDLMTRTLFSSVHGIVLLGLENRISGVPGEQLKTMIRLLLEQVGR
>3CJWA
GSNSHSYLSGYISLLLRAEPYPTSRFGSQCMQPNNIMGIENICELAARMLFSAVEWARNIPFFPDL
QITDQVALLRLTWSELFVLNAAQCSMPLHVAPLLAAAGLHASPMSADRVVAFMDHIRIFQEQVEKL
KALHVDSAEYSCLKAIVLFTSDACGLSDVAHVESLQEKSQCALEEYVRSQYPNQPTRFGKLLLRLP
SLRTVSSSVIEQLFFVRLVGKTPIETLIRDMLLSGSSFNWPYMAIQ
>2CJJA
MASTRGSGRPWSAKENKAFERALAVYDKDTPDRWANVARAVEGRTPEEVKKHYEILVEDIKYIESG
KVPFPNYRTTGGNMKTDEKRFRNLKIR
>1XMKA
GSHMASLDMAEIKEKICDYLFNVSDSSALNLAKNIGLTKARDINAVLIDMERQGDVYRQGTTPPIW
HLTDKKRERMQIK
>2QSXA
SNAIQHATASLIQTNTDQELLVVDVTPSFASLWLVPNINDFHQRHPNIRVKILTGDGAVKNIHGES
DLHVRCLPLSTHYEYSQLLCEETLLLIGNTNLPKLSDNQAISHYPFIPQTTRPQLWEQFKQENDLE
CPITYHSVGFEHFYLACEAVRMEKGLALLPDFMAQFSILRGDIQHIGNLKLHSGYGYYVVIPNFRL
TSRKVALFHDWLKDKLTHHT
>3GVAA
MRMDEFYTKVYDAVCEIPYGKVSTYGEIARYVGMPSYARQVGQAMKHLHPETHVPWHRVINSRGTI
SKRDISAGEQRQKDRLEEEGVEIYQTSLGEYKLNLPEYMWKPGSHHHHHH
>3BBBA
ANLERTFIAIKPDGVQRGLVGEIIKRFEQKGFRLVAMKFLRASEEHLKQHYIDLKDRPFFPGLVKY
MNSGPVVAMVWEGLNVVKTGRVMLGETNPADSKPGTIRGDFCIQVGRNIIHGSDSVKSAEKEISLW
FKPEELVDYKSCAHDWVYE
>3JTZA
MSLTDAKIRTLKPSDKPFKVSDSHGLYLLVKPGGSRHWYLKYRISGKESRIALGAYPAISLSDARQ
QREGIRKMLALNINLEHHHHHH
>3B44A
ERAGPVTWVMMIACVVVFIAMQILGDQEVMLWLAWPFDPTLKFEFARYFTHALMHFSLMHILFNLL
WWWYLGGAVEKRLGSGKLIVITLISALLSGYVQQKFSGPWFGGLSGVVYALMGYVWLRGERDPQSG
IYLQRGLIIFALIWIVAGWFDLFGMSMANGAHIAGLAVGLAMAFVDSL
>3FHWA
MNTLELSARVLECGAMRHTPAGLPALELLLVHESEVVEAGHPRRVELTISAVALGDLALLLADTPL
GTEMQVQGFLAPARKDSVKVKLHLQQARRIAGSMGRDPLVGLEHHHHHH
>3LY7A
GSGMKETAAAKFERQHMDSPDLGTDDDDKAMAHHHHHHSSGHIEGRHSKSRILLNPRDIDINMVNK
SCNSWSSPYQLSYAIGVGDLVATSLNTFSTFMVHDKINYNIDEPSSSGKTLSIAFVNQRQYRAQQC
FMSIKLVDNADGSTMLDKRYVITNGNQLAIQNDLLESLSKALNQPWPQRMQETLQKILPHRGALLT
NFYQAHDYLLHGDDKSLNRASELLGEIVQSSPEFTYARAEKALVDIVRHSQHPLDEKQLAALNTEI

DNIVTLPELNNLSIIYQIKAVSALVKGKTDESYQAINTGIDLEMSWLNYVLLGKVYEMKGMNREAA
DAYLTAFNLRPGANTLYWIENGIFQTSVPYVVPYLDKFLASE
>2F2EA
MVKRTSHKQASCPVARPLDVIGDGWSMLIVRDAFEGLTRFGEFQKSLGLAKNILAARLRNLVEHGV
MVAVPAESGSHQEYRLTDKGRALFPLLVAIRQWGEDYFFAPDESHVRLVERDSGQPVPRLQVRAGD
GSPLAAEDTRVSRD
>2WLUA
MTNTLVENIYASVTHNISKKEASKNEKTKAVLNQAVADLSVAASIVHQVHWYMRGPGFLYLHPKMD
ELLDSLNANLDEVSERLITIGGAPYSTLAEFSKHSKLDEAKGTYDKTVAQHLARLVEVYLYLSSLY
QVGLDITDEEGDAGTNDLFTAAKTEAEKTIWMLQAERGQGPAL
>3E97A
GVGRLDDLKRSPLFQNVPEDAMREALKVVTERNFQPDELVVEQDAEGEALHLVTTGVVRVSRVSLG
GRERVLGDIYAPGVVGETAVLAHQERSASVRALTPVRTLMLHREHFELILRRHPRVLWNLAEMLAR
RVTFLNDELIAFGQNTEAALTHVFANLYRQRLAAGVPQPEVLPLGTQDIMARTSSSRETVSRVLKR
LEAHNILEVSPRSVTLLDLAALEALSFEGAETD
>1O7IA
MEEKVGNLKPNMESVNVTVRVLEASEARQIQTKNGVRTISEAIVGDETGRVKLTLWGKHAGSIKEG
QVVKIENAWTTAFKGQVQLNAGSKTKIAEASEDGFPESSQIPENTPTAPQQMR
>2AIFA
GSSQNEASEDTGFNPKAFPLASPDLNNKIINLVQQACNYKQLRKGANEATKALNRGIAEIVLLAAD
AEPLEILLHLPLVCEDKNTPYVFVRSKVALGRACGVSRPVIAAAITSKDGSSLSSQITELKDQIEQ
ILV
>3HWUA
GQELKGKYMKTPTGYLMVLRHGDNVLQNLEQLARDEHIPSASFVGIGFMSEATFGFYDFGRKQFDP
KTYRNVEMANMTGSIAWKEGKPSIHAHGTVTDGTFQGAGGHLLGLTVGTGSCEITVTVYPQRLDRF
VDPEIQANVLGLPQQ
>3K2ZA
MDLTERQRKVLLFIEEFIEKNGYPPSVREIARRFRITPRGALLHLIALEKKGYIERKNGKPRALRI
SKSIRNKIPLIGEIRAGEKREAIEYLEDYIEIPESFLSSGYDHFLLKVKGESMIEEHICDGDLVLV
RRQDWAQNGDIVAAMVDGEVTLAKFYQRGDTVELRPANREMSSMFFRAEKVKILGKVVGVFRKL
>3K2AA
GSGIFPKVATNIMRAWLFQHLTHPYPSEEQKKQLAQDTGLTILQVNNWFINARRRIVQPMIDQSNR
A
>1C1KA
MIKLRMPAGGERYIDGKSVYKLYLMIKQHMNGKYDVIKYNWCMRVSDAAYQKRRDKYFFQKLSEKY
KLKELALIFISNLVANQDAWIGDISDADALVFYREYIGRLKQIKFKFEEDIRNIYYFSKKVEVSAF
KEIFEYNPKVQSSYIFKLLQSNIISFETFILLDSFLNIIDKHDEQTDNLVWNNYSIKLKAYRKILN
IDSQKAKNVFIETVKSCKY
>3C1DA
GPAYARLLDRAVRILAVRDHSEQELRRKLAAPIMGKNGPEEIDATAEDYERVIAWCHEHGYLDDSR
FVARFIASRSRKGYGPARIRQELNQKGISREATEKAMREADIDWAALARDQATRKYGEPLPTVFSE
KVKIQRFLLYRGYLMEDIQDIWRNFAD
>3CZ6A
GAIRSDFSNEDIYDNIDPDTISFPPKIATTDLFLPLFFHFGSTRQFMDKLHEVISGDYEPSQAEKL

VQDLCDETGIRKNFSTSILTCLSGDLMVFPRYFLNMFKDNVNPPPNVPGIWTHDDDESLKSNDQEQ
IRKLVKKHGTGRMEMRKRFFEKDLLVPRGSHHHHHH
>3BS1A
MDNSVETIELKRGSNSVYVQYDDIMFFESSTKSHRLIAHLDNRQIEFYGNLKELSQLDDRFFRCHN
SFVVNRHNIESIDSKERIVYFKNKEHCYASVRNVKKI
>3BS3A
SNAMSNNQQMMLNRIKVVLAEKQRTNRWLAEQMGKSENTISRWCSNKSQPSLDMLVKVAELLNVDP
RQLINGKIKI
>1YCZA
MKEKIRKKILLAPEEPGVYIFKNKGVPIYIGKAKRLSNRLRSYLNPQTEKVFRIGEEADELETIVV
MNEREAFILEANLIKKYRPKYNVRLKDTDF
>1G8EA
MHTSELLKHIYDINLSYLLLAQRLIVQDKASAMFRLGINEEMATTLAALTLPQMVKLAETNQLVCH
FRFDSHQTITQLTQDSRVDDLQQIHTGIMLSTRLLNDVNQPEEALRKKRA
>3G85A
MSLRSKNSQSKPTIALYWSSDISVNIISRFLRGLQSKLAKQNYNYNVVICPYKTDCLHLEKGISKE
NSFDAAIIANISNYDLEYLNKASLTLPIILFNRLSNKYSSVNVDNYKMGEKASLLFAKKRYKSAAA
ILTESLNDAMDNRNKGFIETCHKNGIKISENHIIAAENSIHGGVDAAKKLMKLKNTPKALFCNSDS
IALGVISVLNKRQISIPDDIEIVAIGMNDREYTEFSTPPVTIVDIPIEEMAGTCISLVEKLINRDI
ENPTSILFDGPLILRNSEGHHHHHH
>3D2WA
MRGSHHHHHHGSKVFVGRCTEDMTAEELQQFFCQYGEVVDVFIPKPFRAFAFVTFADDKVAQSLCG
EDLIIKGISVHISNAEPKHNKLN
>3QAOA
SNAMQIKELAELTGVSVRTLHHYDKIGLLVPQKDDWNGYRIYSEKDVDKLQQILFFKELDFPLKKI
QQILDDPLFDKNVALDMQRHLLIEKKQRIETMLATLDLTIKNEKGEITMTNKEKFTGFDFSSNPYE
EEARKLWGDKVVEKANEKVNNMSEKEQLTLKESFDAEFRHLASVRKLTPESEEAQLEIDHFFHYLN
DTHGNIYSLEAFASLGEMYVNDERFTKNIDQFGDGLSQFLQEAMTIYAKNK
>3ABDA
MGSSHHHHHHSQDPNSMTTLTRQDLNFGQVVADVLCEFLEVAVHLILYVREVYPVGIFQKRKKYNV
PVQMSCHPELNQYIQDTLHCVKPLLEKNDVEKVVVVILDKEHRPVEKFVFEITQPPLLSISSDSLL
SHVEQLLAAFILKISVCDAVLDHNPPGCTFTVLVHTREAATRNMEKIQVIKDFPWILADEQDVHMH
DPRLIPLKTMTSDILKMQLYVEERAHKGS
>2IKSA
EHEHNYHPNAVAAGLRAGRTRSIGLVIPDLENTSYTRIANYLERQARQRGYQLLIACSEDQPDNEM
RCIEHLLQRQVDAIIVSTSLPPEHPFYQRWANDPFPIVALDRALDREHFTSVVGADQDDAEMLAEE
LRKFPAETVLYLGALPELSVSFLREQGFRTAWKDDPREVHFLYANSYEREAAAQLFEKWLETHPMP
QALFTTSFALLQGVMDVTLRRDGKLPSDLAIATFGDNELLDFLQCPVLAVAQRHRDVAERVLEIVL
ASLDEPRKPKPGLTRIKRNLYRRGVLSRS
>1KW4A
METKRVNGTDRPPISSWSVDDVSNFIRELPGCQDYVDDFIQQEIDGQALLRLKEKHLVNAMGMKLG
PALKIVAKVESIKEVRDHHHHHH
>1N83A
GSSHHHHHHLEVLFQGPAELEHLAQNISKSHLETCQYLREELQQITWQTFLQEEIENYQNKQREVM

WQLCAIKITEAIQYVVEFAKRIDGFMELCQNDQIVLLKAGSLEVVFIRMCRAFDSQNNTVYFDGKY
ASPDVFKSLGCEDFISFVFEFGKSLCSMHLTEDEIALFSAFVLMSADRSWLQEKVKIEKLQQKIQL
ALQHVLQKNHREDGILTKLICKVSTLRALCGRHTEKLMAFKAIYPDIVRLHFPPLYKELFTSEFEP
AMQIDG
>3L15A
AWQARGLGTARLQLVEFSAFVEPPDAVDSYQRHLFVHISQHCPSPGAPPLESVDVRQIYDKFPEKK
GGLRELYDRGPPHAFFLVKFWADLNWGPSGEEAGAGGSISSGGFYGVSSQYESLEHMTLTCSSKVC
SFGKQVVEKVETERAQLEDGRFVYRLLRSPMCEYLVNFLHKLRQLPERYMMNSVLENFTILQVVTN
RDTQELLLCTAYVFEVSTSERGAQHHIYRLVRD
>3NO7A
GPEASARSEVKMTVTVGEERRARLRTAYTLTHLQEGHRTFSGFIAAALDAEVQRLEQRYNEGRRFE
NAERGVTRGRPLGS
>3NO0A
GSITVAVLQDGSIIPVEELPLEKAPVVNILRVPFTEGLFLVSNRGRVYWIAGSQALQGSKVSLKSR
EEKIVGAFIREKFGNRLLLATKKGYVKKIPLAEFEYKAQGMPIIKLTEGDEVVSIASSVDETHILL
FTKKGRVARFSVREVPPSTPGARGVQGIKLEKNDETSGLRIWNGEPYLLVITAKGRVKKISHEEIP
KTNRGVKGTEVSGTKDTLVDLIPIKEEVELLITTKNGKAFYDKINQKDIPLSTKKSIPRTRWKLED
DEIIKVVIKKSE
>3KDEC
MKYCKFCCKAVTGVKLIHVPKCAIKRKLWEQSLGCSLGENSQICDTHFNDSQWKAAPAKGQTFKRR
RLNADAVPSKV
>3CTPA
SLANIREIAKRAGISIATVSRHLNNTGYVSEDAREKIQKVVDELNYTPNALARAMFTKNSKTIGLM
VPNISNPFFNQMASVIEEYAKNKGYTLFLCNTDDDKEKEKTYLEVLQSHRVAGIIASRSQCEDEYA
NIDIPVVAFENHILDNIITISSDNYNGGRMAFDHLYEKGCRKILHIKGPEVFEATELRYKGFLDGA
RAKDLEIDFIEFQHDFQVKMLEEDINSMKDIVNYDGIFVFNDIAAATVMRALKKRGVSIPQEVQII
GFDNSFIGELLYPSLTTINQPIEALAYTIIELLIKIINGEGVLIEDYIMEVKLIERETTISLKDEG
>3CTBA
MKKGHHHHHHGSERTGTQPLGVQGLTEEQRMMIRELMDAQMKTFDTTFSHFKNFRLPGVLSSGCEL
PESLQAPSREEAAKWSQVRKDLCSLKVSLQLRGEDGSVWNYKPPADSGGKEIFSLLPHMADMSTYM
FKGIISFAKVISYFRDLPIEDQISLLKGAAFELCQLRFNTVFNAETGTWECGRLSYCLEDTAGGFQ
QLLLEPMLKFHYMLKKLQLHEEEYVLMQAISLFSPDRPGVLQHRVVDQLQEQFAITLKSYIECNRP
QPAHRFLFLKIMAMLTELRSINAQHTQRLLRIQDIHPFATPLMQELFGITGSGGSGGSSHSSLTER
HKILHRLLQEGSPS
>1UDVA
MTEKLNEIVVRKTKNVEDHVLDVIVLFNQGIDEVILKGTGREISKAVDVYNSLKDRLGDGVQLVNV
QTGSEVRDRRRISYILLRLKRVY
>3EULA
MHHHHHHMSNPQPEKVRVVVGDDHPLFREGVVRALSLSGSVNVVGEADDGAAALELIKAHLPDVAL
LDYRMPGMDGAQVAAAVRSYELPTRVLLISAHDEPAIVYQALQQGAAGFLLKDSTRTEIVKAVLDC
AKGRDVVAPSLVGGLAGEIR
>3EUSA
QAMTKTLRTPEHVYLCQRLRQARLDAGLTQADLAERLDKPQSFVAKVETRERRLDVIEFAKWMAAC
EGLDVVSEIVATIAEGRAQA

>2V79A

MKKQQFIDMQEQGTSTIPNLLLTHYKQLGLNETELILLLKIKMHLEKGSYFPTPNQLQEGMSISVE
ECTNRLRMFIQKGFLFIEECEDQNGIKFEKYSLQPLWGKLYEYIQLAQNQTQERKAEGEQKLEHHH
HHH

>3GEZA

MLESNENRIQIMSTIAKIYRAMSRELNRRLGELNLSYLDFLVLRATSDGPKTMAYLANRYFVTQSA
ITASVDKLEEMGLVVRVRDREDRRKILIEITEKGLETFNKGIEIYKKLANEVTGDLSEDEVILVLD
KISKILKRIEEISQ

>3B84A

SMSFVQHSVRVLQELNKQREKGQYCDATLDVGGLVFKAHWSVLACCSHFFQSLYGDGSGGSVVLPA
GFAEIFGLLLDFFYTGHLALTSGNRDQVLLAARELRVPEAVELCQSFKPKTSV

>1B8ZA

MNKKELIDRVAKKAGAKKKDVKLILDTILETITEALAKGEKVQIVGFGSFEVRKAAARKGVNPQTR
KPITIPERKVPKFKPGKALKEKVK

>1Q8RA

MNTYSITLPWPPSNNRYYRHNRGRTHVSAEGQAYRDNVARIIKNAMLDIGLAMPVKIRIECHMPDR
RRRDLDNLQKAAFDALTKAGFWLDDAQVVDYRVVKMPVTKGGRLELTITEMGNE

>3IU5A

SMTVDPIAVCHELYNTIRDYKDEQGRLLCELFIRAPKRRNQPDYYEVVSQPIDLMKIQQKLKMEEY
DDVNLLTADFQLLFNNAKSYYKPDSPEYKAACKLWDLYLRTRNEFVQKGE

>3IU6A

SMNVTLLIQELIHNLFVSVMSHQDDEGRCYSDSLAEIPAVDPNFPNKPPLTFDIIRKNVENNRYRR
LDLFQEHMFEVLERARRMNRTDSEIYEDAVELQQFFIKIRDELCKNGEILLSPALSYTTKHLHNDV
EKERKEKLPKEIEED

>2OXLA

NLLEEESAVLGQAVTNLMLSGDNVNNKNIILSLIHSLETTSDILKADVIRKTLEIVLRYTADDM

>3LHQA

SNAMARKTKQQALETRQHILDVALRLFSQQGVSATSLAEIANAAGVTRGAIYWHFKNKSDLFSEIW
ELSESNIGELEIEYQAKFPDDPLSVLREILVHILEATVTEERRRLLMEIIFHKCEFVGEMVVVQQA
QRSLCLESYDRIEQTLKHCINAKMLPENLLTRRAAILMRSFISGLMENWLFAPQSFDLKKEARAYV
TILLEMYQLCPTLRASTVNGSP

>3LQHA

SGNFCPLCDKCYDDDDYESKMMQCGKCDRWVHSKCENLSDEMYEILSNLPESVAYTCVNCTERHPA
EWRLALEKELQISLKQVLTALLNSRTTSHLLRYRQQQPLDLEGVKRKMDQGNYTSVLEFSDDIVKI
IQAAINSDGGQPEIKKANSMVKSFFIRQMERVFPWFSVKKSRFWEPNKVSS

>2ICPA

MKMANHPRPGDIIQESLDELNVSLREFARAMEIAPSTASRLLTGKAALTPEMAIKLSVVIGSSPQM
WLNLQNAWSLAEAEKTVDVSRLRRLVTQ

>3M8EA

MGSSHHHHHHSSGLVPRGSHMNRDHFYTLNIAEIAERIGNDDCAYQVLMAFINENGEAQMLNKTAV
AEMIQLSKPTVFATVNSFYCAGYIDETRVGRSKIYTLSDLGVEIVECFKQKAMEMRNL

>3KCZA

MHHHHHHSSGVDLGTENLYFQSMDLRVQELIKLICNVQAMEEMMMEMKYNTKKAPLGKLTVAQIKA
GYQSLKKIEDCIRAGQHGRALMEACNEFYTRIPHDFGLRTPPLIRTQKELSEKIQLLEALGDIEIA

IKLVKTELQSPEHPLDQHYRNLHCALRPLDHESYEFKVISQYLQSTHAPTHSDYTMTLLDLFEVEK
DGEKEAFREDLHNRMLLWHGSRMSNWVGILSHGLRIAHPEAPITGYMFGKGIYFADMSSKSANYCF
ASRLKNTGLLLLSEVALGQCNELLEANPKAEGLLQGKHSTKGLGKMAPSSAHFVTLNGSTVPLGPA
SDTGILNPDGYTLNYNEYIVYNPNQVRMRYLLKVQFNF

>2ZCWA

MTQVRETVSFKAGDVILYPGVPGPRDRAYRVLEGLVRLEAVDEEGNALTLRLVRPGGFFGEEALFG
QERIYFAEAATDVRLEPLPENPDPELLKDLAQHLSQGLAEAYRRIERLATQRLKNRMAAALLELSE
TPLAHEEEGKVVLKATHDELAAAVGSVRETVTKVIGELAREGYIRSGYGKIQLLDLKGLKELAESR
GQGR

>2ZCMA

MHHHHHHMKDKIIDNAITLFSEKGYDGTTLDDISKSVNIKKASLYYHYDNKEEIYRKSVENCFNYF
IDFMMRNHDDNYSIDGLYQFLFKFIFDVDERYIKLYVQLSSAPEALNSEIKHHLQEINTTLHDELI
KYYDPTHIALDKEDFINMILMFLETWYFRASFSQKFGIIEDSKNRFKDQVYSLLNVFLKK

>1SD4A

MTNKQVEISMAEWDVMNIIWDKKSVSANEIVVEIQKYKEVSDKTIRTLITRLYKKEIIKRYKSENI
YFYSSNIKEDDIKMKTAKTFLNKLYGGDMKSLVLNFAKNEELNNKEIEELRDILNDISKK

>2C62A

AMFQIGKMRYVSVRDFKGKVLIDIREYWMDPEGEMKPGRKGISLNPEQWSQLKEQISDIDDAVRKL

>2ETWA

GPLGSMNEMENTDPVLQDDLVSKYERELSTEQEEDTPVILTQLNEDGTTSNYFDKRKLKIAPRSTL
QFKVGPPFELVRDYCPVVESHTGRTLDLRIIPRIDRGFDHIDEEWVGYKRNYFTLVSTFETANCDL
DTFLKSSFDLLVEDSSVEGRLRVQYFAIKIKAKNDDDDTEINLVQHTAKRDKGPQFCPSVCPLVPS
PLPKHQTIREASNVRNITKMKKYDSTFYLHRDHVNYEEYGVDSLLFSYPEDSIQKVARYERVQFAS
SISVKKPSQQNKHFSLHVILGAVVDPDTFHGENPGIPYDELALKNGSKGMFVYLQEMKTPPLIIRG
RSPSNYASSQRITVR

>2FD5A

MSDKKTQTRARILGAATQALLERGAVEPSVGEVMGAAGLTVGGFYAHFQSKDALMLEAFEQLLGKR
RELLGELDPGLSGKERRALAAAFYLSRKHRDAQVDAGCPLPATLAEVARLPEGFREVLSRHVEIMV
TSLAESPEETDVALADLVLMIGGLALARALGPGELSDRVLRAAKQAVN

>3FDQA

MPKSEIRKLLQEIKKQVDNPGNSSTTEIKKMASEAGIDEQTAEEIYHLLTEFYQAVEEHGGIEKYM
HSNISWLKIELELLSACYQIAILEDMKVLDISEMLSLNDLRIFPKTPSQLQNTYYKLKKELIQVED
IPKNKPGRKRKTQKNTKKEKTNIFGKVVPALEHHHHHH

>2X35A

GGSEDEDSPLDALDLVWAKCRGYPSYPALIIDPKMPREGMFHHGVPIPVPPLEVLKLGEQMTQEAR
EHLYLVLFFDNKRTWQWLPRTKLVPLGVNQDLDKEKMLEGRKSNIRKSVQIAYHRALQHRSKVQGE

>1G5HA

WLSGYAGPADGTQQPDAPEHAVAREALVDLCRRRHFLSGTPQQLSTAALLSGCHARFGPLGVELRK
NLASQWWSSMVVFREQVFAVDSLHQEPGSSQPRDSAFRLVSPESIREILQDREPSKEQLVAFLENL
LKTSGKLRATLLHGALEHYVNCLDLVNRKLPFGLAQIGVCFHPVSNSNQTPSSVTRVGEKTEASLV
WFTPTRTSSQWLDFWLRHRLLWWRKFAMSPSNFSSADCQDELGRKGSKLYYSFPWGKEPIETLWNL
GDQELLHTYPGNVSTIQGRDGRKNVVPCVLSVSGDVDLGTLAYLYDSFQLAENSFARKKSLQRKVL
KLHPCLAPIKVALDVGKGPTVELRQVCQGLLNELLENGISVWPGYSETVHSSLEQLHSKYDEMSVL
FSVLVTETTLENGLIQLRSRDTTMKEMMHISKLRDFLVKYLASASNVAAALDHHHHHH

>3B7GA

SMRTAQDLSSPRTRTGDVLLAEPADFESLLLSRPVLEGLRAAGFERPSPVQLKAIPLGRCGLDLIV
QAKSGTGKTCVFSTIALDSLVLENLSTQILILAPTREIAVQIHSVITAIGIKMEGLECHVFIGGTP
LSQDKTRLKKCHIAVGSPGRIKQLIELDYLNPGSIRLFILDEADKLLEEGSFQEQINWIYSSLPAS
KQMLAVSATYPEFLANALTKYMRDPTFVRLNS

>2ZKZA

MTVFVDHKIEYMSLEDDAELLKTMAHPMRLKIVNELYKHKALNVTQIIQILKLPQSTVSQHLCKMR
GKVLKRNRQGLEIYYSINNPKVEGIIKLLNPIQ

>3M66A

DYVDHSETLQKLVLLGVDLSKIEKHPEAANLLLRLDFEKDIKQMLLFLKDVGIEDNQLGAFLTKNH
AIFSEDLENLKTRVAYLHSKNFSKADVAQMVRKAPFLLNFSVERLDNRLGFFQKELELSVKKTRDL
VVRLPRLLTGSLEPVKENMKVYRLELGFKHNEIQHMITRIPKMLTANKMKLTETFDFVHNVMSIPH
HIIVKFPQVFNTRLFKVKERHLFLTYLGRAQYDPAKPNYISLDKLVSIPDEIFCEEIAKASVQDFE
KFLKTL

>3JVEA

GPLGSEAQSEKEEAPKPLHKVVVCVSKKLSKKQSELNGIAASLGADYRWSFDETVTHFIYQGRPND
TNREYKSVKERGVHIVSEHWLLDCAQECKHLPESLYPHTYNPK

>3IFJA

CLAEGTRIFDPVTGTTHRIEDVVDGRKPIHVVAAAKDGTLHARPVVSWFDQGTRDVIGLRIAGGAI
VWATPDHKVLTEYGWRAAGELRKGDRVAVRDVETGELRYSVIREVLPTRRARTYDLEVEELHTLVA
EGVVVHD

>3K9JA

HLKQIGKVKKLDKWVPHELTENQKNRRFEVSSSLILRNHNEPFLDRIVTCDEKWILYDNRRRSAQW
LDQEEAPKHFPKPILHPKKVMVTIWWSAAGLIHYSFLNPGETITSEKYAQEIDEMNQKLQRLQLAL
VRRKRPILLHDNARPHVAQPTLQKLNELGYEVLPHPPYSPDLLPTNYHVFKHLNNFLQGKRFHNQQ
DAENAFQEFVESQSTDFYWTGINQLISRWQKCVDCNGSYFD

>2W9RA

MGKTNDWLDFDQLAEEKVRDALKPPSMYKVILVNDDYTPMEFVIDVLQKFFSYDVERATQLMLAVH
YQGKAICGVFTAEVAETKVAMVNKYARENEHPLLCTLEKAGA

>2DB7A

GSSGSSGGYFDAHALAMDYRSLGFRECLAEVARYLSIIEGLDASDPLRVRLVSHLNNYASQREA

>2HKVA

GMTDWQQALDRHVGVGVRTTRDLIRLIQPEDWDKRPISGKRSVYEVAVHLAVLLEADLRIATGATA
DEMAQFYAVPVLPEQLVDRLDQSWQYYQDRLMADFSTETTYWGVTDSTTGWLLEAAVHLYHHRSQL
LDYLNLLGYDIKLDLFE

>3M7KA

MTQCPRCQRNLAADEFYAGSSKMCKGCMTWQNLSYNANKEGHANTFTKATFLAWYGLSAQRHCGYC
GISEAGFTSLHRTNPRGYHIQCLGVDRSDSFEGYSPQNARLACFICNRIKSNIFSASEMDVLGEAI
SKAWHGRGIA

>2ZOYA

MRTSKKEMILRTAIDYIGEYSLETLSYDSLAEATGLSKSGLIYHFPSRHALLLGMHELLADDWDKE
LRDITRDPEDPLERLRAVVVTLAENVSRPELLLLIDAPSHPDFLNAWRTVNHQWIPDTDDLENDAH
KRAVYLVQLAADGLFVHDYIHDDVLSKSKRQAMLETILELIPSQTLEHHHHHH

>1B9MA

GSHMQAEILLTLKLQQKLFADPRRISLLKHIALSGSISQGAKDAGISYKSAWDAINEMNQLSEHIL
VERATGGKGGGGAVLTRYGQRLIQLYDLLAQIQQKAFDVLSDDDALPLNSLLAAISRFSLQTSARN
QWFGTITARDHDDVQQHVDVLLADGKTRLKVAITAQSGARLGLDEGKEVLILLKAPWVGITQDEAV
AQNADNQLPGIISHIERGAEQCEVLMALPDGQTLCATVPVNEATSLQQGQNVTAYFNADSVIIATL
C
>3JX7A
PMHPFVKALQEHFTAHQNPEKAEPMARYMKNHFLFLGIQTPERRQLLKDIIQIHTLPDQKDFQIII
RELWDLPEREFQAAALDIMQKYKKHINETHIPFLEELIVTKSWWDSVDSIVPTFLGDIFLKHPELI
SAYIPKWIASDNIWLQRAAILFQLKYKQKMDEELLFWIIGQLHSSKEFFIQKAIGWVLREYAKTNP
DVVWEYVQNNELAPLSKREAIKHIKQNYGINNEK
>3L51A
LQFAYKDPEKNWNRNSVKGLVASLINVKDNSTATALEVVAGERLYNVVVDTEVTAKKLLEKGELKR
RYTIIPLNKISARCIAPETLRVAQNLVGPDNVHVALSLVDYKPELQKGMEFVFGTTFVCNNMDNAK
KVAFDKRIMTRTVTLGGDVFDPHGTLSGG
>2QXVA
SFKCVNSLKEDHNQPLFGVQFNWHSKEGDPLVFATVGSNRVTLYECHSQGEIRLLQSYVDADADEN
FYTCAWTYDSNTSHPLLAVAGSRGIIRIINPITMQCIKHYVGHGNAINELKFHPRDPNLLLSVSKD
HALRLWNIQTDTLVAIFGGVEGHRDEVLSADYDLLGEKIMSCGMDHSLKLWRINSKRMMNAIKESY
DYNPNKTNRPFISQKIHFPDFSTRDIHRNYVDCVRWLGDLILSKSCENAIVCWKPGKMEDDIDKIK
PSESNVTILGRFDYSQCDIWYMRFSMDFWQKMLALGNQVGKLYVWDLEVEDPHKAKCTTLTHHKCG
AAIRQTSFSRDSSILIAVCDDASIWRWDRLR
>3FAJA
MGSSHHHHHHSSGLVPRGSHMAKYEPKKGDYAGGAVKILDMFENGQLGYPEVTLKLAGEEANARRA
GDERTKEAIHAIVKMISDAMKPYRNKGSGFQSQPIPGEVIAQVTSNPEYQQAKAFLASPATQVRNI
EREEVLSKGAKKLAQAMAS
>2FAZA
GSMWIQVRTMDGRQTHTVDSLSRLTKVEELRRKIQELFHVEPGLQRLFYRGKQMEDGHTLFDYEVR
LNDTIQLLVRQS
>2OD5A
GMTGAVETESMKTVRIREKIKKFLGDRPRNTAEILEHINSTMRHGTTSQQLGNVLSKDKDIVKVGY
IKRSGILSGGYDICEWATRNWVAEHCPEWTEGQPIILNEEGDFTLGPLPE
>1DD9A
MRGSHHHHHHGSGSMHQRQTLYQLMDGLNTFYQQSLQQPVATSARQYLEKRGLSHEVIARFAIGFA
PPGWDNVLKRFGGNPENRQSLIDAGMLVTNDQGRSYDRFRERVMFPIRDKRGRVIGFGGRVLGNDT
PKYLNSPETDIFHKGRQLYGLYEAQQDNAEPNRLLVVEGYMDVVALAQYGINYAVASLGTSTTADH
IQLLFRATNNVICCYDGDRAGRDAAWRALETALPYMTDGRQLRFMFLPDGEDPDTLVRKEGKEAFE
ARMEQAMPLSAFLFNSLMPQVDLSTPDGRARLSTLALPLISQVPGETLRIYLRQELGNKLGILDDS
QLERLMPK
>1L3SA
AKMAFTLADRVTEEMLADKAALVVEVVEENYHDAPIVGIAVVNEHGRFFLRPETALADPQFVAWLG
DETKKKSMFDSKRAAVALKWKGIELCGVSFDLLLAAYLLDPAQGVDDVAAAAKMKQYEAVRPDEAV
YGKGAKRAVPDEPVLAEHLVRKAAAIWELERPFLDELRRNEQDRLLVELEQPLSSILAEMEFAGVK
VDTKRLEQMGKELAEQLGTVEQRIYELAGQEFNINSPKQLGVILFEKLQLPVLKKTKTGYSTSADV
LEKLAPYHEIVENILHYRQLGKLQSTYIEGLLKVVRPDTKKVHTIFNQALTQTGRLSSTEPNLQNI

PIRLEEGRKIRQAFVPSESDWLIFAADYSQIELRVLAHIAEDDNLMEAFRRDLDIHTKTAMDIFQV
SEDEVTPNMRRQAKAVNFGIVYGISDYGLAQNLNISRKEAAEFIERYFESFPGVKRYMENIVQEAK
QKGYVTTLLHRRRYLPDITSRNFNVRSFAERMAMNTPIQGSAADIIKKAMIDLNARLKEERLQAHL
LLQVHDELILEAPKEEMERLCRLVPEVMEQAVTLRVPLKVDYHYGSTWYDAK
>1KAFA
MEITSDMEEDKDLMLKLLDKNGFVLKKVEIYRSNYLAILEKRTNGIRNFEINNNGNMRIFGYKMME
HHIQKFTDIGMSCKIAKNGNVYLDIKRSAENIEAVITVASEL
>3DJLA
MHWQTHTVFNQPIPLNNSNLYLSDGALCEAVTREGAGWDSDFLASIGQQLGTAESLELGRLANVNP
PELLRYDAQGRRLDDVRFHPAWHLLMQALCTNRVHNLAWEEDARSGAFVARAARFMLHAQVEAGSL
CPITMTFAATPLLLQMLPAPFQDWTTPLLSDRYDSHLLPGGQKRGLLIGMGMTEKQGGSDVMSNTT
RAERLEDGSYRLVGHKWFFSVPQSDAHLVLAQTAGGLSCFFVPRFLPDGQRNAIRLERLKDKLGNR
SNASCEVEFQDAIGWLLGLEGEGIRLILKMGGMTRFDCALGSHAMMRRAFSLAIYHAHQRHVFGNP
LIQQPLMRHVLSRMALQLEGQTALLFRLARAWDRRADAKEALWARLFTPAAKFVICKRGMPFVAEA
MEVLGGIGYCEESELPRLYREMPVNSIWEGSGNIMCLDVLRVLNKQAGVYDLLSEAFVEVKGQDRY
FDRAVRRLQQQLRKPAEELGREITHQLFLLGCGAQMLKYASPPMAQAWCQVMLDTRGGVRLSEQIQ
NDLLLRATGGVCV
>3BXEA
SNAKDVLGLTLLEKTLKERLNLKDAIIVSGDSDQSPWVKKEMGRAAVACMKKRFSGKNIVAVTGGT
TIEAVAEMMTPDSKNRELLFVPARGGLGEDVKNQANTICAHMAEKASGTYRLLFVPGQLSQGAYSS
IIEEPSVKEVLNTIKSASMLVHGIGEAKTMAQRRNTPLEDLKKIDDNDAVTEAFGYYFNADGEVVH
KVHSVGMQLDDIDAIPDIIAVAGGSSKAEAIEAYFKKPRNTVLVTDEGAAKKLLRDE
>3F1NB
GEFKGLNVCQPTRFISRHNIEGIFTFVDHRCVATVGYQPQELLGKNIVEFCHPEDQQLLRDSFQQV
VKLKGQVLSVMFRFRSKNQEWLWMRTSSFTFQNPYSDEIEYIICTNTNVKNSSQE
>1F1EA
MAVELPKAAIERIFRQGIGERRLSQDAKDTIYDFVPTMAEYVANAAKSVLDASGKKTLMEEHLKAL
ADVLMVEGVEDYDGELFGRATVRRILKRAGIERASSDAVDLYNKLICRATEELGEKAAEYADEDGR
KTVQGEDVEKAITYSMPKGGEL
>3IXSA
ASEIELVFRPHPTLMEKDDSAQTRYIKTSGNATVDHLSKYLAVRLALEELRSKGESNQMNLDTASE
KQYTIYIATASGQFTVLDGSFSLELVSEKYWKVNKPMELYYAPTK
>3GJ7B
GPLGSAGSSWQCDTCLLQNKVTDNKCIACQAAKLPLKETAKQTGIGTPSKSDKPASTSGTGFGDKF
KPAIGTWDCDTCLVQNKPEAVKCVACETPKPG
>1EANA
SGDRSMVEVLADHPGELVRTDSPNFLSSVLPTHWRSNKTLPIAFKVVALGDVPDGTLVTVMAGNDE
NYSAELRNATAAMKNQVARFNDLRFVGRSGRGKSFTLTITVFTNPPQVATYHRAIKITVDGPREPR
RHRQKLDD
>3HQRA
PNGQTKPLPALKLALEYIVPCMNKHGICVVDDFLGKETGQQIGDEVRALHDTGKFTDGQLVSQKSD
SSKDIRGDKITWIEGKEPGCETIGLLMSSMDDLIRHCNGKLGSYKINGRTKAMVACYPGNGTGYVR
HVDNPNGDGRCVTCIYYLNKDWDAKVSGGILRIFPEGKAQFADIEPKFDRLLFFWSDRRNPHEVQP
AYATRYAITVWYFDADERAAAKVKYLTGEKGVRVELNKPSDSVGKDVF

>2HQLA
GGGGGGMLNRVFLEGEIESSCWSVKKTGFLVTIKQMRFFGERLFTDYYVIYANGQLAYELEKHTKK
YKTISIEGILRTYLERKSEIWKTTIEIVKIFNPKNEIVIDYKEI
>3HQMA
GSGGSGKVVKFSYMWTINNFSFCREEMGEVIKSSTFSSGANDKLKWCLRVNPKGLDEESKDYLSLY
LLLVSCPKSEVRAKFKFSILNAKGEETKAMESQRAYRFVQGKDWGFKKFIRRGFLLDEANGLLPDD
KLTLFCEVSVVQD
>3OQGA
MGHHHHHHEFMAKRKSDIILKSVDDLKDEIDYKDFEYKEYFNLLCELVPNNSLEKLEINAIDEKNM
KNEGLVYYVFVIQGKIFKIGHSITPITKRVQSYNCGKVEYRKNGTCSTTNYFVLQSLLKINKIVQVY
AFFPEQPTYTLFGKTYQDSFSTSKRAENVILENFIKNHNKKPIGCTQT
>1OQJA
GAMEDMEIAYPITCGESKAILLWKKFVCPGINVKCVKFNDQLISPKHFVHLAGKSTLKDWKRAIRL
GGIMLRKMMDSGQIDFYQHDKVCSNTCRSTK
>3FC3A
MGHHHHHHEFMLKNDDFVIAKNQLGNIVPNSVGVIRAVNGKSAMVLFIGLNELKRVDFSELEAIDI
YRTGKGYDKKICNICHILKNTDGFEINQTDAKGRKTTRPSCRECRKNIDGVKLSSTEKKKMDEIAP
PKGSVFTCPICEKRSIVGVTANLVHDHNHDTGWGREWICDSCNTGLGRFKDNPKFLEKVIEYLKKY
EK
>2NX4A
GVPKLVDHDERRRSITAAAWRLIAARGIEAANMRDIATEAGYTNGALSHYFAGKDEILRTSYEHIS
EATDRRIAEALGDATGLDALRILCREVMPINEEQLLEARIAASLWPRAMYDEQMAATNRRTMDNWR
EQMAIFLEQAREEGSVGDIDVTIVVEQLLNMMMGMQILGVLTPGETSSERQLEMLEQFVAAL
>3HI2B
GSHMEKRTPHTRLSQVKKLVNAGQVRTTRSALLNADELGLDFDGMCNVIIGLSESDFYKSMTTYSD
HTIWQDVYRPRLVTGQVYLKITVIHDVLIVSFKEK
>2HINA
MKPEELVRHFGDVEKAAVGVGVTPGAVYQWLQAGEIPPLRQSDIEVRTAYKLKSDFTSQRMGKEGH
NSGTK
>2EWTA
MSSEYAKQLGAKLRAIRTQQGLSLHGVEEKSQGRWKAVVVGSYERGDRAVTVQRLAELADFYGVPV
QELLP
>2Z4PA
MVTAFILMVTAAGKEREVMEKLLAMPEVKEAYVVYGEYDLIVKVETDTLKDLDQFITEKIRKMPEI
QMTSTMIAI
>2CBOA
AAPTATVTPSSGLSDGTVVKVAGAGLQAGTAYWVAQWARVDTGVWAYNPADNSSVTADANGSASTS
LTVRRSFEGFLFDGTRWGTVDCTTAACQVGLSDAAGNGPEGVAISFNHH
>3CBMA
KDNIRHGVCWIYYPDGGSLVGEVNEDGEMTGEKIAYVYPDERTALYGKFIDGEMIEGKLATLMSTE
EGRPHFELMPGNSVYHFDKSTSSCISTNALLPDPYESERVYVAESLISSAGEGLFSKVAVGPNTVM
SFYNGVRITHQEVDSRDWALNGNTLSLDEETVIDVPEPYNHVSKYCASLGHKANHSFTPNCIYDMF
VHPRFGPIKCIRTLRAVEADEELTVAYGYDHSPPGKSGPEAPEWYQVELKAFQATQQK
>2NMUA

MAGDPNSMTVSHHNASTARFYALRLLPGQEVFSQLHAFVQQNQLRAAWIAGCTGSLTDVALRYAGQ
EATTSLTGTFEVISLNGTLELTGEHLHLAVSDPYGVMLGGHMMPGCTVRTTLELVIGELPALTFSR
QPCAISGYDELHISSRLEHHHHHH
>3EIKA
GSSHHHHHHSSGLVPRGSHMMDAPDISYEHQETSVPNRSGIIPTLQNVVATVNLSCKLDLKNIALR
ARNAEYNPKRFAAVIMRIREPKTTALIFASGKMVITGAKSEKSSRMAAQRYAKIIHKLGFNATFDD
FKIQNIVSSCDIKFSIRLEGLAYAHSNYCSYEPELFPGLIYRMVKPKIVLLIFVSGKIVLTGAKVR
DDIYQAFNNIYPVLIQHRKA
>1OR7A
GSHMSEQLTDQVLVERVQKGDQKAFNLLVVRYQHKVASLVSRYVPSGDVPDVVQEAFIKAYRALDS
FRGDSAFYTWLYRIAVNTAKNYLVAQGRRPPSSDVDAIEAENFESGGALKEISNPENLMLSEELRQ
IVFRTIESLPEDLRMAITLRELDGLSYEEIAAIMDCPVGTVRSRIFRAREAIDNKVQPLIRR
>2GX5A
MGSSHHHHHHMALLQKTRIINSMLQAAAGKPVNFKEMAETLRDVIDSNIFVVSRRGKLLGYSINQQ
IENDRMKKMLEDRQFPEEYTKNLFNVPETSSNLDINSEYTAFPVENRDLFQAGLTTIVPIIGGGER
LGTLILSRLQDQFNDDDLILAEYGATVVGMEILREKAE
>2W42A
MMEYKIVENGLTYRIGNGASVPISNTGELIKGLRNYGPYEVPSLKYNQIALIHNNQFSSLINQLKS
QISSKIDEVWHIHNINISEFIYDSPHFDSIKSQVDNAIDTGVDGIMLVLPEYNTPLYYKLKSYLIN
SIPSQFMRYDILSNRNLTFYVDNLLVQFVSKLGGKPWILNVDPEKGSDIIIGTGATRIDNVNLFCF
AMVFKKDGTMLWNEISPIVTSSEYLTYLKSTIKKVVYGFKKSNPDWDVEKLTLHVSGKRPKMKDGE
TKILKETVEELKKQEMVSRDVKYAILHLNETHPFWVMGDPNNRFHPYEGTKVKLSSKRYLLTLLQP
YLKRNGLEMVTPIKPLSVEIVSDNWTSEEYYHNVHEILDEIYYLSKMNWRGFRSRNLPVTVNYPKL
VAGIIANVNRYGGYPINPEGNRSLQTNPWFL
>3FGHA
GKPKRPRSAYNVYVAERFQEAKGDSPQEKLKTVKENWKNLSDSEKELYIQHAKEDETRYHNEMKSW
A
>2B29A
GSHMVGQLSEGAIAAIMQKGDTNIKPILQVINIRPITTGNSPPRYRLLMSDGLNTLSSFMLATQLN
PLVEEEQLSSNCVCQIHRFIVNTLKDGRRVVILMELEVLKSAEAVGVKIGNPVPYNE
>3K6GA
SEEKVSQPEVGAAIKIIRQLMEKFNLDLSTVTQAFLKNSGELEATSAFLASGQRADGYPIWSRQDD
IDLQKDDEDTREALVKKFGAQNVARRIEFRKKGGSGGSGGSGGKL
>1SE8A
MARGMNHVYLIGALARDPELRYTGNGMAVFEATVAGEDRVIGNDGRERNLPWYHRVSILGKPAEWQ
AERNLKGGDAVVVEGTLEYRQWEAPEGGKRSAVNVKALRMEQLGTQPELIQDAGGGVRMSGAMNEV
LVLGNVTRDPEIRYTPAGDAVLSLSIAVNENYQDRQGQRQEKVHYIDATLWRDLAENMKELRKGDP
VMIMGRLVNEGWTDKDGNKRNSTRVEATRVEALARGAGNANSGYAAATPAAPRTQTASSAARPTSG
GYQSQPSRAANTGSRSGGLDIDQGLDDFPPEEDDLPF
>1BM9A
MKEEKRSSTGFLVKQRAFLKLYMITMTEQERLYGLKLLEVLRSEFKEIGFKPNHTEVYRSLHELLD
DGILKQIKVKKEGAKLQEVVLYQFKDYEAAKLYKKQLKVELDRCKKLIEKALSDNF
>2QR3A
MSLGTIIIVDDNKGVLTAVQLLLKNHFSKVITLSSPVSLSTVLREENPEVVLLDMNFTSGINNGNE

GLFWLHEIKRQYRDLPVVLFTAYADIDLAVRGIKEGASDFVVKPWDNQKLLETLLNAASQAKDGKK
EGHHHHHH
>3LGFA
GPLGSNSFVGLRVVAKWSSNGYFYSGKITRDVGAGKYKLLFDDGYECDVLGKDILLCDPIPLDTEV
TALSEDEYFSAGVVKGHRKESGELYYSIEKEGQRKWYKRMAVILSLEQGNRLREQYGLG
>3LGBA
SDEINAQSVWSEEISSNYPLCIKNLMEGLKKNHHLRYYGRQQLSLFLKGIGLSADEALKFWSEAFT
NMTMEKFNKEYRYSFRHNYGLEGNRINYKPWDCHTILSKPRPGRGDYHGCPFRDWSHERLSAELRS
MKLTQAQIISVLDSCQKGEYTIACTKVFEMTHNSASADLEIGEQTHIAHPNLYFERSRQLQK
>1UB4A
VSRYVPDMGDLIWVDFDPTKGSEQAGHRPAVVLSPFMYNNKTGMCLCVPCTTQSKGYPFEVVLSGQ
ERDGVALADQVKSIAWRARGATKKGTVAPEELQLIKAKINVLIG
>2AUWA
GHMNEYFFPKLTAVEALAPYRLRTTWSTGEVLEVDVGDILRKIPDLAPILDPEAFARVHIAEWEGS
VEWFDTEFGRDNVYAWAKEQAGEVSHEMFGDWMHRNNLSLTTAAEALGISRRMVSYYRTAHKIIPR
TIWLACLGWEATRPETKTLPRTLPAAYAKGVSASLSGS
>2G9WA
MAKLTRLGDLERAVMDHLWSRTEPQTVRQVHEALSARRDLAYTTVMAVLQRLAKKNLVLQIRDDRA
HRYAPVHGRDELVAGLMVDALAQAEDSGSRQAALVHFVERVGADEADALRRALAELEAGHGNRPPA
GAATET
>3KT9A
MMRVCWLVRQDSRHQRIRLPHLEAVVIGRGPETKITDKKCSRQQVQLKAECNKGYVKVKQVGVNPT
SIDSVVIGKDQEVKLQPGQVLHMVNELYPYIVEFEE
>3GBGA
MIGKKSFQTNVYRMSKFDTYIFNNLYINDYKMFWIDSGIAKLIDKNCLVSYEINSSSIILLKKNSI
QRFSLTSLSDENINVSVITISDSFIRSLKSYILGDLMIRNLYSENKDLLLWNCEHNDIAVLSEVVN
GFREINYSDEFLKVFFSGFFSKVEKKYNSIFITDDLDAMEKISCLVKSDITRNWRWADICGELRTN
RMILKKELESRGVKFRELINSIRISYSISLMKTGEFKIKQIAYQSGFASVSYFSTVFKSTMNVAPS
EYLFMLTGVAEK
>3E61A
MSLYKRKSKLIGLLLPDMSNPFFTLIARGVEDVALAHGYQVLIGNSDNDIKKAQGYLATFVSHNCT
GMISTAFNENIIENTLTDHHIPFVFIDRINNEHNGISTNHFKGGQLQAEVVRKGKGKNVLIVHENL
LIDAFHQRVQGIKYILDQQRIDYKMLEATLLDNDKKFIDLIKELSIDSIICSNDLLAINVLGIVQR
YHFKVPAEIQIIGYDNIPFSEMTYPQITTIDQSAYHLGEIAVSQLLGLNTDNLTNNHKQLALTVKH
RGSTREGHHHHHH
>1A73A
MALTNAQILAVIDSWEETVGQFPVITHHVPLGGGLQGTLHCYEIPLAAPYGVGFAKNGPTRWQYKR
TINQVVHRWGSHTVPFLLEPDNINGKTCTASHLCHNTRCHNPLHLCWESLDDNKGRNWCPGPNGGC
VHAVVCLRQGPLYGPGATVAGPQQRGSHFVV
>3IAYA
MGTQLESTFEQDVSQMEHDMADQEEHDLSSFERKKLPTDFDPSLYDISFQQIDAEQSVLNGIKDEN
TSTVVRFFGVTSEGHSVLCNVTGFKNYLYVPAPNSSDANDQEQINKFVHYLNETFDHAIDSIEVVS
KQSIWGYSGDTKLPFWKIYVTYPHMVNKLRTAFERGHLSFNSWFSNGTTTYDNIAYTLRLMVDCGI
VGMSWITLPKGKYSMIEPNNRVSSCQLEVSINYRNLIAHPAEGDWSHTAPLRIMSFDIECAGRIGV

FPEPEYDPVIQIANVVSIAGAKKPFIRNVFTLNTCSPITGSMIFSHATEEEMLSNWRNFIIKVDPD
VIIGYNTTNFDIPYLLNRAKALKVNDFPYFGRLKTVKQEIKESVFSSKAYGTRETKNVNIDGRLQL
DLLQFIQREYKLRSYTLNAVSAHFLGEQKEDVHYSIISDLQNGDSETRRRLAVYCLKDAYLPLRLM
EKLMALVNYTEMARVTGVPFSYLLARGQQIKVVSQLFRKCLEIDTVIPNMQSQASDDQYEGATVIE
PIRGYYDVPIATLDFNSLYPSIMMAHNLCYTTLCNKATVERLNLKIDEDYVITPNGDYFVTTKRRR
GILPIILDELISARKRAKKDLRDEKDPFKRDVLNGRQLALKISANSVYGFTGATVGKLPCLAISSS
VTAYGRTMILKTKTAVQEKYCIKNGYKHDAVVVYGDTDSVMVKFGTTDLKEAMDLGTEAAKYVSTL
FKHPINLEFEKAYFPYLLINKKRYAGLFWTNPDKFDKLDQKGLASVRRDSCSLVSIVMNKVLKKIL
IERNVDGALAFVRETINDILHNRVDISKLIISKTLAPNYTNPQPHAVLAERMKRREGVGPNVGDRV
DYVIIGGNDKLYNRAEDPLFVLENNIQVDSRYYLTNQLQNPIISIVAPIIGDKQANGMFVV
>3IA8A
PPKMNPVVEPLSWMLGTWLSDPPGAGTYPTLQPFQYLEEVHISHVGQPMLNFSFNSFHPDTRKPMH
RECGFIRLKPDTNKVAFVSAQNTGVVEVEEGEVNGQELCIASHSIARISFAKEPHVEQITRKFRLN
SEGKLEQTVSMATTTQPMTQHLHVTYKKVTP
>3FXQA
MLKLQTLQALICIEEVGSLRAAAQLLHLSQPALSAAIQQLEDELKAPLLVRTKRGVSLTSFGQAFM
KHARLIVTESRRAQEEIGQLRGRWEGHITFAASPAIALAALPLALASFAREFPDVTVNVRDGMYPA
VSPQLRDGTLDFALTAAHKHDIDTDLEAQPLYVSDVVIVGQRQHPMANATRLAELQECRWAFSSAP
RGPGAIIRNAFARYGLPEPKLGLVCESFLALPGVVAHSDLLTTMPRTLYERNAFKDQLCSIPLQDA
LPNPTIYVLRRHDLPVTPAAAGLIRWIQHHALQTGHHHHHH
>1L8RA
GSQNNECKMVDLRGAKVASFTVEGCELICLPQAFDLFLKHLVGGLHTVYTKLKRLEITPVVCNVEQ
VRILRGLGAIQPGVNRCKLISRKDFETLYNDCTNA
>2Q79A
TTPIVHLKGDANTLKCLRYRFKKHCTLYTAVSSTWHWTGHNVKHKSAIVTLTYDSEWQRDQFLSQV
KIPKTITVSTGFMSIGGGTGGGSGGGS
>3CWRA
GMVEQRNRGRPAVPDAVVRESIVGAAQRLLSSGGAAAMTMEGVASEAGIAKKTLYRFASGRADLIG
LLVESWIAPIFPGFEADPQDAAAALERIVYDIAQAVLSREAVSLFRMLASDADLRNRFLPAYNANG
IERSRRELARWLDQQASAGRLPLPIPAERVADLLLSAVIAEPLRQITLGLREPLPAWDIAPRVADA
VRLIAPGRER
>1BGFA
GGSQWNQVQQLEIKFLEQVDQFYDDNFPMEIRHLLAQWIETQDWEVASNNETMATILLQNLLIQLD
EQLGRVSKEKNLLLIHNLKRIRKVLQGKFHGNPMHVAVVISNCLREERRILAAANMPI
>1T0FA
GSAMAKANSSFSEVQIARRIKEGRGQGHGKDYIPWLTVQEVPSSGRSHRIYSHKTGRVHHLLSDLE
LAVFLSLEWESSVLDIREQFPLLPSDTRQIAIDSGIKHPVIRGVDQVMSTDFLVDCKDGPFEQFAI
QVKPAAALQDERTLEKLELERRYWQQKQIPWFIFTDKEINPVVKENIEWLYSVKTEEVSAELLAQL
SPLAHILQEKGDENIINVCKQVDIAYDLELGKTLSEIRALTANGFIKFNIYKSFRANKCADLCISQ
VVNMEELRYVAN
>3JY6A
MSLTQSSKLIAVIVANIDDYFSTELFKGISSILESRGYIGVLFDANADIEREKTLLRAIGSRGFDG
LILQSFSNPQTVQEILHQQMPVVSVDREMDACPWPQVVTDNFEAAKAATTAFRQQGYQHVVVLTSE
LELSRTRQERYRGILAAAQDVDVLEVSESSYNHSEVHQRLTQLITQNDQKTVAFALKERWLLEFFP

NLIISGLIDNQTVTATGFADTDFIRRMEPKLTLITQNPFLMGASSAEIMLRQLAGEKVAPEKMVIP
AKLQEGHHHHHH
>2PI2A
MWNSGFESYGSSSYGGAGGYTQSPGGFGSPAPSQAEKKSRARAQHIVPCTISQLLSATLVDEVFRI
GNVEISQVTIVGIIRHAEKAPTNIVYKIDDMTAAPMDVRQWVDTDDTSSENTVVPPETYVKVAGHL
RSFQNKKSLVAFKIMPLEDMNEFTTHILEVINAHMVLSKANSQPSAGRAPISNPGMSEAGNFGGNS
FMPANGLTVAQNQVLNLIKACPRPEGLNFQDLKNQLKHMSVSSIKQAVDFLSNEGHIYSTVDDDHF
KSTDAE
>2PY5A
MKHMPRKMYSCAFETTTKVEDCRVWAYGYMNIEDHSEYKIGNSLDEFMAWVLKVQADLYFHNLKFA
GAFIINWLERNGFKWSADGLPNTYNTIISRMGQWYMIDICLGYKGKRKIHTVIYDSLKKLPFPVKK
IAKDFKLTVLKGDIDYHKERPVGYKITPEEYAYIKNDIQIIAEALLIQFKQGLDRMTAGSDSLKGF
KDIITTKKFKKVFPTLSLGLDKEVRYAYRGGFTWLNDRFKEKEIGEGMVFDVNSLYPAQMYSRLLP
YGEPIVFEGKYVWDEDYPLHIQHIRCEFELKEGYIPTIQIKRSRFYKGNEYLKSSGGEIADLWLSN
VDLELMKEHYDLYNVEYISGLKFKATTGLFKDFIDKWTYIKTTSEGAIKQLAKLMLNSLYGKFASN
PDVTGKVPYLKENGALGFRLGEEETKDPVYTPMGVFITAWARYTTITAAQACYDRIIYCDTDSIHL
TGTEIPDVIKDIVDPKKLGYWAHESTFKRAKYLRQKTYIQDIYMKEVDGKLVEGSPDDYTDIKFSV
KCAGMTDKIKKEVTFENFKVGFSRKMKPKPVQVPGGVVLVDDTFTIK
>1Y0UA
GHMSLEEWIKADSLEKADEYHKRYNYAVTNPVRRKILRMLDKGRSEEEIMQTLSLSKKQLDYHLKV
LEAGFCIERVGERWVVTDAGKIVDKIRGGS
>1CI4A
MTTSQKHRDFVAEPMGEKPVGSLAGIGEVLGKKLEERGFDKAYVVLGQFLVLKKDEDLFREWLKDT
CGANAKQSRDCFGCLREWCDAFL
>2UV0E
GAMALVDGFLELERSSGKLEWSAILQKMASDLGFSKILFGLLPKDSQDYENAFIVGNYPAAWREHY
DRAGYARVDPTVSHCTQSVLPIFWEPSIYQTRKQHEFFEEASAAGLVYGLTMPLHGARGELGALSL
SVEAENRAEANRFMESVLPTLWMLKDYALQSGAGLAFEHPVSK
>2O99A
GHMSRNLLAIVHPILRNLMEESGETVNMAVLDQSDHEAIIIDQVQCTHLMRMSAPIGGKLPMHASG
AGKAFLAQLSEEQVTKLLHRKGLHAYTHATLVSPVHLKEDLAQTRKRGYSFDDEEHALGLRCLAAC
IFDEHREPFAAISISGPISRITDDRVTEFGAMVIKAAKEVTLAYGGMRGS
>3G7RA
MGSSHHHHHHSSGRENLYFQGMSPSTEAAARTPSEARARLLGTATRIFYAEGIHSVGIDRITAEAQ
VTRATLYRHFSGKDDLILAYLDQADRGIRAQVTAARGSSPAADGQVRAVARSIVDGIRSPGFRGCA
FLNAVAEYPDPAHPVHRAVLAHRQWFLDTVTELLAQVGDGDGVAAGRHLVMLRDGAMAAGCLFDPE
LVSETFLHGVEGVLRDVSEKTSA
>3G7UA
MSLNVIDLFSGVGGLSLGAARAGFDVKMAVEIDQHAINTHAINFPRSLHVQEDVSLLNAEIIKGFF
KNDMPIDGIIGGPPCQGFSSIGKGNPDDSRNQLYMHFYRLVSELQPLFFLAENVPGIMQEKYSGIR
NKAFNLVSGDYDILDPIKVKASDYGAPTIRTRYFFIGVKKSLKLDISDEVFMPKMIDPVTVKDALY
GLPDIIDANWQSDSESWRTIKKDRKGGFYEKLWGQIPRNVGDTESIAKLKNNIISGCTGTLHSKIV
QERYASLSFGETDKISRSTRLDPNGFCPTLRAGTARDKGSFQAVRPIHPYHPRVITPREAARLQGF
PDWFRFHVTKWHSFRQIGNSVSPIVAEYILKGLYNLLNEGHHHHHH

>2G7OA
ESAFNQTEFNKLLLECVVKTQSSVAKILGIESLSPHVSGNSKFEYANMVEDIREKVSSEMERFFPK
NDDE
>3CNVA
VRYRFLRLAPDEEGEGGRAESRILECRRLRAPAEIARALELRAGETVVTIRRQLSMNHMPTVIDDL
WLPGTHFRGLTLELLTASKAPLYGLFESEFGVSMVRADEKLRAVAASPEIAPLLGVEPGRPLLQVD
RISYTYGDRPMEVRRGLYLTDHYHYRNSLN
>3KGKA
MKTLMVFDPAMAASTGVCGTDVDQALVDFSTDVQWLKQSGVQIERFNLAQQPMSFVQNEKVKAFIE
ASGAEGLPLLLLDGETVMAGRYPKRAELARWFGIPLDKVGLAPG
>1KGSA
MNVRVLVVEDERDLADLITEALKKEMFTVDVCYDGEEGMYMALNEPFDVVILDIMLPVHDGWEILK
SMRESGVNTPVLMLTALSDVEYRVKGLNMGADDYLPKPFDLRELIARVRALIRRKSESKSTKLVCG
DLILDTATKKAYRGSKEIDLTKKEYQILEYLVMNKNRVVTKEELQEHLWSFDDEVFSDVLRSHIKN
LRKKVDKGFKKKIIHTVRGIGYVARDE
>3DSHA
EQLLPDLLISPHMLPLTDLEIKFQYRGRPPRALTISNPHGCRLFYSQLEATQEQVELFGPISLEQV
RFPSPEDIPSDKQRFYTNQLLDVLDRGLILQLQGQDLYAIRLCQCKVFWSGPCASAHDSCPNPIQR
EVKTKLFSLEHFLNELILFQKGQTNTPPPFEIFFCFGEEWPDRKPREKKLITVQVVPVAARLLLEM
FSGELSWSADDIRLQISNPDLKDRMVEQFKELHHIWQSQQRLQPVAQA
>1J75A
GSHMLSTGDNLEQKILQVLSDDGGPVKIGQLVKKCQVPKKTLNQVLYRLKKEDRVSSPEPATWSIG
G
>3GPVA
MSLALYLGNKRMNDMYYTIGQVAKMQHLTISQIRYYDKQGLFPFLQRNEKGDRIFNEEALKYLEMI
LCLKNTGMPIQKIKQFIDWSMEGDSTILHRLKLMKQQEANVLQLIQDTEKNLKKIQQKIAKYEDEI
SSANATTKEGHHHHHH
>3GP4A
MSLNIKEASEKSGVSADTIRYYERIGLIPPIHRNESGVRKFGAEDLRWILFTRQMRRAGLSIEALI
DYLALFREGEHTLEARAELLKKQRIELKNRIDVMQEALDRLDFKIDNYDTHLIPAQEELKDFNVER
SNEGHHHHHH
>2R4GA
MKGFQFKVIQEKLQGRQFINSDKIKPDHPQTIIKKTLLKEYQSKNFSCQEERDLFLEFTEKIVQNF
HNINFNYLLKKFCKLPENYQSLKSQVKQIVQSENKANQQSCENLFNSLYDTEISYKQITNFLRQII
QNCVPNQLLGKKNFKVFLEKLYEFVQMKRFENQKVLDYICFMDVFDVEWFVDLKNQKFTQKRKYIS
DKRKILGDLIVFIINKIVIPVLRYNFYITEKHKEGSQIFYYRKPIWKLVSKLTIVKLEEENLEKVE
EKL
>3AL2A
GPLGSLKKQYIFQLSSLNPQERIDYCHLIEKLGGLVIEKQCFDPTCTHIVVGHPLRNEKYLASVAA
GKWVLHRSYLEACRTAGHFVQEEDYEWGSSSILDVLTGINVQQRRLALAAMRWRKKIQQRQESGIV
EGAFSGWKVILHVDQSREAGFKRLLQSGGAKVLPGHSVPLFKEATHLFSDLNKLKPDDSGVNIAEA
AAQNVYCLRTEYIADYLMQESPPHVENYCLPEAISFI
>2CMPA
GSHMKEPKLSPKQERFIEEYFINDMNATKAAIAAGYSKNSASAIGAENLQKPAIRARIDARLK

>1UCRA
MEEAKQKVVDFLNSKSGSKSKFYFNDFTDLFPDMKQREVKKILTALVNDEVLEYWSSGSTTMYGLK
GAGKQAAAEHED
>1AKOA
MKFVSFNINGLRARPHQLEAIVEKHQPDVIGLQETKVHDDMFPLEEVAKLGYNVFYHGQKGHYGVA
LLTKETPIAVRRGFPGDDEEAQRRIIMAEIPSLLGNVTVINGYFPQGESRDHPIKFPAKAQFYQNL
QNYLETELKRDNPVLIMGDMNISPTDLDIGIGEENRKRWLRTGKCSFLPEEREWMDRLMSWGLVDT
FRHANPQTADRFSWFDYRSKGFDDNRGLRIDLLLASQPLAECCVETGIDYEIRSMEKPSDHAPVWA
TFRR
>1X2IA
MEKKALTLAERQRLIVEGLPHVSATLARRLLKHFGSVERVFTASVAELMKVEGIGEKIAKEIRRVI
TAPYIEDEE
>3OIOA
SNAGSQPKLTEAVSLMEANIEEPLSTDDIAYYVGVSRRQLERLFKQYLGTVPSKYYLELRLNRARQ
LLQQTSKSIVQIGLACGFSSGPHFSSTYRNHFNITPREERAQRAQPG
>1U09A
GLIVDTRDVEERVHVMRKTKLAPTVAHGVFNPEFGPAALSNKDPRLNEGVVLDEVIFSKHKGDTKM
SAEDKALFRRCAADYASRLHSVLGTANAPLSIYEAIKGVDGLDAMEPDTAPGLPWALQGKRRGALI
DFENGTVGPEVEAALKLMEKREYKFACQTFLKDEIRPMEKVRAGKTRIVDVLPVEHILYTRMMIGR
FCAQMHSNNGPQIGSAVGCNPDVDWQRFGTHFAQYRNVWDVDYSAFDANHCSDAMNIMFEEVFRTE
FGFHPNAEWILKTLVNTEHAYENKRITVEGGMPSGCSATSIINTILNNIYVLYALRRHYEGVELDT
YTMISYGDDIVVASDYDLDFEALKPHFKSLGQTITPADKSDKGFVLGHSITDVTFLKRHFHMDYGT
GFYKPVMASKTLEAILSFARRGTIQEKLISVAGLAVHSGPDEYRRLFEPFQGLFEIPSYRSLYLRW
VNAVCGDAAALEHH
>1LJ9A
TDILREIGMIARALDSISNIEFKELSLTRGQYLYLVRVCENPGIIQEKIAELIKVDRTTAARAIKR
LEEQGFIYRQEDASNKKIKRIYATEKGKNVYPIIVRENQHSNQVALQGLSEVEISQLADYLVRMRK
NVSEDWEFVKKG
>2V1XA
MCLEDSDAGASNEYDSSPAAWNKEDFPWSGKVKDILQNVFKLEKFRPLQLETINVTMAGKEVFLVM
PTGGGKSLCYQLPALCSDGFTLVICPLISLMEDQLMVLKQLGISATMLNASSSKEHVKWVHAEMVN
KNSELKLIYVTPEKIAKSKMFMSRLEKAYEARRFTRIAVDEVHCCSQWGHDFRPDYKALGILKRQF
PNASLIGLTATATNHVLTDAQKILCIEKCFTFTASFNRPNLYYEVRQKPSNTEDFIEDIVKLINGR
YKGQSGIIYCFSQKDSEQVTVSLQNLGIHAGAYHANLEPEDKTTVHRKWSANEIQVVVATVAFGMG
IDKPDVRFVIHHSMSKSMENYYQESGRAGRDDMKADCILYYGFGDIFRISSMVVMENVGQQKLYEM
VSYCQNISKCRRVLMAQHFDEVWNSEACNKMCDNCCKDSAFERKNITEYCRDLIKILKQAEELNEK
LTPLKLIDSWMGKGAAKLRVAGVVAPTLPREDLEKIIAHFLIQQYLKEDYSFTAYATISYLKIGPK
ANLLNNEAHAITMQVTKSTQNSFRAESSQTCHSEQGDKKMEAENLYFQSHHHHHHDYKDDDDK
>3EQXA
GMEWQAEQAYNHLPPLPLDSKLAELAETLPILKACIPARAALAELKQAGELLPNQGLLINLLPLLE
AQGSSEIENIVTTTDKLFQYAQEDSQADPMTKEALRYRTALYQCFTQLSNRPLCVTTALEICSTIK
SVQMDVRKVPGTSLTNQATGEVIYTPPAGESVIRDLLSNWEAFLHNQDDVDPLIKMAMAHYQFEAI
HPFIDGNGRTGRVLNILYLIDQQLLSAPILYLSRYIVAHKQDYYRLLLNVTTQQEWQPWIIFILNA
VEQTAKWTTHKIAAARELIAHTTEYVRQQLPKIYSHELVQVIFEQPYCRIQNLVESGLAKRQTASV

YLKQLCDIGVLEEVQSGKEKLFVHPKFVTLMTKDSNQFSRYAL
>3HTSB
GSRRASVGSMARPAFVNKLWSMVNDKSNEKFIHWSTSGESIVVPNRERFVQEVLPKYFKHSNFASF
VRQLNMYGWHKVQDVKSGSMLSNNDSRWEFENERHA
>1VJFA
MGSDKIHHHHHHMKTRADLFAFFDAHGVDHKTLDHPPVFRVEEGLEIKAAMPGGHTKNLFLKDAKG
QLWLISALGETTIDLKKLHHVIGSGRLSFGPQEMMLETLGVTPGSVTAFGLINDTEKRVRFVLDKA
LADSDPVNFHPLKNDATTAVSQAGLRRFLAALGVEPMIVDFAAMEVVG
>1NLIA
MDVSFRLSGATSSSYGVFISNLRKALPNERKLYDIPLLRSSLPGSQRYALIHLTNYADETISVAID
VTNVYIMGYRAGDTSYFFNEASATEAAKYVFKDAMRKVTLPYSGNYERLQTAAGKIRENIPLGLPA
LDSAITTLFYYNANSAASALMVLIQSTSAAARYKFIEQQIGKRVDKTFLPSLAIISLANSWSALSK
QIQIASTNNGQFESPVVLINAQNQRVTITNVDAGVVTSNIALLLNRNNMA
>3KJOA
MSLVPATNYIYTPLNQLKGGTIVNVYGVVKFFKPPYLSKGTDYCSVVTIVDQTNVKLTCLLFSGNY
EALPIIYKNGDIVRFHRLKIQVYKKETQGITSSGFASLTFEGTLGAPIIPRTSSKYFNFTTEDHKM
VEALRVWASTHMSPSWTLLKLCDVQPMQYFDLTCQLLGKAEVDGASFLLKVWDGTRTPFPSWRVLI
QDLVLEGDLSHIHRLQNLTIDILVYDNHVHVARSLKVGSFLRIYSLHTKLQSMNSENQTMLSLEFH
LHGGTSYGRGIRVLPESNSDVDQLKKDLESANLTA
>1UFIA
GSHMPVPSFGEAMAYFAMVKRYLTSFPIDDRVQSHILHLEHDLVHVTRKNHARQAGVRGLGHQS
>3FRQA
GMPRPKLKSDDEVLEAATVVLKRCGPIEFTLSGVAKEVGLSRAALIQRFTNRDTLLVRMMERGVEQ
VRHYLNAIPIGAGPQGLWEFLQVLVRSMNTRNDFSVNYLISWYELQVPELRTLAIQRNRAVVEGIR
KRLPPGAPAAAELLLHSVIAGATMQWAVDPDGELADHVLAQIAAILCLMFPEHDDFQLLQAHA
>1MK0A
MKSGIYQIKNTLNNKVYVGSAKDFEKRWKRHFKDLEKGCHSSIKLQRSFNKHGNVFECSILEEIPY
EKDLIIERANFWIKELNSKINGYNIADATFG
>2FPHX
GIYQHFSIEDRPFLDKGMEWIKKVEDSYAPFLTPFINPHQEKLLKILAKTYGLACSSSGEFVSSEY
VRVLLYPDYFQPEFSDFEISLQEIVYSNKFEYLTHAKILGTVINQLGIERKLFGDILVDEERAQIM
INQQFLLLFQDGLKKIGRIPVSLEERPFTEKID
>3FYMA
MKTVGEALKGRRERLGMTLTELEQRTGIKREMLVHIENNEFDQLPNKNYSEGFIRKYASVVNIEPN
QLIQAHQDEIPSNQAEWDEVITVFNNNKDLDYKSKSKEPIQLLVIMGITVLITLLLWIMLVLIF
>2W7NA
MKKRLTESQFQEAIQGLEVGQQTIEIARGVLVDGKPQATFATSLGLTRGAVSQAVHRVWAAFEDKN
LPEGYARVTAVLPEHQAYIVRKWEADAKKKQETKR
>2J6VA
MGHHHHHHHHHHSSGHIEGRHMIRLGYPCENLTLGATTNRTLRLAHLTEERVREKAAENLRDLERI
LRFNADHGFALFRIGQHLIPFASHPLFPYDWEGAYEEELARLGALARAFGQRLSMHPGQYVNPGSP
DPEVVERSLAELRYSARLLSLLGAEDGVLVLHLGGAYGEKGKALRRFVENLRGEEEVLRYLALEND
ERLWNVEEVLKAAEALGVPVVVDTLHHALNPGRLPLEEALRLAFPTWRGRPXKVHLASQDPKKRPG
AHAFRVTREDWERLLSALPGPADVMVEAKGKEQGLATP

>1D02A
MGKSELSGRLNWQALAGLKASGAEQNLYNVFNAVFEGTKYVLYEKPKHLKNLYAQVVLPDDVIKEI
FNPLIDLSTTQWGVSPAFAIENTETHKILFGEIKRQDGWVEGKDPSAGRGNAHERSCKLFTPGLLK
AYRTIGGINDEEILPFWVVFEGDITRDPKRVREITFWYDHYQDNYFMWRPNESGEKLVQHFNEKLK
KYLD
>3KF6A
SLENENQLTHQFPTLSRWNPMFISDVHKISFHPHLQRYIGFWMGFPIRWIQIVGYIAAIDIYEGKH
VLTVDDCSGMVLRVVFIIQDDFSMSKRAISMSPGNVVCVFGKINSFRSEVELIAQSFEELRDPNDE
WKAWQKRMRYKKNLTKISKNHHSIIRT

(2) 212 non DNA-binding proteins

>3CGIA
MERQPTTDRMIQEYVPGKQVTLAHLIANPGKDLFKKLGLQDAVSAIGILTITPSEASIIACDIATK
SGAVEIGFLDRFTGAVVLTGDVSAVEYALKQVTRTLGEMMQFTTCSITRTLEHHHHHH
>1RQWA
ATFEIVNRCSYTVWAAASKGDAALDAGGRQLNSGESWTINVEPGTKGGKIWARTDCYFDDSGSGIC
KTGDCGGLLRCKRFGRPPTTLAEFSLNQYGKDYIDISNIKGFNVPMDFSPTTRGCRGVRCAADIVG
QCPAKLKAPGGGCNDACTVFQTSEYCCTTGKCGPTEYSRFFKRLCPDAFSYVLDKPTTVTCPGSSN
YRVTFCPTA
>1H2GB
SNMWVIGKSKAQDAKAIMVNGPQFGWYAPAYTYGIGLHGAGYDVTGNTPFAYPGLVFGHNGVISWG
STAGLGDDVDIFAERLSAEKPGYYLHNGKWVKMLSREETITVKNGQAETFTVWRTVHGNILQTDQT
TQTAYAKSRAWDGKEVASLLAWTHQMKAKNWQEWTQQAAKQALTINWYYADVNGNIGYVHTGAYPD
RQSGHDPRLPVPGTGKWDWKGLLPFEMNPKVYNPQSGYIANWNNSPQKDYPASDLFAFLWGGADRV
TEIDRLLEQKPRLTADQAWDVIRQTSRQDLNLRLFLPTLQAATSGLTQSDPRRQLVETLTRWDGIN
LLNDDGKTWQQPGSAILNVWLTSMLKRTVVAAVPMPFDKWYSASGYETTQDGPTGSLNISVGAKIL
YEAVQGDKSPIPQAVDLFAGKPQQEVVLAALEDTWETLSKRYGNNVSNWKTPAMALTFRANNFFGV
PQAAAEETRHQAEYQNRGTENDMIVFSPTTSDRPVLAWDVVAPGQSGFIAPDGTVDKHYEDQLKMY
ENFGRKSLWLTKQDVEAHKESQEVLHVQR
>1A12A
RRSPPADAIPKSKKVKVSHRSHSTEPGLVLTLGQGDVGQLGLGENVMERKKPALVSIPEDVVQAEA
GGMHTVCLSKSGQVYSFGCNDEGALGRDTSVEGSEMVPGKVELQEKVVQVSAGDSHTAALTDDGRV
FLWGSFRDNNGVIGLLEPMKKSMVPVQVQLDVPVVKVASGNDHLVMLTADGDLYTLGCGEQGQLGR
VPELFANRGGRQGLERLLVPKCVMLKSRGSRGHVRFQDAFCGAYFTFAISHEGHVYGFGLSNYHQL
GTPGTESCFIPQNLTSFKNSTKSWVGFSGGQHHTVCMDSEGKAYSLGRAEYGRLGLGEGAEEKSIP
TLISRLPAVSSVACGASVGYAVTKDGRVFAWGMGTNYQLGTGQDEDAWSPVEMMGKQLENRVVLSV
SSGGQHTVLLVKDKEQS
>3INGA
GMKEIRIILMGTGNVGLNVLRIIDASNRRRSAFSIKVVGVSDSRSYASGRNLDISSIISNKEKTGR
ISDRAFSGPEDLMGEAADLLVDCTPASRDGVREYSLYRMAFESGMNVVTANKSGLANKWHDIMDSA
NQNSKYIRYEATVAGGVPLFSVLDYSILPSKVKRFRGIVSSTINYVIRNMANGRSLRDVVDDAIKK
GIAESNPQDDLNGLDAARKSVILVNHIFGTEYTLNDVEYSGVDERSYNANDRLVTEVYVDDRRPVA
VSRIISLNKDDFLMSIGMDGLGYQIETDSNGTVNVSDIYDGPYETAGAVVNDILLLSKVQK
>2RAUA
GMYEEWKIVKREAPILGNDQLIENIWKMKREDSPYDIISLHKVNLIGGGNDAVLILPGTWSSGEQL

VTISWNGVHYTIPDYRKSIVLYLARNGFNVYTIDYRTHYVPPFLKDRQLSFTANWGWSTWISDIKE
VVSFIKRDSGQERIYLAGESFGGIAALNYSSLYWKNDIKGLILLDGGPTKHGIRPKFYTPEVNSIE
EMEAKGIYVIPSRGGPNNPIWSYALANPDMPSPDPKYKSISDFLMDSLYVTGSANPYDYPYSKKED
MFPILASFDPYWPYRLSLERDLKFDYEGILVPTIAFVSERFGIQIFDSKILPSNSEIILLKGYGHL
DVYTGENSEKDVNSVVLKWLSQQR
>1UAIA
AEPCDYPAQQLDLTDWKVTLPIGSSGKPSEIEQPALDTFATAPWFQVNAKCTGVQFRAAVNGVTTS
GSGYPRSELREMTDGGEEKASWSATSGTHTMVFREAFNHLPEVKPHLVGAQIHDGDDDVTVFRLEG
TSLYITKGDDTHHKLVTSDYKLNTVFEGKFVVSGGKIKVYYNGVLQTTISHTSSGNYFKAGAYTQA
NCSNSSPCSSSNYGQVSLYKLQVTHS
>3H5QA
SNAMRMIDIIEKKRDGHTLTTEEINFFIGGYVKGDIPDYQASSLAMAIYFQDMNDDERVALTMAMV
NSGDMIDLSDIKGVKVDKHSTGGVGDTTTLVLAPLVAAVDVPVAKMSGRGLGHTGGTIDKLEAIDG
FHVEIDEATFVKLVNENKVAVVGQSGNLTPADKKLYALRDVTGTVNSIPLIASSIMSKKIAAGADA
IVLDVKTGSGAFMKTLEDAEALAHAMVRIGNNVGRNTMAIISDMNQPLGRAIGNALELQEAIDTLK
GQGPKDLTELVLTLGSQMVVLANKAETLEEARALLIEAINSGAALEKFKTFIKNQGGDETVIDHPE
RLPQAQYQIEYKAKKSGYVTELVSNDIGVASMMLGAGRLTKEDDIDLAVGIVLNKKIGDKVEEGES
LLTIHSNRQDVDDVVKKLDSSITIADHVVSPTLIHKIITE
>3N20A
MSMLKREDWYDLTRTTNWTPKYVTENELFPEEMSGARGISMEAWEKYDEPYKITYPEYVSIQREKD
SGAYSIKAALERDGFVDRADPGWVSTMQLHFGAIALEEYAASTAEARMARFAKAPGNRNMATFGMM
DENRHGQIQLYFPYANVKRSRKWDWAHKAIHTNEWAAIAARSFFDDMMMTRDSVAVSIMLTFAFET
GFVNMQFLGLAADAAEAGDHTFASLISSIQTDESRHAQQGGPSLKILVENGKKDEAQQMVDVAIWR
SWKLFSVLTGPIMDYYTPLESRNQSFKEFMLEWIVAQFERQLLDLGLDKPWYWDQFMQDLDETHHG
MHLGVWYWRPTVWWDPAAGVSPEEREWLEEKYPGWNDTWGQCWDVITDNLVNGKPELTVPETLPTI
CNMCNLPIAHTPGNKWNVKDYQLEYEGRLYHFGSEADRWCFQIDPERYENHTNLVDRFLKGEIQPA
DLAGALMYMSLEPGVMGDDAHDYEWVKAYQKKTNAA
>3N2BA
MHHHHHHSSGVDLGTENLYFQSNAMDYFNYQEDGQLWAEQVPLADLANQYGTPLYVYSRATLERHW
HAFDKSVGDYPHLICYAVKANSNLGVLNTLARLGSGFDIVSVGELERVLAAGGDPSKVVFSGVGKT
EAEMKRALQLKIKCFNVESEPELQRLNKVAGELGVKAPISLRINPDVDAKTHPYISTGLRDNKFGI
TFDRAAQVYRLAHSLPNLDVHGIDCHIGSQLTALAPFIDATDRLLALIDSLKAEGIHIRHLDVGGG
LGVVYRDELPPQPSEYAKALLDRLERHRDLELIFEPGRAIAANAGVLVTKVEFLKHTEHKNFAIID
AAMNDLIRPALYQAWQDIIPLRPRQGEAQTYDLVGPVCETSDFLGKDRDLVLQEGDLLAVRSSGAY
GFTMSSNYNTRPRVAEVMVDGNKTYLVRQREELSSLWALESVLPE
>1P0WA
TIKEMPQPKTFGELKNLPLLNTDKPVQALMKIADELGEIFKFEAPGRVTRYLSSQRLIKEACDESR
FDKNLSQALKFVRDFAGDGLFTSWTHEKNWKKAHNILLPSFSQQAMKGYHAMMVDIAVQLVQKWER
LNADEHIEVPEDMTRLTLDTIGLCGFNYRFNSFYRDQPHPFITSMVRALDEAMNKLQRANPDDPAY
DENKRQFQEDIKVMNDLVDKIIADRKASGEQSDDLLTHMLNGKDPETGEPLDDENIRYQIITFLIA
GHETTSGLLSFALYFLVKNPHVLQKAAEEAARVLVDPVPSYKQVKQLKYVGMVLNEALRLWPTAPA
FSLYAKEDTVLGGEYPLEKGDELMVLIPQLHRDKTIWGDDVEEFRPERFENPSAIPQHAFKPWGNG
QRACIGQQFALHEATLVLGMMLKHFDFEDHTNYELDIKETLTLKPEGFVVKAKSKKIPL
>1UJ0A

AGHMARRVRALYDFEAVEDNELTFKHGELITVLDDSDANWWQGENHRGTGLFPSNFVTTDLS
>1UJMA
AKIDNAVLPEGSLVLVTGANGFVASHVVEQLLEHGYKVRGTARSASKLANLQKRWDAKYPGRFETA
VVEDMLKQGAYDEVIKGAAGVAHIASVVSFSNKYDEVVTPAIGGTLNALRAAAATPSVKRFVLTSS
TVSALIPKPNVEGIYLDEKSWNLESIDKAKTLPESDPQKSLWVYAASKTEAELAAWKFMDENKPHF
TLNAVLPNYTIGTIFDPETQSGSTSGWMMSLFNGEVSPALALMPPQYYVSAVDIGLLHLGCLVLPQ
IERRRVYGTAGTFDWNTVLATFRKLYPSKTFPADFPDQGQDLSKFDTAPSLEILKSLGRPGWRSIE
ESIKDLVGSETA
>1PAHA
TVPWFPRTIQELDRFANQILSYGAELDADHPGFKDPVYRARRKQFADIAYNYRHGQPIPRVEYMEE
EKKTWGTVFKTLKSLYKTHACYEYNHIFPLLEKYCGFHEDNIPQLEDVSQFLQTCTGFRLRPVAGL
LSSRDFLGGLAFRVFHCTQYIRHGSKPMYTPEPDICHELLGHVPLFSDRSFAQFSQEIGLASLGAP
DEYIEKLATIYWFTVEFGLCKQGDSIKAYGAGLLSSFGELQYCLSEKPKLLPLELEKTAIQNYTVT
EFQPLYYVAESFNDAKEKVRNFAATIPRPFSVRYDPYTQRIEVL
>1XKWA
ESTSATQPPGVTTLGKVPLKPRELPQSASVIDHERLEQQNLFSLDEAMQQATGVTVQPFQLLTTAY
YVRGFKVDSFELDGVPALLGNTASSPQDMAIYERVEILRGSNGLLHGTGNPAATVNLVRKRPQREF
AASTTLSAGRWDRYRAEVDVGGPLSASGNVRGRAVAAYEDRDYFYDVADQGTRLLYGVTEFDLSPD
TLLTVGAQYQHIDSITNMAGVPMAKDGSNLGLSRDTYLDVDWDRFKWDTYRAFGSLEQQLGGGWKG
KVSAEYQEADSRLRYAGSFGAIDPQTGDGGQLMGAAYKFKSIQRSLDANLNGPVRLFGLTHELLGG
VTYAQGETRQDTARFLNLPNTPVNVYRWDPHGVPRPQIGQYTSPGTTTTTQKGLYALGRIKLAEPL
TLVVGGRESWWDQDTPATRFKPGRQFTPYGGLIWDFARDWSWYVSYAEVYQPQADRQTWNSEPLSP
VEGKTYETGIKGELADGRLNLSLAAFRIDLENNPQEDPDHPGPPNNPFYISGGKVRSQGFELEGTG
YLTPYWSLSAGYTYTSTEYLKDSQNDSGTRYSTFTPRHLLRLWSNYDLPWQDRRWSVGGGLQAQSD
YSVDYRGVSMRQGGYALVNMRLGYKIDEHWTAAVNVNNLFDRTYYQSLSNPNWNNRYGEPRSFNVS
LRGAF
>2EX4A
MGSSHHHHHHSSGLVPRGSTSEVIEDEKQFYSKAKTYWKQIPPTVDGMLGGYGHISSIDINSSRKF
LQRFLREGPNKTGTSCALDCGAGIGRITKRLLLPLFREVDMVDITEDFLVQAKTYLGEEGKRVRNY
FCCGLQDFTPEPDSYDVIWIQWVIGHLTDQHLAEFLRRCKGSLRPNGIIVIKDNMAQEGVILDDVD
SSVCRDLDVVRRIICSAGLSLLAEERQENLPDEIYHVYSFALR
>1ZDQA
ATAAEIAALPRQKVELVDPPFVHAHSQVAEGGPKVVEFTMVIEEKKIVIDDAGTEVHAMAFNGTVP
GPLMVVHQDDYLELTLINPETNTLMHNIDFHAATGALGGGGLTEINPGEKTILRFKATKPGVFVYH
CAPPGMVPWHVVSGGNGAIMVLPREGLHDGKGKALTYDKIYYVGEQDFYVPRDENGKYKKYEAPGD
AYEDTVKVMRTLTPTHVVFNGAVGALTGDKAMTAAVGEKVLIVHSQANRDTRPHLIGGHGDYVWAT
GKFNTPPDVDQETWFIPGGAAGAAFYTFQQPGIYAYVNHNLIEAFELGAAAHFKVTGEWNDDLMTS
VLAPSG
>3ME7A
MSLGTYVPGDITLVDSYGNEFQLKNLKGKPIILSPIYTHCRAACPLITKSLLKVIPKLGTPGKDFW
VITFTFDPKDTLEDIKRFQKEYGIDGKGWKVVKAKTSEDLFKLLDAIDFRFMTAGNDFIHPNVVVV
LSPELQIKDYIYGVNYNYLEFVNALRLARGEGHHHHHH
>2NT3A
GSHMSKKILIVESDTALSATLRSALEGRGFTVDETTDGKGSVEQIRRDRPDLVVLAVDLSAGQNGY

LICGKLKKDDDLKNVPIVIIGNPDGFAQHRKLKAHADEAVAKPVDADQLVERAGALIGFPE
>1RFXA
SSMPLCPIDEAIDKKIKQDFNSLFPNAIKNIGLNCWTVSSRGKLASCPEGTAVLSCSCGSACGSWD
IREEKVCHCQCARIDWTAARCCKLQVAS
>1C7JA
MTHQIVTTQYGKVKGTTENGVHKWKGIPYAKPPVGQWRFKAPEPPEVWEDVLDATAYGPVCPQPSD
LLSLSYTELPRQSEDCLYVNVFAPDTPSQNLPVMVWIHGGAFYLGAGSEPLYDGSKLAAQGEVIVV
TLNYRLGPFGFMHLSSFDEAYSDNLGLLDQAAALKWVRENISAFGGDPDNVTVFGESAGGMSIAAL
LAMPAAKGLFQKAIMESGASRTMTKEQAASTAAAFLQVLGINESQLDRLHTVAAEDLLKAADQLRI
AEKENIFQLFFQPALDPKTLPEEPEKSIAEGAASGIPLLIGTTRDEGYLFFTSDSDVRSQETLDAA
LEYSLGKPLAEKAADLYPRSLESQIHMVTDLLFWRPAVAFASAQSHYAPVWMYRFDWHPEKPPYNK
AFHALELPFVFGNLDGLERMAKAEITDEVKQLSHTIQSAWITFAKTGNPSTEAVNWPAYHEETRET
VILDSEITIENDPESEKRQKLFPSKGE
>1H0HB
SKGFFVDTTRCTACRGCQVACKQWHGNPATPTENTGFHQNPPDFNFHTYKLVRMHEQEIDGRIDWL
FFPDQCRHCIAPPCKATADMEDESAIIHDDATGCVLFTPKTKDLEDYESVISACPYDVPRKVAESN
QMAKCDMCIDRITNGLRPACVTSCPTGAMNFGDLSEMEAMASARLAEIKAAYSDAKLCDPDDVRVI
FLTAHNPKLYHEYAVA
>1ZE3D
DLYFNPRFLADDPQAVADLSRFENGQELPPGTYRVDIYLNNGYMATRDVTFNTGDSEQGIVPCLTR
AQLASMGLNTASVAGMNLLADDACVPLTTMVQDATAHLDVGQQRLNLTIPQAFMSNRAR
>1UHAA
APECGERASGKRCPNGKCCSQWGYCGTTDNYCGQGCQSQCDYWRCGRDFGGRLCEEDMCCSKYGWC
GYSDDHCEDGCQSQCD
>1J8MF
SKLLDNLRDTVRKFLTGSSSYDKAVEDFIKELQKSLISADVNVKLVFSLTNKIKERLKNEKPPTYI
ERREWFIKIVYDELSNLFGGDKEPKVIPDKIPYVIMLVGVQGTGKTTTAGKLAYFYKKKGFKVGLV
GADVYRPAALEQLQQLGQQIGVPVYGEPGEKDVVGIAKRGVEKFLSEKMEIIIVDTAGRHGYGEEA
ALLEEMKNIYEAIKPDEVTLVIDASIGQKAYDLASKFNQASKIGTIIITKMDGTAKGGGALSAVAA
TGATIKFIGTGEKIDELEVFNPRRFVARLHHHH
>3K8GA
GSGAWKASVDPLGVVGSGADVYLYFPVAGNENLISRIIENHESKADIKKIVDRTTAVYGAFFARSK
EFRLFGSGSYPYAFTNLIFSRSDGWASTKTEHGITYYESEHTDVSIPAPHFSCVIFGSSKRERMSK
MLSRLVNPDRPQLPPRFEKECTSEGTSQTVALYIKNGGHFITKLLNFPQLNLPLGAMELYLTARRN
EYLYTLSLQLGNAKINFPIQFLISRVLNAHIHVEGDRLIIEDGTISAERLASVISSLYSKKGSS
>2B1LA
MQFYQADVLTQGKPVLLNVWATWCPTCRAEHQYLNQLSAQGIRVVGMNYKDDRQKAISWLKELGNP
YALSLFDGDGMLGLDLGVYGAPETFLIDGNGIIRYRHAGDLNPRVWEEEIKPLWEKYSKEAAQ
>2ZZ3A
GSHMRSRRVDVMDVMNRLILAMDLMNRDDALRVTGEVREYIDTVKIGYPLVLSEGMDIIAEFRKRF
GCRIIAAFKVADIPETNEKICRATFKAGADAIIVHGFPGADSVRACLNVAEEMGREVFLLTEMSHP
GAEMFIQGAADEIARMGVDLGVKNYVGPSTRPERLSRLREIIGQDSFLISPGVGAQGGDPGETLRF
ADAIIVGRSIYLADNPAAAAAGIIESIKDLRIPEDPAANKARKEAELAAATAEQ
>3GY1A

MSLEPTIITDVLCYITKPDRHNLVVVKVETNKGIYGLGCATFQQRPKAVSLVVSEYLKPILIGRDA
NNIEDLWQMMMVNSYWRNGPILNNAISGVDMALWDIKGKLANMPLYQLFGGKSRDAIAAYTHAVAD
NLEDLYTEIDEIRKKGYQHIRCQLGFYGGNSSEFHTTDNPTQGSYFDQDEYMRTTVSMFSSLREKY
GYKFHILHDVHERLFPNQAVQFAKDVEKYKPYFIEDILPPDQNEWLGQIRSQTSTPLATGELFNNP
MEWKSLIANRQVDFIRCHVSQIGGITPALKLGSLCAAFGVRIAWHTPSDITPIGVAVNIHLNINLH
NAAIQENIEINDNTRCVFSGIPEAKNGFFYPIESPGIGVDIDENEIIKYPVEYRPHEWTQSRIPDG
TIVTEGHHHHHH
>1XMZA
MRGSHHHHHHGSASFLKKTMPFKTTIEGTVNGHYFKCTGKGEGNPFEGTQEMKIEVIEGGPLPFAF
HILSTSCXSKTFIKYVSGIPDYFKQSFPEGFTWERTTTYEDGGFLTAHQDTSLDGDCLVYKVKILG
NNFPADGPVMQNKAGRWEPGTEIVYEVDGVLRGQSLMALKCPGGRHLTCHLHTTYRSKKPASALKM
PGFHFEDHRIEIMEEVEKGKCYKQYEAAVGRYCDAAPSKLGHN
>2P35A
QGHMAWSAQQYLKFEDERTRPARDLLAQVPLERVLNGYDLGCGPGNSTELLTDRYGVNVITGIDSD
DDMLEKAADRLPNTNFGKADLATWKPAQKADLLYANAVFQWVPDHLAVLSQLMDQLESGGVLAVQM
PDNLQEPTHIAMHETADGGPWKDAFSGGGLRRKPLPPPSDYFNALSPKSSRVDVWHTVYNHPMKDA
DSIVEWVKGTGLRPYLAAAGEENREAFLADYTRRIAAAYPPMADGRLLLRFPRLFVVAVKK
>2P3JA
VLDGPYQPTTFNPPVDYWMLLAPTAAGVVVEGTNNTDAWLATILVEPNVTSETRSYTLFGTQEQIT
IANASQTQWKFIDVVKTTQNGSYSQYGPLQSTPKLYAVMKHNGKIYTYNGETPNVTTKYYSTTNYD
SVNMTAFCDFYIIPREEESTCTEYINNGL
>2OFXA
QGHMATNVTYQAHHVSRNKRGQVVGTRGGFRGCTVWLTGLSGAGKTTVSMALEEYLVCHGIPCYTL
DGDNIRQGLNKNLGFSPEDREENVRRIAEVAKLFADAGLVCITSFISPYTQDRNNARQIHEGASLP
FFEVFVDAPLHVCEQRDVKGLYKKARAGEIKGFTGIDSEYEKPEAPELVLKTDSCDVNDCVQQVVE
LLQERDIVP
>2QSIA
GHMSGSLARAAARPNAPTLVDEATVDDFIAHSGKIVVLFFRGDAVRFPEAADLAVVLPELINAFPG
RLVAAEVAAEAERGLMARFGVAVCPSLAVVQPERTLGVIAKIQDWSSYLAQIGAMLAEVDQPGEAE
LQSGS
>2J9FA
SSLDDKPQFPGASAEFIDKLEFIQPNVISGIPIYRVMDRQGQIINPSEDPHLPKEKVLKLYKSMTL
LNTMDRILYESQRQGRISFYMTNYGEEGTHVGSAAALDNTDLVFGQYREAGVLMYRDYPLELFMAQ
CYGNISDLGKGRQMPVHYGCKERHFVTISSPLATQIPQAVGAAYAAKRANANRVVICYFGEGAASE
GDAHAGFNFAATLECPIIFFCRNNGYAISTPTSEQYRGDGIAARGPGYGIMSIRVDGNDVFAVYNA
TKEARRRAVAENQPFLIEAMTYRIGHHSTSDDSSAYRPVDEVNYWDKQDHPISRLRHYLLSQGWWD
EEQEKAWRKQSRRKVMEAFEQAERKPKPNPNLLFSDVYQEMPAQLRKQQESLARHLQTYGEHYPLD
HFDK
>3BB0A
MGSVTPIPLPKIDEPEEYNTNYILFWNHVGLELNRVTHTVGGPLTGPPLSARALGMLHLAIHDAYF
SICPPTDFTTFLSPDTENAAYRLPSPNGANDARQAVAGAALKMLSSLYMKPVEQPNPNPGANISDN
AYAQLGLVLDRSVLEAPGGVDRESASFMFGEDVADVFFALLNDPRGASQEGYHPTPGRYKFDDEPT
HPVVLIPVDPNNPNGPKMPFRQYHAPFYGKTTKRFATQSEHFLADPPGLRSNADETAEYDDAVRVA
IAMGGAQALNSTKRSPWQTAQGLYWAYDGSNLIGTPPRFYNQIVRRIAVTYKKEEDLANSEVNNAD

FARLFALVDVACTDAGIFSWKEKWEFEFWRPLSGVRDDGRPDHGDPFWLTLGAPATNTNDIPFKPP
FPAYPSGHATFGGAVFQMVRRYYNGRVGTWKDDEPDNIAIDMMISEELNGVNRDLRQPYDPTAPIE
DQPGIVRTRIVRHFDSAWELMFENAISRIFLGVHWRFDAAAARDILIPTTTKDVYAVDNNGATVFQ
NVEDIRYTTRGTREDREGLFPIGGVPLGIEIADEIFNNGLKPTPPEIQPMPQETPVQKPVGQQPVK
GMWEEEQAPVVKEAP
>3H9CA
TQVAKKILVTCALPYANGSIHLGHMLEHIQADVWVRYQRMRGHEVNFICADDAHGTPIMLKAQQLG
ITPEQMIGEMSQEHQTDFAGFNISYDNYHSTHSEENRQLSELIYSRLKENGFIKNRTISQLYDPEK
GMFLPDRFVKGTCPKCKSPDQYGDNCEVCGATYSPTELIEPKSVVSGATPVMRDSEHFFFDLPSFS
EMLQAWTRSGALQEQVANKMQEWFESGLQQWDISRDAPYFGFEIPNAPGKYFYVWLDAPIGYMGSF
KNLCDKRGDSVSFDEYWKKDSTAELYHFIGKDIVYFHSLFWPAMLEGSNFRKPSNLFVHGYVTVNG
AKMSKSRGTFIKASTWLNHFDADSLRYYYTAKLSSRIDDIDLNLEDFVQRVNADIVNKVVNLASRN
AGFINKRFDGVLASELADPQLYKTFTDAAEVIGEAWESREFGKAVREIMALADLANRYVDEQAPWV
VAKQEGRDADLQAICSMGINLFRVLMTYLKPVLPKLTERAEAFLNTELTWDGIQQPLLGHKVNPFK
ALYNRIDMRQVEALVEASK
>2FHLA
ARKCSLTGKWTNNLGSIMTIRAVNSRGEFTGTYLTAVADNPGNITLSPLLGIQHKRASQPTFGFTV
HWNFSESTTVFTGQCFIDRNGKEVLKTMWLLRSSVNDISYDWKATRVGYNNFTRLS
>2PRVA
GMIYSKVENFINENKQNAIFTEGASHENIGRIEENLQCDLPNSYKWFLEKYGAGGLFGVLVLGYNF
DHASVVNRTNEYKEHYGLTDGLVVIEDVDYFAYCLDTNKMKDGECPVVEWDRVIGYQDTVADSFIE
FFYNKIQEAKDDWDEDEDWDD
>1JLJA
MATEGMILTNHDHQIRVGVLTVSDSCFRNLAEDRSGINLKDLVQDPSLLGGTISAYKIVPDEIEEI
KETLIDWCDEKELNLILTTGGTGFAPRDVTPEATKEVIEREAPGMALAMLMGSLNVTPLGMLSRPV
CGIRGKTLIINLPGSKKGSQECFQFILPALPHAIDLLRDAIVKVKEVHDRSHHHHHH
>1XFIA
SESDSEMVPFPQLPMPIENNYRACTIPYRFPSDDPKKATPNEISWINVFANSIPSFKKRAESDITV
PDAPARAEKFAERYAGILEDLKKDPESHGGPPDGILLCRLREQVLRELGFRDIFKKVKDEENAKAI
SLFPQVVSLSDAIEDDGKRLENLVRGIFAGNIFDLGSAQLAEVFSRDGMSFLASCQNLVPRPWVID
DLENFQAKWINKSWKKAVIFVDNSGADIILGILPFARELLRRGAQVVLAANELPSINDITCTELTE
ILSQLKDENGQLLGVDTSKLLIANSGNDLPVIDLSRVSQELAYLSSDADLVIVEGMGRGIETNLYA
QFKCDSLKIGMVKHLEVAEFLGGRLYDCVFKFNEVQS
>1KZ1A
MFSGIKGPNPSDLKGPELRILIVHARGNLQAIEPLVKGAVETMIEKHDVKLENIDIESVPGSWELP
QGIRASIARNTYDAVIGIGVLIKGSTMHFEYISEAVVHGLMRVGLDSGVPVILGLLTVLNEEQALY
RAGLNGGHNHGNDWGSAAVEMGLKALY
>2WL5A
STPSIVIASAARTAVGSFNGAFANTPAHELGATVISAVLERAGVAAGEVNEVILGQVLPAGEGQNP
ARQAAMKAGVPQEATAWGMNQLCGSGLRAVALGMQQIATGDASIIVAGGMESMSMAPHCAHLRGGV
KMGDFKMIDTMIKDGLTDAFYGYHMGTTAENVAKQWQLSRDEQDAFAVASQNKAEAAQKDGRFKDE
IVPFIVKGRKGDITVDADEYIRHGATLDSMAKLRPAFDKEGTVTAGNASGLNDGAAAALLMSEAEA
SRRGIQPLGRIVSWATVGVDPKVMGTGPIPASRKALERAGWKIGDLDLVEANEAFAAQACAVNKDL
GWDPSIVNVNGGAIAIGNPIGASGARILNTLLFEMKRRGARKGLATLCIGGGMGVAMCIESL

>1E2XA

FADRMVIKAQSPAGFAEEYIIESIWNNRFPPGTILPAERELSELIGVTRTTLREVLQRLARDGWLT
IQHGKPTKVNNFWETSGLNILETLARLDHESVPQLIDNLLSVRTNISTIFIRTAFRQHPDKAQEVL
ATANEVADHADAFAELDYNIFRGLAFASGNPIYGLILNGMKGLYTRIGRHYFANPEARSLALGFYH
KLSALCSEGAHDQVYETVRRYGHESGEIWHRMQKNLPGDLAIQGR

>2O7IA

MQVSLPREDTVYIGGALWGPATTWNLYAPQSTWGTDQFMYLPAFQYDLGRDAWIPVIAERYEFVDD
KTLRIYIRPEARWSDGVPITADDFVYALELTKELGIGPGGGWDTYIEYVKAVDTKVVEFKAKEENL
NYFQFLSYSLGAQPMPKHVYERIRAQMNIKDWINDKPEEQVVSGPYKLYYYDPNIVVYQRVDDWWG
KDIFGLPRPKYLAHVIYKDNPSASLAFERGDIDWNGLFIPSVWELWEKKGLPVGTWYKKEPYFIPD
GVGFVYVNNTKPGLSDPAVRKAIAYAIPYNEMLKKAYFGYGSQAHPSMVIDLFEPYKQYIDYELAK
KTFGTEDGRIPFDLDMANKILDEAGYKKGPDGVRVGPDGTKLGPYTISVPYGWTDWMMMCEMIAKN
LRSIGIDVKTEFPDFSVWADRMTKGTFDLIISWSVGPSFDHPFNIYRFVLDKRLSKPVGEVTWAGD
WERYDNDEVVELLDKAVSTLDPEVRKQAYFRIQQIIYRDMPSIPAFYTAHWYEYSTKYWINWPSED
NPAWFRPSPWHADAWPTLFIISKKSDPQPVPSWLGTVDEGGIEIPTAKIFEDLQKATMHHHHHH

>1YU0A

MSTAVQFRGGTTAQHATFTGAAREITVDTDKNTVVVHDGATAGGFPLARHDLVKTAFIKADKSAVA
FTRTGNATASIKAGTIVEVNGKLVQFTADTAITMPALTAGTDYAIYVCDDGTVRADSNFSAPTGYT
STTARKVGGFHYAPGSNAAAQAGGNTTAQINEYSLWDIKFRPAALDPRGMTLVAGAFWADIYLLGV
NHLTDGTSKYNVTIADGSASPKKSTKFGGDGSAAYSDGAWYNFAEVMTHHGKRLPNYNEFQALAFG
TTEATSSGGTDVPTTGVNGTGATSAWNIFTSKWGVVQASGCLWTWGNEFGGVNGASEYTANTGGRG
SVYAQPAAALFGGAWNGTSLSGSRAALWYSGPSFSFAFFGARGVCDHLILE

>1YUKA

QECTKFKVSSCRECIESGPGCTWCQKLNFTGPGDPDSIRCDTRPQLLMRGCAADDIMDPTSLAETQ
EDHNGGQKQLSPQKVTLYLRPGQAAAFNVTFRRAKGY

>2QNDA

ASRFHEQFIVREDLMGLAIGTHGANIQQARKVPGVTAIDLDEDTCTFHIYGEDQDAVKKARSFLEF
AEDVIQVPRNLVGKVIGKNGKLIQEIVDKSGVVRVRIEAENEKNVPQEEGMVPFVFVGTKDSIANA
TVLLDYHLNYLK

>3EOFA

GMMDTVKNRRTIRKYQQKDITPDLLNDLLETSFRASTMGGMQLYSVVVTRDAEKKEILSPAHFNQP
MVKEAPVVLTFCADFRRFCKYCQERNAVPGYGNLMSFLNAAMDTLLVAQTFCTLAEEAGLGICYLG
TTTYNPQMIIDALHLPELVFPITTVTVGYPAESPKQVDRLPIEGIIHEESYHDYTAEDINRLYAYK
ESLPENKLFIEENQKETLPQVFTDVRYTKKDNEFMSENLLKVLRRQGFMD

>2GRRA

GSHMSGIALSRLAQERKAWRKDHPFGFVAVPTKNPDGTMNLMNWECAIPGKKGTPWEGGLFKLRML
FKDDYPSSPPKCKFEPPLFHPNVYPSGTVCLSILEEDKDWRPAITIKQILLGIQELLNEPNIQSPA
QAEAYTIYCQNRVEYEKRVRAQAKKFAPS

>3EJVA

MGSDKIHHHHHHENLYFQGMTMADETIILNVLGQYTRAHDRRDPDAMAALFAPEATIEIVDAVGGA
SRSISRLEGRDAIRVAVRQMMAPHGYRAWSQNVVNAPIIVIEGDHAVLDAQFMVFSILAAEVPDGG
WPTGTFGAQGRIVPIEAGQYRLTLRTVADGWVISAMRIEHRLPMAFG

>3GWBA

ELDGKAPSHRNLNVQTWSTAEGAKVLFVEARELPMFDLRLIFAAGSSQDGNAPGVALLTNAMLNEG

VAGKDVGAIAQGFEGLGADFGNGAYKDMAVASLRSLSAVDKREPALKLFAEVVGKPTFPADSLARI
KNQMLAGFEYQKQNPGKLASLELMKRLYGTHPYAHASDGDAKSIPPITLAQLKAFHAKAYAAGNVV
IALVGDLSRSDAEAIAAQVSAALPKGPALAKIEQPAEPKASIGHIEFPSSQTSLMLAQLGIDRDDP
DYAAVSLGNQILGGGGFGTRLMSEVREKRGLTYGVYSGFTPMQARGPFMINLQTRAEMSEGTLKLV
QDVFAEYLKNGPTQKELDDAKRELAGSFPLSTASNADIVGQLGAMGFYNLPLSYLEDFMRQSQELT
VEQVKAAMNKHLNVDKMVIVSAGPTVAQKPLEHHHHHH
>1GWTA
MQLTPTFYDNSCPNVSNIVRDTIVNELRSDPRIAASILRLHFHDCFVNGCDASILLDNTTSFRTEK
DAFGNANSARGFPVIDRMKAAVESACPRTVSCADLLTIAAQQSVTLAGGPSWRVPLGRRDSLQAFL
DLANANLPAPFFTLPQLKDSFRNVGLNRSSDLVALSGGHTFGKNQCRFIMDRLYNFSNTGLPDPTL
NTTYLQTLRGLCPLNGNLSALVDMDLRTPTIFDNKYYVNLEEQKGLIQSDQELFSSPNATDTIPLV
RSFANSTQTFFNAFVEAMDRMGNITPLTGTQGQIRLNCRVVNSNS
>1E39A
ADNLAEFHVQNQECDSCHTPDGELSNDSLTYENTQCVSCHGTLAEVAETTKHEHYNAHASHFPGEV
ACTSCHSAHEKSMVYCDSCHSFDFNMPYAKKWLRDEPTIAELAKDKSERQAALASAPHDTVDVVVV
GSGGAGFSAAISATDSGAKVILIEKEPVIGGNAKLAAGGMNAAWTDQQKAKKITDSPELMFEDTMK
GGQNINDPALVKVLSSHSKDSVDWMTAMGADLTDVGMMGGASVNRAHRPTGGAGVGAHVVQVLYDN
AVKRNIDLRMNTRGIEVLKDDKGTVKGILVKGMYKGYYWVKADAVILATGGFAKNNERVAKLDPSL
KGFISTNQPGAVGDGLDVAENAGGALKDMQYIQAAPTLSVKGGVMVTEAVRGNGAILVNREGKRFV
NEITTRDKASAAILAQTGKSAYLIFDDSVRKSLSKIDKYIGLGVAPTADSLVKLGKMEGIDGKALT
ETVARYNSLVSSGKDTDFERPNLPRALNEGNYYAIEVTPGVHHTMGGVMIDTKAEVMNAKKQVIPG
LYGAGEVTGGVHGANRLGGNAISDIITFGRLAGEEAAKYSKKN
>3E3UA
MAVVPIRIVGDPVLHTATTPVTVAADGSLPADLAQLIATMYDTMDAANGVGLAANQIGCSLRLFVY
DCAADRAMTARRRGVVINPVLETSEIPETMPDPDTDDEGCLSVPGESFPTGRAKWARVTGLDADGS
PVSIEGTGLFARMLQHETGHLDGFLYLDRLIGRYARNAKRAVKSHGWGVPGLSWLPGEDPDPFGH
>1E30A
GTLDTTWKEATLPQVKAMLEKDTGKVSGDTVTYSGKTVHVVAAAVLPGFPFPSFEVHDKKNPTLEI
PAGATVDVTFINTNKGFGHSFDITKKGPPYAVMPVIDPIVAGTGFSPVPKDGKFGYTNFTWHPTAG
TYYYVCQIPGHAATGQFGKIVVK
>3K2CA
MAHHHHHHMGTLEAQTQGPGSMAKEASGNVYFDVYANEESLGRIVMKLEDDIVPKTAKNFRTLCER
PKGEGYKGSTFHRIIPGFMVQGGDYTAHNGTGGRSIYGEKFPDENFELKHTKEGILSMANCGAHTN
GSQFFITLGKTQWLDEKHVVFGEVVEGMDVVHKIAKYGSESGQVKKGYRIEIRDCGVLGSN
>2R1BA
GSSLRGGHAGTTYIFSKGGGQITYKWPPNDRPSTRADRLAIGFSTVQKEAVLVRVDSSSGLGDYLE
LHIHQGKIGVKFNVGTDDIAIEESNAIINDGKYHVVRFTRSGGNATLQVDSWPVIERYPAGNNDNE
RLAIARQRIPYRLGRVVDEWLLDKGRQLTIFNSQATIIIGGKEQGQPFQGQLSGLYYNGLKVLNMA
AENDANIAIVGNVRLVGEVPSS
>1I9YA
YDPIHEYVNHELRKRENEFSEHKNVKIFVASYNLNGCSATTKLENWLFPENTPLADIYVVGFQEIV
QLTPQQVISADPAKRREWESCVKRLLNGKCTSGPGYVQLRSGQLVGTALMIFCKESCLPSIKNVEG
TVKKTGLGGVSGNKGAVAIRFDYEDTGLCFITSHLAAGYTNYDERDHDYRTIASGLRFRRGRSIFN
HDYVVWFGDFNYRISLTYEEVVPCIAQGKLSYLFEYDQLNKQMLTGKVFPFFSELPITFPPTYKFD

IGTDIYDTSDKHRVPAWTDRILYRGELVPHSYQSVPLYYSDHRPIYATYEANIVKVDREKKKILFE
ELYNQRKQEVRDASQTS
>1OAIA
PTLSPEQQEMLQAFSTQSGMNLEWSQKCLQDNNWDYTRSAQAFTHLKAKGEIPEVAFMK
>3C1JA
APAVADKADNAFMMICTALVLFMTIPGIALFYGGLIRGKNVLSMLTQVTVTFALVCILWVVYGYSL
AFGEGNNFFGNINWLMLKNIELTAVMGSIYQYIHVAFQGSAACITVGLIVGALAERIRFSAVLIFV
VVWLTLSYIPIAHMVWGGGLLASHGALDFAGGTVVHINAAIAGLVGAYLIGKRVGFGKEAFKPHNL
PMVFTGTAILYIGWFGANAGSAGTANEIAALAFVNTVVATAAAILGWIFGEWALRGKPSLLGACSG
AIAGLVGVTPACGYIGVGGALIIGVVAGLAGLWGVTMLKRLLRVDDPCDVFGVHGVCGIVGCIMTG
IFAASSLGGVGFAEGVTMGHQLLVQLESIAITIVWSGVVAFIGYKLADLTVGLRVPEEQEREGLDV
NSHGENAYNADQAQQPAQADLEHHHHHH
>2C15A
MSFTPANRAYPYTRLRRNRRDDFSRRLVRENVLTVDDLILPVFVLDGVNQRESIPSMPGVERLSID
QLLIEAEEWVALGIPALALFPVTPVEKKSLDAAEAYNPEGIAQRATRALRERFPELGIITDVALDP
FTTHGQDGILDDDGYVLNDVSIDVLVRQALSHAEAGAQVVAPSDMMDGRIGAIREALESAGHTNVR
VMAYSAKYASAYYGPFRDAVGSASNLGKGNKATYQMDPANSDEALHEVAADLAEGADMVMVXPGMP
YLDIVRRVKDEFRAPTFVYQVSGEYAMHMGAIQNGWLAESVILESLTAFKRAGADGILTYFAKQAA
EQLRRGR
>1QL3A
ADPAAGEKVFGKCKACHKLDGNDGVGPHLNGVVGRTVAGVDGFNYSDPMKAHGGDWTPEALQEFLT
NPKAVVKGTKMAFAGLPKIEDRANLIAYLEGQQ
>1CHMA
QMPKTLRIRNGDKVRSTFSAQEYANRQARLRAHLAAENIDAAIFTSYHNINYYSDFLYCSFGRPYA
LVVTEDDVISISANIDGGQPWRRTVGTDNIVYTDWQRDNYFAAIQQALPKARRIGIEHDHLNLQNR
DKLAARYPDAELVDVAAACMRMRMIKSAEEHVMIRHGARIADIGGAAVVEALGDQVPEYEVALHAT
QAMVRAIADTFEDVELMDTWTWFQSGINTDGAHNPVTTRKVNKGDILSLNCFPMIAGYYTALERTL
FLDHCSDDHLRLWQVNVEVHEAGLKLIKPGARCSDIARELNEIFLKHDVLQYRTFGYGHSFGTLSH
YYGREAGLELREDIDTVLEPGMVVSMEPMIMLPEGLPGAGGYREHDILIVNENGAENITKFPYGPE
KNIIR
>3CZVA
GSMSRLSWGYREHNGPIHWKEFFPIADGDQQSPIEIKTKEVKYDSSLRPLSIKYDPSSAKIISNSG
HSFNVDFDDTENKSVLRGGPLTGSYRLRQVHLHWGSADDHGSEHIVDGVSYAAELHVVHWNSDKYP
SFVEAAHEPDGLAVLGVFLQIGEPNSQLQKITDTLDSIKEKGKQTRFTNFDLLSLLPPSWDYWTYP
GSLTVPPLLESVTWIVLKQPINISSQQLAKFRSLLCTAEGEAAAFLVSNHRPPQPLKGRKVRASFH
>1CZYA
AMADLEQKVLEMEASTYDGVFIWKISDFPRKRQEAVAGRIPAIFSPAFYTSRYGYKMCLRIYLNGD
GTGRGTHLSLFFVVMKGPNDALLRWPFNQKVTLMLLDQNNREHVIDAFRPDVTSSSFQRPVNDMNI
ASGCPLFCPVSKMEAKNSYVRDDAIFIKAIVDLTGL
>2V5IA
MVSVGDAAFRQEANKKFKYSVKLSDYSTLQDAVTDAVDGLLIDINYNFTDGESVDFXGKILTINCK
AKFIGDGALIFNNMGPGSVINQPFMESKTTPWVIFPWDADGKWITDAALVAATLKQSKIEGYQPGV
NDWVKFPGLEALLPQNVKDQHIAATLDIRSASRVEIRNAGGLMAAYLFRSCHHCKVIDSDSIIGGK
DGIITFENLSGDWGLGNYVIGGRVHYGSGSGVQFLRNNGGESHNGGVIGVTSWRAGESGFKTYQGS

VGGGTARNYNLQFRDSVALSPVWDGFDLGSDPGMAPEPDRPGDLPVSEYPFHQLPNNHLVDNILVM
NSLGVGLGMDGSGGYVSNVTVQDCAGAGMLAHTYNRVFSNITVIDCNYLNFDSDQIIIIGDCIVNG
IRAAGIKPQPSNGLVISAPNSTISGLVGNVPPDKILVGNLLDPVLGQSRVIGFNSDTAELALRINK
LSATLDSGALRSHLNGYAGSGSAWTELTALSGSTPNAVSLKVNRGDYKTTEIPISGTVLPDEGVLD
INTMSLYLDAGALWALIRLPDGSKTRMKLSV
>1YCLA
PSVESFELDHNAVVAPYVRHCGVHKVGTDGVVNKFDIRFCQPNKQAMKPDTIHTLEHLLAFTIRSH
AEKYDHFDIIDISPMGAQTGYYLVVSGEPTSAEIVDLLEDTMKEAVEITEIPAANEKQCGQAKLHD
LEGAKRLMRFWLSQDKEELLKVFG
>2G8FA
GSHMAKEEIIWESLSVDVGSQGNPGIVEYKGVDTKTGEVLFEREPIPIGTNNMGEFLAIVHGLRYL
KERNSRKPIYSDSQTAIKWVKDKKAKSTLVRNEETALIWKLVDEAEEWLNTHTYETPILKWQTDKW
GAIKADYGRK
>1G8KB
RTTLAYPATAVSVAKNLAANEPVSFTYPDTSSPCVAVKLGAPVPGGVGPDDDIVAYSVLCTHMGCP
TSYDSSSKTFSCPCHFTEFDAEKAGQMICGEATADLPRVLLRYDAASDALTAVGVDGLIYGRQANV
I
>2NS9A
SLRLHAGVWGLKVRYEGSFEVSKTPEEVFEFLTDPKRFSRAFPGFKSVEVEDGSFTIELRLSLGPL
RGDARVRASFEDLEKPSKATVKGSGRGAGSTLDFTLRFAVEPSGGGSRVSWVFEGNVGGLAASMGG
RVLDSLARRMINDVISGVKRELGEA
>2V9MA
MQNITQSWFVQGMIKATTDAWLKGWDERNGGNLTLRLDDADIAPYHDNFHQQPRYIPLSQPMPLLA
NTPFIVTGSGKFFRNVQLDPMANLGIVKVDSDGAGYHILWGLFNEAVPTSELPAHFLSHCERIKAT
NGKDRVIMHCHATNLIALTYVLENDTAVFTRQLWEGSTECLVVFPDGVGILPWMVPGTDAIGQATA
QEMQKHSLVLWPFHGVFGSGPTLDETFGLIDTAEKSAQVLVKVYSMGGMKQTISREELIALGKRFG
VTPLASALAL
>2QIQA
AGFRKMAFPSGKVEGCMVQVTCGTTTLNGLWLDDTVYCPRHVICTAEDMLNPNYEDLLIRKSNHSF
LVQAGNVQLRVIGHSMQNCLLRLKVDTSNPKTPKYKFVRIQPGQTFSVLACYNGSPSGVYQCAMRP
NHTIKGSFLNGSCGSVGFNIDYDCVSFCYMHHMELPTGVHAGTDLEGKFYGPFVDRQTAQAAGTDT
TITLNVLAWLYAAVINGDRWFLNRFTTTLNDFNLVAMKYNYEPLTQDHVDILGPLSAQTGIAVLDM
CAALKELLQNGMNGRTILGSTILEDEFTPFDVVRQCS
>2Z25A
TAPSQVLKIRRPDDWHLHLRDGDMLKTVVPYTSEIYGRAIVMPNLAPPVTTVEAAVAYRQRILDAV
PAGHDFTPLMTCYLTDSLDPNELERGFNEGVFTAAKLYPANATVNSSHGVTSVDAIMPVLERMEKI
GMPLLVHGEVTHADIDIFDREARFIESVMEPLRQRLTALKVVFEHITTKDAADYVRDGNERLAATI
TPQHLMFNRNHMLVGGVRPHLYCLPILKRNIHQQALRELVASGFNRVFLGTDSAPHARHRKESSCG
CAGCFNAPTALGSYATVFEEMNALQHFEAFCSVNGPQFYGLPVNDTFIELVREEQQVAESIALTDD
TLVPFLAGETVRWSVKQ
>3NZNA
SNAVNLFGQKDRGNHVSGVDRGKVIMYGLSTCVWCKKTKKLLTDLGVDFDYVYVDRLEGKEEEEAV
EEVRRFNPSVSFPTTIINDEKAIVGFKEKEIRESLGF
>1YPQA

RVANCSAPCPQDWIWHGENCYLFSSGSFNWEKSQEKCLSLDAKLLKINSTADLDFIQQAISYSSFP
FWMGLSRRNPSYPWLWEDGSPLMPHLFRVRGAVSQTYPSGTCAYIQRGAVYAENCILAAFSICQKK
ANL
>3ENUA
TIEVPVLTFVPVQVSAELENRGCWVKFFDKKNFQGDSLFLSGPATLPRLIGPFGYDWENKVRSVKV
GPRANLTIFDNHNYRDEDKFLDAGANVANLSKEMGFFDNFRSMVLNCI
>3APAA
GSARSSSYSGEYGSGGGKRFSHSGNQLDGPITALRVRVNTYYIVGLQVRYGKVWSDYVGGRNGDLE
EIFLHPGESVIQVSGKYKWYLKKLVFVTDKGRYLSFGKDSGTSFNAVPLHPNTVLRFISGRSGSLI
DAIGLHWDV
>2Q5XA
GIILTKVGYYTIPSMDDLAKITNEKGECIVSDFTIGRKGYGSIYFEGDVNLTNLNLDDIVHIRRKE
VVVYLDDNQKPPVGEGLNRKAEVTLDGVWPTDKTSRCLIKSPDRLADINYEGRLEAVSRKQGAQFK
EYRPETGSWVFKVSHFAKYGLQD
>2Q66A
KVFGITGPVSTVGATAAENKLNDSLIQELKKEGSFETEQETANRVQVLKILQELAQRFVYEVSKKK
NMSDGMARDAGGKIFTYGSYRLGVHGPGSDIDTLVVVPKHVTREDFFTVFDSLLRERKELDEIAPV
PDAFVPIIKIKFSGISIALICARLDQPQVPLSLTLSDKNLLRNLDEKDLRALNGTRVTDEILELVP
KPNVFRIALRAIKLWAQRRAVYANIFGFPGGVAWAMLVARICQLYPNACSAVILNRFFIILSEWNW
PQPVILKPIEDGPLQVRVWNPKIYAQDRSHRMPVITPAYPSMCATHNITESTKKVILQEFVRGVQI
TNDIFSNKKSWANLFEKNDFFFRYKFYLEITAYTRGSDEQHLKWSGLVESKVRLLVMKLEVLAGIK
IAHPFTKPFESSYCCPTEDDYEMIQDKYGSHKTETALNALKLVTDENKEEESIKDAPKAYLSTMYI
GLDFNIENKKEKVDIHIPCTEFVNLCRSFNEDYGDHKVFNLALRFVKGYDLPDEVFDENEKRP
>1DQEA
SQEVMKNLSLNFGKALDECKKEMTLTDAINEDFYNFWKEGYEIKNRETGCAIMCLSTKLNMLDPEG
NLHHGNAMEFAKKHGADETMAQQLIDIVHGCEKSTPANDDKCIWTLGVATCFKAEIHKLNWAPSMD
VAVGE
>1PC5A
MAFVVTDNCIKCKYTDCVEVCPVDCFYEGPNFLVIHPDECIDCALCEPECGAQAIFSEDEVPEDMQ
EFIQLNAELAEVWPNITEKKDPLPDAEDWDGVKGKLQHLER
>2FW6A
MMSETAPLPSASSALEDKAASAPVVGIIMGSQSDWETMRHADALLTELEIPHETLIVSANRTPDRL
ADYARTAAERGLNVIIAGAGGAAHLPGMCAAWTRLPVLGVPVESRALKGMDSLLSIVQMPGGVPVG
TLAIGASGAKNAALLAASILALYNPALAARLETWRALQTASVPNSPITEDK
>2QQRA
GHMQSITAGQKVISKHKNGRFYQCEVVRLTTETFYEVNFDDGSFSDNLYPEDIVSQDCLQFGPPAE
GEVVQVRWTDGQVYGAKFVASHPIQMYQVEFEDGSQLVVKRDDVYTLDEELP
>1BF6A
SFDPTGYTLAHEHLHIDLSGFKNNVDCRLDQYAFICQEMNDLMTRGVRNVIEMTNRYMGRNAQFML
DVMRETGINVVACTGYYQDAFFPEHVATRSVQELAQEMVDEIEQGIDGTELKAGIIAEIGTSEGKI
TPLEEKVFIAAALAHNQTGRPISTHTSFSTMGLEQLALLQAHGVDLSRVTVGHCDLKDNLDNILKM
IDLGAYVQFDTIGKNSYYPDEKRIAMLHALRDRGLLNRVMLSMDITRRSHLKANGGYGYDYLLTTF
IPQLRQSGFSQADVDVMLRENPSQFFQ
>3AHYA

MHHHHHHMLPKDFQWGFATAAYQIEGAVDQDGRGPSIWDTFCAQPGKIADGSSGVTACDSYNRTAE
DIALLKSLGAKSYRFSISWSRIIPEGGRGDAVNQAGIDHYVKFVDDLLDAGITPFITLFHWDLPEG
LHQRYGGLLNRTEFPLDFENYARVMFRALPKVRNWITFNEPLCSAIPGYGSGTFAPGRQSTSEPWT
VGHNILVAHGRAVKAYRDDFKPASGDGQIGIVLNGDFTYPWDAADPADKEAAERRLEFFTAWFADP
IYLGDYPASMRKQLGDRLPTFTPEERALVHGSNDFYGMNHYTSNYIRHRSSPASADDTVGNVDVLF
TNKQGNCIGPETQSPWLRPCAAGFRDFLVWISKRYGYPPIYVTENGTSIKGESDLPKEKILEDDFR
VKYYNEYIRAMVTAVELDGVNVKGYFAWSLMDNFEWADGYVTRFGVTYVDYENGQKRFPKKSAKSL
KPLFDELIAAA
>2AHEA
MALSMPLNGLKEEDKEPLIELFVKAGSDGESIGNCPFSQRLFMILWLKGVVFSVTTVDLKRKPADL
QNLAPGTHPPFITFNSEVKTDVNKIEEFLEEVLCPPKYLKLSPKHPESNTAGMDIFAKFSAYIKNS
RPEANEALERGLLKTLQKLDEYLNSPLPDEIDENSMEDIKFSTRKFLDGNEMTLADCNLLPKLHIV
KVVAKKYRNFDIPKEMTGIWRYLTNAYSRDEFTNTCPSDKEVEIAYSDVAKRLPSKVPKGEFQHTG
GRY
>1OX3A
ADIVLNDLPFVDGPPAEGQSRISWIKNGEEILGADTQYGSEGSMNRPTVSVLRNVEVLDKNIGILK
TSLETANSDIKTIQEAGYIPEAPRDGQAYVRKDGEWVLLSTFL
>1QZ0A
MRERPHTSGHHGAGEARATAPSTVSPYGPEARAELSSRLTTLRNTLAPATNDPRYLQACGGEKLNR
FRDIQCRRQTAVRADLNANYIQVGNTRTIACQYPLQSQLESHFRMLAENRTPVLAVLASSSEIANQ
RFGMPDYFRQSGTYGSITVESKMTQQVGLGDGIMADMYTLTIREAGQKTISVPVVHVGNWPDQTAV
SSEVTKALASLVDQTAETKRNMYESKGSSAVADDSKLRPVIHCRAGVGRTAQLIGAMCMNDSRNSQ
LSVEDMVSQMRVQRNGIMVQKDEQLDVLIKLAEGQGRPLLNS
>1LT4A
NGDRLYRADSRPPDEIKRSGGLMPRGHNEYFDRGTQMNINLYDHARGTQTGFVRYDDGYVSTKLSL
RSAHLAGQSILSGYSTYYIYVIATAPNMFNVNDVLGVYSPHPYEQEVSALGGIPYSQIYGWYRVNF
GVIDERLHRNREYRDRYYRNLNIAPAEDGYRLAGFPPDHQAWREEPWIHHAPQGCGNSSNSSRTIT
RTITGDTCNEETQNLSTIYLREYQSKVKRQIFSDYQSEVDIYNRIRDEL
>1OCBA
YNGNPFEGVQLWANNYYRSEVHTLAIPQITDPALRAAASAVAEVPSFQWLDRNVTVDTLLVQTLSE
IREANQAGANPQYAAQIVVYDLPDRDCAAAASNGEWAIANNGVNNYKAYINRIREILISFSDVRTI
LVIEPDSLANMVTNMNVPKCSGAASTYRELTIYALKQLDLPHVAMYMDAGHAGWLGWPANIQPAAE
LFAKIYEDAGKPRAVRGLATNVANYNAWSVSSPPPYTSPNPNYDEKHYIEAFRPLLEARGFPAQFI
VDQGRSGKQPTGQKEWGHWCNAIGTGFGMRPTANTGHQYVDAFVWVKPGGECDGTSDTTAARYDYH
CGLEDALKPAPEAGQWFNEYFIQLLRNANPPF
>3OCCA
MATPHINAEMGDFADVVLMPGDPLRAKFIAETFLQDVREVNNVRGMLGFTGTYKGRKISVMGHGMG
IPSCSIYAKELITDFGVKKIIRVGSCGAVRTDVKLRDVVIGMGACTDSKVNRMRFKDHDYAAIADF
EMTRNAVDAAKAKGVNVRVGNLFSADLFYTPDPQMFDVMEKYGILGVEMEAAGIYGVAAEFGAKAL
TICTVSDHIRTGEQTTAAERQTTFNDMIEIALESVLLGDNA
>1ZCJA
ASGQAKALQYAFFAEKSANKWSTPSGASWKTASAQPVSSVGVLGLGTMGRGIAISFARVGISVVAV
ESDPKQLDAAKKIITFTLEKEASRAHQNGQASAKPKLRFSSSTKELSTVDLVVEAVFEDMNLKKKV
FAELSALCKPGAFLCTNTSALNVDDIASSTDRPQLVIGTHFFSPAHVMRLLEVIPSRYSSPTTIAT

VMSLSKKIGKIGVVVGNCYGFVGNRMLAPYYNQGFFLLEEGSKPEDVDGVLEEFGFKMGPFRVSDL
AGLDVGWKIRKGQGLTGPSLPPGTPVRKRGNSRYSPLGDMLCEAGRFGQKTGKGWYQYDKPLGRIH
KPDPWLSTFLSQYREVHHIEQRTISKEEILERCLYSLINEAFRILEEGMAARPEHIDVIYLHGYGW
PRHKGGPMFYAASVGLPTVLEKLQKYYRQNPDIPQLEPSDYLRRLVAQGSPPLKEWQSLAGPHGSK
L

>1LVMA

GHHHHHHHGESLFKGPRDYNPISSTICHLTNESDGHTTSLYGIGFGPFIITNKHLFRRNNGTLLVQ
SLHGVFKVKNTTTLQQHLIDGRDMIIIRMPKDFPPFPQKLKFREPQREERICLVTTNFQTKSMSSM
VSDTSCTFPSSDGIFWKHWIQTKDGQCGSPLVSTRDGFIVGIHSASNFTNTNNYFTSVPKNFMELL
TNQEAQQWVSGWRLNADSVLWGGHKVFMDKP

>1E0WA

AESTLGAAAAQSGRYFGTAIASGRLSDSTYTSIAGREFNMVTAENEMKIDATEPQRGQFNFSSADR
VYNWAVQNGKQVRGHTLAWHSQQPGWMQSLSGSALRQAMIDHINGVMAHYKGKIVQWDVVNEAFAD
GSSGARRDSNLQRSGNDWIEVAFRTARAADPSAKLCYNDYNVENWTWAKTQAMYNMVRDFKQRGVP
IDCVGFQSHFNSGSPYNSNFRTTLQNFAALGVDVAITELDIQGAPASTYANVTNDCLAVSRCLGIT
VWGVRDSDSWRSEQTPLLFNNDGSKKAAYTAVLDALNGGDSSEPPADGG

>3EMHA

GPLGSPEFQSKPTPVKPNYALKFTLAGHTKAVSSVKFSPNGEWLASSSADKLIKIWGAYDGKFEKT
ISGHKLGISDVAWSSDSNLLVSASDDKTLKIWDVSSGKCLKTLKGHSNYVFCCNFNPQSNLIVSGS
FDESVRIWDVKTGKCLKTLPAHSDPVSAVHFNRDGSLIVSSSYDGLCRIWDTASGQCLKTLIDDDN
PPVSFVKFSPNGKYILAATLDNTLKLWDYSKGKCLKTYTGHKNEKYCIFANFSVTGGKWIVSGSED
NLVYIWNLQTKEIVQKLQGHTDVVISTACHPTENIIASAALENDKTIKLWKSDC

>3G5JA

SNAMSVIKIEKALKLDKVIFVDVRTEGEYEEDHILNAINMPLFKNNEHNEVGTIYKMQGKHEAIQK
GFDYVSYKLKDIYLQAAELALNYDNIVIYCARGGMRSGSIVNLLSSLGVNVYQLEGGYKAYRNFVL
EY

>3B7AA

MTMEQFLTSLDMIRSGCAPKFKLKTEDLDRLRVGDFNFPPSQDLMCYTKCVALMAGTVNKKGEFNA
PKALAQLPHLVPPEMMEMSRKSVEACRDTHKQFKESCERVYQTAKCFSENADGQFMWP

>3B7CA

GMPTDDIVQLLKGQEEAWNRGDLDAYMQGYWQNEQLMLISNGKFRNGWDETLAAYKKNYPDKESLG
ELKFTIKEIKMLSNYAAMVVGRWDLKRLKDTPTGVFTLLVEKIDDRWVITMDHSSD

>2AD6A

DADLDKQVNTAGAWPIATGGYYSQHNSPLAQINKSNVKNVKAAWSFSTGVLNGHEGAPLVIGDMMY
VHSAFPNNTYALNLNDPGKIVWQHKPKQDASTKAVMCCDVVDRGLAYGAGQIVKKQANGHLLALDA
KTGKINWEVEVCDPKVGSTLTQAPFVAKDTVLMGCSGAELGVRGAVNAFDLKTGELKWRAFATGSD
DSVRLAKDFNSANPHYGQFGLGTKTWEGDAWKIGGGTNWGWYAYDPKLNLFYYGSGNPAPWNETMR
PGDNKWTMTIWGRDLDTGMAKWGYQKTPHDEWDFAGVNQMVLTDQPVNGKMTPLLSHIDRNGILYT
LNRENGNLIVAEKVDPAVNVFKKVDLKTGTPVRDPEFATRMDHKGTNICPSAMGFHNQGVDSYDPE
SRTLYAGLNHICMDWEPFMLPYRAGQFFVGATLAMYPGPNGPTKKEMGQIRAFDLTTGKAKWTKWE
KFAAWGGTLYTKGGLVWYATLDGYLKALDNKDGKELWNFKMPSGGIGSPMTYSFKGKQYIGSMYGV
GGWPGVGLVFDLTDPSAGLGAVGAFRELQNHTQMGGGLMVFSL

>1WSRA

AQEVLRRTPLYDFHLAHGGKMVAFAGWSLPVQYRDSHTDSHLHTRQHCSLFDVSHMLQTKILGSDR

VKLMESLVVGDIAELRPNQGTLSLFTNEAGGILDDLIVTNTSEGHLYVVSNAGCWEKDLALMQDKV
RELQNQGRDVGLEVLDNALLALQGPTAAQVLQAGVADDLRKLPFMTSAVMEVFGVSGCRVTRCGYT
GEDGVEISVPVAGAVHLATAILKNPEVKLAGLAARDSLRLEAGLCLYGNDIDEHTTPVEGSLSWTL
GKRRRAAMDFPGAKVIVPQLKGRVQRRRVGLMCEGAPMRAHSPILNMEGTKIGTVTSGCPSPSLKK
NVAMGYVPCEYSRPGTMLLVEVRRKQQMAVVSKMPFVPTNYYTLK
>1BCH1
AIEVKLANMEAEINTLKSKLELTNKLHAFSMGKKSGKKFFVTNHERMPFSKVKALCSELRGTVAIP
RNAEENKAIQEVAKTSAFLGITDEVTEGQFMYVTGGRLTYSNWKKDQPDDWYGHGLGGGEDCVHIV
DNGLWNDISCQASHTAVCEFPA
>1DXKA
SQKVEKTVIKNETGTISISQLNKNVWVHTELGSFNGEAVPSNGLVLNTSKGLVLVDSSWDDKLTKE
LIEMVEKKFQKRVTDVIITHAHADRIGGIKTLKERGIKAHSTALTAELAKKNGYEEPLGDLQTVTN
LKFGNMKVETFYPGKGHTEDNIVVWLPQYNILVGGSLVKSTSAKDLGNVADAYVNEWSTSIENVLK
RYRNINAVVPGHGEVGDKGLLLHTLDLLK
>3H8TA
DEPNQPSTPEAVTKTVTIDASKYETWQYFSFSKGEVVNVTDYKNDLNWDMALHRYDVRLNCGESGK
GKGGAVFSGKTEMDQATTVPTDGYTVDVLGRITVKYEMGPDGHQMEYEEQGFSEVITGKKNAQGFA
SGGWLEFSHGPAGPTYKLSKRVFFVRGADGNIAKVQFTDYQDAELKKGVITFTYTYPVK
>3M7OA
MNGVAAALLVWILTSPSSSDHGSENGWPKHTACNSGGLEVVYQSCDPLQDFGLSIDQCSKQIQSNL
NIRFGIILRQDIRKLFLDITLMAKGSSILNYSYPLCEEDQPKFSFCGRRKGEQIYYAGPVNNPGLD
VPQGEYQLLLELYNENRATVACANATVTSS
>3OYYA
MASMKTAQEFRAGQVANINGAPWVIQKAEFNKSGRNAAVVKMKLKNLLTGAGTETVFKADDKLEPI
ILDRKEVTYSYFADPLYVFMDSEFNQYEIEKDDLEGVLTFIEDGMTDICEAVFYNDKVISVELPTT
IVRQIAYTEPAVRGDTSGKVMKTARLNNGAELQVSAFCEIGDSIEIDTRTGEYKSRVKA
>1FAZA
APADKPQVLASFTQTSASSQNAWLAANRNQSAWAAYEFDWSTDLCTQAPDNPFGFPFNTACARHDF
GYRNYKAAGSFDANKSRIDSAFYEDMKRVCTGYTGEKNTACNSTAWTYYQAVKIFG
>1ODZA
MRADVKPVTVKLVDSQATMETRSLFAFMQEQRRHSIMFGHQHETTQGLTITRTDGTQSDTFNAVGD
FAAVYGWDTLSIVAPKAEGDIVAQVKKAYARGGIITVSSHFDNPKTDTQKGVWPVGTSWDQTPAVV
DSLPGGAYNPVLNGYLDQVAEWANNLKDEQGRLIPVIFRLYHENTGSWFWWGDKQSTPEQYKQLFR
YSVEYLRDVKGVRNFLYAYSPNNFWDVTEANYLERYPGDEWVDVLGFDTYGPVADNADWFRNVVAN
AALVARMAEARGKIPVISGIGIRAPDIEAGLYDNQWYRKLISGLKADPDAREIAFLLVWRNAPQGV
PGPNGTQVPHYWVPANRPENINNGTLEDFQAFYADEFTAFNRDIEQVYQRPTLIVK
>1PF3A
AERPTLPIPDLLTTDARNRIQLTIGAGQSTFGGKTATTWGYNGNLLGPAVKLQRGKAVTVDIYNQL
TEETTLHWHGLEVPGEVDGGPQGIIPPGGKRSVTLNVDQPAATCWFHPHQHGKTGRQVAMGLAGLV
VIEDDEILKLMLPKQWGIDDVPVIVQDKKFSADGQIDYQLDVMTAAVGWFGDTLLTNGAIYPQHAA
PRGWLRLRLLNGCNARSLNFATSDNRPLYVIASDGGLLPEPVKVSELPVLMGERFEVLVEVNDNKP
FDLVTLPVSQMGMAIAPFDKPHPVMRIQPIAISASGALPDTLSSLPALPSLEGLTVRKLQLSMDPM
LDMMGMQMLMEKYGDQAMAGMDHSQMMGHMGHGNMNHMNHGGKFDFHHANKINGQAFDMNKPMFAA
AKGQYERWVISGVGDMLLHPFHIHGTQFRILSENGKPPAAHRAGWKDTVKVEGNVSEVLVKFNHDA

PKEHAYMAHCHLLEHEDTGMMLGFTVSAWSHPNFEK
>1UWLA
MTDNNNYRDVEIRAPRGNKLTAKSWLTEAPLRMLMNNLDPQVAENPKELVVYGGIGRAARNWECYD
KIVETLTRLEDDETLLVQSGKPVGVFKTHSNAPRVLIANSNLVPHWANWEHFNELDAKGLAMYGQM
TAGSWIYIGSQGIVQGTYETFVEAGRQHYGGSLKGKWVLTAGLGGMGGAQPLAATLAGACSLNIES
QQSRIDFRLETRYVDEQATDLDDALVRIAKYTAEGKAISIALHGNAAEILPELVKRGVRPDMVTDQ
TSAHDPLNGYLPAGWTWEQYRDRAQTEPAAVVKAAKQSMAVHVQAMLDFQKQGVPTFDYGNNIRQM
AKEEGVANAFDFPGFVPAYIRPLFCRGVGPFRWAALSGEAEDIYKTDAKVKELIPDDAHLHRWLDM
ARERISFQGLPARICWVGLGLRAKLGLAFNEMVRSGELSAPVVIGRDHLDSGSVSSPNRETEAMRD
GSDAVSDWPLLNALLNTAGGATWVSLHHGGGVGMGFSQHSGMVIVCDGTDEAAERIARVLTNDPGT
GVMRHADAGYDIAIDCAKEQGLDLPMITG
>3MCXA
GDWLDLNTTSSVETGQAIVTLDDAQIALNGIYRLASGHSYYGDNYWYYGDCRAADVQARITKGDGK
RVSPYYEYNVLASDNLNIVLPWNTVYKVIRQTNNLIQKIESGSIQSSDTKELNRIKSEALVMRGLS
LFNLTRLFGMPYTNDKGASLGVPIETSPSDPTHKPSRSTVAQCYEQVVSDMSNALSGLRQETSNGY
INYWAAQALLSRVYLNMGEYQKAYDAATDVIKNNGGRYQLYSYEEYPNVWGQDFQSESLFELYITL
SEPSGGTGGEGAPMVYANEATVDWNNLILSEDFLNLLNEDPKDVRHCLTKESVIENNTGLPAAAMH
EKVYLAKFPGKTGDDPKTNNICIIRLSEVYLNAAEAGLKKGTDIEEAQGYLNDIISRRTTDTSQQV
STETFTLDRILKERRKELVGEGEVFYDYLRNGLAIERKGSWHLETLKASNAQKIEATDLRIALPIP
QSEIDANPNIQQNPR
>1Z82A
MGSDKIHHHHHHMEMRFFVLGAGSWGTVFAQMLHENGEEVILWARRKEIVDLINVSHTSPYVEESK
ITVRATNDLEEIKKEDILVIAIPVQYIREHLLRLPVKPSMVLNLSKGIEIKTGKRVSEIVEEILGC
PYAVLSGPSHAEEVAKKLPTAVTLAGENSKELQKRISTEYFRVYTCEDVVGVEIAGALKNVIAIAA
GILDGFGGWDNAKAALETRGIYEIARFGMFFGADQKTFMGLAGIGDLMVTCNSRYSRNRRFGELIA
RGFNPLKLLESSNQVVEGAFTVKAVMKIAKENKIDMPISEEVYRVVYEGKPPLQSMRDLMRRSLKD
EFWAS
>3GZRA
GGEGTDAIQALIQAYFTAWNTNAPERFAEIFWPDGSWVNVVGMHWRGRDQIVFAHTAFLKTIFKDC
KQELVTIEARTIAPGSALAVVTLIQDAYVTPDGRQMPRAHDRLTLLAVEREGVWRFIHGHNTIVNP
DAANNDPVLRMKPA
>2A33A
MEIKGESMQKSKFRRICVFCGSSQGKKSSYQDAAVDLGNELVSRNIDLVYGGGSIGLMGLVSQAVH
DGGRHVIGIIPKTLMPRELTGETVGEVRAVADMHQRKAEMAKHSDAFIALPGGYGTLEELLEVITW
AQLGIHDKPVGLLNVDGYYNSLLSFIDKAVEEGFISPTAREIIVSAPTAKELVKKLEEYAPCHERV
ATKLCWEMERIGYSSEE
>1DHIA
MISLIAALAVDRVIGMENAMPWNLPASLAWFKRNTLDKPVIMGRHTWESIGRPLPGRKNIILSSQP
GTDDRVTWVKSVDEAIAACGDVPEIMVIGGGRVYEQFLPKAQKLYLTHIDAEVEGDTHFPDYEPDD
WESVFSEFHDADAQNSHSYCFEILERR
>3A8GA
MSVTIDHTTENAAPAQAPVSDRAWALFRALDGKGLVPDGYVEGWKKTFEEDFSPRRGAELVARAWT
DPEFRQLLLTDGTAAVAQYGYLGPQGEYIVAVEDTPTLKNVIVCSLCACTAWPILGLPPTWYKSFE
YRARVVREPRKVLSEMGTEIASDIEIRVYDTTAETRYMVLPQRPAGTEGWSQEQLQEIVTKDCLIG

VAIPQVPTV
>1VDWA
MSVKTWRKIAIDIIRDFDHNIMPLFGNPKASETISISPSGDETKVVDKVAENIIISKFKDLGVNVV
SEEIGRIDQGSDYTVVVDPLDGSYNFINGIPFFAVSVAIFHEKDPIYAFIYEPIVERLYEGIPGKG
SYLNGEKIKVRELAEKPSISFYTKGKGTKIIDKVKRTRTLGAIALELAYLARGALDAVVDIRNYLR
PTDIAAGVVIAREAGAIVKDLDGKDVEITFSATEKVNIIAANNEELLETILRSIEK
>2QKFA
MDIKINDITLGNNSPFVLFGGINVLESLDSTLQTCAHYVEVTRKLGIPYIFKASFDKANRSSIHSY
RGVGLEEGLKIFEKVKAEFGIPVITDVHEPHQCQPVAEVCDVIQLPAFLARQTDLVVAMAKTGNVV
NIKKPQFLSPSQMKNIVEKFHEAGNGKLILCERGSSFGYDNLVVDMLGFGVMKQTCGNLPVIFDVT
HSLQTRDAGSAASGGRRAQALDLALAGMATRLAGLFLESHPDPKLAKCDGPSALPLHLLEDFLIRI
KALDDLIKSQPILTIE
>2ZU1A
GPAFEFAVAMMKRNSSTVKTEYGEFTMLGIYDRWAVLPRHAKPGPTILMNDQEVGVLDAKELVDKD
GTNLELTLLKLNRNEKFRDIRGFLAKEEVEVNEAVLAINTSKFPNMYIPVGQVTEYGFLNLGGTPT
KRMLMYNFPTRAGQAGGVLMSTGKVLGIHVGGNGHQGFSAALLKHYFNDEQ
>2GJPA
HHMGTNGTMMQYFEWHLPNDGQHWNRLRDDASNLRNRGITAIWIPPAWKGTSQNDVGYGAYDLYDL
GEFNQKGTVRTKYGTRSQLESAIHALKNNGVQVYGDVVMNHKGGADATENVLAVEVNPNNRNQEIS
GDYTIEAWTKFDFPGRGNTYSDFKWRWYHFDGVDWDQSRQFQNRIYKFRGDGKAWDWEVDSENGNY
DYLMYADVDMDHPEVVNELRRWGEWYTNTLNLDGFRIDAVKHIKYSFTRDWLTHVRNATGKEMFAV
AEFWKNDLGALENYLNKTNWNHSVFDVPLHYNLYNASNSGGNYDMAKLLNGTVVQKHPMHAVTFVD
NHDSQPGESLESFVQEWFKPLAYALILTREQGYPSVFYGDYYGIPTHSVPAMKAKIDPILEARQNF
AYGTQHDYFDHHNIIGWTREGNTTHPNSGLATIMSDGPGGEKWMYVGQNKAGQVWHDITGNKPGTV
TINADGWANFSVNGGSVSIWVKR
>3B5NC
GSIKFTKQSSVASTRNTLKMAQDAERAGMNTLGMLGHQSEQLNNVEGNLDLMKVQNKVADEKVAEL
KKLQ
>1HQGA
MSSKPKPIEIIGAPFSKGQPRGGVEKGPAALRKAGLVEKLKETEYNVRDHGDLAFVDVPNDSPFQI
VKNPRSVGKANEQLAAVVAETQKNGTISVVLGGDHSMAIGSISGHARVHPDLCVIWVDAHTDINTP
LTTSSGNLCGQPVAFLLKELKGKFPDVPGFSWVTPCISAKDIVYIGLRDVDPGEHYIIKTLGIKYF
SMTEVDKLGIGKVMEETFSYLLGRKKRPIHLSFDVDGLDPVFTPATGTPVVGGLSYREGLYITEEI
YKTGLLSGLDIMEVNPTLGKTPEEVTRTVNTAVALTLSCFGTKREGNHKPETDYLKPPK
>2OQYA
MKITDLELHAVGIPRHTGFVNKHVIVKIHTDEGLTGIGEMSDFSHLPLYSVDLHDLKQGLLSILLG
QNPFDLMKINKELTDNFPETMYYYEKGSFIRNGIDNALHDLCAKYLDISVSDFLGGRVKEKIKVCY
PIFRHRFSEEVESNLDVVRQKLEQGFDVFRLYVGKNLDADEEFLSRVKEEFGSRVRIKSYDFSHLL
NWKDAHRAIKRLTKYDLGLEMIESPAPRNDFDGLYQLRLKTDYPISEHVWSFKQQQEMIKKDAIDI
FNISPVFIGGLTSAKKAAYAAEVASKDVVLGTTQELSVGTAAMAHLGCSLTNINHTSDPTGPELYV
GDVVKNRVTYKDGYLYAPDRSVKGLGIELDESLLAKYQVPDLSWDNVTVHQLQDRTADTKS
>2OQAA
DVSFSLSGSSSTSYSKFIGALRKALPSNGTVYNITLLLSSASGASRYTLMKLSNYDGKAITVAIDV
TNVYIMGYLVNSTSYFFNESDAKLASQYVFAGSTIVTLPYSGNYEKLQTAAGKIREKIPLGFPALD

SAITTLFHYDSTAAAAAFLVIIQTTAESSRFKYIEGQIIMRISKNGVPSLATISLENEWSALSKQI
QLAQTNNGTFKTPVVIMDAGGQRVEIGNVGSKVVTKNIQLLLN
>3QD5A
GPGSMAATPLPPLRLAIACDDAGVSYKEALKAHLSDNPLVSSITDVGVTSTTDKTAYPHVAIQAAQ
LIKDGKVDRALMICGTGLGVAISANKVPGIRAVTAHDTFSVERAILSNDAQVLCFGQRVIGIELAK
RLAGEWLTYRFDQKSASAQKVQAISDYEKKFVEVN
>3D1RA
GMRRELAIEFSRVTESAALAGYKWLGRGDKNTADGAAVNAMRIMLNQVNIDGTIVIGEGEIAEAPM
LYIGEKVGTGRGDAVDIAVDPIEGTRMTAMGQANALAVLAVGDKGCFLNAPDMYMEKLIVGPGAKG
TIDLNLPLADNLRNVAAALGKPLSELTVTILAKPRHDAVIAEMQQLGVRVFAIPDGDVAASILTCM
PDSEVDVLYGIGGAPEGVVSAAVIRALDGDMNGRLLARHDVKGDNEENRRIGEQELARCKAMGIEA
GKVLRLGDMARSDNVIFSATGITKGDLLEGISRKGNIATTETLLIRGKSRTIRRIQSIHYLDRKDP
EMQVHIL
>1RWIB
RPSWSPTQASGQTVLPFTGIDFRLSPSGVAVDSAGNVYVTSEGMYGRVVKLATGSTGTTVLPFNGL
YQPQGLAVDGAGTVYVTDFNNRVVTLAAGSNNQTVLPFDGLNYPEGLAVDTQGAVYVADRGNNRVV
KLAAGSKTQTVLPFTGLNDPDGVAVDNSGNVYVTDTDNNRVVKLEAESNNQVVLPFTDITAPWGIA
VDEAGTVYVTEHNTNQVVKLLAGSTTSTVLPFTGLNTPLAVAVDSDRTVYVADRGNDRVVKLTSLE
HHHHHH
>3NXDC
GSHMGGVEVLEVKTGVDSITEVECFLTPEMGDPDEHLRGFSKSISISDTFESDSPNRDMLPCYSVA
RIPLPNLNEDLTCGNILMWEAVTLKTEVIGVTSLMNVHSNGQATHDNGAGKPVQGTSFHFFSVGGE
ALELQGVLFNYRTKYPDGTIFPKNATVQSQVMNTEHKAYLDKNKAYPVECWVPDPTRNENTRYFGT
LTGGENVPPVLHITNTATTVLLDEFGVGPLCKGDNLYLSAVDVCGMFTNRSGSQQWRGLSRYFKVQ
LRKRRVKN
>1TEJB
NSVNPCCDPQTCKPIEGKHCISGPCCENCYFLRSGTICQRARGDGNNDYCTGITPDCPRNRYNV
>3EWDA
MNILQEPIDFLKKEELKNIDLSQMSKKERYKIWKRIPKCELHCHLDLCFSADFFVSCIRKYNLQPN
LSDEEVLDYYLFAKGGKSLGEFVEKAIKVADIFHDYEVIEDLAKHAVFNKYKEGVVLMEFRYSPTF
VAFKYNLDIELIHQAIVKGIKEVVELLDHKIHVALMCIGTGHEAANIKASADFCLKHKADFVGFDH
GGHEVDLKEYKEIFDYVRESGVPLSVHAGEDVTLPNLNTLYSAIQVLKVERIGHGIRVAESQELID
MVKEKNILLEVCPISNVLLKNAKSMDTHPIRQLYDAGVKVSVNSDDPGMFLTNINDDYEELYTHLN
FTLEDFMKMNEWALEKSFMDSNIKDKIKNLYFKGEFEAYV
>1EW4A
MNDSEFHRLADQLWLTIEERLDDWDGDSDIDCEINGGVLTITFENGSKIIINRQEPLHQVWLATKQ
GGYHFDLKGDEWICDRSGETFWDLLEQAATQQAGETVSFR
>1H6WA
LSYPNATESVYGLTRYSTNDEAIAGVNNESSITPAKFTVALNNVFETRVSTESSNGVIKISSLPQA
LAGADDTTAMTPLKTQQLAVKLIAQIAPSKNAATESEQGVIQLATVAQARQGTLREGYAISPYTFM
NSTATEEYKGVIKLGTQSEVNSNNASVAVTGATLNGRGSTTSMRGVVKLTTTAGSQSGGDASSALA
WNADVIHQRGGQTINGTLRINNTLTIASGGANITGTVNMTGGYIQGKRVVTQNEIDRTIPVGAIMM
WAADSLPSDAWRFCHGGTVSASDCPLYASRIGTRYGGSSSNPGLPDMR
>2CB9A

MARSQLSAAGEQHVIQLNQQGGKNLFCFPPISGFGIYFKDLALQLNHKAAVYGFHFIEEDSRIEQY
VSRITEIQPEGPYVLLGYSAGGNLAFEVVQAMEQKGLEVSDFIIVDAYKKDQSITADTENDDSAAY
LPEAVRETVMQKKRCYQEYWAQLINEGRIKSNIHFIEAGIQTETSGAMVLQKWQDAAEEGYAEYTG
YGAHKDMLEGEFAEKNANIILNILDKINSDQKVLPNKHGSHHHHHH
>1GXJA
EKEMIERDMREYRGFSRAVRAVFEEKERFPGLVDVVSNLIEVDEKYSLAVSVLLGGTAQNIVVRNV
DTAKAIVEFLKQNEAGRVTILPLDLIDGSFNRISGLENERGFVGYAVDLVKFPSDLEVLGGFLFGN
SVVVETLDDAIRMKKKYRLNTRIATLDGELISGRGAITGGREERSSNVFERRIK
>1VLCA
MGSDKIHHHHHHMKIAVLPGDGIGPEVVREALKVLEVVEKKTGKTFEKVFGHIGGDAIDRFGEPLP
EETKKICLEADAIFLGSVGGPKWDDLPPEKRPEIGGLLALRKMLNLYANIRPIKVYRSLVHVSPLK
EKVIGSGVDLVTVRELSYGVYYGQPRGLDEEKGFDTMIYDRKTVERIARTAFEIAKNRRKKVTSVD
KANVLYSSMLWRKVVNEVAREYPDVELTHIYVDNAAMQLILKPSQFDVILTTNMFGDILSDESAAL
PGSLGLLPSASFGDKNLYEPAGGSAPDIAGKNIANPIAQILSLAMMLEHSFGMVEEARKIERAVEL
VIEEGYRTRDIAEDPEKAVSTSQMGDLICKKLEEIW
>2VLBA
GQMQQASTPTIGMIVPPAAGLVPADGARLYPDLPFIASGLGLGSVTPEGYDAVIESVVDHARRLQK
QGAAVVSLMGTSLSFYRGAAFNAALTVAMREATGLPCTTMSTAVLNGLRALGVRRVALATAYIDDV
NERLAAFLAEESLVPTGCRSLGITGVEAMARVDTATLVDLCVRAFEAAPDSDGILLSCGGLLTLDA
IPEVERRLGVPVVSSSPAGFWDAVRLAGGGAKARPGYGRLFDESGGSHHHHHH
>2VLPB
MESKRNKPGKATGKGKPVGDKWLDDAGKDSGAPIPDRIADKLRDKEFKSFDDFAKAVWEEVSKDPE
LSKNLNPSNKSSVSKGYSPFTPKNQQVGGRKVYELHHDKPISQGGEVYDMDNIRVTTPKRHIDIHR
GK
>2Y88A
MPLILLPAVNVVEGRAVRLVQGKAGSQTEYGSAVDAALGWQRDGAEWIHLVDLDAAFGRGSNHELL
AEVVGKLDVQVELSGGIRDDESLAAALATGCARVNVGTAALENPQWCARVIGEHGDQVAVGLDVQI
IDGEHRLRGRGWETDGGDLWDVLERLDSEGCSRFVVTDITKDGTLGGPNLDLLAGVADRTDAPVIA
SGGVSSLDDLRAIATLTHRGVEGAIVGKALYARRFTLPQALAAVRD
>3DV9A
SNAMFKEAINNYLHTHGYESIDLKAVLFDMDGVLFDSMPNHAESWHKIMKRFGFGLSREEAYMHEG
RTGASTINIVSRRERGHDATEEEIKAIYQAKTEEFNKCPKAERMPGALEVLTKIKSEGLTPMVVTG
SGQTSLLDRLNHNFPGIFQANLMVTAFDVKYGKPNPEPYLMALKKGGFKPNEALVIENAPLGVQAG
VAAGIFTIAVNTGPLHDNVLLNEGANLLFHSMPDFNKNWETLQSALKQD
>2QRJA
MGHHHHHHHHHHSSGHIEGRHMAAVTLHLRAETKPLEARAALTPTTVKKLIAKGFKIYVEDSPQST
FNINEYRQAGAIIVPAGSWKTAPRDRIIIGLKEMPETDTFPLVHEHIQFAHCYKDQAGWQNVLMRF
IKGHGTLYDLEFLENDQGRRVAAFGFYAGFAGAALGVRDWAFKQTHSDDEDLPAVSPYPNEKALVK
DVTKDYKEALATGARKPTVLIIGALGRCGSGAIDLLHKVGIPDANILKWDIKETSRGGPFDEIPQA
DIFINCIYLSKPIAPFTNMEKLNNPNRRLRTVVDVSADTTNPHNPIPIYTVATVFNKPTVLVPTTA
GPKLSVISIDHLPSLLPREASEFFSHDLLPSLELLPQRKTAPVWVRAKKLFDRHCARVKRSSRL
>2O6XA
NDDLWHQWKRMYNKEYNGADDQHRRNIWEKNVKHIQEHNLRHDLGLVTYTLGLNQFTDMTFEEFKA
KYLTEMSRASDILSHGVPYEANNRAVPDKIDWRESGYVTEVKDQGNCGSGWAFSTTGTMEGQYMKN

ERTSISFSEQQLVDCSRPWGNNGCGGGLMENAYQYLKQFGLETESSYPYTAVEGQCRYNKQLGVAK
VTGFYTVHSGSEVELKNLVGAEGPAAVAVDVESDFMMYRSGIYQSQTCSPLRVNHAVLAVGYGTQG
GTDYWIVKNSWGLSWGERGYIRMVRNRGNMCGIASLASLPMVARFP
>1GQGA
DTSSLIVEDAPDHVRPYVIRHYSHARAVTVDTQLYRFYVTGPSSGYAFTLMGTNAPHSDALGVLPH
IHQKHYENFYCNKGSFQLWAQSGNETQQTRVLSSGDYGSVPRNVTHTFQIQDPDTEMTGVIVPGGF
EDLFYYLGTNATDTTHTPYIPSSSDSSSTTGPDSSTISTLQSFDVYAELSFTPRTDTVNGTAPANT
VWHTGANALASTAGDPYFIANGWGPKYLNSQYGYQIVAPFVTATQAQDTNYTLSTISMSTTPSTVT
VPTWSFPGACAFQVQEGRVVVQIGDYAATELGSGDVAFIPGGVEFKYYSEAYFSKVLFVSSGSDGL
DQNLVNGGEEWSSVSFPADW
>3MSUA
SNAMEVMLMSKYATLKYADKNIEIELPVYSPSLGNDCIDVSSLVKHGIFTYDPGFMSTAACESKIT
YIDGGKGVLLHRGYPIEEWTQKSNYRTLCYALIYGELPTDEQVKSFRQEIINKMPVCEHVKAAIAA
MPQHTHPMSSLIAGVNVLAAEHIHNGQKESQDEVAKNIVAKIATIAAMAYRHNHGKKFLEPKMEYG
YAENFLYMMFADDESYKPDELHIKAMDTIFMLHADHEQNASTSTVRLSGSTGNSPYAAIIAGITAL
WGPAHGGANEAVLKMLSEIGSTENIDKYIAKAKDKDDPFRLMGFGHRVYKNTDPRATAMKKNCEEI
LAKLGHSDNPLLTVAKKLEEIALQDEFFIERKLFSNVDFYSGIILKAMGIPEDMFTAIFALARTSG
WISQWIEMVNDPAQKIGRPRQLYTGATNRNF
>1KTBA
LENGLARTPPMGWLAWERFRCNVNCREDPRQCISEMLFMEMADRIAEDGWRELGYKYINIDDCWAA
KQRDAEGRLVPDPERFPRGIKALADYVHARGLKLGIYGDLGRLTCGGYPGTTLDRVEQDAQTFAEW
GVDMLKLDGCYSSGKEQAQGYPQMARALNATGRPIVYSCSWPAYQGGLPPKVNYTLLGEICNLWRN
YDDIQDSWDSVLSIVDWFFTNQDVLQPFAGPGHWNDPDMLIIGNFGLSYEQSRSQMALWTIMAAPL
LMSTDLRTISPSAKKILQNRLMIQINQDPLGIQGRRIIKEGSHIEVFLRPLSQAASALVFFSRRTD
MPFRYTTSLAKLGFPMGAAYEVQDVYSGKIISGLKTGDNFTVIINPSGVVMWYLCPKALLIQQQAP
GGPSRLPLL
>2WGVA
MGSITENTSWNKEFSAEAVNGVFVLCKSSSKSCATNDLARASKEYLPASTFKIPNAIIGLETGVIK
NEHQVFKWDGKPRAMKQWERDLTLRGAIQVSATPVFQQIAREVGEVRMQKYLKKFSYGNQNISGGI
DKFWLEGQLRISAVNQVEFLESLYLNKLSASKENQLIVKEALVTEAAPEYLVHSKTGFSGVGTESN
PGVAWWVGWVEKETEVYFFAFNMDIDNESKLPLRKSIPTKIMESEGIIGG
>3GBXA
SNAMLKREMNIADYDAELWQAMEQEKVRQEEHIELIASENYTSPRVMQAQGSQLTNKYAEGYPGKR
YYGGCEYVDVVEQLAIDRAKELFGADYANVQPHSGSQANFAVYTALLQPGDTVLGMNLAQGGHLTH
GSPVNFSGKLYNIVPYGIDESGKIDYDEMAKLAKEHKPKMIIGGFSAYSGVVDWAKMREIADSIGA
YLFVDMAHVAGLIAAGVYPNPVPHAHVVTTTTHKTLAGPRGGLILAKGGDEELYKKLNSAVFPSAQ
GGPLMHVIAGKAVALKEAMEPEFKVYQQQVAKNAKAMVEVFLNRGYKVVSGGTENHLFLLDLVDKN
LTGKEADAALGRANITVNKNSVPNDPKSPFVTSGIRIGSPAVTRRGFKEAEVKELAGWMCDVLDNI
NDEATIERVKAKVLDICARFPVYA
>1E6WA
AAAVRSVKGLVAVITGGASGLGLSTAKRLVGQGATAVLLDVPNSEGETEAKKLGGNCIFAPANVTS
EKEVQAALTLAKEKFGRIDVAVNCAGIAVAIKTYHEKKNQVHTLEDFQRVINVNLIGTFNVIRLVA
GVMGQNEPDQGGQRGVIINTASVAAFEGQVGQAAYSASKGGIVGMTLPIARDLAPIGIRVVTIAPG
LFATPLLTTLPDKVRNFLASQVPFPSRLGDPAEYAHLVQMVIENPFLNGEVIRLDGAIRMQP

>1E6FA
MKSNEHDDCQVTNPSTGHLFDLSSLSGRAGFTAAYSEKGLVYMSICGENENCPPGVGACFGQTRIS
VGKANKRLRYVDQVLQLVYKDGSPCPSKSGLSYKSVISFVCRPEAGPTNRPMLISLDKQTCTLFFS
WHTPLACEQAT
>3A75B
TTHFTVADRWGNVVSYTTTIEQLFGTGIMVPDYGVILNNELTDFDAIPGGANEVQPNKRPLSSMTP
TILFKDDKPVLTVGSPGGATIISSVLQTILYHIEYGMELKAAVEEPRIYTNSMSSYRYEDGVPKDV
LSKLNGMGHKFGTSPVDIGNVQSISIDHENGTFKGVADSSRNGAAIGINLKRK
>3FIRA
MKKIITLFGACALAFSMANADVNLYGPGGPHTALKDIANKYSEKTGVKVNVNFGPQATWFEKAKKD
ADILFGASDQSALAIASDFGKDFNVSKIKPLYFREAIILTQKGNPLKIKGLKDLANKKVRIVVPEG
AGESNTSGTGVWEDMIGRTQDIKTIQNFRNNIVAFVPNSGSARKLFAQDQADAWITWIDWSKSNPD
IGTAVAIEKDLVVYRTFNVIAKEGASKETQDFIAYLSSKEAKEIFKKYGWREH
>2IAPA
IPVIEPLFTKVTEDIPGAQGPVFDKNGDFYIVAPEVEVNGKPAGEILRIDLKTGKKTVICKPEVNG
YGGIPAGCQCDRDANQLFVADMRLGLLVVQTDGTFEEIAKKDSEGRRMQGCNDCAFDYEGNLWITA
PAGEVAPADYTRSMQEKFGSIYCFTTDGQMIQVDTAFQFPNGIAVRHMNDGRPYQLIVAETPTKKL
WSYDIKGPAKIENKKVWGHIPGTHEGGADGMDFDEDNNLLVANWGSSHIEVFGPDGGQPKMRIRCP
FEKPSNLHFKPQTKTIFVTEHENNAVWKFEWQRNGKKQYCETLKFGIF
>1Q74A
MSETPRLLFVHAHPDDESLSNGATIAHYTSRGAQVHVVTCTLGEEGEVIGDRWAQLTADHADQLGG
YRIGELTAALRALGVSAPIYLGGAGRWRDSGMAGTDQRSQRRFVDADPRQTVGALVAIIRELRPHV
VVTYDPNGGYGHPDHVHTHTVTTAAVAAAGVGSGTADHPGDPWTVPKFYWTVLGLSALISGARALV
PDDLRPEWVLPRADEIAFGYSDDGIDAVVEADEQARAAKVAALAAHATQVVVGPTGRAAALSNNLA
LPILADEHYVLAGGSAGARDERGWETDLLAGLGFTASGT
>2CWLA
MFLRIDRLQIELPMPKEQDPNAAAAVQALLGGRFGEMSTLMNYMYQSFNFRGKKALKPYYDLIANI
ATEELGHIELVAATINSLLAKNPGKDLEEGVDPESAPLGFAKDVRNAAHFIAGGANSLVMGAMGEH
WNGEYVFTSGNLILDLLHNFFLEVAARTHKLRVYEMTDNPVAREMIGYLLVRGGVHAAAYGKALES
LTGVEMTKMLPIPKIDNSKIPEAKKYMDLGFHRNLYRFSPEDYRDLGLIWKGASPEDGTEVVVVDG
PPTGGPVFDAGHDAAEFAPEFHPGELYEIAKKLYEKAK
>3NK6A
SEFMTEPAIITNASDPAVQRIIDVTKHSRASIKTTLIEDTEPLMECIRAGVQFIEVYGSSGTPLDP
ALLDLCRQREIPVRLIDVSIVNQLFKAERKAKVFGIARVPRPARLADIAERGGDVVVLDGVKIVGN
IGAIVRTSLALGAAGIVLVDSDLATIADRRLLRASRGYVFSLPVVLADREEAVSFLRDNDIALMVL
DTDGDLGVKDLGDRADRMALVFGSEKGGPSGLFQEASAGTVSIPMLSSTESLNVSVSVGIALHERS
ARNFAVRRAAAQA
>1V3IA
ATSDSNMLLNYVPVYVMLPLGVVNVDNVFEDPDGLKEQLLQLRAAGVDGVMVDVWWGIIELKGPKQ
YDWRAYRSLLQLVQECGLTLQAIMSFHQCGGNVGDIVNIPIPQWVLDIGESNHDIFYTNRSGTRNK
EYLTVGVDNEPIFHGRTAIEIYSDYMKSFRENMSDFLESGLIIDIEVGLGPAGELRYPSYPQSQGW
EFPGIGEFQCYDKYLKADFKAAVARAGHPEWELPDDAGKYNDVPESTGFFKSNGTYVTEKGKFFLT
WYSNKLLNHGDQILDEANKAFLGCKVKLAIKVSGIHWWYKVENHAAELTAGYYNLNDRDGYRPIAR
MLSRHHAILNFTCLEMRDSEQPSDAKSGPQELVQQVLSGGWREDIRVAGQNALPRYDATAYNQIIL

NARPQGVNNNGPPKLSMFGVTYLRLSDDLLQKSNFNIFKKFVLKMHADQDYCANPQKYNHAITPLK
PSAPKIPIEVLLEATKPTLPFPWLPETDMKVDG
>2W87A
ALLLQEAQAGFCRVDGTIDNNHTGFTGSGFANTNNAQGAAVVWAIDATSSGRRTLTIRYANGGTAN
RNGSLVINGGSNGNYTVSLPTTGAWTTWQTATIDVDLVQGNNIVQLSATTAEGLPNIDSLSVVGGT
VRAGNCG
>2W8TA
TEAAAQPHALPADAPDIAPERDLLSKFDGLIAERQKLLDSGVTDPFAIVMEQVKSPTEAVIRGKDT
ILLGTYNYMGMTFDPDVIAAGKEALEKFGSGTCGSRMLNGTFHDHMEVEQALRDFYGTTGAIVFST
GYMANLGIISTLAGKGEYVILDADSHASIYDGCQQGNAEIVRFRHNSVEDLDKRLGRLPKEPAKLV
VLEGVYSMLGDIAPLKEMVAVAKKHGAMVLVDEAHSMGFFGPNGRGVYEAQGLEGQIDFVVGTFSK
SVGTVGGFVVSNHPKFEAVRLACRPYIFTASLPPSVVATATTSIRKLMTAHEKRERLWSNARALHG
GLKAMGFRLGTETCDSAIVAVMLEDQEQAAMMWQALLDGGLYVNMARPPATPAGTFLLRCSICAEH
TPAQIQTVLGMFQAAGRAVGVIGLEHHHHHH
>1Y0BA
SNAMEALKRKIEEEGVVLSDQVLKVDSFLNHQIDPLLMQRIGDEFASRFAKDGITKIVTIESSGIA
PAVMTGLKLGVPVVFARKHKSLTLTDNLLTASVYSFTKQTESQIAVSGTHLSDQDHVLIIDDFLAN
GQAAHGLVSIVKQAGASIAGIGIVIEKSFQPGRDELVKLGYRVESLARIQSLEEGKVSFVQEVHS
>3K3KA
MGSSHHHHHHSSGLVPRGSHMPSELTPEERSELKNSIAEFHTYQLDPGSCSSLHAQRIHAPPELVW
SIVRRFDKPQTYKHFIKSCSVEQNFEMRVGCTRDVIVISGLPANTSTERLDILDDERRVTGFSIIG
GEHRLTNYKSVTTVHRFEKENRIWTVVLESYVVDMPEGNSEDDTRMFADTVVKLNLQKLATVAEAM
ARNSGDGSGSQVT
>3LIDA
MEKYQALLANNVENTAKEALHQLAYTGREYNNIQDQIETISDLLGHSQSLYDYLREPSKANLTILE
NMWSSVARNQKLYKQIRFLDTSGTEKVRIKYDFKTSIAGPSLILRDKSAREYFKYAQSLDNEQISA
WGIELERDKGELVYPLSPSLRILMPISVNDVRQGYLVLNVDIEYLSSLLNYSPVRDFHIELVKHKG
FYIASPDESRLYGDIIPERSQFNFSNMYPDIWPRVVSEQAGYSYSGEHLIAFSSIKFVSNEPLHLI
IDLSNEQLSKRATRDINDLIQESLEHHHHHH
>1PGTA
MPPYTVVYFPVRGRCAALRMLLADQGQSWKEEVVTVETWQEGSLKASCLYGQLPKFQDGDLTLYQS
NTILRHLGRTLGLYGKDQQEAALVDMVNDGVEDLRCKYVSLIYTNYEAGKDDYVKALPGQLKPFET
LLSQNQGGKTFIVGDQISFADYNLLDLLLIHEVLAPGCLDAFPLLSAYVGRLSARPKLKAFLASPE
YVNLPINGNGKQ
>1ZLHB
NECVSKGFGCLPQSDCPQEARLSYGGCSTVCCDLSKLTGCKGKGGECNPLDRQCKELQAESASCGK
GQKCCVWLH
>3CIFA
MGSSHHHHHHSSGLVPRGSHMTATLGINGFGRIGRLVLRACMERNDITVVAINDPFMDVEYMAYLL
KYDSVHGNFNGTVEVSGKDLCINGKVVKVFQAKDPAEIPWGASGAQIVCESTGVFTTEEKASLHLK
GGAKKVIISAPPKDNVPMYVMGVNNTEYDPSKFNVISNASSTTNCLAPLAKIINDKFGIVEGLMTT
VHSLTANQLTVDGPSKGGKDWRAGRCAGNNIIPASTGAAKAVGKVIPALNGKLTGMAIRVPTPDVS
VVDLTCKLAKPASIEEIYQAVKEASNGPMKGIMGYTSDDVVSTDFIGCKYSSILDKNACIALNDSF
VKLISWYDNESGYSNRLVDLAVYVASRGL

>2JINA
SMRVDYLVTEEEINLTRGPSGLGFNIVGGTDQQYVSNDSGIYVSRIKENGAAALDGRLQEGDKILS
VNGQDLKNLLHQDAVDLFRNAGYAVSLRVQHRESSI
>2RK6A
GSHMASKRALVILAKGAEEMETVIPVDVMRRAGIKVTVAGLAGKDPVQCSRDVVICPDASLEDAKK
EGPYDVVVLPGGNLGAQNLSESAAVKEILKEQENRKGLIAAICAGPTALLAHEIGFGSKVTTHPLA
KDKMMNGGHYTYSENRVEKDGLILTSRGPGTSFKFALAIVEALNGKEVAAQVKAPLVLKD
>2GAIA
MAKKVKKYIVVESPAKAKTIKSILGNEYEVFASMGHIIDLPKSKFGVDLEKDFEPEFAVIKGKEKV
VEKLKDLAKKGELLIASDMDREGEAIAWHIARVTNTLGRKNRIVFSEITPRVIREAVKNPREIDMK
KVRAQLARRILDRIVGYSLSPVLWRNFKSNLSAGRVQSATLKLVCDREREILRFVPKKYHRITVNF
DGLTAEIDVKEKKFFDAETLKEIQSIDELVVEEKKVSVKKFAPPEPFKTSTLQQEAYSKLGFSVSK
TMMIAQQLYEGVETKDGHIAFITYMRTDSTRVSDYAKEEARNLITEVFGEEYVGSKRERRKSNAKI
QDAHEAIRPTNVFMTPEEAGKYLNSDQKKLYELIWKRFLASQMKPSQYEETRFVLRTKDGKYRFKG
TVLKKIFDGYEKVWKTERNTGEFPFEEGESVKPVVVKIEEQETKPKPRYTEGSLVKEMERLGIGRP
STYASTIKLLLNRGYIKKIRGYLYPTIVGSVVMDYLEKKYSDVVSVSFTAEMEKDLDEVEQGKKTD
KIVLREFYESFSSVFDRNDRIVVDFPTNQKCSCGKEMRLSFGKYGFYLKCECGKTRSVKNDEIAVI
DDGKIFLGRKDSESGSPDGRSVEGKGNLSEKRRKGKKGS
>2GAKA
PEFFSVRHLELAGDDPYSNVNCTKILQGDPEEIQKVKLEILTVQFKKRPRWTPHDYINMTRDCASF
IRTRKYIVEPLTKEEVGFPIAYSIVVHHKIEMLDRLLRAIYMPQNFYCIHVDRKAEESFLAAVQGI
ASCFDNVFVASQLESVVYASWTRVKADLNCMKDLYRMNANWKYLINLCGMDFPIKTNLEIVRKLKC
STGENNLETEKMPPNKEERWKKRYAVVDGKLTNTGIVKAPPPLKTPLFSGSAYFVVTREYVGYVLE
NENIQKLMEWAQDTYSPDEFLWATIQRIPEVPGSFPSSNKYDLSDMNAIARFVKWQYFEGDVSNGA
PYPPCSGVHVRSVCVFGAGDLSWMLRQHHLFANKFDMDVDPFAIQCLDEHLRRKALENLEH
>2RLCA
CTGLALETKDGLHLFGRNMDIEYSFNQSIIFIPRNFKCVNKSNKKELTTKYAVLGMGTIFDDYPTF
ADGMNEKGLGCAGLNFPVYVSYSKEDIEGKTNIPVYNFLLWVLANFSSVEEVKEALKNANIVDIPI
SENIPNTTLHWMISDITGKSIVVEQTKEKLNVFDNNIGVLTNSPTFDWHVANLNQYVGLRYNQVPE
FKLGDQSLTALGQGTGLVGLPGDFTPASRFIRVAFLRDAMIKNDKDSIDLIEFFHILNNVAMVRGS
TRTVEEKSDLTQYTSCMCLEKGIYYYNTYENNQINAIDMNKENLDGNEIKTYKYNKTLSINHVN
>3DR3A
SNAMLNTLIVGASGYAGAELVTYVNRHPHMNITALTVSAQSNDAGKLISDLHPQLKGIVELPLQPM
SDISEFSPGVDVVFLATAHEVSHDLAPQFLEAGCVVFDLSGAFRVNDATFYEKYYGFTHQYPELLE
QAAYGLAEWCGNKLKEANLIAVPGCYPTAAQLALKPLIDADLLDLNQWPVINATSGVSGAGRKAAI
SNSFCEVSLQPYGVFTHRHQPEIATHLGADVIFTPHLGNFPRGILETITCRLKSGVTQAQVAQALQ
QAYAHKPLVRLYDKGVPALKNVVGLPFCDIGFAVQGEHLIIVATEDNLLKGAAAQAVQCANIRFGY
AETQSLI
>2CN3A
ISSQAVTSVPYKWDNVVIGGGGGFMPGIVFNETEKDLIYARAAIGGAYRWDPSTETWIPLLDHFQM
DEYSYYGVESIATDPVDPNRVYIVAGMYTNDWLPNMGAILRSTDRGETWEKTILPFKMGGNMPGRS
MGERLAIDPNDNRILYLGTRCGNGLWRSTDYGVTWSKVESFPNPGTYIYDPNFDYTKDIIGVVWVV
FDKSSSTPGNPTKTIYVGVADKNESIYRSTDGGVTWKAVPGQPKGLLPHHGVLASNGMLYITYGDT
CGPYDGNGKGQVWKFNTRTGEWIDITPIPYSSSDNRFCFAGLAVDRQNPDIIMVTSMNAWWPDEYI

FRSTDGGATWKNIWEWGMYPERILHYEIDISAAPWLDWGTEKQLPEINPKLGWMIGDIEIDPFNSD
RMMYVTGATIYGCDNLTDWDRGGKVKIEVKATGIEECAVLDLVSPPEGAPLVSAVGDLVGFVHDDL
KVGPKKMHVPSYSSGTGIDYAELVPNFMALVAKADLYDVKKISFSYDGGRNWFQPPNEAPNSVGGG
SVAVAADAKSVIWTPENASPAVTTDNGNSWKVCTNLGMGAVVASDRVNGKKFYAFYNGKFYISTDG
GLTFTDTKAPQLPKSVNKIKAVPGKEGHVWLAAREGGLWRSTDGGYTFEKLSNVDTAHVVGFGKAA
PGQDYMAIYITGKIDNVLGFFRSDDAGKTWVRINDDEHGYGAVDTAITGDPRVYGRVYIATNGRGI
VYGEPASDEPV
>1XA3A
MHHHHHHGSTSLYKKAGSETLYIQGDHLPMPKFGPLAGLRVVFSGIEIAGPFAGQMFAEWGAEVIW
IENVAWADTIRVQPNYPQLSRRNLHALSLNIFKDEGREAFLKLMETTDIFIEASKGPAFARRGITD
EVLWQHNPKLVIAHLSGFGQYGTEEYTNLPAYNTIAQAFSGYLIQNGDVDQPMPAFPYTADYFSGL
TATTAALAALHKVRETGKGESIDIAMYEVMLRMGQYFMMDYFNGGEMCPRMSKGKDPYYAGCGLYK
CADGYIVMELVGITQIEECFKDIGLAHLLGTPEIPEGTQLIHRIECPYGPLVEEKLDAWLATHTIA
EVKERFAELNIACAKVLTVPELESNPQYVARESITQWQTMDGRTCKGPNIMPKFKNNPGQIWRGMP
SHGMDTAAILKNIGYSENDIQELVSKGLAKVEDSTHHHHHH
>1GPIA
QQAGTNTAENHPQLQSQQCTTSGGCKPLSTKVVLDSNWRWVHSTSGYTNCYTGNEWDTSLCPDGKT
CAANCALDGADYSGTYGITSTGTALTLKFVTGSNVGSRVYLMADDTHYQLLKLLNQEFTFDVDMSN
LPCGLNGALYLSAMDADGGMSKYPGNKAGAKYGTGYCDSQCPKDIKFINGEANVGNWTETGSNTGT
GSYGTCCSEMDIWEANNDAAAFTPHPCTTTGQTRCSGDDCARNTGLCDGDGCDFNSFRMGDKTFLG
KGMTVDTSKPFTVVTQFLTNDNTSTGTLSEIRRIYIQNGKVIQNSVANIPGVDPVNSITDNFCAQQ
KTAFGDTNWFAQKGGLKQMGEALGNGMVLALSIWDDHAANMLWLDSDYPTDKDPSAPGVARGTCAT
TSGVPSDVESQVPNSQVVFSNIKFGDIGSTFSGTS
>1CMNA
ASGAPSFPFSRASGPEPPAEFAKLRATNPVSQVKLFDGSLAWLVTKHKDVCFVATSEKLSKVRTRQ
GFPELSASGKQAAKAKPTFVDMDPPEHMHQRSMVEPTFTPEAVKNLQPYIQRTVDDLLEQMKQKGC
ANGPVDLVKEFALPVPSYIIYTLLGVPFNDLEYLTQQNAIRTNGSSTAREASAANQELLDYLAILV
EQRLVEPKDDIISKLCTEQVKPGNIDKSDAVQIAFLLLVAGNATMVNMIALGVATLAQHPDQLAQL
KANPSLAPQFVEELCRYHTAVALAIKRTAKEDVMIGDKLVRANEGIIASNQSANRDEEVFENPDEF
NMNRKWPPQDPLGFGFGDHRCIAEHLAKAELTTVFSTLYQKFPDLKVAVPLGKINYTPLNRDVGIV
DLPVIF
>1LRIA
TACTASQQTAAYKTLVSILSDASFNQCSTDSGYSMLTAKALPTTAQYKLMCASTACNTMIKKIVTL
NPPNCDLTVPTSGLVLNVYSYANGFSNKCSSL
>2QECA
GMSPTVLPATQADFPKIVDVLVEAFANDPTFLRWIPQPDPGSAKLRALFELQIEKQYAVAGNIDVA
RDSEGEIVGVALWDRPDGNHSAKDQAAMLPRLVSIFGIKAAQVAWTDLSSARFHPKFPHWYLYTVA
TSSSARGTGVGSALLNHGIARAGDEAIYLEATSTRAAQLYNRLGFVPLGYIPSDDDGTPELAMWKP
PAMPTV
>1VAJA
MVFKIKDEWGEFLVRLARRAIEEYLKTGKEIEPPKDTPPELWEKMGVFVTLNRYNVPPQTALRGCI
GFPTPIYPLVEATIKAAIYSAVDDPRFPPVKLEEMDNLVVEVSVLTPPELIEGPPEERPRKIKVGR
DGLIVEKGIYSGLLLPQVPVEWGWDEEEFLAETCWKAGLPPDCWLDEDTKVYKFTAEIFEEEYPRG
PIKRKPLVLEHHHHHH

>1D7EA
VAFGSEDIENTLAKMDDGQLDGLAFGAIQLDGDGNILQYNAAEGDITGRDPKQVIGKNFFKDVAPC
TDSPEFYGKFKEGVASGNLNTMFEYTFDYQMTPTKVKVHMKKALSGDSYWVFVKRV
>1YNVX
DVSGTVCLSALPPEATDTLNLIASDGPFPYSQDGVVFQNRESVLPTQSYGYYHEYTVITPGARTRG
TRRIITGEATQEDYYTGDHYATFSLIDKTC
>1XH9A
GNAAAAKKGSEQESVKEFLAKAKEDFLKKWENPAQNTAHLDQFERIKTLGTGSFGRVMLVKHMETG
NHYAMKILDKQKVVKLKEIEHTLNEKRILQAVNFPFLVKLEFSFKDNSNLYMVMEYAPGGEMFSHL
RRIGRFSEPHARFYAAQIVLTFEYLHSLDLIYRDLKPENLMIDQQGYIKVTDFGLAKRVKGRTWTL
CGTPEYLAPEIILSKGYNKAVDWWALGVLIYEMAAGYPPFFADQPIQIYEKIVSGKVRFPSHFSSD
LKDLLRNLLQVDLTKRFGNLKNGVNDIKNHKWFATTDWIAIYQRKVEAPFIPKFKGPGDTSNFDDY
EEEEIRVSINEKCGKEFSEF
>3OIRA
SNADGLEGMDDPDATSKKVVPLGVEIYEINGPFFFGVADRLKGVLDVIEETPKVFILRMRRVPVID
ATGMHALWEFQESCEKRGTILLLSGVSDRLYGALNRFGFIEALGEERVFDHIDKALAYAKLLVETA
EER
>2V4CA
ADYDLKFGMNAGTSSNEYKAAEMFAKEVKEKSQGKIEISLYPSSQLGDDRAMLKQLKDGSLDFTFA
ESARFQLFYPEAAVFALPYVISNYNVAQKALFDTEFGKDLIKKMDKDLGVTLLSQAYNGTRQTTSN
RAINSIADMKGLKLRVPNAATNLAYAKYVGASPTPMAFSEVYLALQTNAVDGQENPLAAVQAQKFY
EVQKFLAMTNHILNDQLYLVSNETYKELPEDLQKVVKDAAENAAKYHTKLFVDGEKDLVTFFEKQG
VKITHPDLVPFKESMKPYYAEFVKQTGQKGESALKQIEAINPHHH
>1JNRA
MVYYPKKYELYKADEVPTEVVETDILIIGGGFSGCGAAYEAAYWAKLGGLKVTLVEKAAVERSGAV
AQGLSAINTYIDLTGRSERQNTLEDYVRYVTLDMMGLAREDLVADYARHVDGTVHLFEKWGLPIWK
TPDGKYVREGQWQIMIHGESYKPIIAEAAKMAVGEENIYERVFIFELLKDNNDPNAVAGAVGFSVR
EPKFYVFKAKAVILATGGATLLFRPRSTGEAAGRTWYAIFDTGSGYYMGLKAGAMLTQFEHRFIPF
RFKDGYGPVGAWFLFFKCKAKNAYGEEYIKTRAAELEKYKPYGAAQPIPTPLRNHQVMLEIMDGNQ
PIYMHTEEALAELAGGDKKKLKHIYEEAFEDFLDMTVSQALLWACQNIDPQEQPSEAAPAEPYIMG
SHSGEAGFWVCGPEDLMPEEYAKLFPLKYNRMTTVKGLFAIGDCAGANPHKFSSGSFTEGRIAAKA
AVRFILEQKPNPEIDDAVVEELKKKAYAPMERFMQYKDLSTADDVNPEYILPWQGLVRLQKIMDEY
AAGIATIYKTNEKMLQRALELLAFLKEDLEKLAARDLHELMRAWELVHRVWTAEAHVRHMLFRKET
RWPGYYYRTDYPELNDEEWKCFVCSKYDAEKDEWTFEKVPYVQVIEWSF
>2QM0A
SNAMNTTVEKQQIITSNTEQWKMYSKLEGKEYQIHISKPKQPAPDSGYPVIYVLDGNAFFQTFHEA
VKIQSVRAEKTGVSPAIIVGVGYPIEGAFSGEERCYDFTPSVISKDAPLKPDGKPWPKTGGAHNFF
TFIEEELKPQIEKNFEIDKGKQTLFGHXLGGLFALHILFTNLNAFQNYFISSPSIWWNNKSVLEKE
ENLIIELNNAKFETGVFLTVGSLEREHMVVGANELSERLLQVNHDKLKFKFYEAEGENHASVVPTS
LSKGLRFISYV
>2QM6A
MGSSHHHHHHSSGLVPRGSHMASAASYPPIKNTKVGLALSSHPLASEIGQKVLEEGGNAIDAAVAI
GFALAVVHPAAGNIGGGGFAVIHLANGENVALDFREKAPLKATKNMFLDKQGNVVPKLSEDGYLAA
GVPGTVAGMEAMLKKYGTKKLSQLIDPAIKLAENGYAISQRQAETLKEARERFLKYSSSKKYFFKK

GHLDYQEGDLFVQKDLAKTLNQIKTLGAKGFYQGQVAELIEKDMKKNGGIITKEDLASYNVKWRKP
VVGSYRGYKIISMSPPSSGGTHLIQILNVMENADLSALGYGASKNIHIAAEAMRQAYADRSVYMGD
ADFVSVPVDKLINKAYAKKIFDTIQPDTVTPSSQIKPGMGQLHEGSN
>3GHJA
MGSSHHHHHHSSGRENLYFQGVPMNIKGLFEVAVKVKNLEKSSQFYTEILGFEAGLLDSARRWNFL
WVSGRAGMVVLQEEKENWQQQHFSFRVEKSEIEPLKKALESKGVSVHGPVNQEWMQAVSLYFADPN
GHALEFTAL
>2Z58B
NTIRVIVSVDKAKFNPHEVLGIGGHIVYQFKLIPAVVVDVPANAVGKLKKMPWVEKVEFDHQAVLL
>1CUHA
LPTSNPAQELEARQLGRTTRDDLINGNSASCADVIFIYARGSTETGNLGTLGPSIASNLESAFGKD
GVWIQGVGGAYRATLGDNALPRGTSSAAIREMLGLFQQANTKCPDATLIAGGYSQGAALAAASIED
LDSAIRDKIAGTVLFGYTKNLQNRGRIPNYPADRTKVFCNTGDLVCTGSLIVAAPHLAYGPDAEGP
APEFLIEKVRAVRGSA
>1OJQA
AETKNFTDLVEATKWGNSLIKSAKYSSKDKMAIYNYTKNSSPINTPLRSANGDVNKLSENIQEQVR
QLDSTISKSVTPDSVYVYRLLNLDYLSSITGFTREDLHMLQQTNNGQYNEALVSKLNNLMNSRIYR
ENGYSSTQLVSGAALAGRPIELKLELPKGTKAAYIDSKELTAYPGQQEVLLPRGTEYAVGSVKLSD
NKRKIIITAVVFKK
>1NIJA
MNPIAVTLLTGFLGAGKTTLLRHILNEQHGYKIAVIENEFGEVSVDDQLIGDRATQIKTLTNGCIC
CSRSNELEDALLDLLDNLDKGNIQFDRLVIECTGMADPGPIIQTFFSHEVLCQRYLLDGVIALVDA
VHADEQMNQFTIAQSQVGYADRILLTKTDVAGEAEKLHERLARINARAPVYTVTHGDIDLGLLFNT
NGFMLEENVVSTKPRFHFIADKQNDISSIVVELDYPVDISEVSRVMENLLLESADKLLRYKGMLWI
DGEPNRLLFQGVQRLYSADWDRPWGDEKPHSTMVFIGIQLPEEEIRAAFAGLRK
>2O2GA
GMDRTLTHQPQEYAVSVSVGEVKLKGNLVIPNGATGIVLFAHGSGSSRYSPRNRYVAEVLQQAGLA
TLLIDLLTQEEEEIDLRTRHLRFDIGLLASRLVGATDWLTHNPDTQHLKVGYFGASTGGGAALVAA
AERPETVQAVVSRGGRPDLAPSALPHVKAPTLLIVGGYDLPVIAMNEDALEQLQTSKRLVIIPRAS
HLFEEPGALTAVAQLASEWFMHYLR
>1O2DA
MGSDKIHHHHHHVWEFYMPTDVFFGEKILEKRGNIIDLLGKRALVVTGKSSSKKNGSLDDLKKLLD
ETEISYEIFDEVEENPSFDNVMKAVERYRNDSFDFVVGLGGGSPMDFAKAVAVLLKEKDLSVEDLY
DREKVKHWLPVVEIPTTAGTGSEVTPYSILTDPEGNKRGCTLMFPVYAFLDPRYTYSMSDELTLST
GVDALSHAVEGYLSRKSTPPSDALAIEAMKIIHRNLPKAIEGNREARKKMFVASCLAGMVIAQTGT
TLAHALGYPLTTEKGIKHGKATGMVLPFVMEVMKEEIPEKVDTVNHIFGGSLLKFLKELGLYEKVA
VSSEELEKWVEKGSRAKHLKNTPGTFTPEKIRNIYREALGV
>3MR1A
GPGSMIKIHTEKDFIKMRAAGKLAAETLDFITDHVKPNVTTNSLNDLCHNFITSHNAIPAPLNYKG
FPKSICTSINHVVCHGIPNDKPLKNGDIVNIDVTVILDGWYGDTSRMYYVGDVAIKPKRLIQVTYD
AMMKGIEVVRPGAKLGDIGYAIQSYAEKHNYSVVRDYTGHGIGRVFHDKPSILNYGRNGTGLTLKE
GMFFTVEPMINAGNYDTILSKLDGWTVTTRDKSLSAQFEHTIGVTKDGFEIFTL
>3NJCA
MGHHHHHHSHMKSKFEASIDNLKEIEMNAYAYELIREIVLPDMLGQDYSSMMYWAGKHLARKFPLE

SWEEFPAFFEEAGWGTLTNVSAKKQELEFELEGPIISNRLKHQKEPCFQLEAGFIAEQIQLMNDQI
AESYEQVKKRADKVVLTVKWDMKDPV
>3R0NA
MQDVRVQVLPEVRGQLGGTVELPCHLLPPVPGLYISLVTWQRPDAPANHQNVAAFHPKMGPSFPSP
KPGSERLSFVSAKQSTGQDTEAELQDATLALHGLTVEDEGNYTCEFATFPKGSVRGMTWLRV
>1FPZA
MKPPSSIQTSEFDSSDEEPIEDEQTPIHISWLSLSRVNCSQFLGLCALPGCKFKDVRRNVQKDTEE
LKSCGIQDIFVFCTRGELSKYRVPNLLDLYQQCGIITHHHPIADGGTPDIASCCEIMEELTTCLKN
YRKTLIHSYGGLGRSCLVAACLLLYLSDTISPEQAIDSLRDLRGSGAIQTIKQYNYLHEFRDKLAA
HLSSRDSQSRSVSR
>3LX4A
MGSSHHHHHHSQDPNSAAPAAEAPLSHVQQALAELAKPKDDPTRKHVCVQVAPAVRVAIAETLGLA
PGATTPKQLAEGLRRLGFDEVFDTLFGADLTIMEEGSELLHRLTEHLEAHPHSDEPLPMFTSCCPG
WIAMLEKSYPDLIPYVSSCKSPQMMLAAMVKSYLAEKKGIAPKDMVMVSIMPCTRKQSEADRDWFC
VDADPTLRQLDHVITTVELGNIFKERGINLAELPEGEWDNPMGVGSGAGVLFGTTGGVMEAALRTA
YELFTGTPLPRLSLSEVRGMDGIKETNITMVPAPGSKFEELLKHRAAARAEAAAHGTPGPLAWDGG
AGFTSEDGRGGITLRVAVANGLGNAKKLITKMQAGEAKYDFVEIMACPAGCVGGGGQPRSTDKAIT
QKRQAALYNLDEKSTLRRSHENPSIRELYDTYLGEPLGHKAHELLHTHYVAGGVEEKDEKK
>1SCTB
SKVAELANAVVSNADQKDLLRMSWGVLSVDMEGTGLMLMANLFKTSPSAKGKFARLGDVSAGKDNS
KLRGHSITLMYALQNFVDALDDVERLKCVVEKFAVNHINRQISADEFGEIVGPLRQTLKARMGNYF
DEDTVAAWASLVAVVQASL
>3M1MA
SSNFSSERIRYAKWFLEHGFNIIPIDPESKKPVLKEWQKYSHEMPSDEEKQRFLKMIEEGYNYAIP
GGQKGLVILDFESKEKLKAWIGESALEELCRKTLCTNTVHGGIHIYVLSNDIPPHKINPLFEENGK
GIIDLQSYNSYVLGLGSCVNHLHCTTDKCPWKEQNYTTCYTLYNELKEISKVDLKSLLRFLAEKGK
RLGITLSKTAKEWLEGKKEEEDTVVEFEELRKELVKRDSGKPVEKIKEEICTKSPPKLIKEIICEN
KTYADVNIDRSRGDWHVILYLMKHGVTDPDKILELLPRDSKAKENEKWNTQKYFVITLSKAWSVVK
KYLEA
>3M12A
STHFDVIVVGAGSMGMAAGYQLAKQGVKTLLVDAFDPPHTNGSHHGDTRIIRHAYGEGREYVPLAL
RSQELWYELEKETHHKIFTKTGVLVFGPKGESAFVAETMEAAKEHSLTVDLLEGDEINKRWPGITV
PENYNAIFEPNSGVLFSENCIRAYRELAEARGAKVLTHTRVEDFDISPDSVKIETANGSYTADKLI
VSMGAWNSKLLSKLNLDIPLQPYRQVVGFFESDESKYSNDIDFPGFMVEVPNGIYYGFPSFGGCGL
RLGYHTFGQKIDPDTINREFGVYPEDESNLRAFLEEYMPGANGELKRGAVCMYTKTLDEHFIIDLH
PEHSNVVIAAGFSGHGFKFSSGVGEVLSQLALTGKTEHDISIFSINRPALKESLQKTTI
>3BDZA
TSLFTTADHYHTPLGPDGTPHAFFEALRDEAETTPIGWSEAYGGHWVVAGYKEIQAVIQNTKAFSN
KGVTFPRYETGEFELMMAGQDDPVHKKYRQLVAKPFSPEATDLFTEQLRQSTNDLIDARIELGEGD
AATWLANEIPARLTAILLGLPPEDGDTYRRWVWAITHVENPEEGAEIFAELVAHARTLIAERRTNP
GNDIMSRVIMSKIDGESLSEDDLIGFFTILLLGGIDATARFLSSVFWRLAWDIELRRRLIAHPELI
PNAVDELLRFYGPAMVGRLVTQEVTVGDITMKPGQTAMLWFPIASRDRSAFDSPDNIVIERTPNRH
LSLGHGIHRCLGAHLIRVEARVAITEFLKRIPEFSLDPNKECEWLMGQVAGMLHVPIIFPKGKRLS
E

>1FY3A
IVGGRKARPRQFPFLASIQNQGRHFCGGALIHARFVMTAASCFQSQNPGVSTVVLGAYDLRRRERQ
SRQTFSISSMSENGYDPQQNLNDLMLLQLDREANLTSSVTILPLPLQNATVEAGTRCQVAGWGSQR
SGGRLSRFPRFVNVTVTPEDQCRPNNVCTGVLTRRGGICNGDQGTPLVCEGLAHGVASFSLGPCGR
GPDFFTRVALFRDWIDGVLNNPGPGPA
>2VR3A
MRGSHHHHHHGSGTDITNQLTNVTVGIDSGTTVYPHQAGYVKLNYGFSVPNSAVKGDTFKITVPKE
LNLNGVTSTAKVPPIMAGDQVLANGVIDSDGNVIYTFTDYVNTKCDVKATLTMPAYIDPENVKKTG
NVTLATGIGSTTANKTVLVDYEKYGKFYNLSIKGTIDQIDKTNNTYRQTIYVNPSGDNVIAPVLTG
NLKPNTDSNALIDQQNTSIKVYKVDNAADLSESYFVNPENFEDVTNSVNITFPNPNQYKVEFNTPD
DQITTPYIVVVNGHIDPNSKGDLALRSTLYGYNSNIIWRSMSWDNEVAFNNGSGSGDGIDCPVVP
>1R8ME
LEANEGSKTLQRNRKMAMGRKKFNMDPKKGIQFLVENELLQNTPEEIARFLYKGEGLNKTAIGDYL
GEREELNLAVLHAFVDLHEFTDLNLVQALRQFLWSFRLPGEAQKIDRMMEAFAQRYCLCNPGVFQS
TDTCYVLSYSVIMLNTDLHNPNVRDKMGLERFVAMNRGINEGGDLPEELLRNLYDSIRNEPFKIPE
DDGND
>1BYPA
AEVLLGSSDGGLAFVPSDLSIASGEKITFKNNAGFPHNDLFDKKEVPAGVDVTKISMPEEDLLNAP
GEEYSVTLTEKGTYKFYCAPHAGAGMVGKVTVN
>3D0KA
SNAMKPADLTNADRIALELGHAGRNAIPYLDDDRNADRPFTLNTYRPYGYTPDRPVVVVQHGVLRN
GADYRDFWIPAADRHKLLIVAPTFSDEIWPGVESYNNGRAFTAAGNPRHVDGWTYALVARVLANIR
AAEIADCEQVYLFGHSAGGQFVHRLMSSQPHAPFHAVTAANPGWYTLPTFEHRFPEGLDGVGLTED
HLARLLAYPMTILAGDQDIATDDPNLPSEPAALRQGPHRYARARHYYEAGQRAAAQRGLPFGWQLQ
VVPGIGHDGQAMSQVCASLWFDGRMPDAAELARLAGSQSA
>1ZHVA
APRIKLKILNGSYGIARLSASEAIPAWADGGGFVSITRTDDELSIVCLIDRIPQDVRVDPGWSCFK
FQGPFAFDETGIVLSVISPLSTNGIGIFVVSTFDGDHLLVRSNDLEKTADLLANAGHSLLLEHHHH
HH
>3H3JA
MNKFKGNKVVLIGNGAVGSSYAFSLVNQSIVDELVIIDLDTEKVRGDVMDLKHATPYSPTTVRVKA
GEYSDCHDADLVVICAGARQKPGETRLDLVSKNLKIFKSIVGEVMASKFDGIFLVATNPVDILAYA
TWKFSGLPKERVIGSGTILDSARFRLLLSEAFDVAPRSVDAQIIGEHGDTELPVWSHANIAGQPLK
TLLEQRPEGKAQIEQIFVQTRDAAYDIIQAKGATYYGVAMGLARITEAIFRNEDAVLTVSALLEGE
YEEEDVYIGVPAVINRNGIRNVVEIPLNDEEQSKFAHSAKTLKDIMAEAEELK
>3H36A
SNAVELLQVDADLQAEIVGKYNADLQKAVQIEEKKASEIATEAVKEHVTAEYEERYAEHEEHDRIM
RDVAEILEQMEHAEVRRLITEDKVRPD
>1Z3EB
MEKEKVLEMTIEELDLSVRSYNCLKRAGINTVQELANKTEEDMMKVRNLGRKSLEEVKAKLEELGL
GLRKDDG
>3KFFA
EEATSKGQNLNVEKINGEWFSILLASDKREKIEEHGSMRVFVEHIHVLENSLAFKFHTVIDGECSE
IFLVADKTEKAGEYSVMYDGFNTFTILKTDYDNYIMFHLINEKDGKTFQLMELYGRKADLNSDIKE

KFVKLCEEHGIIKENIIDLTKTNRCLKARE
>2RB8A
MRLDAPSQIEVKDVTDTTALITWMPPSQPVDGFELTYGIKDVPGDRTTIDLTEDENQYSIGNLKPD
TEYEVSLISRRGDMSSNPAKETFTTGLAAALEHHHHHH
>2RBDA
GMGILSGNPQDEPLHYGEVFSTWTYLSTNNGLINGYRSFINHTGDEDLKNLIDEAIQAMQDENHQL
EELLRSNGVGLPPAPPDRPAARLDDIPVGARFNDPEISATISMDVAKGLVTCSQIIGQSIREDVAL
MFSQFHMAKVQFGGKMLKLNKNKGWLIPPPLHSDRPIKE
