# Supplementary material for: iDNA-Prot: Identification of DNA Binding Proteins Using Random Forest with Grey Model
Source: PLoS One. 2011 Sep 15;6(9):e24756. doi: 10.1371/journal.pone.0024756 (PMC3174210; doi:10.1371/journal.pone.0024756)
Supplement: Information S2 — List of protein codes that occur in both the training and testing datasets for DNA-Prot (Kumar et al., 2009). See the main paper for further explanation. (PDF) [file pone.0024756.s002.pdf]

Online Supporting Information S2. List of protein codes that occur in both the training and testing datasets for **DNA-Prot** (Kumar et al., 2009). See the main paper for further explanation.

(a) The 10 DNA-binding protein sequences that occur in both the training dataset S_{tran} and the testing dataset S^1_{test}

Training dataset S_{tran}		Testing dataset S^1_{test}	
No.[a]	ID[b]	No.[a]	ID[b]
2	D1A0AA_	1	1A0A:A
23	D1DCTA_	10	1DCT:A
24	D1DFMA_	11	1DFM:A
39	D1FIUA_	20	1FIU:A
56	D1J1VA_	32	1J1V:A
94	D1OMHA_	60	1OSB:A
99	D1QRVA_	67	1QRV:A
110	D1RH6A_	72	1RH6:A
118	D1SA3A_	74	1SA3:A
140	D2DNJA_	91	2DNJ:A

[a] See (Kumar et al., 2009) for the definition of No. used here.

[b] See (Kumar et al., 2009) for the definition of ID used here.

(b) The 19 DNA-binding protein sequences that occur in both the training dataset S_{tran} and the testing dataset S^3_{test}

Training dataset S_{tran}		Testing dataset S^3_{test}	
No.[a]	ID[b]	No.[a]	ID[b]

5	D1A73A_	4	1a73A
8	D1AZPA_	6	1azpA
9	D1B3TA_	7	1b3tA
16	D1CF7A_	17	1cf7A
19	D1D02A_	24	1d02A
23	D1DCTA_	26	1dctA
25	D1DMUA_	29	1dmuA
26	D1DP7P_	30	1dp7P
30	D1ECRA_	31	1ecrA
40	D1FJLA_	34	1fjlA
44	D1G9ZA	14	1bp7A
48	D1HCRA_	41	1hcrA
80	D1LMB3_	49	1lmb3
98	D1PDNC_	55	1pdnC
18	D1CKQA_	62	1qpsA
99	D1QRVA_	63	1qrvA
120	D1SKNP_	67	1sknP
123	D1TC3C_	71	1tc3C
139	D2BOPA_	81	2bopA

[a] See (Kumar et al., 2009) for the definition of No. used here.

[b] See (Kumar et al., 2009) for the definition of ID used here.

(c) List of the PDB codes for the 94 non-DNA-binding protein sequences that occur in both the training dataset S_{tran} and the testing dataset S^{3}_{test}

1A53	1A8E	1AC5	1AH7	1AHO	1AJJ	1ALY
1AMM	1AMP	1ATG	1AXN	1AAC	1AOL	1BD8
1BDO	1BEO	1BHE	1BHP	1BPI	1BQK	1BS9
1BTN	1BV1	1C52	1CA1	1CEM	1CFB	1CPQ
1CPT	1CSH	1CTJ	1CTT	1DFX	1DHN	1DOI
1DPE	1EAF	1ECY	1EDG	1FCE	1FKJ	1FRB
1GAI	1GCA	1GOF	1HKA	1HOE	1HTP	1IAE
1INP	1IOV	1KLO	1KOE	1KTE	1KUH	1LAM
1LBU	1LID	1LST	1MRP	1NFP	1NG1	1NLS
1NNC	1NSJ	1OPR	1OPS	1OSA	1PBV	1PHP
1PLC	1PMI	1POA	1POC	1PPN	1PRN	1RA9
1RCB	1RMG	1RSY	1RZL	1SKF	1SRA	1SVB
1TCA	1TDE	1TEN	1THV	1TML	1TN3	1TRY
1UOK	1UTG	1VLS				

REFERENCE

Kumar, K.K., Pugalenthi, G., and Suganthan, P.N., 2009. DNA-Prot: identification of DNA binding proteins from protein sequence information using random forest. J Biomol Struct Dyn 26, 679-86.
