# Supplementary material for: iDNA-Prot: Identification of DNA Binding Proteins Using Random Forest with Grey Model
Source: PLoS One. 2011 Sep 15;6(9):e24756. doi: 10.1371/journal.pone.0024756 (PMC3174210; doi:10.1371/journal.pone.0024756)
Supplement: Information S3 — A step-by-step guide on how to use the web-server of iDNA-Prot to get the desired results. (PDF) [file pone.0024756.s003.pdf]

Online Supporting Information S3. A protocol guide for **iDNA-Prot** web-server.

For the convenience of the vast majority of experimental scientists, here let us give a step-by-step guide on how to use the web-server to get the desired results without the need to follow the complicated mathematic equations involved in developing the **iDNA-Prot** predictor.

Step 1. Open the web server at site http://icpr.jci.edu.cn/bioinfo/iDNA-Prot or http://www.jci-bioinfo.cn/iDNA-Prot and you will see the top page of the predictor on your computer screen, as shown in **Fig.S1**. Click on the Read Me button to see a brief introduction about **iDNA-Prot** predictor and the caveat when using it.

Step 2. Either type or copy and paste the query protein sequence into the input box at the center of **Fig.S1**. The input sequence should be in the FASTA format. A sequence in FASTA format consists of a single initial line beginning with a greater-than symbol (“>”) in the first column, followed by lines of sequence data. The words right after the “>” symbol in the single initial line are optional and only used for the purpose of identification and description. All lines have no limitation in the number of characters. The sequence ends if another line starting with a “>” appears; this indicates the start of another sequence. Example sequences in FASTA format can be seen by clicking on the Example button right above the input box. For more information about FASTA format, visit http://en.wikipedia.org/wiki/Fasta_format. As shown in **Fig.S1**, the maximum number of query proteins for each submission is 50.

Step 3. Click on the Submit button to see the predicted result. For example, if you use the three query protein sequences in the Example window as the input as shown in **Fig.S2**, after clicking the Submit button, you will see **Fig.S3** shown on your screen, indicating that the predicted result for the 1st query protein is “**DNA-binding protein**”, that for the 2nd one is “**DNA-binding protein**”, and that for the 3rd one is “**non DNA-binding protein**”. All these results are fully consistent with the experimental observation as summarized in the Online Supporting Information S1. It takes about a few seconds for the above computation before the predicted result appears on your computer screen; the more number of query proteins, the more time it is usually needed.

Step 4. Click on the Citation button to find the relevant paper that documents the detailed development and algorithm of **iDNA-Prot**.

Step 5. Click on the Data button to download the benchmark datasets used to train and test the **iDNA-Prot** predictor.

Caveat. To obtain the predicted result with the expected success rate, the entire sequence of the query protein rather than its fragment should be used as an input. A sequence with less than 50 amino acid residues is generally deemed as a fragment. Also, a sequence with more than 10 consecutive character of “X” is not a valid input because containing too many unknown amino acids.

FIGURE LEGENDS

Figure S1. A semi-screenshot to show the top page of the iDNA-Prot web-server. Its website address is at http://icpr.jci.edu.cn/bioinfo/iDNA-Prot/.

Figure S2. A semi-screenshot to show the input of iDNA-Prot. The input was taken from the three protein sequences listed in the Example window of the **iDNA-Prot** web-server (cf. **Fig.S1**).

Figure S3. A semi-screenshot to show the output generated by iDNA-Prot.

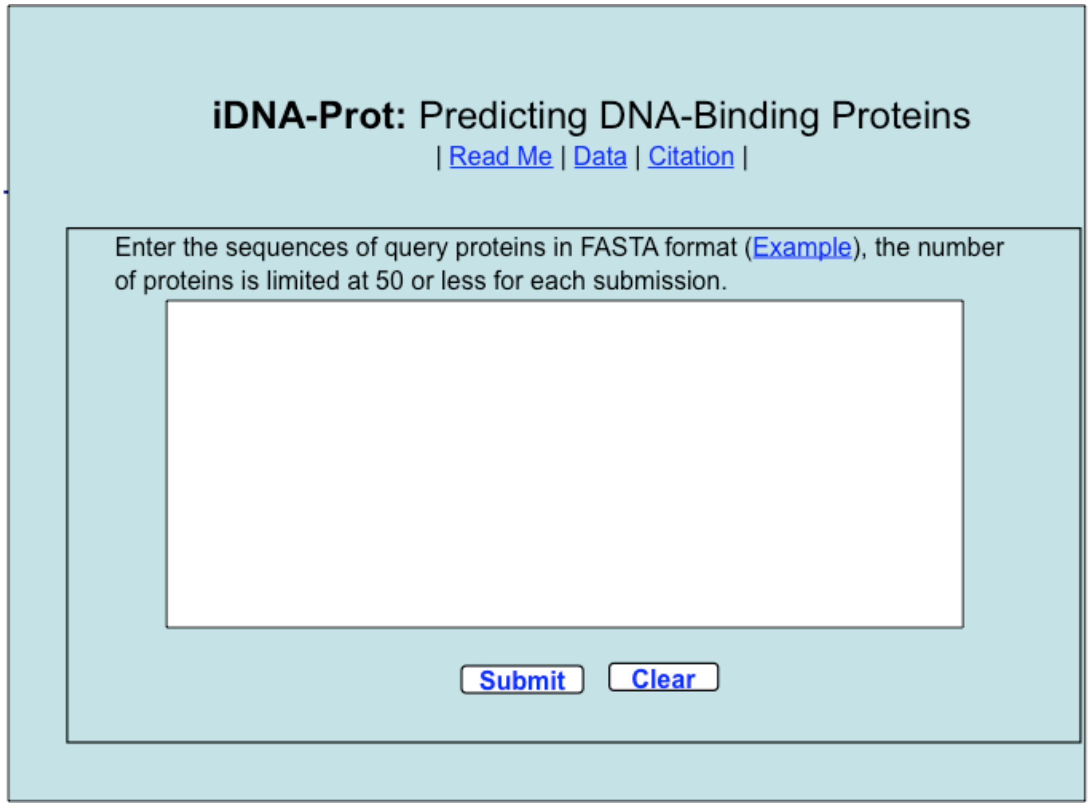
iDNA-Prot: Predicting DNA-Binding Proteins
| Read Me | Data | Citation |
Enter the sequences of query proteins in FASTA format (Example), the number of proteins is limited at 50 or less for each submission.
Submit
Clear

Figure S1

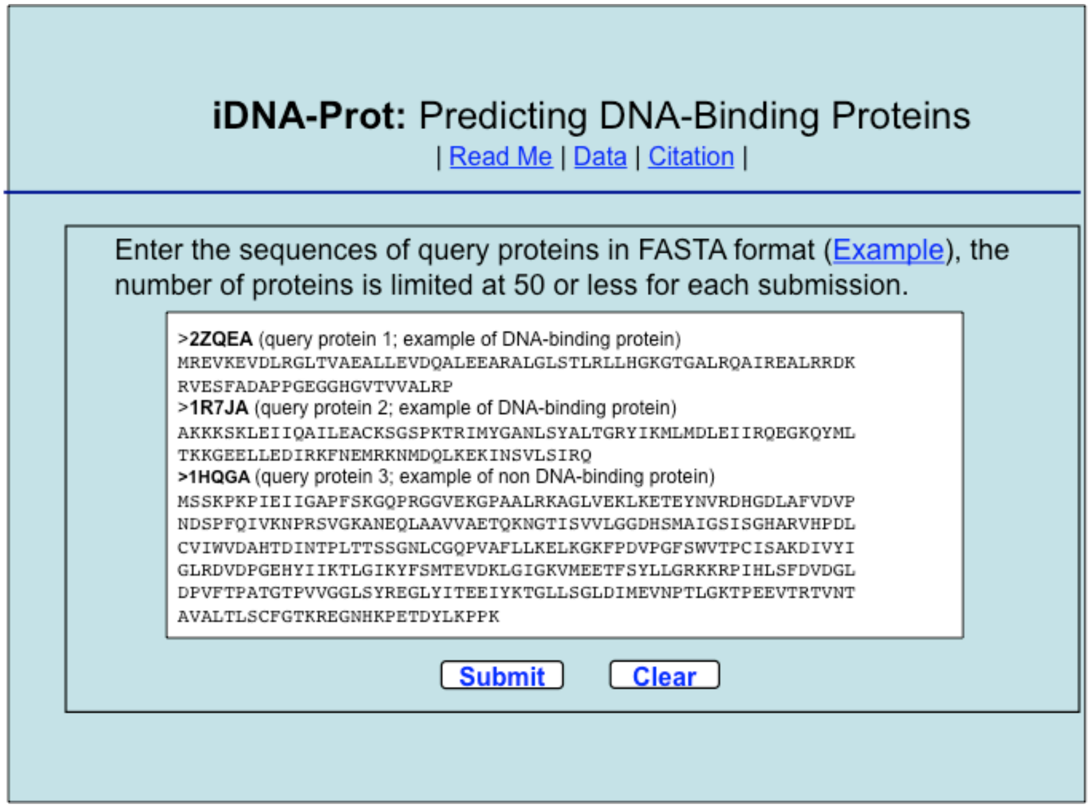
iDNA-Prot: Predicting DNA-Binding Proteins
| Read Me | Data | Citation |
Enter the sequences of query proteins in FASTA format (Example), the number of proteins is limited at 50 or less for each submission.
>2ZQEA (query protein 1; example of DNA-binding protein)
MREVKEVDLRGLTVAEALLEVDQALEEARALGLSTLRLLHGKGTGALRQAIREALRRDK
RVESFADAPPGEGGHGVTVVALRP
>1R7JA (query protein 2; example of DNA-binding protein)
AKKKSKLEIIQAILEACKSGSPKTRIMYGANLSYALTGRYIKMLMDLEIIRQEGKQYML
TKKGEELLEDIRKFNEMRKNMDQLKEKINSVLSIRQ
>1HQGA (query protein 3; example of non DNA-binding protein)
MSSKPKPIEIIGAPFSKGQPRGGVEKGPAALRKAGLVEKLKETEYNVRDHGDLAFVDVP
NDSPFQIVKNPRSVGKANEQLAAVVAETQKNGTISVVLGGDHSMAIGSISGHARVHPDL
CVIWVDAHTDINTPLTTSSGNLCGQPVAFLLKELKGKFPDVPGFSWVTPCISAKDIVYI
GLRDVDPGEHYIIKTLGIKYFSMTEVDKLGIGKVMEETFSYLLGRKKRPIHLSFDVDGL
DPVFTPATGTPVVGGLSYREGLYITEEIYKTGLLSGLDIMEVNPTLGKTPEEVTRTVNT
AVALTLSCFGTKREGNHKPETDYLKPPK
Submit
Clear

Figure S2

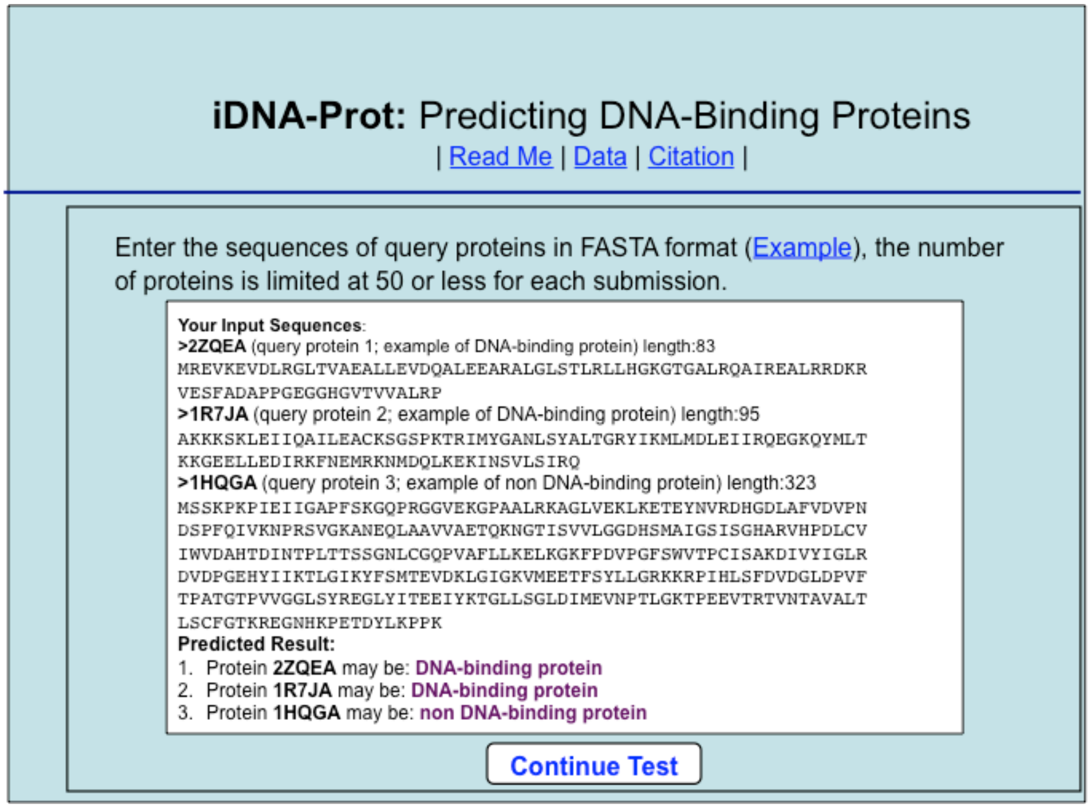
iDNA-Prot: Predicting DNA-Binding Proteins
| Read Me | Data | Citation |
Enter the sequences of query proteins in FASTA format (Example), the number of proteins is limited at 50 or less for each submission.
Your Input Sequences:
>2ZQEA (query protein 1; example of DNA-binding protein) length:83
MREVKEVDLRGLTVAEALLEVDQALEEARALGLSTLRLLHGKGTGALRQAIREALRRDKR
VESFADAPPGEGGHGVTVVALRP
>1R7JA (query protein 2; example of DNA-binding protein) length:95
AKKKSKLEIIQAILEACKSGSPKTRIMYGANLSYALTGRYIKMLMDLEIIRQEGKQYMLT
KKGEELLEDIRKFNEMRKNMDQLKEKINSVLSIRQ
>1HQGA (query protein 3; example of non DNA-binding protein) length:323
MSSKPKPIEIIGAPFSKGQPRGGVEKGPAALRKAGLVEKLKETEYNVRDHGDLAFVDVPN
DSPFQIVKNPRSVGKANEQLAAVVAETQKNGTISVVLGGDHSMAIGSISGHARVHPDLCV
IWVDAHTDINTPLTTSSGNLCGQPVAFLLKELKGKFPDVPGFSWVTPCISAKDIVYIGLR
DVDPGEHYIIKTLGIKYFSMTEVDKLGIGKVMEETFSYLLGRKKRPIHLSFDVDGLDPVF
TPATGTPVVGGLSYREGLYITEEIYKTGLLSGLDIMEVNPTLGKTPEEVTRTVNTAVALT
LSCFGTKREGNHKPETDYLKPPK
Predicted Result:
1. Protein 2ZQEA may be: DNA-binding protein
2. Protein 1R7JA may be: DNA-binding protein
3. Protein 1HQGA may be: non DNA-binding protein
Continue Test

Figure S3
