# Supplementary material for: iDNA-Prot: Identification of DNA Binding Proteins Using Random Forest with Grey Model
Source: PLoS One. 2011 Sep 15;6(9):e24756. doi: 10.1371/journal.pone.0024756 (PMC3174210; doi:10.1371/journal.pone.0024756)
Supplement: Information S4 — The independent dataset includes 244 proteins, classified into 122 DNA-binding proteins and 122 non DNA-binding proteins. Both the accession identifier of PDB (Protein Data Bank) and sequences are given. None of the proteins has more than 40% sequence identity to any other in a same subset. See the text of the paper for further explanation. (PDF) [file pone.0024756.s004.pdf]

**Online Supporting Information S4**. The independent dataset $\mathbb{S}^{\mathrm{Ind}}$ includes 244 proteins, classified into 122 DNA-binding proteins and 122 non DNA-binding proteins. Both the accession identifier of PDB (Protein Data Bank) and sequences are given. None of the proteins included here has more than 40% sequence identity to any other in a same subset. See the text of the paper for further explanation.

---

(1) 122 DNA-binding proteins

```
>1A02N
MRGSHHHHHHTDPHASSVPLEWPLSSQSGSYELRIEVQPKPHHRAHYETEGSRGAVKAPTGGHPVV
QLHGYMENKPLGLQIFIGTADERILKPHAFYQVHRITGKTVTTTSYEKIVGNTKVLEIPLEPKNNM
RATIDCAGILKLRNADIELRKGETDIGRKNTRVRLVFRVHIPESSGRIVSLQTASNPIECSQRSAH
ELPMVERQDTDSCLVYGGQQMILTGQNFTSESKVVFTEKTTDGQQIWEMEATVDKDKSQPNMLFVE
IPEYRNKHIRTPVKVNFYVINGKRKRSQPQHFTYHPV
>1A0PA
QDLARIEQFLDALWLEKNLAENTLNAYRRDLSMMVEWLHHRGLTLATAQSDDLQALLAERLEGGYK
ATSSARLLSAVRRLFQYLYREKFREDDPSAHLASPKLPQRLPKDLSEAQVERLLQAPLIDQPLELR
DKAMLEVLYATGLRVSELVGLTMSDISLRQGVVRVIGKGNKERLVPLGEEAVYWLETYLEHGRPWL
LNGVSIDVLFPSQRAQQMTRQTFWHRIKHYAVLAGIDSEKLSPHVLRHAFATHLLNHGADLRVVQM
LLGHSDLSTTQIYTHVATERLRQLHQ
>1AKHA
KKEKSPKGKSSISPQARAFLEEVFRRKQSLNSKEKEEVAKKCGITPLQVRVWFINKRMRSK
>1BDXA
MIGRLRGIIIEKQPPLVLIEVGGVGYEVHMPMTCFYELPEAGQEAIVFTHFVVREDAQLLYGFNNK
QERTLFKELIKTNGVGPKLALAILSGMSAQQFVNAVEREEVGALVKLPGIGKKTAERLIVEMKDRF
KGLHGDLFTPAADLVLTSPASPATDDAEQEAVAALVALGYKPQEASRMVSKIARPDASSETLIREA
LRAAL
>1BG1A
GQANHPTAAVVTEKQQMLEQHLQDVRKRVQDLEQKMKVVENLQDDFDFNYKTLKSQGDMQDLNGNN
QSVTRQKMQQLEQMLTALDQMRRSIVSELAGLLSAMEYVQKTLTDEELADWKRRQQIACIGGPPNI
CLDRLENWITSLAESQLQTRQQIKKLEELQQKVSYKGDPIVQHRPMLEERIVELFRNLMKSAFVVE
RQPCMPMHPDRPLVIKTGVQFTTKVRLLVKFPELNYQLKIKVCIDKDSGDVAALRGSRKFNILGTN
TKVMNMEESNNGSLSAEFKHLTLREQRCGNGGRANCDASLIVTEELHLITFETEVYHQGLKIDLET
HSLPVVVISNICQMPNAWASILWYNMLTNNPKNVNFFTKPPIGTWDQVAEVLSWQFSSTTKRGLSI
EQLTTLAEKLLGPGVNYSGCQITWAKFCKENMAGKGFSFWVWLDNIIDLVKKYILALWNEGYIMGF
ISKERERAILSTKPPGTFLLRFSESSKEGGVTFTWVEKDISGSTQIQSVEPYTKQQLNNMSFAEII
MGYKIMDATNILVSPLVYLYPDIPKEEAFGKYCRPESQEHPEADPGSAAPYLKTKFICVTPFIDAV
WK
>1C9BA
SDRAMMNAFKEITTMADRINLPRNIVDRTNNLFKQVYEQKSLKGRANDAIASACLYIACRQEGVPR
TFKEICAVSRISKKEIGRCFKLILKALETSVDLITTGDFMSRFCSNLCLPKQVQMAATHIARKAVE
LDLVPGRSPISVAAAAIYMASQASAEKRTQKEIGDIAGVADVTIRQSYRLIYPRAPDLFPTDFKFD
```

TPVDKLPQL
>1CDZA
ELPDFFQGKHFFLYGEFPGDERRKLIRYVTAFNGELEDYMSDRVQFVITAQEWDPSFEEALMDNPS
LAFVRPRWIYSCNEKQKLLPHQLYGVVPQA
>1G4DA
KSIWCSPQEIMAADGMPGSVAGVHYRANVQGWTKRKKEGVKGGKAVEYDVMSMPTKEREQVIAHLG
LST
>1GVPA
MIKVEIKPSQAQFTTRSGVSRQGKPYSLNEQLCYVDLGNEYPVLVKITLDEGQPAYAPGLYTVHLS
SFKVGQFGSLMIDRLRLVPAK
>1GXPA
SPMAVEEVIEMQGLSLDPTSHRVMAGEEPLEMGPTEFKLLHFFMTHPERVYSREQLLNHVWGTNVY
VEDRTVDVHIRRLRKALEPGGHDRMVQTVRGTGYRFSTRF
>1ID3D
SSAAEKKPASKAPAEKKPAAKKTSTSVDGKKRSKVRKETYSSYIYKVLKQTHPDTGISQKSMSILN
SFVNDIFERIATEASKLAAYNKKSTISAREIQTAVRLILPGELAKHAVSEGTRAVTKYSSSTQA
>1MM8A
MVTFMITSALHRAADWAKSVFSSAALGDPRRTARLVNVAAQLAKYSGKSITISSEGSKAAQEGAYR
FIRNPNVSAEAIRKAGAMQTVKLAQEFPELLAIEDTTSLSYRHQVAEELGKLGSIQDKSRGWWVHS
VLLLEATTFRTVGLLHQEWWMRPDDPADADEKESGKWLAAAATSRLRMGSMMSNVIAVCDREADIH
AYLQDKLAHNERFVVRSKHPRKDVESGLYLYDHLKNQPELGGYQISIPQKGVVDKRGKRKNRPARK
ASLSLRSGRITLKQGNITLNAVLAEEINPPKGETPLKWLLLTSEPVESLAQALRVIDIYTHRWRIE
EFHKAWKTGAGAERQRMEKPDNLERMVSILSFVAVRLLQLRESFTPPQALRAQGLLKEAEHVESQS
AETVLTPDECQLLGYLDKGKRKRKEKAGSLQWAYMAIARLGGFMDSKRTGIASWGALWEGWEALQS
KLDGFLAAKDLMAQGIKIG
>1OZJA
MSSILPFTPPIVKRLLGWKKGEQNGQEEKWCEKAVKSLVKKLKKTGQLDELEKAITTQNVNTKCIT
IPRSLDGRLQVSHRKGLPHVIYCRLWRWPDLHSHHELRAMELCEFAFNMKKDEVCVNPYHYQRVET
PVLPPVLVPRHT
>1P4EA
SQFDILCKTPPKVLVRQFVERFERPSGEKIASCAAELTYLCWMITHNGTAIKRATFMSYNTIISNS
LSFDIVNKSLQFKYKTQKATILEASLKKLIPAWEFTIIPYNGQKHQSDITDIVSSLQLQFESSEEA
DKGNSHSKKMLKALLSEGESIWEITEKILNSFEYTSRFTKTKTLYQFLFLATFINCGRFSDIKNVD
PKSFKLVQNKYLGVIIQCLVTETKTSVSRHIYFFSARGRIDPLVYLDEFLRNSEPVLKRVNRTGNS
SSNKQEYQLLKDNLVRSYNKALKKNAPYPIFAIKNGPKSHIGRHLMTSFLSMKGLTELTNVVGNFS
DKRASAVARTTYTHQITAIPDHYFALVSRYYAYDPISKEMIALKDETNPIEEWQHIEQLKGSAEGS
IRYPAWNGIISQEVLDYLSSYINRRIGHHHHHH
>1QAIA
LHETSKEPDVSLGSTWLSDFPQAWAETGGMGLAVRQAPLIIPLKATSTPVSIKQYPMSQEARLGIK
PHIQRLLDQGILVPCQSPWNTPLLPVKKPGTNDYRPVQDLREVNKRVEDIHPTVPNPYNLLSGLPP
SHQWYTVLDLKDAFFCLRLHPTSQPLFAFEWRDPEMGISGQLTWTRLPQGFKNSPTLFDEALHRDL
ADFRIQHPDLILLQYVDDLLLAATSELDCQQGTRALLQTLGNLGYRASAKKAQICQKQVKYLGYLL
K
>1RZSA

MYKKDVIDHFGTQRAVAKALGISDAAVSQWKEVIPEKDAYRLEIVTAGALKYQENAYRQAA
>1SFEA
MTAKQFRHGGENLAVRYALADCELGRCLVAESERGICAILLGDDDATLISELQQMFPAADNAPADL
MFQQHVREVIASLNQRDTPLTLPLDIRGTAFQQQVWQALRTIPCGETVSYQQLANAIGKPKAVRAV
ASACAANKLAIVIPCHRVVRGDGSLSGYRWGVSRKAQLLRREAENEER
>1T4WA
EKWMEIDVLKQKVAKSSDMAFAISSEHEKYLWTKMGCLVPIQVKWKLDKRHFNSNLSLRIRFVKYD
KKENVEYAIRNPRSDVMKCRSHTEREQHFPFDSFFYIRNSEHEFSYSAEKGSTFTLIMYPGAVQAN
FDIIFMCQEKCLDLDDRRKTMCLAVFLDDENGNEILHAYIKQVRIVAYPRRDWKNFCEREDAKQ
>1TAUA
MRGMLPLFEPKGRVLLVDGHHLAYRTFHALKGLTTSRGEPVQAVYGFAKSLLKALKEDGDAVIVVF
DAKAPSFRHEAYGGYKAGRAPTPEDFPRQLALIKELVDLLGLARLEVPGYEADDVLASLAKKAEKE
GYEVRILTADKDLYQLLSDRIHVLHPEGYLITPAWLWEKYGLRPDQWADYRALTGDESDNLPGVKG
IGEKTARKLLEEWGSLEALLKNLDRLKPAIREKILAHMDDLKLSWDLAKVRTDLPLEVDFAKRREP
DRERLRAFLERLEFGSLLHEFGLLESPKALEEAPWPPPEGAFVGFVLSRKEPMWADLLALAAARGG
RVHRAPEPYKALRDLKEARGLLAKDLSVLALREGLGLPPGDDPMLLAYLLDPSNTTPEGVARRYGG
EWTEEAGERAALSERLFANLWGRLEGEERLLWLYREVERPLSAVLAHMEATGVRLDVAYLRALSLE
VAEEIARLEAEVFRLAGHPFNLNSRDQLERVLFDELGLPAIGKTEKTGKRSTSAAVLEALREAHPI
VEKILQYRELTKLKSTYIDPLPDLIHPRTGRLHTRFNQTATATGRLSSSDPNLQNIPVRTPLGQRI
RRAFIAEEGWLLVALDYSQIELRVLAHLSGDENLIRVFQEGRDIHTETASWMFGVPREAVDPLMRR
AAKTINFGVLYGMSAHRLSQELAIPYEEAQAFIERYFQSFPKVRAWIEKTLEEGRRRGYVETLFGR
RRYVPDLEARVKSVREAAERMAFNMPVQGTAADLMKLAMVKLFPRLEEMGARMLLQVHDELVLEAP
KERAEAVARLAKEVMEGVYPLAVPLEVEVGIGEDWLSAKE
>1TNSA
MELWVSPKELANLPGLPKTSAGVIYVAKKQGWQNRTRAGVKGGKAIEYNANSLPVEAKAALLLRQG
EIETSLGYFE
>1TX3A
SFIKPIYQDINSILIGQKVKRPKSGTLSGHAAGEPFEKLVYKFLKENLSDLTFKQYEYLNDLFMKN
PAIIGHEARYKLFNSPTLLFLLSRGKAATENWSIENLFEEKQNDTADILLVKDQFYELLDVKTRNI
SKSAQAPNIISAYKLAQTCAKMIDNKEFDLFDINYLEVDWELNGEDLVCVSTSFAELFKSEPSELY
INWAAAMQIQFHVRDLDQGFNGTREEWAKSYLKHFVTQAEQRAISMIDKFVKPFKKYIL
>1UW0A
MAEQRFCVDYAKRGTAGCKKCKEKIVKGVCRIGKVVPNPFSESGGDMKEWYHIKCMFEKLERARAT
TKKIEDLTELEGWEELEDNEKEQITQHIADLSSKAAGTPKKKAVVQAKLTT
>1V1QA
MASMTGGQQMGRDPNSLMTNRLVLSGTVCRAPLRKVSPSGIPHCQFVLEHRSVQEEAGFHRQAWCQ
MPVIVSGHENQAITHSITVGSRITVQGFISCHKAKNGLSKMVLHAEQIELIDSVDKLAAALEHHHH
HH
>1WG2A
GSSGSSGPSRPVRPNNRCFSCNKKVGVMGFKCKCGSTFCGSHRYPEKHECSFDFKEVGSGPSSG
>1WG6A
GSSGSSGLKGEPDCYALSLESSEQLTLEIPLNDSGSAGLGVSLKGNKSRETGTDLGIFIKSIIHGG
AAFKDGRLRMNDQLIAVNGETLLGKSNHEAMETLRRSMSMEGNIRGMIQLVILRRSGPSSG
>1WIJA

GSSGSSGSQFVLQDLQDATLGSLLSSLMQHCDPPQRKYPLEKGTPPPWWPTGNEEWWVKLGLPKSQ
SPPYRKPHDLKKMWKVGVLTAVINHMLPDIAKIKRHVRQSKCLQDKMTAKESAIWLAVLNQEESLI
QQSGPSSG
>1WVLA
MVKVKFKYKGEEKEVDTSKIKKVWRVGKMVSFTYDDNGKTGRGAVSEKDAPKELLDMLARAEREKK
GVLKKLRAVENELH
>1X37A
MAGYTEIEKLEIVKDHLLPKQIKEHGLKKSNLQLRDQAILDIIRYYTREAGVRSLERQLAAICRKA
AKAIVAEERKRITVTEKNLQDFIGKRIFRYGQAETEDQVGVVTGLAYTTVLRHHHHHH
>1X51A
GSSGSSGPRKASRKPPREESSATCVLEQPGALGAQILLVQRPNSGLLAGLWEFPSVTWEPSEQLQR
KALLQELQRWAGPLPATHLRHLGEVVHTFSHIKLTYQVYGLALEGQTPVTTVPPGARWLTQEEFHT
AAVSTAMKKVFRVYQGQSGPSSG
>1YFJA
MLGAIAYTGNKQSLLPELKSHFPKYNRFVDLFCGGLSVSLNVNGPVLANDIQEPIIEMYKRLINVS
WDDVLKVIKQYKLSKTSKEEFLKLREDYNKTRDPLLLYVLHFHGFSNMIRINDKGNFTTPFGKRTI
NKNSEKRFNHFKQNCDKIIFSSLHFKDVKILDGDFVYVDPPYLITVADYNKFWSEDEEKDLLNLLD
SLNDRGIKFGLSNVLEHHGKENTLLKEWSKKYNVKHLNKKYVFNIYHSKEKNGTDEVYIFN
>1YIOA
MTAKPTVFVVDDDMSVREGLRNLLRSAGFEVETFDCASTFLEHRRPEQHGCLVLDMRMPGMSGIEL
QEQLTAISDGIPIVFITAHGDIPMTVRAMKAGAIEFLPKPFEEQALLDAIEQGLQLNAERRQARET
QDQLEQLFSSLTGREQQVLQLTIRGLMNKQIAGELGIAEVTVKVHRHNIMQKLNVRSLANLVHLVE
KYESFERGVS
>1YJMA
MSQLGSRGRLWLQSPTGGPPPIFLPSDGQALVLGRGPLTQVTDRKCSRNQVELIADPESRTVAVKQ
LGVNPSTVGVHELKPGLSGSLSLGDVLYLVNGLYPLTLRWEELS
>1YLFA
SNAMITMKISSRFSIAVHILSILKNNPSSLCTSDYMAESVNTNPVVIRKIMSYLKQAGFVYVNRGP
GGAGLLKDLHEITLLDVYHAVNVVEEDKLFHIHEQPNPDCPIGANIQAVLEIILIQAQSAMEEVLR
NITMGQLFETLQEKMNA
>1Z63A
MGSSHHHHHHSSGLVPRGSHMASKSFQLLEPYNIKANLRPYQIKGFSWMRFMNKLGFGICLADDMG
LGKTLQTIAVFSDAKKENELTPSLVICPLSVLKNWEEELSKFAPHLRFAVFHEDRSKIKLEDYDII
LTTYAVLLRDTRLKEVEWKYIVIDEAQNIKNPQTKIFKAVKELKSKYRIALTGTPIENKVDDLWSI
MTFLNPGLLGSYSEFKSKFATPIKKGDNMAKEELKAIISPFILRRTKYDKAIINDLPDKIETNVYC
NLTPEQAAMYKAEVENLFNNIDSVTGIKRKGMILSTLLKLKQIVDHPALLKGGEQSVRRSGKMIRT
MEIIEEALDEGDKIAIFTQFVDMGKIIRNIIEKELNTEVPFLYGELSKKERDDIISKFQNNPSVKF
IVLSVKAGGFGINLTSANRVIHFDRWWNPAVEDQATDRVYRIGQTRNVIVHKLISVGTLEEKIDQL
LAFKRSLFKDIISSGDSWITELSTEELRKVIELSVGGY
>1ZS3A
MITKLMIDEKYAKELDKAEIDHHKPTAGAMLGHVLSNLFIENIRLTQAGIYAKSPVKCEYLREIAQ
REVEYFFKISDLLLDENEIVPSTTEEFLKYHKFITEDPKAKYWTDEDLLESFIVDFQAQNMFITRA
IKLANKEEKFALAAGVVELYGYNLQVIRNLAGDLGKSVADFHDEDEDNDN
>2A1JB

MGSSHHHHHHSQDPADLLMEKLEQDFVSRVTECLTTVKSVNKTDSQTLLTTFGSLEQLIAASREDL
ALCPGLGPQKARRLFDVLHEPFLKV
>2AWIA
MFKIGSVLKQIRQELNYHQIDLYSGIMSKSVYIKVEADSRPISVEELSKFSERLGVNFFEILNRAG
MNTKSVNETGKEKLLISKIFTNPDLFDKNFQRIEPKRLTSLQYFSIYLGYISIAHHYNIEVPTFNK
TITSDLKHLYDKRTTFFGIDCEIVSNLLNVLPYEEVSSIIKPMYPIVDSFGKDYDLTIQTVLKNAL
TISIMNRNLKEAQYYINQFEHLKTIKNISINGYYDLEINYLKQIYQFLTDKNIDSYLNAVNIINIF
KIIGKEDIHRSLVEELTKISAKEKFTPPKEVTMYYENYVAIENNPIPEIKEQS
>2B18A
GSSHHHHHHMALLQKTRIINSMLQAAAGKPVNFKEMAETLRDVIDSNIFVVSRRGKLLGYSINQQI
ENDRMKKMLEDRQFPEEYTKNLFNVPETSSNLDINSEYTAFPVENRDLFQAGLTTIVPIIGGGERL
GTLILSRLQDQFNDDDLILAEYGATVVGMEIL
>2B2YA
MKKHHHHHHEEEFETIERFMDCRIGRKGATGATTTIYAVEADGDPNAGFEKNKEPGEIQYLIKWKG
WSHIHNTWETEETLKQQNVRGMKKLDNYKKKDQETKRWLKNASPEDVEYYNCQQELTDDLHKQYQI
VGRIIAHSNQKSAAGYPDYYCKWQGLPYSECSWEDGALISKKFQACIDEYFSRKK
>2C22A
HHHHHHMIVLFVDFDYFYAQVEEVLNPSLKGKPVVVCVFSGRFEDSGAVATANYEARKFGVKAGIP
IVEAKKILPNAVYLPMRKEVYQQVSSRIMNLLREYSEKIEIASIDEAYLDISDKVRDYREAYNLGL
EIKNKILEKEKITVTVGISKNKVFAKIAADMAKPNGIKVIDDEEVKRLIRELDIADVPGIGNITAE
KLKKLGINKLVDTLSIEFDKLKGMIGEAKAKYLISLARDEYNEPIRTRVRKSIGRIVTMKRNSRNL
EEIKPYLFRAIEESYYKLDKRIPKAIHVVAVTEDLDIVSRGRTFPHGISKETAYSESVKLLQKILE
EDERKIRRIGVRFSKFIEAIGLDKFFDT
>2CQGA
GSSGSSGVKRAVQKTSDLIVLGLPWKTTEQDLKEYFSTFGEVLMVQVKKDLKTGHSKGFGFVRFTE
YETQVKVMSQRHMIDGRWCDCKLPNSKQSQDSGPSSG
>2E5RA
GSSGSSGVFHPVECSYCHSESMMGFRYRCQQCHNYQLCQDCFWRGHAGGSHSNQHQMKEYTSW
>2EO0A
MYIVNSNKSRGSSVERYIVSRLRDKGFAVIRAPASGSKRKDHVPDIIALKSGVIILIEVKSRKNGQ
KIYIEKEQAEGIREFAKRSGGELFLGVKLPKMLRFIKFDMLRQTEGGNYAIDLETVEKGMELEDLV
RYVESKISRTLDSFL
>2JULA
MQRTKEAVKASDGNLLGDPGRIPLSKRESIKWQRPRFTRQALMRCCLIKWILSSAAPQGSDSSDSE
LELSTVRHQPEGLDQLQAQTKFTKKELQSLYRGFKNECPTGLVDEDTFKLIYSQFFPQGDATTYAH
FLFNAFDADGNGAIHFEDFVVGLSILLRGTVHEKLKWAFNLYDINKDGCITKEEMLAIMKSIYDMM
GRHTYPILREDAPLEHVERFFQKMDRNQDGVVTIDEFLETCQKDENIMNSMQLFENVI
>2KDZA
KVKFTEEEDLKLQQLVMRYGAKDWIRISQLMITRNPRQCRERWNNYINPALRTDPWSPEEDMLLDQ
KYAEYGPKWNKISKFLKNRSDNNIRNRWMMIARHRAKHQKS
>2KWQA
GPMGMQSIREQSCRVVTCKTCKYTHFKPKETCVSENHDFHWHNGVKRFFKCPCGNRTISLDRLPKK
HCSTCGLFKWERVGMLKEKTGPKLGG
>2L31A

GSKAEKTLGDFAAEYAKSNRSTCKGCMEKIEKGQVRLSKKMVDPEKPQLGMIDRWYHPGCFVKNRE
ELGFRPEYSASQLKGFSLLATEDKEALKKQLPGVKSEGKRKGDEVD
>2L3NA
SVSILRSSVNHREVDEAIDNILRYTNSTEQQFLEAMESTGGRVRIAIAKLLSKQTSGGSGGSKLGG
SGGSRKDLSVKGMLYDSDSQQILNRLRERVSGSTAQSA
>2QU7A
MSLKTGRSNIIAFIVPDQNPFFTEVLTEISHECQKHHLHVAVASSEENEDKQQDLIETFVSQNVSA
IILVPVKSKFQMKREWLKIPIMTLDRELESTSLPSITVDNEEAAYIATKRVLESTCKEVGLLLANP
NISTTIGRKNGYNKAISEFDLNVNPSLIHYSDQQLGTNAQIYSGYEATKTLLSKGIKGIVATNHLL
LLGALQAIKESEKEIKKDVIIVGFDDSYWNEIYTPKLTVISQPVKEMGQVAAKMIYKLIKGKDVTS
IKLSTKLIIRESCSFNEGHHHHHH
>2R5UA
GPHMAVVDDLAPGMDSSPPSEDYGRQPPQDLAAEQSVLGGMLLSKDAIADVLERLRPGDFYRPAHQ
NVYDAILDLYGRGEPADAVTVAAELDRRGLLRRIGGAPYLHTLISTVPTAANAGYYASIVAEKALL
RRLVEAGTRVVQYGYAGAEGADVAEVVDRAQAEIYDVADRRLSEDFVALEDLLQPTMDEIDAIASS
GG
>2UXZA
PQITLWQRPLVTIKIGGQLKEALLDTGADDTVLEEMSLPGRWKPKMIGGIGGFIKVRQYDQILIEI
CGHKAIGTVLVGPTPVNIIGRNLLTQIGCTLNF
>2UYEA
MDLRDIDLNLLVVFNQLLLDRSVSTAGEKLGLTQPAVSNSLKRLRTALNDDLFLRTSKGMEPTPYA
LHLAEPVIYALNTLQTALTTRDSFDPFASTRTFNLAMTDIGEMSVMPPLMEALAQRAPHIQISTLR
PNAGNLKEDMESGAVDLALGLLPELQTGFFQRRLFRHRYVCMFRKDHPSAKSPMSLKQFSELEHVG
VVALNTGHGEVDGLLERAGIKRRMRLVVPHFIAIGPILHSTDLIATVPQRFAVRCEVPFGLTTSPH
PAKLPDIAINLFWHAKYNRDPGNMWLRQLFVELFSEAHHHHHH
>2V57A
GMTSPSIESGARERTRRAILDAAMLVLADHPTAALGDIAAAAGVGRSTVHRYYPERTDLLRALARH
VHDLSNAAIERADPTSGPVDAALRRVVESQLDLGPIVLFVYYEPSILADPELAAYFDIGDEAIVEV
LNRASTERPEYPPGWARRVFWALMQAGYEAAKDGMPRHQIVDAIMTSLTSGIITLPRT
>2V9PA
TLNESLQTEKFDFGTMVQWAYDHKYAEESKIAYEYALAAGSDSNARAFLATNSQAKHVKDCATMVR
HYLRAETQALSMPAYIKARCKLATGEGSWKSILTFFNYQNIELITFINALKLWLKGIPKKNCLAFI
GPPNTGKSMLCNSLIHFLGGSVLSFANHKSHFWLASLADTRAALVDDATHACWRYFDTYLRNALDG
YPVSIDRKHKAAVQIKAPPLLVTSNIDVQAEDRYLYLHSRVQTFRFEQPCTDESGEQPFNITDADW
KSFFVRLWGRLDLIDEEEDSEEDGDSMRTFTCSARNTNAVD
>2WQGA
GSADYSSLTVVQLKDLLTKRNLSVGGLKNEWVQRLIKDDEESKGESEVSPQ
>2XMAA
GSHMTYVILPLEMKKGRGYVYQLEYHLIWCVKYRHQVLVGEVADGLKDILRDIAAQNGLEVITMEV
MPDHVHLLLSATPQQAIPDFVKALKGASARRMFVAYPQLKEKLWGGNLWNPSYCILTVSENTRAQI
QKYIESQHDKE
>2XRNA
SIQVIARAASIMRALGSHPHGLSLAAIAQLVGLPRSTVQRIINALEEEFLVEALGPAGGFRLGPAL
GQLINQAQTDILSLVKPYLRSLAEELDESVSLASLAGDKIYVLDRIVSERELRVVFPIGINVPAAA

TAAGKVLLAALPDETLQAALGEQLPVLTSNTLGRKALVKQLSEVRQSGVASDLDEHIDGVSSFATL
LDTYLGYYSLAIVMPSSRASKQSDLIKKALLQSKLNIERAIGR
>2Y3MA
IHMQNPVFSIRLKQAPLVPTLQQLALAHNTNLIIDDELQGTVSLQLENVDLDQLFRSVAKIKQLDL
WQENGIYYFTKGDTNTKFAGKMEEPFPLSLPMAEEPAQLNTATIKLHFAKASEVMKSLTGGSGSLL
SPNGSITFDDRSNLLLIQDEPRSVRNIKKLIKELDKPIEQLEY
>2Y9ZB
MVAPKPKPAHEQVEPALIPSNWTSVIPLLTSDFKNQYSVISRLKNPNMKPVPYAGDIIKLMAFINK
FSSFFHSDLQNLSFQDFEVGLDLYPGDPNGSAAGIVKGPEDTSLLLYPDFMAIKDIVYCQDKMNLL
FLSLLDLTFTENFDGKSAKKKGPLTTWENLKSSSKKVFSNPLYRLRLVAREWGYPREWRQQLPSDQ
DISKPKTALFEQDEQTPVVDPSHPEILTPNIYTWNANEPLPLESNPLYNREMDKNGILALKPMDRV
VLLRALTDWCASHSSAIHDEIYKLTHGKKDPVFGIQTQQVPRYTIEGVDNTINQFKKLCSLIQSRY
EIRSKKKHFVKQLKEGKKPDLSRKLEILKEIKAELKNAVKSEKDELLFSLYDKWVPLFEGELPDQP
LANPFSERLYKLRLQEFFLGRVPHIGDFYMPRLHSYGDSLEMSTFTDLRNLQALLSKFKNNEYNAF
TLFENDGQSMSAQFKLFYHDTPSLAHDVARGRNTSGKVYWYELCHDSATLLEFLEFLDYKIVKPQD
EKKEGNEKEKEALNNEAHILEQKSTTDNNPSINTNPLPKDAKYNTARKKLQILKEFLSDYYFILRQ
FEQMKVQFADMKPGKRQLRRIQRQTVNYNT
>2YTVA
GSSGSSGLRRATVECVKDQFGFINYEVGDSKKLFFHVKEVQDGIELQAGDEVEFSVILNQRTGKCS
ACNVWRVSGPSSG
>2ZCXA
MPYDDVMTGSGFQRARSAQAKQQREEAILDAARELGTERGIREITLTDIAATVGMHKSALLRYFET
REQIFLKITAEGWKEWSAELCARLRELPGAAPDAVGQVFAATLAARPLFCDLLAQAPLNLERNVSV
ESVRSFKIATLDEVGRIGAELRRLLGVDETQAVDVIATATSLAGALWQMATPGPHIQTLYRSDPRL
AHAVVEVEPRLNRVLGALLRGIADGLEHHHHHH
>3ANGA
AVREYQKKRRRERIFRAAMELFRNRGFQETTATEIAKAAHVSRGTFFNYYPYKEAVLLDYGSQLLA
GLREEVRRLLAQGREPVEVLRHLFRVLAEGTAREKDLLLPMFYELLNPDPVRARAAFEALPLGDLI
AEILKPLREQGVLRQDFSLERMGRTLADLYFLSALRWAAYTPGRDLAEELEKNLRLLLEGMLVREA
PAPGGL
>3AQQA
MSSEPPPPPQPPTHQASVGLLDTPRSRERSPSPLRGNVVPSPLPTRRTRTFSATVRASQGPVYKGV
CKCFCRSKGHGFITPADGGPDIFLHISDVEGEYVPVEGDEVTYKMCSIPPKNEKLQAVEVVITHLA
PGTKHETWSGHVISS
>3AXJB
MPKNGGAGHRNTAPRKRQIPAAQLDEDSPIVQQFRIYSNELIMKHDRHERIVKLSRDITIESKRII
FLLHSIDSRKQNKEKVLEEARQRLNKLIAVNFRAVALELRDQDVYQFRSSYSPGLQEFIQAYTYME
YLCHEDAEGENETKSVSDWQAIQAVMQYVEESSQPKEEPTEGEDVQAIAQVESPKKFQFFVDPTEY
ILGLSDLTGELMRRCINSLGSGDTDTCLDTCKALQHFYSGYISLNCQRARELWRKITTMKQSVLKA
ENVCYNVKVRGGEAAKWGATFDQKPADEVDEGFY
>3B66A
PIFLNVLEAIEPGVVCAGHDNNQPDSFAALLSSLNELGERQLVHVVKWAKALPGFRNLHVDDQMAV
IQYSWMGLMVFAMGWRSFTNVNSRMLYFAPDLVFNEYRMHKSRMYSQCVRMRHLSQEFGWLQITPQ
EFLCMKALLLFSIIPVDGLKNQKFFDELRMNYIKELDRIIACKRKNPTSCSRRFYQLTKLLDSVQP

IARELHQFTFDLLIKSHMVSVDFPEMMAEIISVQVPKILSGKVKPIYFHTQ
>3B6CA
GAMAPLTQDRIVVTALGILDAEGLDALSMRRLAQELKTGHASLYAHVGNRDELLDLVFDIVLTEVE
VPEPEPGRWAEQVKEMCRSLRRMFLAHRDLARIAIDRVPLGPNGMVGMERTMNLLRSGGLHDELAA
YGGDLLSTFVTAEALEQSSRNPGTEQGREQAGVFADQLHGYLKSLPATSFPNLVHLAGPITSLDSD
RRFELGLEIIIAGLLAGAGEAADDQVRTAGSPPAES
>3BFNA
MHHHHHHSSGRENLYFQGPPARVRVAVRLRPFVDGTAGASDPPCVRGMDSCSLEIANWRNHQETLK
YQFDAFYGERSTQQDIYAGSVQPILRHLLEGQNASVLAYGPTGAGKTHTMLGSPEQPGVIPRALMD
LLQLTREEGAEGRPWALSVTMSYLEIYQEKVLDLLDPASGDLVIREDCRGNILIPGLSQKPISSFA
DFERHFLPASRNRTVGATRLNQRSSRSHAVLLVKVDQRERLAPFRQREGKLYLIDLAGSEDNRRTG
NKGLRLKESGAINTSLFVLGKVVDALNQGLPRVPYRDSKLTRLLQDSLGGSAHSILIANIAPERRF
YLDTVSALNFAARSKEVINRPFTNESLQPHALGPVKLSQKELLGPPEAK
>3BJYA
KRIVACDDPDFLTSYFAHSRLHHLSAWKANLKDKFLNENIHKYTKITDKDTYIIFHIDFDCFFATV
AYLCRSSSFSACDFKRDPIVVCHGTKNSDIASCNYVARSYGIKNGMWVSQAEKMLPNGIKLISLPY
TFEQFQLKSEAFYSTLKRLNIFNLILPISIDEAVCVRIIPDNIHNTNTLNARLCEEIRQEIFQGTN
GCTVSIGCSDSLVLARLALKMAKPNGYNITFKSNLSEEFWSSFKLDDLPGVGHSTLSRLESTFDSP
HSLNDLRKRYTLDALKASVGSKLGMKIHLALQGQDDEESLKILYDPKEVLQRKSLSIDINWGIRFK
NITQVDLFIERGCQYLLEKLNEINKTTSQITLKLMRRCKDAPIEPPKYMGMGRCDSFSRSSRLGIP
TNEFGIIATEMKSLYRTLGCPPMELRGLALQFNKLVDV
>3BNIA
MGSSHHHHHHSSGRENLYFQGHMRTVSHPTPLRRAPVQRRSAERLTRILDACADLLDEVGYDALST
RAVALRADVPIGSVYRFFGNKRQMADALAQRNLERYAERVTERLTEAGDGGWRGALDTVLDEYLAM
KRTAPGFSLIDFGNQIPVGDRHAVPNHRVAERLTELLSGYLGRRPDDDLRRVFLVAVETADTLVQL
AFRVAPDGDEKIIEEARELLRAYLGRVLDGS
>3BQOA
EEEEEDAGLVAEAEAVAAGWMLDFLCLSLCRAFRDGRSEDFRRTRNSAEAIIHGLSSLTACQLRTI
YICQFLTRIAAGKTLDAQFENDERITPLESALMIWGSIEKEHDKLHEEIQNLIKIQAIAVCMENGN
FKEAEEVFERIFGDPNSHMPFKSKLLMIISQKDTFHSFFQHFSYNHMMEKIKSYVNYVLSEKSSTF
LMKAAAKVVESKR
>3BROA
SNAMSRDLGRLLKIASNQMSTRFDIFAKKYDLTGTQMTIIDYLSRNKNKEVLQRDLESEFSIKSST
ATVLLQRMEIKKLLYRKVSGKDSRQKCLKLTKKANKLETIILSYMDSDQSQMTSGLNKEEVVFLEK
ILKRMIESD
>3C2GA
MNITQAAEQAIRLWFNTPDPMQRLHMAKTIRTWIRQDKFAQVDQANMPNCVQQILNIIYDGLKPQP
VQLPISYYAQLWYNLLDILRRFTFLPIISPYIHQVVQMFCPRENGPQDFRELICNLISLNWQKDPH
MKHCANQVFQIFNCIIMGVKNEKLRTEFAQHLKFEKLVGTLSEYFNPQVHPGMINPAIFIIFRFII
SKDTRLKDYFIWNNNPHDQPPPPTGLIIKLNAVMIGSYRLIAGQNPETLPQNPELAHLIQVIIRTF
DLLGLLLHDSDAIDGFVRSDGVGAITTVVQYPNNDLIRAGCKLLLQVSDAKALAKTPLENILPFLL
RLIEIHPDDEVIYSGTGFLSNVVAHKQHVKDIAIRSNAIFLLHTIISKYPRLDELTDAPKRNRVCE
IICNCLRTLNNFLMMWIPTPNGETKTAGPNEKQQVCKFIEIDILKKLMSCLSCEGMDTPGLLELRS
TILRSFILLLRTPFVPKDGVLNVIDENRKENLIGHICAAYSWVFRQPNNTRTQSTKQQLVERTISL

LLVLMEQCGAEKEVAQYSYSIDCPLNLLNGNQVKPTFIHNVLVVCDKILEHCPTRADIWTIDRPML
EGLTNHRNSDIAKAANSLLSRFPEN
>3CDLA
SNAMRLTDQKRESIVQAAIAEFGDRGFEITSMDRIAARAEVSKRTVYNHFPSKEELFAEMLQRLWN
CAPPQSEVVYRPLVSLREQLLELLWGKMRNLTDSSFLDLARVVVGATIHSPERAQVWLARINEREE
TFSAWIRAAQKDGRLKPVDPGFAATQMHALLKSFAFWPQVTFNAALLTPQEQSNVVESALNMFLGW
YEIPG
>3CJNA
SNAMAESTDQTEQLRELAEIGLEGYAPYLMNRIMGRYNANLRKEMTALGLSTAKMRALAILSAKDG
LPIGTLGIFAVVEQSTLSRALDGLQADGLVRREVDSDDQRSSRVYLTPAGRAVYDRLWPHMRASHD
RMFQGITPQERQAFLATLNKMLANIRVHEI
>3CZ5A
MSLSTARIMLVDDHPIVREGYRRLIERRPGYAVVAEAADAGEAYRLYRETTPDIVVMDLTLPGPGG
IEATRHIRQWDGAARILIFTMHQGSAFALKAFEAGASGYVTKSSDPAELVQAIEAILAGRRAMSPD
IAQEIAEERVEGREGHHHHHH
>3D6WA
GKSVVTLKTTDGWIPVPFSKVMYLEAKDKKTYVNAEELTGTHKYSLQEFEYLLPKDSFIRCHRSFI
VNVNHIKAIYPDTHSTFLLSMDNGERVPVSQSYASYFRKLLGFGS
>3DM3A
EIKDTYNIGELSPGMTATFEGEVISALPIKEFKRADGSIGKLKSFIVRDETGSIRVTLWDNLTDID
VGRGDYVRVRGYIREGYYGGLECTANYVEILKKGEKIES
>3DMQA
MPFTLGQRWISDTESELGLGTVVAVDARTVTLLFPSTGENRLYARSDSPVTRVMFNPGDTITSHDG
WQMQVEEVKEENGLLTYIGTRLDTEESGVALREVFLDSKLVFSKPQDRLFAGQIDRMDRFALRYRA
RKYSSEQFRMPYSGLRGQRTSLIPHQLNIAHDVGRRHAPRVLLADEVGLGKTIEAGMILHQQLLSG
AAERVLIIVPETLQHQWLVEMLRRFNLRFALFDDERYAEAQHDAYNPFDTEQLVICSLDFARRSKQ
RLEHLCEAEWDLLVVDEAHHLVWSEDAPSREYQAIEQLAEHVPGVLLLTATPEQLGMESHFARLRL
LDPNRFHDFAQFVEEQKNYCPVADAVAMLLAGNKLSNDELNMLGEMIGEQDIEPLLQAANSDSEDA
QSARQELVSMLMDRHGTSRVLFRNTRNGVKGFPKRELHTIKLPLPTQYQTAIKVSGIMGARKSAED
RARDMLYPERIYQEFEGDNATWWNFDPRVEWLMGYLTSHRSQKVLVICAKAATALQLEQVLREREG
IRAAVFHEGMSIIERDRAAAWFAEEDTGAQVLLCSEIGSEGRNFQFASHMVMFDLPFNPDLLEQRI
GRLDRIGQAHDIQIHVPYLEKTAQSVLVRWYHEGLDAFEHTCPTGRTIYDSVYNDLINYLASPDQT
EGFDDLIKNCREQHEALKAQLEQGRDRLLEIHSNGGEKAQALAESIEEQDDDTNLIAFAMNLFDII
GINQDDRGDNMIVLTPSDHMLVPDFPGLSEDGITITFDREVALAREDAQFITWEHPLIRNGLDLIL
SGDTGSSTISLLKNKALPVGTLLVELIYVVEAQAPKQLQLNRFLPPTPVRMLLDKNGNNLAAQVEF
ETFNRQLNAVNRHTGSKLVNAVQQDVHAILQLGEAQIEKSARALIDAARNEADEKLSAELSRLEAL
RAVNPNIRDDELTAIESNRQQVMESLDQAGWRLDALRLIVVTHQ
>3E1SA
MSQQGLERRLLAGLQGLGLTINQAQRAVKHFGADALDRLEKDLFTLTEVEGIGFLTADKLWQARGG
ALDDPRRLTAAAVYALQLAGTQAGHSFLPRSRAEKGVVHYTRVTPGQARLAVETAVELGRLSEDDS
PLFAAEAAATGEGRIYLPHVLRAEKKLASLIRTLLATPPADGAGNDDWAVPKKARKGLSEEQASVL
DQLAGHRLVVLTGGPGTGKSTTTKAVADLAESLGLEVGLCAPTGKAARRLGEVTGRTASTVHRLLG
YGPQGFRHNHLEPAPYDLLIVDEVSMMGDALMLSLLAAVPPGARVLLVGDTDQLPPVDAGLPLLAL
AQAAPTIKLTQVYRQAAKNPIIQAAHGLLHGEAPAWGDKRLNLTEIEPDGGARRVALMVRELGGPG

AVQVLTPMRKGPLGMDHLNYHLQALFNPGEGGVRIAEGEARPGDTVVQTKNDYNNEIFNGTLGMVL
KAEGARLTVDFDGNVVELTGAELFNLQLGYALTVHRAQGSEWGTVLGVLHEAHMPMLSRNLVYTAL
TRARDRFFSAGSASAWQIAAARQREARNTALLERIRAHLEHHHHHH
>3E2QA
LPQSVSRAAITAAYRRPETEAVSMLLEQARLPQPVAEQAHKLAYQLADKLRNQKNASGRAGMVQGL
LQEFSLSSQEGVALMCLAEALLRIPDKATRDALIRDKISNGNWQSHIGRSPSLFVNAATWGLLFTG
KLVSTHNEASLSRSLNRIIGKSGEPLIRKGVDMAMRLMGEQFVTGETIAEALANARKLEEKGFRYS
YDMLGEAALTAADAQAYMVSYQQAIHAIGKASNGRGIYEGPGISIKLSALHPRYSRAQYDRVMEEL
YPRLKSLTLLARQYDIGINIDAEESDRLEISLDLLEKLCFEPELAGWNGIGFVIQAYQKRCPLVID
YLIDLATRSRRRLMIRLVKGAYWDSEIKRAQMDGLEGYPVYTRKVYTDVSYLACAKKLLAVPNLIY
PQFATHNAHTLAAIYQLAGQNYYPGQYEFQCLHGMGEPLYEQVTGKVADGKLNRPCRISAPVGTHE
TLLAYLVRRLLENGANTSFVNRIADTSLPLDELVADPVTAVEKLAQQEGQTGLPHPKIPLPRDLYG
HGRDNSAGLDLANEHRLHHHHHH
>3ECHA
MNYPVNPDLMPALMAVFQHVRTRIQSELDCQRLDLTPPDVHVLKLIDEQRGLNLQDLGRQMCRDKA
LITRKIRELEGRNLVRRERNPSDQRSFQLFLTDEGLAIHLHAELIMSRVHDELFAPLTPVEQATLV
HLLDQCLAAQ
>3EETA
MGSSHHHHHHSSGRENLYFQGHMTFGEQPAYLRVAGDLRKKIVDGSLPPHTRLPSQARIREEYGVS
DTVALEARKVLMAEGLVEGRSGSGTYVRERPVPRRVARSGYRPDSGATPFRQEQADGAVRGTWESH
SEQAEASGAIAERLDIRPGERVMCTKYVFRDAGEVMMLSTSWEPLAVTGRTPVMLPEEGPVGGMGV
VERMAAIDVIVDNVTEEVGARPGLAEELLTLGGVPGHVVLVIQRTYFASGRPVETADVVVPADRYR
VAYHLPVK
>3EYYA
MVSTDWKSDLRQRGYRLTPQRQLVLEAVDTLEHATPDDILGEVRKTASGINISTVYRTLELLEELG
LVSHAHLGHGAPTYHLADRHHHIHLVCRDCTNVIEADLSVAADFTAKLREQFGFDTDMKHFAIFGR
CESCSLKGSTTDS
>3EZ2A
MSDSSQLHKVAQRANRMLNVLTEQVQLQKDELHANEFYQVYAKAALAKLPLLTRANVDYAVSEMEE
KGYVFDKRPAGSSMKYAMSIQNIIDIYEHRGVPKYRDRYSEAYVIFISNLKGGVSKTVSTVSLAHA
MRAHPHLLMEDLRILVIDLDPQSSATMFLSHKHSIGIVNATSAQAMLQNVSREELLEEFIVPSVVP
GVDVMPASIDDAFIASDWRELCNEHLPGQNIHAVLKENVIDKLKSDYDFILVDSGPHLDAFLKNAL
ASANILFTPLPPATVDFHSSLKYVARLPELVKLISDEGCECQLATNIGFMSKLSNKADHKYCHSLA
KEVFGGDMLDVFLPRLDGFERCGESFDTVISANPATYVGSADALKNARIAAEDFAKAVFDRIEFIR
SN
>3GNAA
MGSSHHHHHHSSGLVPGSHMGGRPRQHLLSLTRRAQKHRLRELKIQVKEFADKEEGGDVKAVCLTL
FLLALRARNEHRQADELEAIMQGRGSGLQP
>3GRAA
MSLAPYRVDFILLEHFSMASFTVAMDVLVTANLLRADSFQFTPLSLDGDRVLSDLGLELVATELSA
AALKELDLLVVCGGLRTPLKYPELDRLLNDCAAHGMALGGLWNGAWFLGRAGVLDDYGCSIHPEQR
ASLSERSPQTRITPASFTLDRDRLSAASPNGAMELMLGLVRRLYGDGLAEGVEEILSFSGAREGHH
HHHH
>3HPHA

MVENIPLAEEEHNKWHQDAVSLHLEFGIPRTAAEDIVQQCDVCQENKMPSTLRGSNKRGIDHWQVD
YTHYEDKIILVWVETNSGLIYAERVKGETGQEFRVQTMKWYAMFAPKSLQSDNGPAFVAESTQLLM
KYLGIEHTTGIPWNPQSQALVERTHQTLKNTLEKLIPMFNAFESALAGTLITLNIKRKGGLGTSPM
DIFIFNKEQQRIQQQSKSKQE
>3HQGA
YILPEDWHLRFPSGSEIIQYAASHYVKNSLDPDEQLLDRRRVEYDIFLLVEELHVLDIIRKGFGSV
DEFIALANSVSNRRKSRAGKSLELHLEHLFIEHGLRHFATQAITEGNKKPDFLFPSAGAYHDTEFP
VENLRMLAVKTTCKDRWRQILNEADKIHQVHLFTLQEGVSLAQYREMRESGVRLVVPSSLHKKYPE
AVRAELMTLGAFIAELTGLYADIP
>3HSRA
GSHMYLSKQLCFLFYVSSKEIIKKYTNYLKEYDLTYTGYIVLMAIENDEKLNIKKLGERVFLDSGT
LTPLLKKLEKKDYVVRTREEKDERNLQISLTEQGKAIKSPLAEISVKVFNEFNISEREASDIINNL
RNFVSKNF
>3MKLA
SNALQPNMRTRVCTVINNNIAHEWTLARIASELLMSPSLLKKKLREEETSYSQLLTECRMQRALQL
IVIHGFSIKRVAVSCGYHSVSYFIYVFRNYYGMTPTEYQERSAQRLSNRDSAAS
>1A1VA
MVDFIPVENLETTMRSPVFTDNSSPPAVPQSFQVAHLHAPTGSGKSTKVPAAYAAQGYKVLVLNPS
VAATLGFGAYMSKAHGVDPNIRTGVRTITTGSPITYSTYGKFLADGGCSGGAYDIIICDECHSTDA
TSILGIGTVLDQAETAGARLVVLATATPPGSVTVPHPNIEEVALSTTGEIPFYGKAIPLEVIKGGR
HLIFCHSKKKCDELAAKLVALGINAVAYYRGLDVSVIPTSGDVVVVATDALMTGFTGDFDSVIDCN
TCVTQTVDFSLDPTFTIETTTLPQDAVSRTQRRGRTGRGKPGIYRFVAPGERPSGMFDSSVLCECY
DAGCAWYELTPAETTVRLRAYMNTPGLPVCQDHLEFWEGVFTGLTHIDAHFLSQTKQSGENFPYLV
AYQATVCARAQAPPPSWDQMWKCLIRLKPTLHGPTPLLYRLGAVQNEVTLTHPITKYIMTCMSADL
EVVTGSGSHHHHHH
>1AWCB
DLGKKLLEAARAGQDDEVRILMANGAPFTTDWLGTSPLHLAAQYGHFSTTEVLLRAGVSRDARTKV
DRTPLHMAASEGHANIVEVLLKHGADVNAKDMLKMTALHWATEHNHQEVVELLIKYGADVHTQSKF
CKTAFDISIDNGNEDLAEILQ
>1BRNL
AQVINTFDGVADYLQTYHKLPDNYITKSEAQALGWVASKGNLADVAPGKSIGGDIFSNREGKLPGK
SGRTWREADINYTSGFRNSDRILYSSDWLIYKTTDHYQTFTKIR
>2RF4A
MSQVKRANENRETARFIKKHKKQVTNPIDEKNGTSNCIVRVPIALYVSLAPMYLENPLQGVMKQHL
NPLVMKYNNKVGGVVLGYEGLKILDADPLSKEDTSEKLIKITPDTPFGFTWCHVNLYVWQPQVGDV
LEGYIFIQSASHIGLLIHDAFNASIKKNNIPVDWTFVHNDGNRSLGHWVDSNGEPIDGKLRFTVRN
VHTTGRVVSVDGTLIS
>2VUTA
MAQQKKTIAVVNATGRQAASLIRVAAAVGHHVRAQVHSLKGLIAEELQAIPNVTLFQGPLLNNVPL
MDTLFEGAHLAFINTTSQAGDEIAIGKDLADAAKRAGTIQHYIYSSMPDHSLYGPWPAVPMWAPKF
TVENYVRQLGLPSTFVYAGIYNNNFTSLPYPLFQMELMPDGTFEWHAPFDPDIPLPWLDAEHDVGP
ALLQIFKDGPQKWNGHRIALTFETLSPVQVCAAFSRALNRRVTYVQVPKVEIKVNIPVGYREQLEA
IEVVFGEHKAPYFPLPEFSRPAAGSPKGLGPANGKGAGAGMMQGPGGVISQRVTDEARKLWSGWRD
MEEYAREVFPIEEEANGLDWML

>2WIUA
MPKLVTWMNNQRVGELTKLANGAHTFKYAPEWLASRYARPLSLSLPLQRGNITSDAVFNFFDNLLP
DSPIVRDRIVKRYHAKSRQPFDLLSEIGRDSVGAVTLIPEDETVTHPIMAWEKLTEARLEEVLTAY
KADIPLGMIREENDFRISVAGAQEKTALLRIGNDWCIPKGITPTTHIIKLPIGEIRQPNATLDLSQ
SVDNEYYCLLLAKELGLNVPDAEIIKAGNVRALAVERFDRRWNAERTVLLRLPQEDMCQTFGLPSS
VKYESDGGPGIARIMAFLMGSSEALKDRYDFMKFQVFQWLIGATDGHAKNFSVFIQAGGSYRLTPF
YDIISAFPVLGGTGIHISDLKLAMGLNASKGKKTAIDKIYPRHFLATAKVLRFPEVQMHEILSDFA
RMIPAALDNVKTSLPTDFPENVVTAVESNVLRLHGRLSREYGSKHHHHHH
>3KD1E
MKEFYLTVEQIGDSIFERYIDSNGRERTREVEYKPSLFAHCPESQATKYFDIYGKPCTRKLFANMR
DASQWIKRMEDIGLEALGMDDFKLAYLSDTYNYEIKYDHTKIRVANFDIEVTSPDGFPEPSQAKHP
IDAITHYDSIDDRFYVFDLLNSPYGNVEEWSIEIAAKLQEQGGDEVPSEIIDKIIYMPFDNEKELL
MEYLNFWQQKTPVILTGWNVESFAIPYVYNRIKNIFGESTAKRLSPHRKTRVKVIENMYGSREIIT
LFGISVLDYIDLYKKFSFTNQPSYSLDYISEFELNVGKLKYDGPISKLRESNHQRYISYNIIDVYR
VLQIDAKRQFINLSLDMGYYAKIQIQSVFSPIKTWDAIIFNSLKEQNKVIPQGRSHPVQPYPGAFV
KEPIPNRYKYVMSFDLTSLYPSIIRQVNISPETIAGTFKVAPLHDYINAVAERPSDVYSCSPNGMM
YYKDRDGVVPTEITKWVSQRKEHKGYMLAAQRNGEIIKEALHNPNLSVDEPLDVDYRFDFSDEIKE
KIKKLSAKSLNEMLFRAQRTEVAGMTAQMALKVTCNSLYGALGNVWFRYYDLRNATAITTFGQMAL
QWIERKVNEYLNEVCGTEGEAFVLYGDTDSIYVSADKIIDKVGESKFRDTNHWVDFLDKFARERME
PAIDRGFREMCEYMNNKQHLMFMDREAIAGPPLGSKGIGGFWTGKKRYALNVWDMEGTRYAEPKLK
IMGLETQKSSTPKAVQKALKECIRRMLQEGEESLQEYFKEFEKEFRQLNYISIASVSSANNIAKYD
VGGFPGPKCPFHIRGILTYNRAIKGNIDAPQVVEGEKVYVLPLREGNPFGDKCIAWPSGTEITDLI
KDDVLHWMDYTVLLEKTFIKPLEGFTSAAKLDYEKKASLFDMFDFSAWSHPQFEK
>3IIDA
GAMQGEVSKAASADSTTEGTPADGFTVLSTKSLFLGQKLQVVQADIASIDSDAVVHPTNTDFYIGG
EVGNTLEKKGGKEFVEAVLELRKKNGPLEVAGAAVSAGHGLPAKFVIHCNSPVWGADKCEELLEKT
VKNCLALADDKKLKSIAFPSIGSGRNGFPKQTAAQLILKAISSYFVSTMSSSIKTVYFVLFDSESI
GIYVQEMAKLDAN
>3PGZA
MAHHHHHHMGTLEAQTQGPGSMAGSLNKVILIGNLGADPEIRRLNSGDQVANLRIATSESWRDRNT
NERKERTEWHNIVIFNENLVKVVEQYLKKGSKIYIEGQLQTRKWQDQNGNDRYTTEIVLQKYRGEL
QMLDGRAAAGGEQMQGANQSSGAYSSVGFGDNSANQRDVFGSNNSQLGESFSHKLDDDVPF
>3RH2A
GMKTRDKIIQASLELFNEHGERTITTNHIAAHLDISPGNLYYHFRNKEDIIRCIFDQYEQHLLLGF
KPYADQKVDLELLMSYFDAMFYTMWQFRFMYANLADILARDDTLKARYLKVQQAVLEQSIAVLNQL
KKDGILQIEDERIADLADTIKMIIGFWISYKLTQSSIATISKASLYEGLLRVLMIFKAYSTPDSLA
NFDRLEQHFRSQSN
>2LEFA
MHIKKPLNAFMLYMKEMRANVVAESTLKESAAINQILGRRWHALSREEQAKYYELARKERQLHMQL
YPGWSARDNYGKKKKRKREK
>2NP2A
MSFSRRPKVTKSDIVDQIALNIKNNNLKLEKKYIRLVIDAFFEELKSNLCSNNVIEFRSFGTFEVR
KRKGRLNARNPQTGEYVKVLDHHVAYFRPGKDLKERVWGIKG
>2O8BB

MGSAPQNSESQAHVSGGGDDSSRPTVWYHETLEWLKEEKRRDEHRRRPDHPDFDASTLYVPEDFLN
SCTPGMRKWWQIKSQNFDLVICYKVGKFYELYHMDALIGVSELGLVFMKGNWAHSGFPEIAFGRYS
DSLVQKGYKVARVEQTETPEMMEARCRKMAHISKYDRVVRREICRIITKGTQTYSVLEGDPSENYS
KYLLSLKEKEEDSSGHTRAYGVCFVDTSLGKFFIGQFSDDRHCSRFRTLVAHYPPVQVLFEKGNLS
KETKTILKSSLSCSLQEGLIPGSQFWDASKTLRTLLEEEYFREKLSDGIGVMLPQVLKGMTSESDS
IGLTPGEKSELALSALGGCVFYLKKCLIDQELLSMANFEEYIPLDSDTVSTTRSGAIFTKAYQRMV
LDAVTLNNLEIFLNGTNGSTEGTLLERVDTCHTPFGKRLLKQWLCAPLCNHYAINDRLDAIEDLMV
VPDKISEVVELLKKLPDLERLLSKIHNVGSPLKSQNHPDSRAIMYEETTYSKKKIIDFLSALEGFK
VMCKIIGIMEEVADGFKSKILKQVISLQTKNPEGRFPDLTVELNRWDTAFDHEKARKTGLITPKAG
FDSDYDQALADIRENEQSLLEYLEKQRNRIGCRTIVYWGIGRNRYQLEIPENFTTRNLPEEYELKS
TKKGCKRYWTKTIEKKLANLINAEERRDVSLKDCMRRLFYNFDKNYKDWQSAVECIAVLDVLLCLA
NYSRGGDGPMCRPVILLPEDTPPFLELKGSRHPCITKTFFGDDFIPNDILIGCEEEEQENGKAYCV
LVTGPNMGGKSTLMRQAGLLAVMAQMGCYVPAEVCRLTPIDRVFTRLGASDRIMSGESTFFVELSE
TASILMHATAHSLVLVDELGRGTATFDGTAIANAVVKELAETIKCRTLFSTHYHSLVEDYSQNVAV
RLGHMACMVENECEDPSQETITFLYKFIKGACPKSYGFNAARLANLPEEVIQKGHRKAREFEKMNQ
SLRLFREVCLASERSTVDAEAVHKLLTLIKEL
>1BAZA
MKGMSKMPQVNLRWPREVLDLVRKVAEENGRSVNSEIYQRVMESFKKEGRIGA
>1BJTA
ENALKKSDGTRKSRITNYPKLEDANKAGTKEGYKCTLVLTEGDSALSLAVAGLAVVGRDYYGCYPL
RGKMLNVREASADQILKNAEIQAIKKIMGLQHRKKYEDTKSLRYGHLMIMTDQDHDGSHIKGLIIN
FLESSFLGLLDIQGFLLEFITPIIKVSITKPTKNTIAFYNMPDYEKWREEESHKFTWKQKYYKGLG
TSLAQEVREYFSNLDRHLKIFHSLQGNDKDYIDLAFSKKKADDRKEWLRQYEPGTVLDPTLKEIPI
SDFINKELILFSLADNIRSIPNVLDGFKPGQRKVLYGCFKKNLKSELKVAQLAPYVSECTAYHHGE
QSLAQTIIGLAQNFVGSNNIYLLLPNGAFGTRATGGKDAAAARYIYTELNKLTRKIFHPADDPLYK
YIQEDEKTVEPEWYLPILPMILVNGAEGIGTGWSTYIPPFNPLEIIKNIRHLMNDEELEQMHPWFR
GWTGTIEEIEPLRYRMYGRIEQIGDNVLEITELPARTWTSTIKEYLLLGLSGNDKIKPWIKDMEEQ
HDDNIKFIITLSPEEMAKTRKIGFYERFKLISPISLMNMVAFDPHGKIKKYNSVNEILSEFYYVRL
EYYQKRKDHMSERLQWEVEKYSFQVKFIKMIIEKELTVTNKPRNAIIQELENLGFPRFNKEGKPYY
GSPNDEIAEQINDVKGATSDEEDEESSHEDTENVINGPEELYGTYEYLLGMRIWSLTKERYQKLLK
QKQEKETELENLLKLSAKDIWNTDLKAFEVGYQEFLQRDAEARGGNVPNKGSKTKGKGKRKLVDDE
D
>1CF7B
RSKKGDKNGKGLRHFSMKVCEKVQRKGTTSYNEVADELVSEFTNSNNHLAADSAYDQKNIRRRVYD
ALNVLMAMNIISKEKKEIKWIGLPTNSAQ
>1D3UB
MVSDAAERNLAFALSELDRITAQLKLPRHVEEEAARLYREAVRKGLIRGRSIESVMAACVYAACRL
LKVPRTLDEIADIARVDKKEIGRSYRFIARNLNLTPKKLFVKPTDYVNKFADELGLSEKVRRRAIE
ILDEAYKRGLTSGKSPAGLVAAALYIASLLEGEKRTQREVAEVARVTEVTVRNRYKELVEKLKIKV
PIA
>1D8BA
ELNNLRMTYERLRELSLNLGNRMVPPVGNFMPDSILKKMAAILPMNDSAFATLGTVEDKYRRRFKY
FKATIADLSKKRSSE
>1F4SP

GSMADTRRRQNHSCDPCRKGKRRCDAPENRNEANENGWVSCSNCKRWNKDCTFNWLSSQRSKNSS
>2O8KA
HMLTQGELMKLIKEIVENEDKRKPYSDQEIANILKEKGFKVARRTVAKYREMLGIPSSRERRI
>2OSTA
MGSTKLKGDIAQQAAIMRALKMGWGVLKPLGDRLSYDLVFDVEGILLKVQVKSSWKSEKTGNYVVD
NRRTRTNRRNIVRSPYRGNDFDFAVAYVEELELFYVFPVDVFISYGSEIHLVETDKRQRKPRSFGY
REAWHLILQKGAAQKETSA
>2OWLA
MLWFKNLMVYRLSREISLRAEEMEKQLASMAFTPCGSQDMAKMGWVPPMGSHSDALTHVANGQIVI
CARKEEKILPSPVIKQALEAKIAKLEAEQARKLKKTEKDSLKDEVLHSLLPRAFSRFSQTMMWIDT
VNGLIMVDCASAKKAEDTLALLRKSLGSLPVVPLSMENPIELTLTEWVRSGSAAQGFQLLDEAELK
SLLEDGGVIRAKKQDLTSEEITNHIEAGKVVTKLALDWQQRIQFVMCDDGSLKRLKFCDELRDQNE
DIDREDFAQRFDADFILMTGELAALIQNLIEGLGGEAQR
>2P4WA
MGEELNRLLDVLGNETRRRILFLLTKRPYFVSELSRELGVGQKAVLEHLRILEEAGLIESRVEKIP
RGRPRKYYMIKKGLRLEILLTPTLFGSEMYEAKGVRKSPEYEQAKELIKSQEPINVKMRELAEFLH
ELNERIREIIEEKRELEEARILIETYIENTMRRLAEENRQIIEEIFRDIEKILPPGYARSLKEKFL
NINI
>2P6RA
MKVEELAESISSYAVGILKEEGIEELFPPQAEAVEKVFSGKNLLLAMPTAAGKTLLAEMAMVREAI
KGGKSLYVVPLRALAGEKYESFKKWEKIGLRIGISTGDYESRDEHLGDCDIIVTTSEKADSLIRNR
ASWIKAVSCLVVDEIHLLDSEKRGATLEILVTKMRRMNKALRVIGLSATAPNVTEIAEWLDADYYV
SDWRPVPLVEGVLCEGTLELFDGAFSTSRRVKFEELVEECVAENGGVLVFESTRRGAEKTAVKLSA
ITAKYVENEGLEKAILEENEGEMSRKLAECVRKGAAFHHAGLLNGQRRVVEDAFRRGNIKVVVATP
TLAAGVNLPARRVIVRSLYRFDGYSKRIKVSEYKQMAGRAGRPGMDERGEAIIIVGKRDREIAVKR
YIFGEPERITSKLGVETHLRFHSLSIICDGYAKTLEELEDFFADTFFFKQNEISLSYELERVVRQL
ENWGMVVEAAHLAPTKLGSLVSRLYIDPLTGFIFHDVLSRMELSDIGALHLICRTPDMERLTVRKT
DSWVEEEAFRLRKELSYYPSDFSVEYDWFLSEVKTALCLKDWIEEKDEDEICAKYGIAPGDLRRIV
ETAEWLSNAMNRIAEEVGNTSVSGLTERIKHGVKEELLELVRIRHIGRVRARKLYNAGIRNAEDIV
RHREKVASLIGRGIAERVVEGISVKSLNPESAAALEHHHHHH
>3BHQA
GMKIDGETRSARKDREIIQAATAAFISKGYDGTSMEEIATKAGASKQTVYKHFTDKETLFGEVVLS
TASQVNDIIESVTTLLSEAIFMEGGLQQLARRLIAVLMDEELLKLRRLIIANADRMPQLGRAWYEK
GFERMLASTASCFQKLTNRGLIQTGDPYLAASHLFGMLLWIPMNEAMFTGSNRRSKAELERHADAS
VEAFLAVYGVQPK
>3BRUA
SNAMPLTDTPPSVPQKPRRGRPRGAPDASLAHQSLIRAGLEHLTEKGYSSVGVDEILKAARVPKGS
FYHYFRNKADFGLALIEAYDTYFARLLDQAFLDGSLAPLARLRLFTRMAEEGMARHGFRRGCLVGN
LGQEMGALPDDFRAALIGVLETWQRRTAQLFREAQACGELSADHDPDALAEAFWIGWEGAILRAKL
ELRPDPLHSFTRTFGRHFVTRTQE
>3C07A
MGSSHHHHHHSSGRENLYFQGHMPATNDGPDDGAHLSKSEQTRALILETAMRLFQERGYDRTTMRA
IAQEAGVSVGNAYYYFAGKEHLIQGFYDRIAAEHRAAVREVLARETDLEARLAGVLKVWLDIATPY
HEFAVQFFKNAADPDSPLSPFSPESEHARVEAIGIHRAVLAGAKTKVPEELRDILPELMWLSQMGL

VLYWIFDRTEGRERSYRLAERGARLTARGVVLARFRVLRPLVREVHELFTDFLPGMTKVMPDPAKK
PTRDAGPQA
>3C2GA
MNITQAAEQAIRLWFNTPDPMQRLHMAKTIRTWIRQDKFAQVDQANMPNCVQQILNIIYDGLKPQP
VQLPISYYAQLWYNLLDILRRFTFLPIISPYIHQVVQMFCPRENGPQDFRELICNLISLNWQKDPH
MKHCANQVFQIFNCIIMGVKNEKLRTEFAQHLKFEKLVGTLSEYFNPQVHPGMINPAIFIIFRFII
SKDTRLKDYFIWNNNPHDQPPPPTGLIIKLNAVMIGSYRLIAGQNPETLPQNPELAHLIQVIIRTF
DLLGLLLHDSDAIDGFVRSDGVGAITTVVQYPNNDLIRAGCKLLLQVSDAKALAKTPLENILPFLL
RLIEIHPDDEVIYSGTGFLSNVVAHKQHVKDIAIRSNAIFLLHTIISKYPRLDELTDAPKRNRVCE
IICNCLRTLNNFLMMWIPTPNGETKTAGPNEKQQVCKFIEIDILKKLMSCLSCEGMDTPGLLELRS
TILRSFILLLRTPFVPKDGVLNVIDENRKENLIGHICAAYSWVFRQPNNTRTQSTKQQLVERTISL
LLVLMEQCGAEKEVAQYSYSIDCPLNLLNGNQVKPTFIHNVLVVCDKILEHCPTRADIWTIDRPML
EGLTNHRNSDIAKAANSLLSRFPEN

## (2) 122 non DNA-binding proteins

>1DUR
AYVINDSCIACGACKPECPVNCIQEGSIYAIDADSCIDCGSCASVCPVGAPNPED
>2CCY
QQSKPEDLLKLRQGLMQTLKSQWVPIAGFAAGKADLPADAAQRAENMAMVAKLAPIGWAKGTEALP
NGETKPEAFGSKSAEFLEGWKALATESTKLAAAAKAGPDALKAQAAATGKVCKACHEEFKQD
>2TSC
MKQYLELMQKVLDEGTQKNDRTGTGTLSIFGHQMRFNLQDGFPLVTTKRCHLRSIIHELLWFLQGD
TNIAYLHENNVTIWDEWADENGDLGPVYGKQWRAWPTPDGRHIDQITTVLNQLKNDPDSRRIIVSA
WNVGELDKMALAPCHAFFQFYVADGKLSCQLYQRSCDVFLGLPFNIASYALLVHMMAQQCDLEVGD
FVWTGGDTHLYSNHMDQTHLQLSREPRPLPKLIIKRAPESIFDYRFEDFEIEGYDPHPGIKAPVAI
>4GR1
ACRQEPQPQGPPPAAGAVASYDYLVIGGGSGGLASARRAAELGARAAVVESHKLGGTCVNVGCVPK
KVMWNTAVHSEFMHDHADYGFPSCEGKFNWRVIKEKRDAYVSRLNAIYQNNLTKSHIEIIRGHAAF
TSDPKPTIEVSGKKYTAPHILIATGGMPSTPHESQIPGASLGITSDGFFQLEELPGRSVIVGAGYI
AVEMAGILSALGSKTSLMIRHDKVLRSFDSMISTNCTEELENAGVEVLKFSQVKEVKKTLSGLEVS
MVTAVPGRLPVMTMIPDVDCLLWAIGRVPNTKDLSLNKLGIQTDDKGHIIVDEFQNTNVKGIYAVG
DVCGKALLTPVAIAAGRKLAHRLFEYKEDSKLDYNNIPTVVFSHPPIGTVGLTEDEAIHKYGIENV
KTYSTSFTPMYHAVTKRKTKCVMKMVCANKEEKVVGIHMQGLGCDEMLQGFAVAVKMGATKADFDN
TVAIHPTSSEELVTLR
>1ACX
APAFSVSPASGASDGQSVSVSVAAAGETYYIAQCAPVGGQDACNPATATSFTTDASGAASFSFTVR
KSYAGQTPSGTPVGSVDCATDACNLGAGNSGLNLGHVALTFG
>1ECA
LSADQISTVQASFDKVKGDPVGILYAVFKADPSIMAKFTQFAGKDLESIKGTAPFETHANRIVGFF
SKIIGELPNIEADVNTFVASHKPRGVTHDQLNNFRAGFVSYMKAHTDFAGAEAAWGATLDTFFGMI
FSKM
>2CYP
TTPLVHVASVEKGRSYEDFQKVYNAIALKLREDDEYDNYIGYGPVLVRLAWHTSGTWDKHDNTGGS
YGGTYRFKKEFNDPSNAGLQNGFKFLEPIHKEFPWISSGDLFSLGGVTAVQEMQGPKIPWRCGRVD
TPEDTTPDNGRLPDADKDADYVRTFFQRLNMNDREVVALMGAHALGKTHLKNSGYEGPWGAANNVF

TNEFYLNLLNEDWKLEKNDANNEQWDSKSGYMMLPTDYSLIQDPKYLSIVKEYANDQDKFFKDFSK
AFEKLLENGITFPKDAPSPFIFKTLEEQGL
>4PFK
MKRIGVLTSGGDSPGMNAAIRSVVRKAIYHGVEVYGVYHGYAGLIAGNIKKLEVGDVGDIIHRGGT
ILYTARCPEFKTEEGQKKGIEQLKKHGIQGLVVIGGDGSYQGAKKLTEHGFPCVGVPGTIDNDIPG
TDFTIGFDTALNTVIDAIDKIRDTATSHERTYVIEVMGRHAGDIALWSGLAGGAETILIPEADYDM
NDVIARLKRGHERGKKHSIIIVAEGVGSGVDFGRQIQEATGFETRVTVLGHVQRGGSPTAFDRVLA
SRLGARAVELLLEGKGGRCVGIQNNQLVDHDIAEALANKHTIDQRMYALSKELSI
>1AZU
AECSVDIQGNDQMQFNTNAITVDKSCKQFTVNLSHPGNLPKNVMGHNWVLSTAADMQGVVTDGMAS
GLDKDYLKPDDSRVIAHTKLIGSGEKDSVTFDVSKLKEGEQYMFFCTFPGHSALMKGTLTLK
>1ETU
SKEKFERTKPHVNVGTIGHVDHGKTTLTAAITTVLAKTYGGAARAFDQIDNAPEEKARGITINTSH
VEYDTPTRHYAHVDCPGHADYVKNMITGAAQMDGAILVVAATDGPMPQTREHILLGRQVGVPYIIV
FLNKCDMVDDEELLELVEMEVRELLSQYDFPGDDTPIVRGSALKALEGDAEWEAKILELAGFLDSY
IPEPERAIDKPFLLPIEDVFSISGRGTVVTGRVERGIIKVGEEVEIVGIKETQKSTCTGVEMFRKL
LDEGRAGENVGVLLRGIKREEIERGQVLAKPGTIKPHTKFESEVYILSKDEGGRHTPFFKGYRPQF
YFRTTDVTGTIELPEGVEMVMPGDNIKMVVTLIHPIAMDDGLRFAIREGGRTVGAGVVAKVLG
>1PYP
MKIVYWSGTGNTEKMAELIAKGIIESGKDVNTINVSDVNIDELLNEDILILGCSAMGDEVLEESEF
EPFIEEISTKISGKKVALFGSYGWGDGKWMRDFEERMNGYGCVVVETPLIVQNEPDEAEQDCIEFG
KKIANI
>3AIT
GLGDELEEVIVEKTKQTVASISSGPKHTQKVPILTANETGATMPVLPSDSIETRTTYMHFNGSETD
VECFLGRAACVHVTEIQNKDATGIDNHREAKLFNDWKINLSSLVQLRKKLELFTYVRFDSEYTILA
TASQPDSANYSSNLVVQAMYVPPGAPNPKEWDDYTWQSASNPSVFFKVGDTSRFSVPYVGLASAYN
CFYDGYSHDDAETQYGITVLNHMGSMAFRIVNEHDEHKTLVKIRVYHRAKHVEAWIPRAPRALPYT
SIGRTNYPKNTEPVIKKRKGDIKSY
>4RHV
VVYTDCTESGQNLCLCEGSNVCGQGNKCILGSDGEKNQCVTGEGTPEPQSHNDGDFEEIPEEYLQ
>6HIR
NVYHDGACPEVKPVDNFDWSNYHGKWWEVAKYPNSVEKYGKCGWAEYTPEGKSVKVSNYHVIHGKE
YFIEGTAYPVGDSKIGKIYHKLTYGGVTKENVFNVLSTDNKNYIIGYYCKYDEDKKGHQDFVWVLS
RSKVLTGEAKTAVENYLIGSPVVDSQKLVYSDFSEAACKVN
>1BBP
ADNKFNKEQQNAFYEILHLPNLNEEQRNGFIQSLKDDPSQSANLLAEAKKLNDAQXXK
>1FC2
SPNVEACGYSDRVQQITLGNSTITTQEAANAVVCYAEWPEYLPDVDASDVNKTSKPDTSVCRFYTL
DSKTWTTGSKGWCWKLPDALKDMGVFGQNMFFHSLGRSGYTVHVQCNATKFHSGCLLVVVIPEHQL
ASHEGGNVSVKYTFTHPGERGIDLSSANEVGGPVKDVLYNMNGTLLGNLLIFPHQFINLRTNNTAT
IVIPYINSVPIDSMTRHNNVSLMVIPIAPLTVPTGATPSLPITVTIAPMCTEFSGIRSKSIVPQ
>1R09
ADTRIGVTIYKYDDNFMSVVRKAIEQDAKAAPDVQLLMNDSQNDQSKQNDQIDVLLAKGVKALAIN
LVDPAAAGTVIEKARGQNVPVVFFNKEPSRKALDSYDKAYYVGTDSKESGIIQGDLIAKHWAANQG

WDLNKDGQIQFVLLKGEPGHPDAEARTTYVIKELNDKGIKTEQLQLDTAMWDTAQAKDKMDAWLSG
PNANKIEVVIANNDAMAMGAVEALKAHNKSSIPVFGVDALPEALALVKSGALAGTVLNDANNQAKA
TFDLAKNLADGKGAADGTNWKIDNKVVRVPYVGVDKDNLAEFSKK
>3BLM
GLPTTTLPGSGQFLTTDDRQSPSALPNYEPTPRIHIPGKVHNLLEIIQVDTLIPMNNTHTKDEVNS
YLIPLNANRQNEQVFGTNLFIGDGVFKTTLLGEIVQYYTHWSGSLRFSLMYTGPALSSAKLILAYT
PPGARGPQDRREAMLGTHVVWDIGLQSTIVMTIPWTSGVQFRYTDPDTYTSAGFLSCWYQTSLILP
PETTGQVYLLSFISACPDFKLRLMKDTQTISQTVALTE
>4RHV
ITGTSTVGVGRGVLGDQKNINTTYSTYYYLQDNTRGDGIFTYDAKYRTTLPGSLWADADNQFFASY
DAPAVDAHYYAGVTYDYYKNVHNRLSYDGNNAAIRSSVHYSQGYNNAFWNGSEMVYGDGDGQTFIP
LSGGIDVVAHELTHAVTDYTAGLIYQNESGAINEAISDIFGTLVEFYANKNPDWEIGEDVYTPGIS
GDSLRSMSDPAKYGDPDHYSKRYTGTQDNGGVHINSGIINKAAYLISQGGTHYGVSVVGIGRDKLG
KIFYRALTQYLTPTSNFSQLRAAAVQSATDLYGSTSQEVASVKQAFDAVGVK
>6TMN
AAPCFCSGKPGRGDLWILRGTCPGGYGYTSNCYKWPNICCYPH
>1BDS
QVQLKESGPGLVAPSQSLSITCTVSGFSLTGYGVNWVRQPPGKGLEWLGMIWGDGNTDYNSALKSR
LSISKDNSKSQVFLKMNSLHTDDTARYYCARERDYRLDYWGQGTTLTVSSASTTPPSVFPLAPGSA
AQTNSMVTLGCLVKGYFPEPVTVTWNSGSLSSGVHTFPAVLQSDLYTLSSSVTVPSSPRPSETVTC
NVAHPASSTKVDKKIVPRDC
>1FDL
ERDCRVSSFRVKENFDKARFSGTWYAMAKKDPEGLFLQDNIVAEFSVDETGQMSATAKGRVRLLNN
WDVCADMVGTFTDTEDPAKFKMKYWGVASFLQKGNDDHWIVDTDYDTYAVQYSCRLLNLDGTCADS
YSFVFSRDPNGLPPEAQKIVRQRQEELCLARQYRLIVHNGYCDGRSERNL
>1RBP
MSAEHVLTMLNEHEVKFVDLRFTDTKGKEQHVTIPAHQVNAEFFEEGKMFDGSSIGGWKGINESDM
VLMPDASTAVIDPFFADSTLIIRCDILEPGTLQGYDRDPRSIAKRAEDYLRATGIADTVLFGPEPE
FFLFDDIRFGASISGSHVAIDDIEGAWNSSTKYEGGNKGHRPGVKGGYFPVPPVDSAQDIRSEMCL
VMEQMGLVVEAHHHEVATAGQNEVATRFNTMTKKADEIQIYKYVVHNVAHRFGKTATFMPKPMFGD
NGSGMHCHMSLAKNGTNLFSGDKYAGLSEQALYYIGGVIKHAKAINALANPTTNSYKRLVPGYEAP
VMLAYSARNRSASIRIPVVASPKARRIEVRFPDPAANPYLCFAALLMAGLDGIKNKIHPGEPMDKN
LYDLPPEEAKEIPQVAGSLEEALNALDLDREFLKAGGVFTDEAIDAYIALRREEDDRVRMTPHPVE
FELYYSV
>2GLS
RICFNHQSSQPQTTKTCSPGESSCYHKQWSDFRGTIIERGCGCPTVKPGIKLSCCESEVCNN
>3EBX
GAQVSTQKSGSHENQNILTNGSNQTFTVINYYKDAASTSSAGQSLSMDPSKFTEPVKDLMLKGAPA
LN
>4RHV
ADNRDPASDQMKHWKEQRAAQKPDVLTTGGGNPVGDKLNSLTVGPRGPLLVQDVVFTDEMAHFDRE
RIPERVVHAKGAGAFGYFEVTHDITRYSKAKVFEHIGKRTPIAVRFSTVAGESGSADTVRDPRGFA
VKFYTEDGNWDLVGNNTPIFFIRDALLFPSFIHSQKRNPQTHLKDPDMVWDFWSLRPESLHQVSFL
FSDRGIPDGHRHMDGYGSHTFKLVNADGEAVYCKFHYKTDQGIKNLSVEDAARLAHEDPDYGLRDL

FNAIATGNYPSWTLYIQVMTFSEAEIFPFNPFDLTKVWPHGDYPLIPVGKLVLNRNPVNYFAEVEQ
LAFDPSNMPPGIEPSPDKMLQGRLFAYPDTHRHRLGPNYLQIPVNCPYRARVANYQRDGPMCMMDN
QGGAPNYYPNSFSAPEHQPSALEHRTHFSGDVQRFNSANDDNVTQVRTFYLKVLNEEQRKRLCENI
AGHLKDAQLFIQKKAVKNFSDVHPEYGSRIQALLDKYNEEKPKN
>7CAT
MERYENLFAQLNDRREGAFVPFVTLGDPGIEQSLKIIDTLIDAGADALELGVPFSDPLADGPTIQN
ANLRAFAAGVTPAQCFEMLALIREKHPTIPIGLLMYANLVFNNGIDAFYARCEQVGVDSVLVADVP
VEESAPFRQAALRHNIAPIFICPPNADDDLLRQVASYGRGYTYLLSRSGVTGAENRGALPLHHLIE
KLKEYHAAPALQGFGISSPEQVSAAVRAGAAGAISGSAIVKIIEKNLASPKQMLAELRSFVSAMKA
ASRA
>1FKF
VHQVLYRALVSTKWLAESVRAGKVGPGLRVLDASWYSPGTREARKEYLERHVPGASFFDIEECRDK
ASPYEVMLPSEAGFADYVGSLGISNDTHVVVYNGDDLGSFYAPRVWWMFRVFGHRTVSVLNGGFRN
WLKEGHPVTSEPSRPEPAIFKATLNRSLLKTYEQVLENLESKRFQLVDSRAQGRYLGTQPEPDAVG
LDSGHIRGSVNMPFMDFLTENGFEKSPEELRAMFEAKKVDLTKPLIATCRKGVTACHIALAAYLCG
KPDVAIYDGSWFEWFHRAPPETWVSQGKG
>2HMZ
QDLPGNDNSTATLCLGHHAVPNGTLVKTITDDQIEVTNATELVQSSSTGKICNNPHRILDGIDCTL
IDALLGDPHCDVFQNETWDLFVERSKAFSNCYPYDVPDYASLRSLVASSGTLEFITEGFTWTGVTQ
NGGSNACKRGPGSGFFSRLNWLTKSGSTYPVLNVTMPNNDNFDKLYIWGIHHPSTNQEQTSLYVQA
SGRVTVSTRRSQQTIIPNIGSRPWVRGQSSRISIYWTIVKPGDVLVINSNGNLIAPRGYFKMRTGK
SSIMRSDAPIDTCISECITPNGSIPNDKPFQNVNKITYGACPKYVKQNTLKLATGMRNVPEKQT
>3HMG
MKKYTCTVCGYIYDPEDGDPDDGVNPGTDFKDIPDDWVCPLCGVGKDEFEEVEE
>4RXN
MESKVVVPAQGKKITLQNGKLNVPENPIIPYIEGDGIGVDVTPAMLKVVDAAVEKAYKGERKISWM
EIYTGEKSTQVYGQDVWLPAETLDLIREYRVAIKGPLTTPVGGGIRELNVALRQELDLYICLRPVR
YYQGTPSPVKHPELTDMVIFRENSEDIYAGIEWKADSADAEKVIKFLREEMGVKKIRFPEHCGIGI
KPCSEEGTKRLVRAAIEYAIANDRDSVTLVHKGNIMKFTEGAFKDWGYQLAREEFGGELIDGGPWL
KVKNPNTGKEIVIKDVIADAFLQQILLRPAEYDVIACMNLNGDYISDALAAQVGGIGIAPGANIGD
ECALFEATHGTAPKYAGQDKVNPGSIILSAEMMLRHMGWTEAADLIVKGMEGAINAKTVTYDFERL
MDGAKLLKCSEFGDAIIENM
>7ICD
MTTLLNPYFGEFGGMYVPQILMPALNQLEEAFVRAQKDPEFQAQFADLLKNYAGRPTALTKCQNIT
AGTRTTLYLKREDLLHGGAHKTNQVLGQALLAKRMGKSEIIAETGAGQHGVASALASALLGLKCRI
YMGAKDVERQSPNVFRMRLMGAEVIPVHSGSATLKDACNEALRDWSGSYETAHYMLGTAAGPHPYP
TIVREFQRMIGEETKAQILDKEGRLPDAVIACVGGGSNAIGMFADFINDTSVGLIGVEPGGHGIET
GEHGAPLKHGRVGIYFGMKAPMMQTADGQIEESYSISAGLDFPSVGPQHAYLNSIGRADYVSITDD
EALEAFKTLCRHEGIIPALESSHALAHALKMMREQPEKEQLLVVNLSGRGDKDIFTVHDILKARGE
I
>1FND
AQSVPYGVSQIKAPALHSQGYTGSNVKVAVIDSGIDSSHPDLKVAGGASFVPSETNPFQDNNSHGT
HVAGTVAALDNSIGVLGVAPSASLYAVKVLGADGSGQYSWIINGIEWAIANNMDVINMSLGGPSGS
AALKAAVDKAVASGVVVVAAAGNEGTSGSSSTVGYPAKYPSVIAVGAVDSSNQRASFSSVGPELDV

MAPGVSICSTLPGNKYGAKSGTSMASPHVAGAAALILSKHPNWTNTQVRSSLENTTTKLGDSFYYG
KGLINVQAAAQ
>1S01
TETTSFLITKFSPDQQNLIFQGDGYTTKEKLTLTKAVKNTVGRALYSSPIHIWDRETGNVANFVTS
FTFVINAPNSYNVADGFTFFIAPVDTKPQTGGGYLGVFNSAEYDKTTQTVAVEFDTFYNAAWDPSN
RDRHIGIDVNSIKSVNTKSWKLQNGEEANVVIAFNAATNVLTVSLTYPN
>2LTN
GLFGAIAGFIENGWEGMIDGWYGFRHQNSEGTGQAADLKSTQAAIDQINGKLNRVIEKTNEKFHQI
EKEFSEVEGRIQDLEKYVEDTKIDLWSYNAELLVALENQHTIDLTDSEMNKLFEKTRRQLRENAEE
MGNGCFKIYHKCDNACIESIRNGTYDHDVYRDEALNNRFQIKG
>3HMG
PICTNCCAGYKGCNYYSANGAFICEGQSDPKKPKACPLNCDPHIAYSKCPR
>7RSA
SISQQTVWNQMATVRTPLNFDSSKQSFCQFSVDLLGGGISVDKTGDWITLVQNSPISNLLRVAAWK
KGCLMVKVVMSGNAAVKRSDWASLVQVFLTNSNSTEHFDACRWTKSEPHSWELIFPIEVCGPNNGF
EMWSSEWANQTSWHLSFLVDNPKQSTTFDVLLGISQNFEIAGNTLMPAFSVPQANARSSENAESSA
>1BMV
ASYKVTLKTPDGDNVITVPDDEYILDVAEEEGLDLPYSCRAGACSTCAGKLVSGPAPDEDQSFLDD
DQIQAGYILTCVAYPTGDCVIETHKEEALY
>1FXI
AACKCDDEGPDIRTAPLTGTVDLGSCNAGWEKCASYYTIIADCCRKKK
>2LTN
KSPEELKGIFEKYAAKEGDPNQLSKEELKLLLQTEFPSLLKGPSTLDELFEELDKNGDGEVSFEEF
QVLVKKISQ
>3ICB
MDLLAELQWRGLVNQTTDEDGLRKLLNEERVTLYCGFDPTADSLHIGHLATILTMRRFQQAGHRPI
ALVGGATGLIGDPSGKKSERTLNAKETVEAWSARIKEQLGRFLDFEADGNPAKIKNNYDWIGPLDV
ITFLRDVGKHFSVNYMMAKESVQSRIETGISFTEFSYMMLQAYDFLRLYETEGCRLQIGGSDQWGN
ITAGLELIRKTKGEARAFGLTIPLVTKADGTKFGKTESGTIWLDKEKTSPYEFYQFWINTDDRDVI
RYLKYFTFLSKEEIEALEQELREAPEKRAAQKTLAEEVTKLVHGEEALRQAIRYA
>4TS1
ENLKLGFLVKQPEEPWFQTEWKFADKAGKDLGFEVIKIAVPDGEKTLNAIDSLAASGAKGFVICTP
DPKLGSAIVAKARGYDMKVIAVDDQFVNAKGKPMDTVPLVMLAATKIGERQGQELYKEMQKRGWDV
KESAVMAITANELDTARRRTTGSMDALKAAGFPEKQIYQVPTKSNDIPGAFDAANSMLVQHPEVKH
WLIVGMNDSTVLGGVRATEGQGFKAADIIGIGINGVDAVSELSKAQATGFYGSLLPSPDVHGYKSS
EMLYNWVAKDVEPPKFTEVTDVVLITRDNFKEELEKKGLGGK
>8ABP
METNLFKLSLDDVETPKGSMLDLKISQSKIALPKNTVGGTILRSDLLANFLTEGNFRASVDLQRTH
RIKGMIKMVATVGIPENTGIALACAMNSSIRGRASSDIYTICSQDCELWNPACTKAMTMSFNPNPC
SDAWSLEFLKRTGFHCDIICVTGWTATPMQDVQVTIDWFISSQECVPRTYCVLNPQNPFVLNRWMG
KLTFPQGTSRSVKRMPLSIGGGAGAKSAILMNMPNAVLSMWRYFVGDLVFEVSKMTSPYIKCTVSF
FIAFGNLADDTINFEAFPHKLVQFGEIQEKVVLKFSQEEFLTAWSTQVRPATTLLADGCPYLYAMV
HDSSVSTIPGDFVIGVKLTIIENMCAYGLNPGISGSRLLGTIPQ
>1BMV

GALTESQAALVKSSWEEFNANIPKHTHRFFILVLEIAPAAKDLFSFLKGTSEVPQNNPELQAHAGK
VFKLVYEAAIQLEVTGVVVTDATLKNLGSVHVSKGVADAHFPVVKEAILKTIKEVVGAKWSEELNS
AWTIAYDELAIVIKKEMDDAA
>1TGS
GNSTSSDKNNSSSEGNEGVIINNFYSNQYQNSIDLSANATGSDPPKTYGQFSNLLSGAVNAFSNML
PLLA
>2MEV
VQPTPADHFTFGLWTVGWTGADPFGVATRANLDPVEAVHKLAELGAYGITFHDNDLIPFDATAAER
EKILGDFNQALADTGLKVPMVTTNLFSHPVFKDGGFTSNDRSIRRFALAKVLHNIDLAAEMGAETF
VMWGGREGSEYDGSKDLAAALDRMREGVDTAAGYIKDKGYNLRIALEPKPNEPRGDIFLPTVGHGL
AFIEQLEHGDIVGLNPETGHEQMAGLNFTHGIAQALWAEKLFHIDLNGQRGIKYDQDLVFGHGDLT
SAFFTVDLLENGFPNGGPKYTGPRHFDYKPSRTDGYDGVWDSAKANMSMYLLLKERALAFRADPEV
QEAMKTSGVFELGETTLNAGESAADLMNDSASFAGFDAEAAAERNFAFIRLNQLAIEHLLGSR
>3PGM
STAGKVIKCKAAVLWEEKKPFSIEEVEVAPPKAHEVRIKMVATGICRSDDHVVSGTLVTPLPVIAG
HEAAGIVESIGEGVTTVRPGDKVIPLFTPQCGKCRVCKHPEGNFCLKNDLSMPRGTMQDGTSRFTC
RGKPIHHFLGTSTFSQYTVVDEISVAKIDAASPLEKVCLIGCGFSTGYGSAVKVAKVTQGSTCAVF
GLGGVGLSVIMGCKAAGAARIIGVDINKDKFAKAKEVGATECVNPQDYKKPIQEVLTEMSNGGVDF
SFEVIGRLDTMVTALSCCQEAYGVSVIVGVPPDSQNLSMNPMLLLSGRTWKGAIFGGFKSKDSVPK
LVADFMAKKFALDPLITHVLPFEKINEGFDLLRSGESIRTILTF
>8ADH
SAPANAVAADNATAIALKYNQDATKSERVAAARPGLPPEEQHCADCQFMQADAAGATDEWKGCQLF
PGKLINVNGWCASWTLKAG
>1TNF
ACDYTCGSNCYSSSDVSTAQAAGYKLHEDGETVGSNSYPHKYNNYEGFDFSVSSPYYEWPILSSGD
VYSGGSPGADRVVFNENNQLAGVITHTGASGNNFVECT
>2MHU
?GDVAKGKKTFVQKCAQCHTVENGGKHKVGPNLWGLFGRKTGQAEGYSYTDANKSKGIVWNNDTLM
EYLENPKKYIPGTKMIFAGIKKKGERQDLVAYLKSATS
>5CYT
GGGARSGDDVVAKYCNACHGTGLLNAPKVGDSAAWKTRADAKGGLDGLLAQSLSGLNAMPPKGTCA
DCSDDELKAAIGKMSGL
>9API
SAKELRCQCIKTYSKPFHPKFIKELRVIESGPHCANTEIIVKLSDGRELCLDPKENWVQRVVEKFL
KRAENS
>1UBQ
PSVYDAAAQLTADVKKDLRDSWKVIGSDKKGNGVALMTTLFADNQETIGYFKRLGNVSQGMANDKL
RGHSITLMYALQNFIDQLDNPDDLVCVVEKFAVNHITRKISAAEFGKINGPIKKVLASKNFGDKYA
NAWAKLVAVVQAAL
>2PCY
STGSATTTPIDSLDDAYITPVQIGTPAQTLNLDFDTGSSDLWVFSSETTASEVDGQTIYTPSKSTT
AKLLSGATWSISYGDGSSSSGDVYTDTVSVGGLTVTGQAVESAKKVSSSFTEDSTIDGLLGLAFST
LNTVSPTQQKTFFDNAKASLDSPVFTADLGYHAPGTYNFGFIDTTAYTGSITYTAVSTKQGFWEWT
STGYAVGSGTFKSTSIDGIADTGTTLLYLPATVVSAYWAQVSGAKSSSSVGGYVFPCSATLPSFTF

GVGSARIVIPGDYIDFGPISTGSSSCFGGIQSSAGIGINIFGDVALKAAFVVFNGATTPTLGFASK
>3SDH
SIPPEVKFNKPFVFLMIEQNTKSPLFMGKVVNPTQK
>5ER2
KKVVLGKKGDTVELTCTASQKKSIQFHWKNSNQIKILGNQGSFLTKGPSKLNDRADSRRSLWDQGN
FPLIIKNLKIEDSDTYICEVEDQKEEVQLLVFGLTANSDTHLLQGQSLTLTLESPPGSSPSVQCRS
PRGKNIQGGKTLSVSQLELQDSGTWTCTVLQNQKKVEFKIDIVVLA
>9API
PKYTIVDKETCIACGACGAAAPDIYDYDEDGIAYVTLDDNQGIVEVPDILIDDMMDAFEGCPTDSI
KVADEPFDGDPNKFE
>1CDH
ADLEDNMETLNDNLKVIEKADNAAQVKDALTKMRAAALDAQKATPPKLEDKSPDSPEMKDFRHGFD
ILVGQIDDALKLANEGKVKEAQAAAEQLKTTRNAYHQKTYRARG
>256B
MSKPQPIAAANWKCNGSQQSLSELIDLFNSTSINHDVQCVVASTFVHLAMTKERLSHPKFVIAAQN
AIAKSGAFTGEVSLPILKDFGVNWIVLGHSERRAYYGETNEIVADKVAAAVASGFMVIACIGETLQ
ERESGRTAVVVLTQIAAIAKKLKKADWAKVVIAYEPVWAIGTGKVATPQQAQEAHALIRSWVSSKI
GADVRGELRILYGGSVNGKNARTLYQQRDVNGFLVGGASLKPEFVDIIKATQ
>2PHH
PQITLWQRPLVTIKIGGQLKEALLDTGADDTVLEEMNLPGRWKPKMIGGIGGFIKVRQYDQILIEI
CGHKAIGTVLVGPTPVNIIGRNLLTQIGCTLNF
>9INS
MNIFEMLRIDEGLRLKIYKDTEGYYTIGIGHLLTKSPSLNAAKSELDKAIGRNCNGVITKDEAEKL
FNQDVDAAVRGILRNAKLKPVYDSLDAVRRCALINMVFQMGETGVAGFTNSLRMLQQKRWDEAAVN
LAKSRWYNQTANRAKRVITTFRTGTWDAYKNL
>1CDT
MFENITAAPADPILGLADLFRADERPGKINLGIGVYKDETGKTPVLTSVKKAEQYLLENETTKNYL
GIDGIPEFGRCTQELLFGKGSALINDKRARTAQTPGGTGALRVAADFLAKNTSVKRVWVSNPSWPN
HKSVFNSAGLEVREYAYYDAENHTLDFDALINSLNEAQAGDVVLFHGCCHNPTGIDPTLEQWQTLA
QLSVEKGWLPLFDFAYQGFARGLEEDAEGLRAFAAMHKELIVASSYSANFGLYNERVGACTLVAAD
SETVDRAFSQMKAAIRANYSNPPAHGASVVATILSNDALRAIWEQELTDMRQRIQRMRQLFVNTLQ
EKGANRDFSFIIKQNGMFSFSGLTKEQVLRLREEFGVYAVASGRVNVAGMTPDNMAPLCEAIVAVL
>2AAT
QAGLNSRALWQFNGMIKCKIPSSEPLLDFNNYGCYCGLGGSGTPVDDLDRCCQTHDNCYKQAKKLD
SCKVLVDNPYTNNYSYSCSNNEITCSSENNACEAFICNCDRNAAICFSKVPYNKEHKNLDKKNC
>2RSP
IPEYVDWRQKGAVTPVKNQGSCGSCWAFSAVVTIEGIIKIRTGNLNQYSEQELLDCDRRSYGCNGG
YPWSALQLVAQYGIHYRNTYPYEGVQRYCRSREKGPYAAKTDGVRQVQPYNQGALLYSIANQPVSV
VLQAAGKDFQLYRGGIFVGPCGNKVDHAVAAVGYGPNYILIKNSWGTGWGENGYIRIKRGTGNSYG
VCGLYTSSFYPVKN
>4BP2
TTCCPSIVARSNFNVCRLPGTPEAICATYTGCIIIPGATCPGDYAN
>5LDH
DIVMTQSPSSLSVSAGERVTMSCKSSQSLLNSGNQKNFLAWYQQKPGQPPKLLIYGASTRESGVPD

RFTGSGSGTDFTLTISSVQAEDLAVYYCQNDHSYPLTFGAGTKLEIKRADAAPTVSIFPPSSEQLT
SGGASVVCFLNNFYPKDINVKWKIDGSERQNGVLNSWTDQDSKDSTYSMSSTLTLTKDEYERHNSY
TCEATHKTSTSPIVKSFNRNEC
>1CRN
?ATKAVCVLKGDGPVQGTIHFEAKGDTVVVTGSITGLTEGDHGFHVHQFGDNTQGCTSAGPHFNPL
SKKHGGPKDEERHVGDLGNVTADKNGVAIVDIVDPLISLSGEYSIIGRTMVVHEKPDDLGRGGNEE
STKTGNAGSRLACGVIGIAK
>1MCP
GEVASVPLTNYLDSQYFGKIYLGTPPQEFTVLFDTGSSDFWVPSIYCKSNACKNHQRFDPRKSSTF
QNLGKPLSIHYGTGSMQGILGYDTVTVSNIVDIQQTVGLSTQEPGDVFTYAEFDGILGMAYPSLAS
EYSIPVFDNMMNRHLVAQDLFSVYMDRNGQESMLTLGAIDPSYYTGSLHWVPVTVQQYWQFTVDSV
TISGVVVACEGGCQAILDTGTSKLVGPSSDILNIQQAIGATQNQYGEFDIDCDNLSYMPTVVFEIN
GKMYPLTPSAYTSQDQGFCTSGFQSENHSQKWILGDVFIREYYSVFDRANNLVGLAKAI
>2ALP
KVFGRCELAAAMKRHGLDNYRGYSLGNWVCAAKFESNFNTQATNRNTDGSTDYGILQINSRWWCND
GRTPGSRNLCNIPCSALLSSDITASVNCAKKIVSDGNGMNAWVAWRNRCKGTDVQAWIRGCRL
>2SOD
?RCGEQGSNMECPNNLCCSQYGYCGMGGDYCGKGCQNGACWTSKRCGSQAGGATCPNNHCCSQYGH
CGFGAEYCGAGCQGGPCRADIKCGSQSGGKLCPNNLCCSQWGFCGLGSEFCGGGCQSGACSTDKPC
GKDAGGRVCTNNYCCSKWGSCGIGPGYCGAGCQSGGCDA
>9WGA
MAKQQNNRRKSATMRAVKRMINTHLEHKRFALINSGNTNATAGTVQNLSNGIIQGDDINQRSGDQV
RIVSHKLHVRGTAITVSQTFRFIWFRDNMNRGTTPTVLEVLNTANFMSQYNPITLQQKRFTILKDV
TLNCSLTGESIKDRIINLPGQLVNYNGATAVAASNGPGAIFMLQIGDSLVGLWDSSYEAVYTDA
>1MRT
ZZHADPICNKPCKTHDDCSGAWFCQACWNSARTCGPYV
>2BUK
ARSTNTFNYATYHTLDEIYDFMDLLVAQHPELVSKLQIGRSYEGRPIYVLKFSTGGSNRPAIWIDL
GIHSREWITQATGVWFAKKFTENYGQNPSFTAILDSMDIFLEIVTNPNGFAFTHSENRLWRKTRSV
TSSSLCVGVDANRNWDAGFGKAGASSSPCSETYHGKYANSEVEVKSIVDFVKNHGNFKAFLSIHSY
SQLLLYPYGYTTQSIPDKTELNQVAKSAVAALKSLYGTSYKYGSIITTIYQASGGSIDWSYNQGIK
YSFTFELRDTGRYGFLLPASQIIPTAQETWLGVLTIMEHTVNN
>4CPA
LAAVSVDCSEYPKPACPKDYRPVCGSDNKTYSNKCNFCNAVVESNGTLTLNHFGKC
>6CPA
ASPDWGYDDKNGPEQWSKLYPIANGNNQSPVDIKTSETKHDTSLKPISVSYNPATAKEIINVGHSF
HVNFEDNQDRSVLKGGPFSDSYRLFQFHFHWGSTNEHGSEHTVDGVKYSAELHVAHWNSAKYSSLA
EAASKADGLAVIGVLMKVGEANPKLQKVLDALQAIKTKGKRAPFTNFDPSTLLPSSLDFWTYPGSL
THPPLYESVTWIICKESISVSSEQLAQFRSLLSNVEGDNAVPMQHNNRPTQPLKGRTVRASF
>1OVO
XAFAGVLNDADIAAALEACKAADSFNHKAFFAKVGLTSKSADDVKKAFAIIDQDKSGFIEEDELKL
FLQNFKADARALTDGETKTFLKAGDSDGDGKIGVDEFTALVKA
>2W1VA
MSTFRLALIQLQVSSIKSDNLTRACSLVREAAKQGANIVSLPECFNSPYGTTYFPDYAEKIPGEST

QKLSEVAKESSIYLIGGSIPEEDAGKLYNTCSVFGPDGSLLVKHRKIHLFDIDVPGKITFQESKTL
SPGDSFSTFDTPYCKVGLGICYDMRFAELAQIYAQRGCQLLVYPGAFNLTTGPAHWELLQRARAVD
NQVYVATASPARDDKASYVAWGHSTVVDPWGQVLTKAGTEETILYSDIDLKKLAEIRQQIPILKQK
RADLYTVESKKP
>3OBIA
GMPHHQYVLTLSCPDRAGIVSAVSTFLFENGQNILDAQQYNDTESGHFFMRVVFNAAAKVIPLASL
RTGFGVIAAKFTMGWHMRDRETRRKVMLLVSQSDHCLADILYRWRVGDLHMIPTAIVSNHPRETFS
GFDFGDIPFYHFPVNKDTRRQQEAAITALIAQTHTDLVVLARYMQILSDEMSARLAGRCINIHHSF
LPGFKGAKPYHQAFDRGVKLIGATAHYVTSALDEGPIIDQDVERISHRDTPADLVRKGRDIERRVL
SRALHYHLDDRVILNGRKTVVFTD
>2BZUA
RIAISNSNRTRSVPSLTTIWSISPTPNCSIYETQDANLFLCLTKNGAHVLGTITIKGLKGALREMH
DNALSLKLPFDNQGNLLNCALESSTWRYQETNAVASNALTFMPNSTVYPRNKTAHPGNMLIQISPN
ITFSVVYNEINSGYAFTFKWSAEPGKPFHPPTAVFCYITEQGSHHHHHH
>1Q16A
MSKFLDRFRYFKQKGETFADGHGQLLNTNRDWEDGYRQRWQHDKIVRSTHGVNCTGSCSWKIYVKN
GLVTWETQQTDYPRTRPDLPNHEPRGCPRGASYSWYLYSANRLKYPMMRKRLMKMWREAKALHSDP
VEAWASIIEDADKAKSFKQARGRGGFVRSSWQEVNELIAASNVYTIKNYGPDRVAGFSPIPAMSMV
SYASGARYLSLIGGTCLSFYDWYCDLPPASPQTWGEQTDVPESADWYNSSYIIAWGSNVPQTRTPD
AHFFTEVRYKGTKTVAVTPDYAEIAKLCDLWLAPKQGTDAAMALAMGHVMLREFHLDNPSQYFTDY
VRRYTDMPMLVMLEERDGYYAAGRMLRAADLVDALGQENNPEWKTVAFNTNGEMVAPNGSIGFRWG
EKGKWNLEQRDGKTGEETELQLSLLGSQDEIAEVGFPYFGGDGTEHFNKVELENVLLHKLPVKRLQ
LADGSTALVTTVYDLTLANYGLERGLNDVNCATSYDDVKAYTPAWAEQITGVSRSQIIRIAREFAD
NADKTHGRSMIIVGAGLNHWYHLDMNYRGLINMLIFCGCVGQSGGGWAHYVGQEKLRPQTGWQPLA
FALDWQRPARHMNSTSYFYNHSSQWRYETVTAEELLSPMADKSRYTGHLIDFNVRAERMGWLPSAP
QLGTNPLTIAGEAEKAGMNPVDYTVKSLKEGSIRFAAEQPENGKNHPRNLFIWRSNLLGSSGKGHE
FMLKYLLGTEHGIQGKDLGQQGGVKPEEVDWQDNGLEGKLDLVVTLDFRLSSTCLYSDIILPTATW
YEKDDMNTSDMHPFIHPLSAAVDPAWEAKSDWEIYKAIAKKFSEVCVGHLGKETDIVTLPIQHDSA
AELAQPLDVKDWKKGECDLIPGKTAPHIMVVERDYPATYERFTSIGPLMEKIGNGGKGIAWNTQSE
MDLLRKLNYTKAEGPAKGQPMLNTAIDAAEMILTLAPETNGQVAVKAWAALSEFTGRDHTHLALNK
EDEKIRFRDIQAQPRKIISSPTWSGLEDEHVSYNAGYTNVHELIPWRTLSGRQQLYQDHQWMRDFG
ESLLVYRPPIDTRSVKEVIGQKSNGNQEKALNFLTPHQKWGIHSTYSDNLLMLTLGRGGPVVWLSE
ADAKDLGIADNDWIEVFNSNGALTARAVVSQRVPAGMTMMYHAQERIVNLPGSEITQQRGGIHNSV
TRITPKPTHMIGGYAHLAYGFNYYGTVGSNRDEFVVVRKMKNIDWLDGEGNDQVQESVK
>3CX5C
MAFRKSNVYLSLVNSYIIDSPQPSSINYWWNMGSLLGLCLVIQIVTGIFMAMHYSSNIELAFSSVE
HIMRDVHNGYILRYLHANGASFFFMVMFMHMAKGLYYGSYRSPRVTLWNVGVIIFILTIATAFLGY
CCVYGQMSHWGATVITNLFSAIPFVGNDIVSWLWGGFSVSNPTIQRFFALHYLVPFIIAAMVIMHL
MALHIHGSSNPLGITGNLDRIPMHSYFIFKDLVTVFLFMLILALFVFYSPNTLGHPDNYIPGNPLV
TPASIVPEWYLLPFYAILRSIPDKLLGVITMFAAILVLLVLPFTDRSVVRGNTFKVLSKFFFFIFV
FNFVLLGQIGACHVEVPYVLMGQIATFIYFAYFLIIVPVISTIENVLFYIGRVNK
>3I7QA
MFTGSIVAIVTPMDEKGNVCRASLKKLIDYHVASGTSAIVSVGTTGESATLNHDEHADVVMMTLDL
ADGRIPVIAGTGANATAEAISLTQRFNDSGIVGCLTVTPYYNRPSQEGLYQHFKAIAEHTDLPQIL

YNVPSRTGCDLLPETVGRLAKVKNIIGIREATGNLTRVNQIKELVSDDFVLLSGDDASALDFMQLG
GHGVISVTANVAARDMAQMCKLAAEGHFAEARVINQRLMPLHNKLFVEPNPIPVKWACKELGLVAT
DTLRLPMTPITDSGRETVRAALKHAGLL

>3P1GA

GSILAETHGTRPDLTDQPIPDADYTWYTDGSSFLQEGQRRAGAAVTTETEVIWARALPAGTSAQRA
ELIALTQALKMAEGKKLNVYTDSRYAFATAHVHSEGREIKNKNEILALLKALFLPKRLSIIHCPGH
QKGNSAEARGNRMADQAAREAAMKAVLETSTLL

>3EA6A

QGDIGIDNLRNFYTKKDFVDLKDVKDNDTPIANQLQFSNESYDLISESKDFNKFSNFKGKKLDVFG
ISYNGQCNTKYIYGGVTATNEYLDKSRNIPINIWINGNHKTISTNKVSTNKKFVTAQEIDVKLRKY
LQEEYNIYGHNGTKKGEEYGHKSKFYSGFNIGKVTFHLNNNDTFSYDLFYTGDDGLPKSFLKIYED
NKTVESEKFHLDVDISYKETI

>1EZ6A

ATSTKKLHKEPATLIKAIDGDTVKLMYKGQPMVFRLLLVDIPETKHPKKGVEKYGPEAAAFTKKMV
ENAKKIEVEFDKGQRTDKYGRGLAYIYADGKMVNEALVRQGLAKVAYVYKGNNTHEQLLRKAEAQA
KKEKLNIWSEDNADSGQ

>3GXBA

MVLDVAFVLEGSDKIGEADFNRSKEFMEEVIQRMDVGQDSIHVTVLQYSYMVTVEYPFSEAQSKGD
ILQRVREIRYQGGNRTNTGLALRYLSDHSFLVSQGDREQAPNLVYMVTGNPASDEIKRLPGDIQVV
PIGVGPNANVQELERIGWPNAPILIQDFETLPREAPDLVLQRCCSPHHHHHH

>1LG7A

VDEMDTHDPHQLRYEKFFFTVKMTVRSNRPFRTYSDVAAAVSHWDHMYIGMAGKRPFYKILAFLGS
SNLKATPAVLADQGQPEYHAHCEGRAYLPHRMGKTPPMLNVPEHFRRPFNIGLYKGTVELTMTIYD
DESLEAAPMIWDHFNSSKFSDFREKALMFGLIVEKKASGAWVLDSVSHFK

>2Y0GA

MAHHHHHHGHHHQLVSKGEELFTGVVPILVELDGDVNGHKFSVSGEGEGDATYGKLTLKFICTTGK
LPVPWPTLVTTLXVQCFSRYPDHMKQHDFFKSAMPEGYVQERTIFFKDDGNYKTRAEVKFEGDTLV
NRIELKGIDFKEDGNILGHKLEYNYNSHNVYIMADKQKNGIKVNFKIRHNIEDGSVQLADHYQQNT
PIGDGPVLLPDNHYLSTQSALSKDPNEKRDHMVLLEFVTAAGITLGMDELYK

>1K3UB

TTLLNPYFGEFGGMYVPQILMPALNQLEEAFVSAQKDPEFQAQFADLLKNYAGRPTALTKCQNITA
GTRTTLYLKREDLLHGGAHKTNQVLGQALLAKRMGKSEIIAETGAGQHGVASALASALLGLKCRIY
MGAKDVERQSPNVFRMRLMGAEVIPVHSGSATLKDACNEALRDWSGSYETAHYMLGTAAGPHPYPT
IVREFQRMIGEETKAQILDKEGRLPDAVIACVGGGSNAIGMFADFINDTSVGLIGVEPGGHGIETG
EHGAPLKHGRVGIYFGMKAPMMQTADGQIEESYSISAGLDFPSVGPQHAYLNSIGRADYVSITDDE
ALEAFKTLCRHEGIIPALESSHALAHALKMMREQPEKEQLLVVNLSGRGDKDIFTVHDILKARGEI

>3NYSA

MIEFIDLKNQQARIKDKIDAGIQRVLRHGQYILGPEVTELEDRLADFVGAKYCISCANGTDALQIV
QMALGVGPGDEVITPGFTYVATAETVALLGAKPVYVDIDPRTYNLDPQLLEAAITPRTKAIIPVSL
YGQCADFDAINAIASKYGIPVIEDAAQSFGASYKGKRSCNLSTVACTSFFPSAPLGCYGDGGAIFT
NDDELATAIRQIARHGQDRRYHHIRVGVNSRLDTLQAAILLPKLEIFEEEIALRQKVAAEYDLSLK
QVGIGTPFIEVNNISVYAQYTVRMDNRESVQASLKAAGVPTAVHYPIPLNKQPAVADEKAKLPVGD
KAATQVMSLPMHPYLDTASIKIICAALTNLEHHHHHH

>2A6ZA

GSDASKLSSDYSLPDLINTRKVPNNWQTGEQASLEEGRIVLTSNQNSKGSLWLKQGFDLKDSFTME
WTFRSVGYSGQTDGGISFWFVQDSNIPRDKQLYNGPVNYDGLQLLVDNNGPLGPTLRGQLNDGQKP
VDKTKIYDQSFASCLMGYQDSSVPSTIRVTYDLEDDNLLKVQVDNKVCFQTRKVRFPSGSYRIGVT
AQNGAVNNNAESFEIFKMQFFNGV

>1G72B

YDGQNCKEPGNCWENKPGYPEKIAGSKYDPKHDPVELNKQEESIKAMDARNAKRIANAKSSGNFVF
DVK

>1A8P

SNLNVERVLSVHHWNDTLFSFKTTRNPSLRFENGQFVMIGLEVDGRPLMRAYSIASPNYEEHLEFF
SIKVQNGPLTSRLQHLKEGDELMVSRKPTGTLVTSDLLPGKHLYMLSTGTGLAPFMSLIQDPEVYE
RFEKVVLIHGVRQVNELAYQQFITEHLPQSEYFGEAVKEKLIYYPTVTRESFHNQGRLTDLMRSGK
LFEDIGLPPINPQDDRAMICGSPSMLDESCEVLDGFGLKISPRMGEPGDYLIERAFVEK

>1A8Y

GLDFPEYDGVDRVINVNAKNYKNVFKKYEVLALLYHEPPEDDKASQRQFEMEELILELAAQVLEDK
GVGFGLVDSEKDAAVAKKLGLTEEDSIYVFKEDEVIEYDGEFSADTLVEFLLDVLEDPVELIEGER
ELQAFENIEDEIKLIGYFKNKDSEHYKAFKEAAEEFHPYIPFFATFDSKVAKKLTLKLNEIDFYEA
FMEEPVTIPDKPNSEEEIVNFVEEHRRSTLRKLKPESMYETWEDDMDGIHIVAFAEEADPDGYEFL
EILKSVAQDNTDNPDLSIIWIDPDDFPLLVPYWEKTFDIDLSAPQIGVVNVTDADSVWMEPSAEEL
EDWLEDVL

>1ABE

NLKLGFLVKQPEEPWFQTEWKFADKAGKDLGFEVIKIAVPDGEKTLNAIDSLAASGAKGFVICTPD
PKLGSAIVAKARGYDMKVIAVDDQFVNAKGKPMDTVPLVMMAATKIGERQGQELYKEMQKRGWDVK
ESAVMAITANELDTARRRTTGSMDALKAAGFPEKQIYQVPTKSNDIPGAFDAANSMLVQHPEVKHW
LIVGMNDSTVLGGVRATEGQGFKAADIIGIGINGVDAVSELSKAQATGFYGSLLPSPDVHGYKSSE
MLYNWVAKDVEPPKFTEVTDVVLITRDNFKEELEKKGLGGK

>1AIR

ATDTGGYAATAGGNVTGAVSKTATSMQDIVNIIDAARLDANGKKVKGGAYPLVITYTGNEDSLINA
AAANICGQWSKDPRGVEIKEFTKGITIIGANGSSANFGIWIKKSSDVVVQNMRIGYLPGGAKDGDM
IRVDDSPNVWVDHNELFAANHECDGTPDNDTTFESAVDIKGASNTVTVSYNYIHGVKKVGLDGSSS
SDTGRNITYHHNYYNDVNARLPLQRGGLVHAYNNLYTNITGSGLNVRQNGQALIENNWFEKAINPV
TSRYDGKNFGTWVLKGNNITKPADFSTYSITWTADTKPYVNADSWTSTGTFPTVAYNYSPVSAQCV
KDKLPGYAGVGKNLATLTSTAC

>1AL3

TWPDKGSLYVATTHTQARYALPGVIKGFIERYPRVSLHMHQGSPTQIAEAVSKGNADFAIATEALH
LYDDLVMLPCYHWNRSIVVTPEHPLATKGSVSIEELAQYPLVTYTFGFTGRSELDTAFNRAGLTPR
IVFTATDADVIKTYVRLGLGVGVIASMAVDPVSDPDLVKLDANGIFSHSTTKIGFRRSTFLRSYMY
DFIQRFAPHLTRDVVDTAVALRSNEDIEAMFKDIKLPEK

>1ALHA

MPVLENRAAQGDITAPGGARRLTGDQTAALRDSLSDKPAKNIILLIGDGMGDSEITAARNYAEGAG
GFFKGIDALPLTGQYTHYALNKKTGKPDYVTDSAASATAWSTGVKTYNGALGVDIHEKDHPTILEM
AKAAGLATGNVSTAELQDATPAALVAHVTSRKCYGPSATSEKCPGNALEKGGKGSITEQLLNARAD
VTLGGGAKTFAETATAGEWQGKTLREQAEARGYQLVSDAASLNSVTEANQQKPLLGLFADGNMPVR
WLGPKATYHGNIDKPAVTCTPNPQRNDSVPTLAQMTDKAIELLSKNEKGFFLQVEGASIDKQDHAA
NPCGQIGETVDLDEAVQRALEFAKKEGNTLVIVTANHAHASQIVAPDTKAPGLTQALNTKDGAVMV

MSYGNSEEDSQEHTGSQLRIAAYGPHAANVVGLTDQTDLFYTMKAALGLK
>1AMF
GKITVFAAASLTNAMQDIATQFKKEKGVDVVSSFASSSTLARQIEAGAPADLFISADQKWMDYAVD
KKAIDTATRQTLLGNSLVVVAPKASVQKDFTIDSKTNWTSLLNGGRLAVGDPEHVPAGIYAKEALQ
KLGAWDTLSPKLAPAEDVRGALALVERNEAPLGIVYGSDAVASKGVKVVATFPEDSHKKVEYPVAV
VEGHNNATVKAFYDYLKGPQAAEIFKRYGFTIK
>1AMK
SAKPQPIAAANWKCNGTTASIEKLVQVFNEHTISHDVQCVVAPTFVHIPLVQAKLRNPKYVISAEN
AIAKSGAFTGEVSMPILKDIGVHWVILGHSERRTYYGETDEIVAQKVSEACKQGFMVIACIGETLQ
QREANQTAKVVLSQTSAIAAKLTKDAWNQVVLAYEPVWAIGTGKVATPEQAQEVHLLLRKWVSENI
GTDVAAKLRILYGGSVNAANAATLYAKPDINGFLVGGASLKPEFRDIIDATR
>1AMX
TSSVFYYKTGDMLPEDTTHVRWFLNINNEKSYVSKDITIKDQIQGGQQLDLSTLNINVTGTHSNYY
SGQSAITDFEKAFPGSKITVDNTKNTIDVTIPQGYGSYNSFSINYKTKITNEQQKEFVNNSQAWYQ
EHGKEEVNGKSFNHTVHN
>1ARB
GVSGSCNIDVVCPEGDGRRDIIRAVGAYSKSGTLACTGSLVNNTANDRKMYFLTAHHCGMGTASTA
ASIVVYWNYQNSTCRAPNTPASGANGDGSMSQTQSGSTVKATYATSDFTLLELNNAANPAFNLFWA
GWDRRDQNYPGAIAIHHPNVAEKRISNSTSPTSFVAWGGGAGTTHLNVQWQPSGGVTEPGSSGSPI
YSPEKRVLGQLHGGPSSCSATGTNRSDQYGRVFTSWTGGGAAASRLSDWLDPASTGAQFIDGLDS
>1ARU
SVTCPGGQSTSNSQCCVWFDVLDDLQTNFYQGSKCESPVRKILRIVFHDAIGFSPALTAAGQFGGG
GADGSIIAHSNIELAFPANGGLTDTIEALRAVGINHGVSFGDLIQFATAVGMSNCPGSPRLEFLTG
RSNSSQPSPPSLIPGPGNTVTAILDRMGDAGFSPDEVVDLLAAHSLASQEGLNSAIFRSPLDSTPQ
VFDTQFYIETLLKGTTQPGPSLGFAEELSPFPGEFRMRSDALLARDSRTACRWQSMTSSNEVMGQR
YRAAMAKMSVLGFDRNALTDCSDVIPSAVSNNAAPVIPGGLTVDDIEVSCPSEPFPEIATASGPLP
SLAPAP
>1AT0
CFTPESTALLESGVRKPLGELSIGDRVLSTANGQAVYSEVILFDRNLEQQNFVQLHTDGGAVLTVT
PAHLVSVWQPESQKLTFVFADRIEEKNQVLVRDVETGELRPQRVVKVGSVRSKGVVAPLTREGTIV
VNSVAASCYA
>1AV4
ASPFRLASAGEISEVQGILRTAGLLGPEKRIAYLGVLDPARGAGSEAEDRRFRVFIHDVSGARPQE
VTVSVTNGTVISAVELDTAATGELPVLEEEFEVVEQLLATDERWLKALAARNLDVSKVRVAPLSAG
VFEYAEERGRRILRGLAFVQDFPEDSAWAHPVDGLVAYVDVVSKEVTRVIDTGVFPVPAEHGNYTD
PELTGPLRTTQKPISITQPEGPSFTVTGGNHIEWEKWSLDVGFDVREGVVLHNIAFRDGDRLRPII
NRASIAEMVVPYGDPSPIRSWQNYFDTGEYLVGQYANSLELGCDCLGDITYLSPVISDAFGNPREI
RNGICMHEEDWGILAKHSDLWSGINYTRRNRRMVISFFTTIGNDYGFYWYLYLDGTIEFEAKATGV
VFTSAFPEGGSDNISQLAPGLGAPFHQHIFSARLDMAIDGFTNRVEEEDVVRQTMGPGNERGNAFS
RKRTVLTRESEAVREADARTGRTWIISNPESKNRLNEPVGYKLHAHNQPTLLADPGSSIARRAAFA
TKDLWVTRYADDERYPTGDFVNQHSGGAGLPSYIAQDRDIDGQDIVVWHTFGLTHFPRVEDWPIMP
VDTVGFKLRPEGFFDRSPVLDVPAN
>1AYL
MRVNNGLTPQELEAYGISDVHDIVYNPSYDLLYQEELDPSLTGYERGVLTNLGAVAVDTGIFTGRS

PKDKYIVRDDTTRDTFWWADKGKGKNDNKPLSPETWQHLKGLVTRQLSGKRLFVVDAFCGANPDTR
LSVRFITEVAWQAHFVKNMFIRPSDEELAGFKPDFIVMNGAKCTNPQWKEQGLNSENFVAFNLTER
MQLIGGTWYGGEMKKGMFSMMNYLLPLKGIASMHCSANVGEKGDVAVFFGLSGTGKTTLSTDPKRR
LIGDDEHGWDDDGVFNFEGGCYAKTIKLSKEAEPEIYNAIRRDALLENVTVREDGTIDFDDGSKTE
NTRVSYPIYHIDNIVKPVSKAGHATKVIFLTADAFGVLPPVSRLTADQTQYHFLSGFTAKLAPTPT
FSACFGAAFLSLHPTQYAEVLVKRMQAAGAQAYLVNTGWNGTGKRISIKDTRAIIDAILNGSLDNA
ETFTLPMFNLAIPTELPGVDTKILDPRNTYASPEQWQEKAETLAKLFIDNFDKYTDTPAGAALVAA
GPKL

>1B51A

ADVPAGVQLADKQTLVRNNGSEVQSLDPHKIEGVPESNVSRDLFEGLLISDVEGHPSPGVAEKWEN
KDFKVWTFHLRENAKWSDGTPVTAHDFVYSWQRLADPNTASPYASYLQYGHIANIDDIIAGKKPAT
DLGVKALDDHTFEVTLSEPVPYFYKLLVHPSVSPVPKSAVEKFGDKWTQPANIVTNGAYKLKNWVV
NERIVLERNPQYWDNAKTVINQVTYLPISSEVTDVNRYRSGEIDMTYNNMPIELFQKLKKEIPNEV
RVDPYLCTYYYEINNQKAPFNDVRVRTALKLALDRDIIVNKVKNQGDLPAYSYTPPYTDGAKLVEP
EWFKWSQQKRNEEAKKLLAEAGFTADKPLTFDLLYNTSDLHKKLAIAVASIWKKNLGVNVNLENQE
WKTFLDTRHQGTFDVARAGWCADYNEPTSFLNTMLSDSSNNTAHYKSPAFDKLIADTLKVADDTQR
SELYAKAEQQLDKDSAIVPVYYYVNARLVKPWVGGYTGKDPLDNIYVKNLYIIKH

>1B6A

KVQTDPPSVPICDLYPNGVFPKGQECEYPEEKKALDQASEEIWNDFREAAEAHRQVRKYVMSWIKP
GMTMIEICEKLEDCSRKLIKENGLNAGLAFPTGCSLNNCAAHYTPNAGDTTVLQYDDICKIDFGTH
ISGRIIDCAFTVTFNPKYDTLLKAVKDATNTGIKCAGIDVRLCDVGEAIQEVMESYEVEIDGKTYQ
VKPIRNLNGHSIGQYRIHAGKTVPIVKGGEATRMEEGEVYAIETFGSTGKGVVHDDMECSHYMKNF
DVGHVPIRLPRTKHLLNVINENFGTLAFCRRWLDRLGESKYLMALKNLCDLGIVDPYPPLCDIKGS
YTAQFEHTILLRPTCKEVVSRGDDY

>1BB9

TTGRLDLPPGFMFKVQAQHDYTATDTDELQLKAGDVVLVIPFQNPEEQDEGWLMGVKESDWNQHKE
LEKCRGVFPENFTERVQ

>1BDB

MKLKGEAVLITGGASGLGRALVDRFVAEGAKVAVLDKSAERLAELETDHGDNVLGIVGDVRSLEDQ
KQAASRCVARFGKIDTLIPNAGIWDYSTALVDLPEESLDAAFDEVFHINVKGYIHAVKACLPALVA
SRGNVIFTISNAGFYPNGGGPLYTAAKHAIVGLVRELAFELAPYVRVNGVGSGGINSDLRGPSSLG
PLADMLKSVLPIGRMPEVEEYTGAYVFFATRGDAAPATGALLNYDGGLGVRGFFSGAGGNDLLEQL
NIH

>1BFD

ASVHGTTYELLRRQGIDTVFGNPGSNELPFLKDFPEDFRYILALQEACVVGIADGYAQASRKPAFI
NLHSAAGTGNAMGALSNAWNSHSPLIVTAGQQTRAMIGVEALLTNVDAANLPRPLVKWSYEPASAA
EVPHAMSRAIHMASMAPQGPVYLSVPYDDWDKDADPQSHHLFDRHVSSSVRLNDQDLDILVKALNS
ASNPAIVLGPDVDAANANADCVMLAERLKAPVWVAPSAPRCPFPTRHPCFRGLMPAGIAAISQLLE
GHDVVLVIGAPVFRYHQYDPGQYLKPGTRLISVTCDPLEAARAPMGDAIVADIGAMASALANLVEE
SSRQLPTAAPEPAKVDQDAGRLHPETVFDTLNDMAPENAIYLNESTSTTAQMWQRLNMRNPGSYYF
CAAGGLGFALPAAIGVQLAEPERQVIAVIGDGSANYSISALWTAAQYNIPTIFVIMNNGTYGALRW
FAGVLEAENVPGLDVPGIDFRALAKGYGVQALKADNLEQLKGSLQEALSAKGPVLIEVSTV

>1BG2

DLAECNIKVMCRFRPLNESEVNRGDKYIAKFQGEDTVVIASKPYAFDRVFQSSTSQEQVYNDCAKK

IVKDVLEGYNGTIFAYGQTSSGKTHTMEGKLHDPEGMGIIPRIVQDIFNYIYSMDENLEFHIKVSY
FEIYLDKIRDLLDVSKTNLSVHEDKNRVPYVKGCTERFVCSPDEVMDTIDEGKSNRHVAVTNMNEH
SSRSHSIFLINVKQENTQTEQKLSGKLYLVDLAGSEKVSKTGAEGAVLDEAKNINKSLSALGNVIS
ALAEGSTYVPYRDSKMTRILQDSLGGNCRTTIVICCSPSSYNESETKSTLLFGQRAKTI

>1BG6

SKTYAVLGLGNGGHAFAAYLALKGQSVLAWDIDAQRIKEIQDRGAIIAEGPGLAGTAHPDLLTSDI
GLAVKDADVILIVVPAIHHASIAANIASYISEGQLIILNPGATGGALEFRKILRENGAPEVTIGET
SSMLFTCRSERPGQVTVNAIKGAMDFACLPAAKAGWALEQIGSVLPQYVAVENVLHTSLTNVNAVM
HPLPTLLNAARCESGTPFQYYLEGITPSVGSLAEKVDAERIAIAKAFDLNVPSVCEWYPATIYEAV
QGNPAYRGIAGPINLNTRYFFEDVSTGLVPLSELGRAVNVPTPLIDAVLDLISSLIDTDFRKEGRT
LEKLGLSGLTAAGIRSAVE

>1CHD

LLSSEKLIAIGASTGGTEAIRHVLQPLPLSSPAVIITQHMPPGFTRSFAERLNKLCQISVKEAEDG
ERVLPGHAYIAPGDKHMELARSGANYQIKIHDGPPVNRHRPSVDVLFHSVAKHAGRNAVGVILTGM
GNDGAAGMLAMYQAGAWTIAQNEASCVVFGMPREAINMGGVSEVVDLSQVSQQMLAKISAGQAIRI

>1CIY

YTPIDISLSLTQFLLSEFVPGAGFVLGLVDIIWGIFGPSQWDAFLVQIEQLINQRIEEFARNQAIS
RLEGLSNLYQIYAESFREWEADPTNPALREEMRIQFNDMNSALTTAIPLLAVQNYQVPLLSVYVQA
ANLHLSVLRDVSVFGQRWGFDAATINSRYNDLTRLIGNYTDYAVRWYNTGLERVWGPDSRDWVRYN
QFRRELTLTVLDIVALFSNYDSRRYPIRTVSQLTREIYTNPVLENFDGSFRGMAQRIEQNIRQPHL
MDILNSITIYTDVHRGFNYWSGHQITASPVGFSGPEFAFPLFGNAGNAAPPVLVSLTGLGIFRTLS
SPLYRRIILGSGPNNQELFVLDGTEFSFASLTTNLPSTIYRQRGTVDSLDVIPPQDNSVPPRAGFS
HRLSHVTMLSQAAGAVYTLRAPTFSWQHRSAEFNNIIPSSQITQIPLTKSTNLGSGTSVVKGPGFT
GGDILRRTSPGQISTLRVNITAPLSQRYRVRIRYASTTNLQFHTSIDGRPINQGNFSATMSSGSNL
QSGSFRTVGFTTPFNFSNGSSVFTLSAHVFNSGNEVYIDRIEFVPAEVT

>1CLC

IETKVSAAKITENYQFDSRIRLNSIGFIPNHSKKATIAANCSTFYVVKEDGTIVYTGTATSMFDND
TKETVYIADFSSVNEEGTYYLAVPGVGKSVNFKIAMNVYEDAFKTAMLGMYLLRCGTSVSATYNGI
HYSHGPCHTNDAYLDYINGQHTKKDSTKGWHDAGDYNKYVVNAGITVGSMFLAWEHFKDQLEPVAL
EIPEKNNSIPDFLDELKYEIDWILTMQYPDGSGRVAHKVSTRNFGGFIMPENEHDERFFVPWSSAA
TADFVAMTAMAARIFRPYDPQYAEKCINAAKVSYEFLKNNPANVFANQSGFSTGEYATVSDADDRL
WAAAEMWETLGDEEYLRDFENRAAQFSKKIEADFDWDNVANLGMFTYLLSERPGKNPALVQSIKDS
LLSTADSIVRTSQNHGYGRTLGTTYYWGCNGTVVRQTMILQVANKISPNNDYVNAALDAISHVFGR
NYYNRSYVTGLGINPPMNPHDRRSGADGIWEPWPGYLVGGGWPGPKDWVDIQDSYQTNEIAINWNA
ALIYALAGFVNYN

>1CNV

DISSTEIAVYWGQREDGLLRDTCKTNNYKIVFISFLDKFGCEIRKPELELEGVCGPSVGNPCSFLE
SQIKECQRMGVKVFLALGGPKGTYSACSADYAKDLAEYLHTYFLSERREGPLGKVALDGIHFDIQK
PVDELNWDNLLEELYQIKDVYQSTFLLSAAPGCLSPDEYLDNAIQTRHFDYIFVRFYNDRSCQYST
GNIQRIRNAWLSWTKSVYPRDKNLFLELPASQATAPGGGYIPPSALIGQVLPYLPDLQTRYAGIAL
WNRQADKETGYSTNIIRYL

>1COT

DGDAAKGEKEFNKCKACHMIQAPDGTDIIKGGKTGPNLYGVVGRKIASEEGFKYGEGILEVAEKNP
DLTWTEADLIEYVTDPKPWLVKMTDDKGAKTKMTFKMGKNQADVVAFLAQNSPDA

>1CPO

EPGSGIGYPYDNNTLPYVAPGPTDSRAPCPALNALANHGYIPHDGRAISRETLQNAFLNHMGIANS
VIELALTNAFVVCEYVTGSDCGDSLVNLTLLAEPHAFEHDHSFSRKDYKQGVANSNDFIDNRNFDA
ETFQTSLDVVAGKTHFDYADMNEIRLQRESLSNELDFPGWFTESKPIQNVESGFIFALVSDFNLPD
NDENPLVRIDWWKYWFTNESFPYHLGWHPPSPAREIEFVTSASSAVLAASVTSTPSSLPSGAIGPG
AEAVPLSFASTMTPFLLATNAPYYAQDPTLGPND

>1CV8

NEQYVNKLENFKIRETQGNNGWCAGYTMSALLNATYNTNKYHAEAVMRFLHPNLQGQQFQFTGLTP
REMIYFGQTQGRSPQLLNRMTTYNEVDNLTKNNKGIAILGSRVESRNGMHAGHAMAVVGNAKLNNG
QEVIIIWNPWDNGFMTQDAKNNVIPVSNGDHYQWYSSIYGY

>1CVL

ADTYAATRYPVILVHGLAGTDKFANVVDYWYGIQSDLQSHGAKVYVANLSGFQSDDGPNGRGEQLL
AYVKQVLAATGATKVNLIGHSQGGLTSRYVAAVAPQLVASVTTIGTPHRGSEFADFVQDVLKTDPT
GLSSTVIAAFVNVFGTLVSSSHNTDQDALAALRTLTTAQTATYNRNFPSAGLGAPGSCQTGAATET
VGGSQHLLYSWGGTAIQPTSTVTGATDTSTGTLDVANVTDPSTLALLATGAVMINRASGQNDGLVS
RCSSLFGQVISTSYHWNHLDEINQLLGVRGANAEDPVAVIRTHVNRLKLQGV

>1CYO

SKAVKYYTLEEIQKHNNSKSTWLILHYKVYDLTKFLEEHPGGEEVLREQAGGDATENFEDVGHSTD
ARELSKTFIIGELHPDDRSKIT
